# Combined Experimental and Computational Investigations of Rhodium-Catalysed C–H Functionalisation of Pyrazoles with Alkenes

**DOI:** 10.1002/chem.201405550

**Published:** 2014-12-17

**Authors:** Andrés G Algarra, David L Davies, Qudsia Khamker, Stuart A Macgregor, Claire L McMullin, Kuldip Singh, Barbara Villa-Marcos

**Affiliations:** [a] Institute of Chemical Sciences, Heriot-Watt University Edinburgh EH14 4AS (UK) E-mail: s.a.macgregor@hw.ac.uk; [b] Department of Chemistry, University of Leicester Leicester LE1 7RH (UK) E-mail: dld3@le.ac.uk

**Keywords:** C–H activation, coupling reactions, density functional calculations, reaction mechanisms, rhodium

## Abstract

Detailed experimental and computational studies have been carried out on the oxidative coupling of the alkenes C_2_H_3_Y (Y=CO_2_Me (**a**), Ph (**b**), C(O)Me (**c**)) with 3-aryl-5-R-pyrazoles (R=Me (**1 a**), Ph (**1 b**), CF_3_ (**1 c**)) using a [Rh(MeCN)_3_Cp*][PF_6_]_2_/Cu(OAc)_2_**⋅**H_2_O catalyst system. In the reaction of methyl acrylate with **1 a**, up to five products (**2 aa**–**6 aa**) were formed, including the *trans* monovinyl product, either complexed within a novel Cu^I^ dimer (**2 aa**) or as the free species (**3 aa**), and a divinyl species (**6 aa**); both **3 aa** and **6 aa** underwent cyclisation by an aza-Michael reaction to give fused heterocycles **4 aa** and **5 aa**, respectively. With styrene, only *trans* mono- and divinylation products were observed, whereas with methyl vinyl ketone, a stronger Michael acceptor, only cyclised oxidative coupling products were formed. Density functional theory calculations were performed to characterise the different migratory insertion and β-H transfer steps implicated in the reactions of **1 a** with methyl acrylate and styrene. The calculations showed a clear kinetic preference for 2,1-insertion and the formation of *trans* vinyl products, consistent with the experimental results.

## Introduction

Metal-catalysed C–H functionalisations are now widely studied as atom-efficient methods for the construction of C–C and C–E (E=O, N) bonds.[1] These circumvent the requirement to prefunctionalise the C–H bond and hence avoid the formation of salt waste in the subsequent C–C or C–E bond-formation reaction. Substrates with nitrogen- or oxygen-based directing groups are particularly efficient in this regard and the recognition of the role of carboxylates in providing facile C–H activation[2] has led to a huge growth in the application of these methods in organic synthesis. The C–H activation occurs through a synergic process involving the Lewis acidic metal centre and an intramolecular carboxylate base, emphasised in the term “ambiphilic metal–ligand assistance” (AMLA) put forward by us,[2d, e] whereas Fagnou and co-workers[2j] have termed this “concerted metallation–deprotonation” (CMD).

**Scheme 1:**
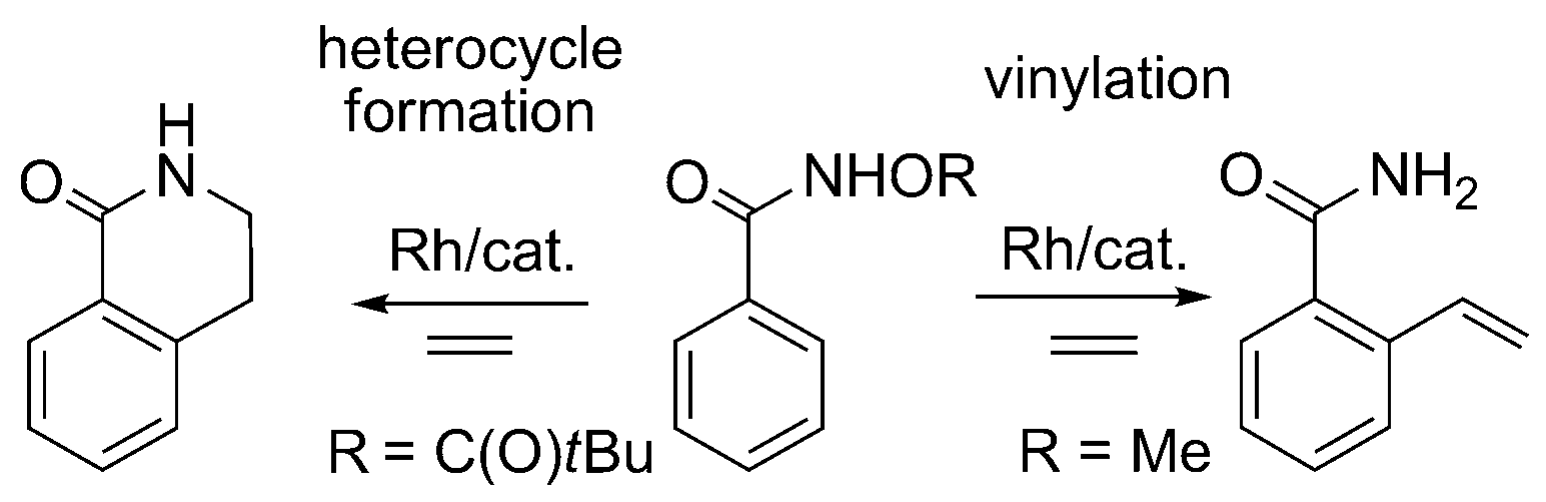
Alternative outcomes of the rhodium-catalysed coupling reactions of ethene and benzamides.

Over the last few years, Rh^III^ catalysts for C–H activation and functionalisation based on [RhCl_2_Cp*]_2_ and related derivatives have been intensively studied and the field has recently been reviewed.[1g, h] Reactions with alkynes often proceed by insertion into a cyclometallated intermediate followed by C,E reductive elimination to generate heterocycles. In contrast, with alkenes, insertion into a cyclometallated intermediate is usually followed by β-H elimination to form a vinyl species that dissociates from the metal. Exceptions to this can occur through the use of internal oxidants, as shown independently by Fagnou[3] and Glorius[4] and their co-workers for the reactions of alkenes and benzamides (Scheme 1). Thus, with R=C(O)*t*Bu, N–C(sp^3^) bond formation leads to a saturated heterocycle in preference to the alternative β-H elimination product observed when R=Me.

There are now numerous stoichiometric precedents for the C–H activation step at complexes based on the {RhCp*} fragment[5] and in some cases for the subsequent alkene insertions.[5g] However, in catalytic reactions intermediates are often not detected and so information on the accessibility of the various steps involved is difficult to establish. One means to gain mechanistic insight is to use density functional theory (DFT) calculations to complement experimental observations. We[6] and others[7] have investigated reactions with alkynes and DFT studies on related coupling reactions with alkenes have also appeared.[8] Xia and co-workers studied dihydroquinolone formation through the coupling of PhC(O)NH(OR) (R=Me, C(O)*t*Bu) and ethene at Rh(OAc)_2_Cp* and invoked a Rh(V)–nitrene intermediate to account for the role of the internal oxidant.[8b] Fu and co-workers also considered the oxidative Heck coupling of phenol carbamates with ethyl acrylate.[8a]

Herein we report the catalytic C–H functionalisation of 3-phenylpyrazoles with alkenes at a {RhCp*} centre. In particular, we have extended the range of alkenes as coupling partners and shown that [Rh(MeCN)_3_Cp*][PF_6_]_2_ is a more efficient catalyst precursor than [RhCl_2_Cp*]_2_. In addition, we have isolated a range of mono- and divinylation products, which lend greater insight into the underpinning mechanism of these reactions. DFT calculations have also been employed to probe the mechanism further and to understand the factors controlling product selectivity. Related studies involving the coupling of alkenes with N-heterocycles with a {RhCp*} catalyst include the vinylation of *N*-phenylpyrazole with styrenes and acrylates,[9] the oxidative vinylation and subsequent aza-Michael cyclisation of pyridineamides with alkenes featuring electron-withdrawing substituents,[10] as well as similar reactions of 3-phenylpyrazoles with ethyl or butyl acrylate.[11]

## Results and Discussion

### Catalysis studies

**Scheme 2:**
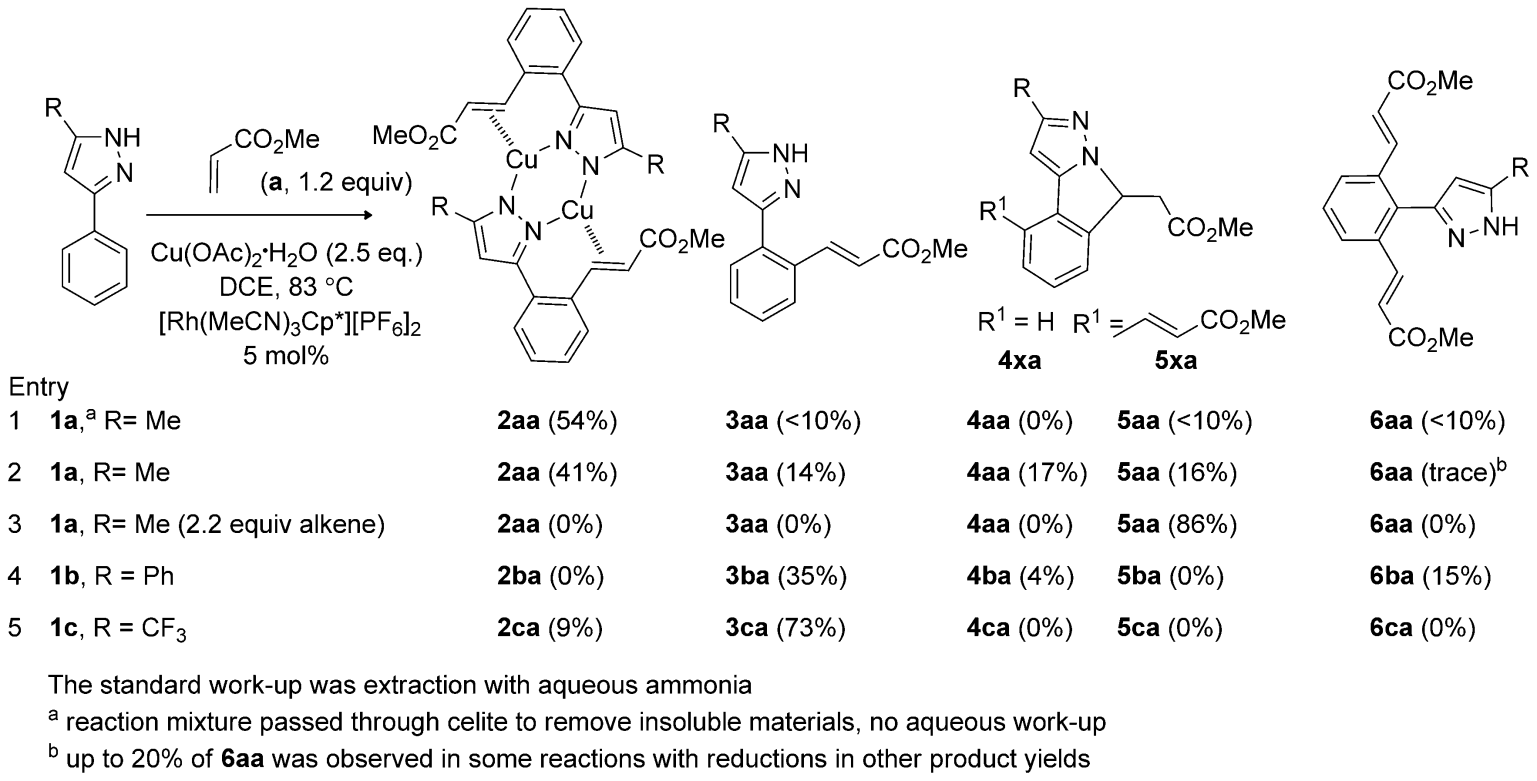
Products from the reactions of 3-phenylpyrazoles with methyl acrylate (yields of isolated product are given in parentheses).[12, 16]

We have examined the reactions of 3-phenyl-5-R-pyrazoles (R=Me (**1 a**), Ph (**1 b**), CF_3_ (**1 c**)) with methyl acrylate (**a**), styrene (**b**) and methyl vinyl ketone (**c**) catalysed by [Rh(MeCN)_3_Cp*][PF_6_]_2_. The reaction of **1 a** with methyl acrylate (**a**) led to a mixture of products, as summarised in Scheme 2.[12] It became apparent that the work-up procedure affected the product distribution. If the reaction mixture was simply passed through Celite three times to remove insoluble inorganic materials, one major product was formed (**2 aa**, entry 1) with minor amounts of three others (**3 aa**, **5 aa** and **6 aa**). The ^1^H NMR spectrum for the major component is consistent with a monovinyl product and shows two mutually coupled 1 H doublets at *δ*=5.06 and 6.47 ppm (*J*=13.7 Hz). This large coupling constant is on the boundary between those of *cis* and *trans* vinyl groups and so the precise identification of this compound from the NMR data alone was not possible.[13] If the reaction mixture was extracted with aqueous ammonia (2 m) to remove any soluble copper species, then **3 aa**, **5 aa** and **6 aa** were again observed along with a new product denoted **4 aa** (entry 2). Further investigation showed that if the major product from entry 1 was isolated and then treated with aqueous ammonia it converted into a mixture of **3 aa** and **4 aa**.

**Figure 1:**
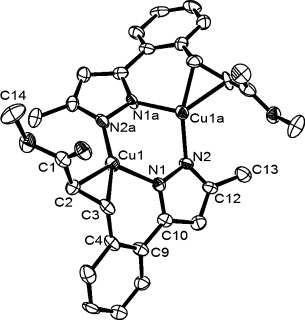
Molecular structure of one molecule of 2 aa. Hydrogen atoms have been omitted for clarity; ellipsoids are set at 50 % probability.

Crystallisation of the initial major product **2 aa** allowed its molecular structure to be determined by X-ray crystallography. Surprisingly, the compound turned out to be a copper(I) dimer with bridging pyrazoles and one alkene bound to each copper (Figure 1). Compared with the free alkene **3 aa** (see below), the protons and carbons in **2 aa** are shifted upfield (1.3–1.6 ppm ^1^H, and 24–50 ppm ^13^C), consistent with some back-bonding component.[14] In accord with this, the C=C distances are relatively long (1.377(12) and 1.410(13) Å in two independent molecules) compared with other copper(I)–alkene complexes.

The other products formed after aqueous extraction were much easier to identify. Compound **3 aa** is formed by simple displacement of the vinyl-pyrazole ligands from copper in **2 aa** by ammonia.[15] The ^1^H NMR spectrum for **3 aa** is very similar to **2 aa** except the vinyl protons are observed considerably downfield at *δ*=6.39 and 8.13 ppm with a coupling of 16.0 Hz, that is, 2–3 Hz larger than that of the copper dimer **2 aa**. The HRMS of compound **4 aa** shows the same mass as **3 aa** and the ^1^H NMR spectrum shows three mutually coupled doublets of doublets at *δ*=5.49, 3.30 and 2.77 ppm, each integrating to one proton. Analysis of the ^13^C NMR and HMQC spectra showed that these signals correspond to a CHCH_2_ group. The HMBC spectrum shows a correlation between the CH_2_ and the CO_2_Me, consistent with the CH_2_ being next to the CO_2_Me. This rules out a six-membered saturated N-heterocyclic product formed by C(sp^3^)–N coupling (see also the Computational Studies section below). Based on these data and the crystal structure of the related compound **4 ba** (see Figure S1 in the Supporting Information), the structure was deduced to be the five-membered heterocyclic compound **4 aa**, formed from **3 aa** by an intramolecular Michael reaction (see below).

The fourth product, **5 aa**, arises from the reaction of **1 a** with 2 equivalents of methyl acrylate and exhibits a vinyl coupling constant of *J*=16.0 Hz, which again suggests a *trans* vinyl. If 2.2 equivalents of methyl acrylate were used, **5 aa** was the only product formed, in a yield of 86 %. On some occasions a fifth product, divinyl **6 aa**, was also observed. The ^1^H NMR spectrum of **6 aa** shows two mutually coupled doublets at *δ*=6.26 and 7.59 ppm (*J*=15.7 Hz) corresponding to four vinyl protons; the low-field shift and large coupling constant both suggest *trans* vinyl groups. This compound was unstable in solution and readily isomerised to **5 aa** within 2 days through a non-catalysed Michael addition. Compound **5 aa** is analogous to the aza-Michael cyclisation products reported by Li and Zhao,[11] but we have now been able to demonstrate that these are formed directly from a divinyl precursor. The cyclisation of **6 aa** to **5 aa** is evidently easier than the cyclisation of **3 aa** to **4 aa**, as these monovinyl compounds are stable for more than a week in solution (see below). All of the compounds observed from the reaction of **1 a** with methyl acrylate result from the regioselective insertion of the alkene with the CO_2_Me substituent placed adjacent to the metal and selective β-H elimination to provide *trans* vinyl compounds (see the Computational Studies section below).

A similar reaction between **1 b** and methyl acrylate (**a**) gave the corresponding monovinyl (**3 ba**), cyclised (**4 ba**) and divinyl (**6 ba**) products in a combined yield of 54 % after chromatography.[17] The low-field shifts (*δ*=6.43 and 8.11 ppm) and large coupling constant (ca. 16 Hz) of the vinyl protons in **3 ba** suggest a *trans* geometry, and in this case no copper complex (**2 ba**) was observed. Similarly, the divinyl product **6 ba** shows a *trans* geometry. Crystals of **4 ba** were obtained and its molecular structure determined by X-ray diffraction (see Figure S1 in the Supporting Information). The structure clearly shows that 1 equivalent of methyl acrylate reacted with **1 b** and that an intramolecular Michael reaction has occurred to form a new five-membered ring. The reaction of **1 c** with methyl acrylate (**a**) gave monovinyl product **3 ca** in a yield of 73 % along with a small amount (9 %) of the copper complex **2 ca**. In this case no cyclised product **4 ca** was observed.

**Scheme 3:**
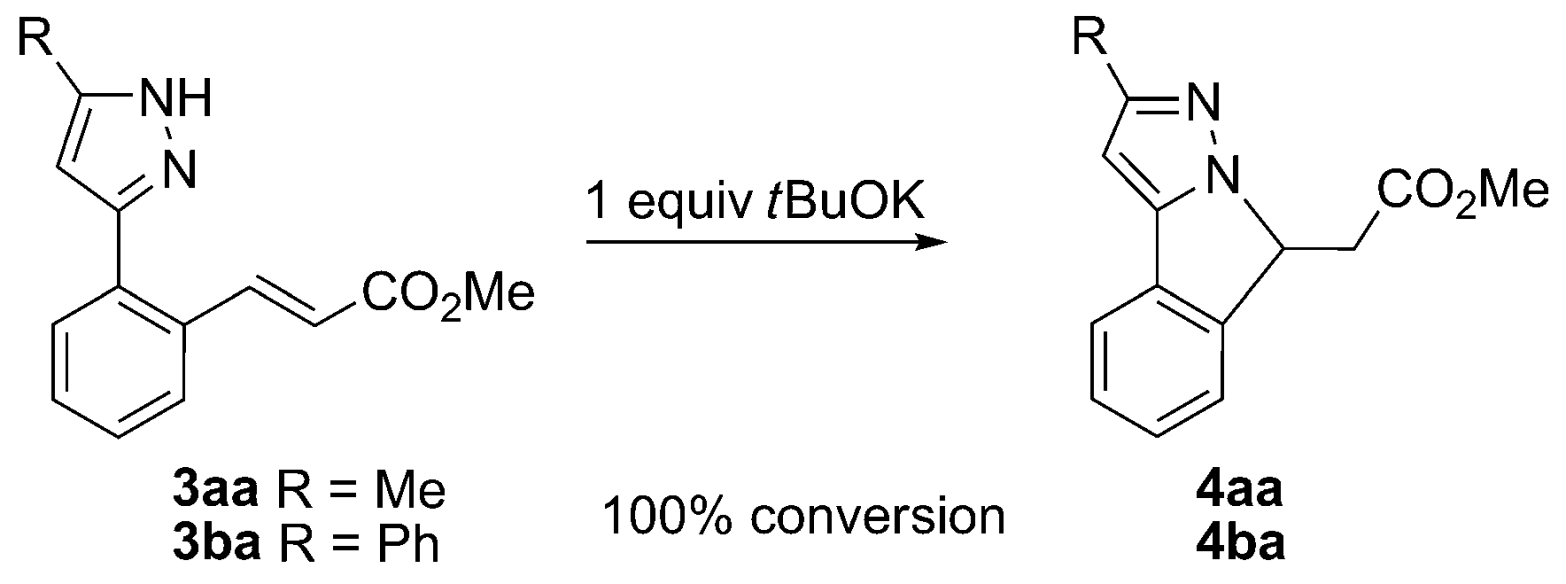
Base-catalysed isomerisation of 3 aa and 3 ba.

The cyclisations of **3 aa** and **3 ba** were attempted by adding base (Scheme 3). For **3 aa** no reaction occurred in the presence of the weak base K_2_CO_3_, but addition of *t*BuOK led almost immediately to the formation of **4 aa** (as shown by ^1^H NMR spectroscopy); the equivalent cyclisation also occurred with **3 ba** to give **4 ba**. It is therefore likely that the aqueous ammonia used to remove the copper acts as a base catalyst for the formation of the cyclisation products observed in the original catalytic reactions. Although the Michael addition cyclisations of the initially formed vinyl products have been observed before, the role of the work-up procedure in forming such products has not been recognised previously.

**Scheme 4:**
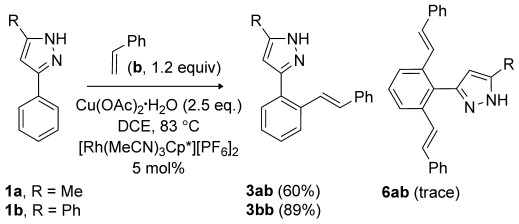
Products from the reactions of 3-phenylpyrazoles with styrene (yields of isolated product are given in parentheses).

We have previously shown that the cationic catalyst [Rh(MeCN)_3_Cp*][PF_6_]_2_ is more reactive than [RhCl_2_Cp*]_2_ in the reactions of pyrazoles with alkynes,[6] and so the reactions of substrates **1** with styrene (**b**), an alkene that has not previously undergone coupling with 3-phenylpyrazoles,[11] were attempted (Scheme 4).

The reaction between **1 a** and styrene worked well and gave mainly the monovinyl product **3 ab** in an overall yield of 60 %. The geometry of the vinyl group was assigned as *trans* based on the chemical shifts and coupling constant. As for methyl acrylate, insertion of styrene is regioselective and occurs such that the phenyl substituent is adjacent to the metal. A minor amount of a product believed to be the divinyl **6 ab** was also isolated. As found in the reaction of **1 a** and methyl acrylate, the products formed with styrene are exclusively *trans*. The reaction of diphenylpyrazole (**1 b**) with styrene gave only the monovinyl product **3 bb** in a yield of 89 %, again as the *trans* isomer. Unlike methyl acrylate, no cyclised products were formed with styrene, consistent with the fact that styrene is a poorer Michael acceptor.

**Scheme 5:**
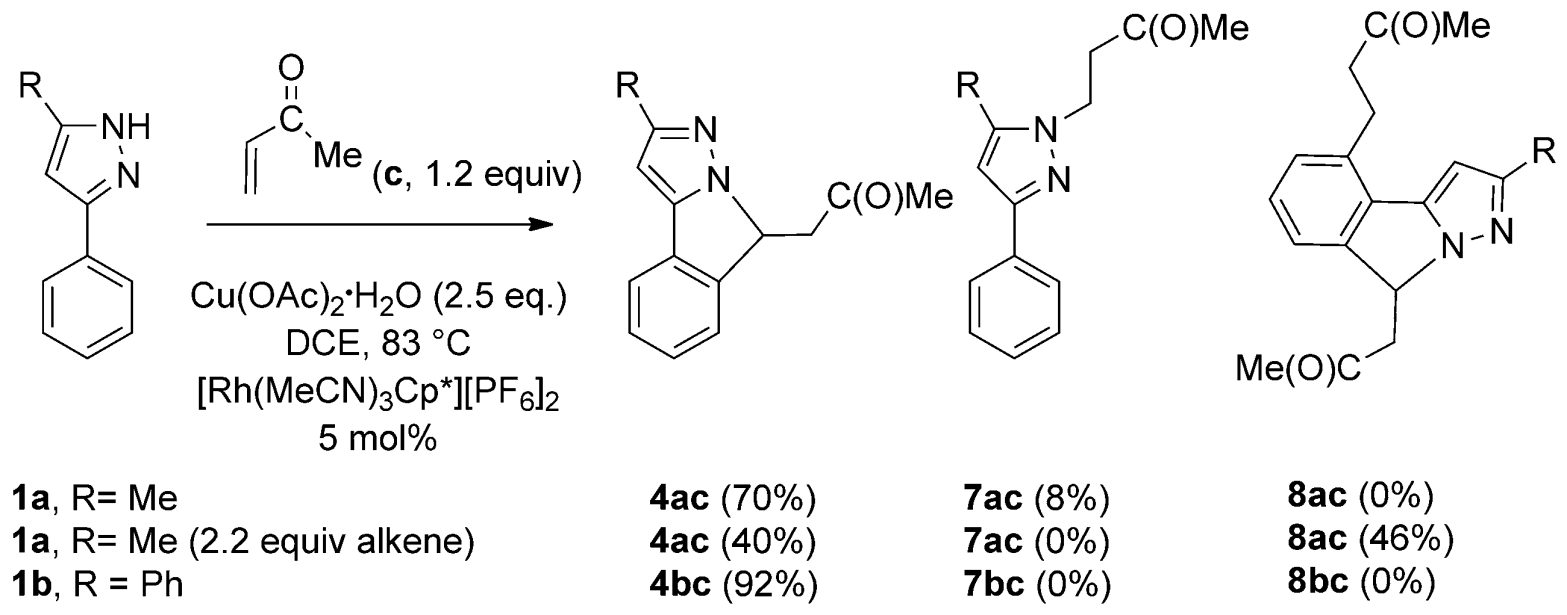
Products from the reactions of 3-phenylpyrazoles with methyl vinyl ketone (yields of isolated product are given in parentheses).

Reactions with the better Michael acceptor methyl vinyl ketone (**c**) were then attempted (Scheme 5). With **1 a**, the tricyclic compound **4 ac** was formed as the major product (70 %) and the simple Michael addition compound **7 ac** as a minor product (8 %). The ^1^H NMR spectrum of **7 ac** shows two 2 H triplets at *δ*=3.06 and 4.24 ppm, the latter shows an NOE to the methyl signal of the pyrazole, but does not show an NOE to any of the phenyl protons, hence the alkyl chain is proposed to be on the nitrogen further away from the phenyl ring. The reaction of **1 b** with methyl vinyl ketone gave solely **4 bc** in high yield (92 %). Products **4 ac** and **4 bc** presumably arise from the initial formation of a monovinyl species that cyclises by an aza-Michael reaction before the second C–H activation and insertion can occur. The failure to observe any monovinyl product is also consistent with the aza-Michael reaction being more favoured with methyl vinyl ketone than with methyl acrylate, as is the formation of a minor amount of the direct Michael addition product **7 ac**.

To determine whether divinylation was possible, the reaction of **1 a** was repeated with 2.2 equivalents of methyl vinyl ketone. This gave two products in a roughly 1:1 ratio and combined yield of 86 %. The first product was **4 ac** (40 %), as found in the reaction with just 1.2 equivalents of methyl vinyl ketone. However, the second product was not the expected monocyclised monovinyl (c.f. **5 aa** found with methyl acrylate), but the related product **8 ac** formed in a yield of 46 %, in which the vinyl group of the expected **5 ac** has been hydrogenated, as confirmed by the mass spectrum. The mechanism of formation of **8 ac** is uncertain. It seems unlikely to arise from **4 ac** because there is no directing group available for the second C–H activation and subsequent addition of the second alkene group. At this stage we cannot say whether hydrogenation occurs before the addition of the second alkene and cyclisation or whether it occurs after the formation of a product of type **5**. In contrast to the reaction with 1.2 equivalents of alkene, excess alkene leads to less competition with the direct aza-Michael addition and so no **7 ac** was formed.

To assess the role of rhodium and copper in the formation of **7 ac**, the reaction of **1 a** with 1.2 equivalents of methyl vinyl ketone was repeated in the absence of [Rh(MeCN)_3_Cp*][PF_6_]_2_ and Cu(OAc)_2_**⋅**H_2_O. This gave **7 ac** as the sole product in a yield of 94 % isolated product in the same period of time, which confirms that **7 ac** is formed by Michael addition without the need for rhodium or copper.

### Computational studies

DFT calculations have been used to probe the mechanism and selectivity of the coupling reactions between 3-phenyl-5-methylpyrazole (**1 a**) and alkenes C_2_H_3_Y in which Y=CO_2_Me (**a**) and Ph (**b**). Previous work on catalytic heterocycle formation between **1 a** and alkynes has shown the importance of including corrections for both solvent and dispersion effects in the computed energetics.[6] A similar approach was adopted in this work, with geometries initially optimised in the gas phase with the BP86 functional and a medium-sized basis set (BS1) and the resultant free energies then being corrected for solvation (dichloroethane (DCE), polarizable continuum model (PCM) approach), dispersion (Grimme’s D3 parameter set) and basis-set effects (with an extended basis set, BS2). The final energies computed by using this protocol are referred to as *G*_DCE_ (see the Supporting Information for full details).

**Scheme 6:**
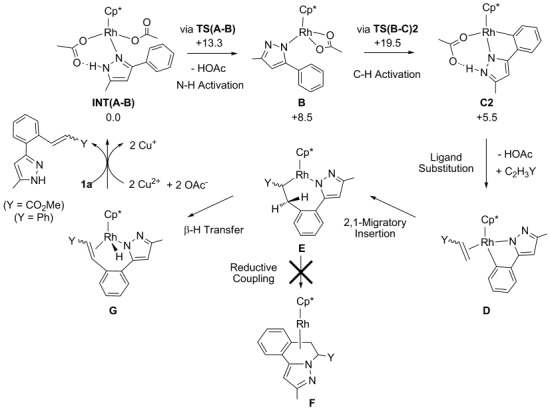
Mechanism for the coupling of 3-phenyl-5-methylpyrazole (1 a) and alkenes C_2_H_3_Y (Y=CO_2_Me (a), Ph (b)) at Rh(OAc)_2_Cp*, illustrated for the 2,1-insertion pathway to give *trans* or *cis* vinylation products.

The key steps in the overall catalytic cycle are summarised in Scheme 6. Under the catalytic conditions we expect Rh(OAc)_2_Cp* to be formed from [Rh(MeCN)_3_Cp*][PF_6_]_2_ and Cu(OAc)_2_**⋅**H_2_O and we have previously shown[6] that this readily forms an *N*-bound adduct with **1 a**. This species, **Int(A-B)**, is the most stable precursor to the subsequent coupling reaction and so all energies are quoted relative to this (and the relevant free alkene) at 0.0 kcal mol^−1^. Sequential N–H and C–H activation of **1 a** in **Int(A-B)** leads to cyclometallated **C2** and the computed energetics of these steps from our earlier work are also shown in Scheme 6.[18] Substitution of acetic acid in **C2** by an alkene gives intermediate **D** in which migratory insertion into the Rh–aryl bond leads to seven-membered metallacycle **E**. In principle, reductive coupling in **E** could give **F** in which a heterocyclic product is bound to rhodium. However, in this case such a C(sp^3^)–N bond formation is disfavoured and instead **E** undergoes β-H transfer to give **G** from which the final C–C coupled product can be displaced by additional **1 a**. This, along with the re-oxidation of the Rh^I^ metal centre by Cu(OAc)_2_, regenerates the catalytically active Rh^III^ species and so completes the cycle.

Within this mechanistic picture, the regio- and stereoselectivity of the reaction are dictated by the migratory insertion and β-H transfer steps, respectively. With the monosubstituted alkenes C_2_H_3_Y (Y=CO_2_Me, Ph), four migratory insertion processes are possible that differ according to the orientation of the alkene in intermediate **D**. Two pathways follow the 2,1 insertion pathway illustrated in Scheme 6 that places the substituent Y beside the metal in metallacycle **E**. β-H transfer then gives 1,2-disubstituted alkenes with potentially either a *trans* or *cis* stereochemistry.

The alternative 1,2-insertions place Y beside the aryl ring in **E** and would give a 1,1-disubstituted alkene, although only 1,2-disubstituted products have been observed experimentally. In the following we consider the details of these processes, first for methyl acrylate (**a**) and then styrene (**b**), both coupling with 3-phenyl-5-methylpyrazole (**1 a**).

#### Reaction with methyl acrylate: Migratory insertion

**Figure 2:**
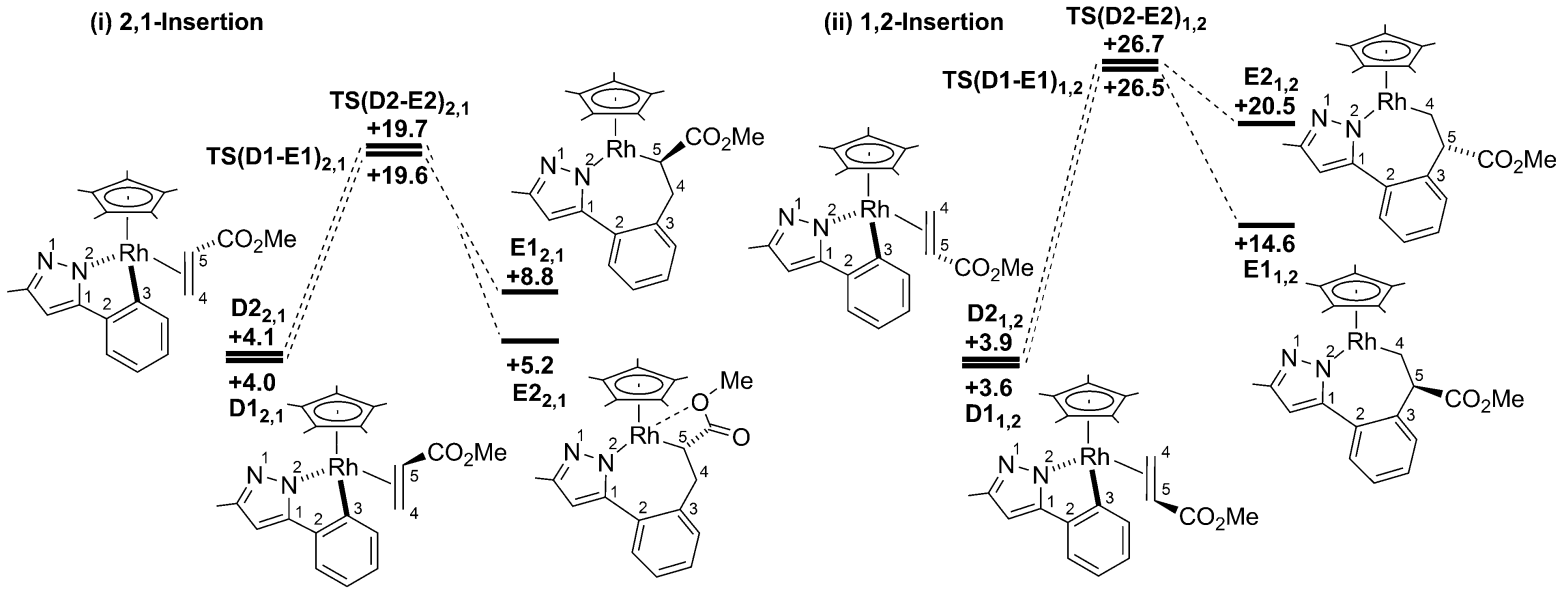
Computed energy profiles (*G*_DCE_, kcal mol^−1^) for the migratory insertion of methyl acrylate into adduct D by i) 2,1-insertion and ii) 1,2-insertion. Energies (*G*_DCE_) are quoted relative to Int(A-B) and free methyl acrylate set to 0.0 kcal mol^−1^.

**Figure 3:**
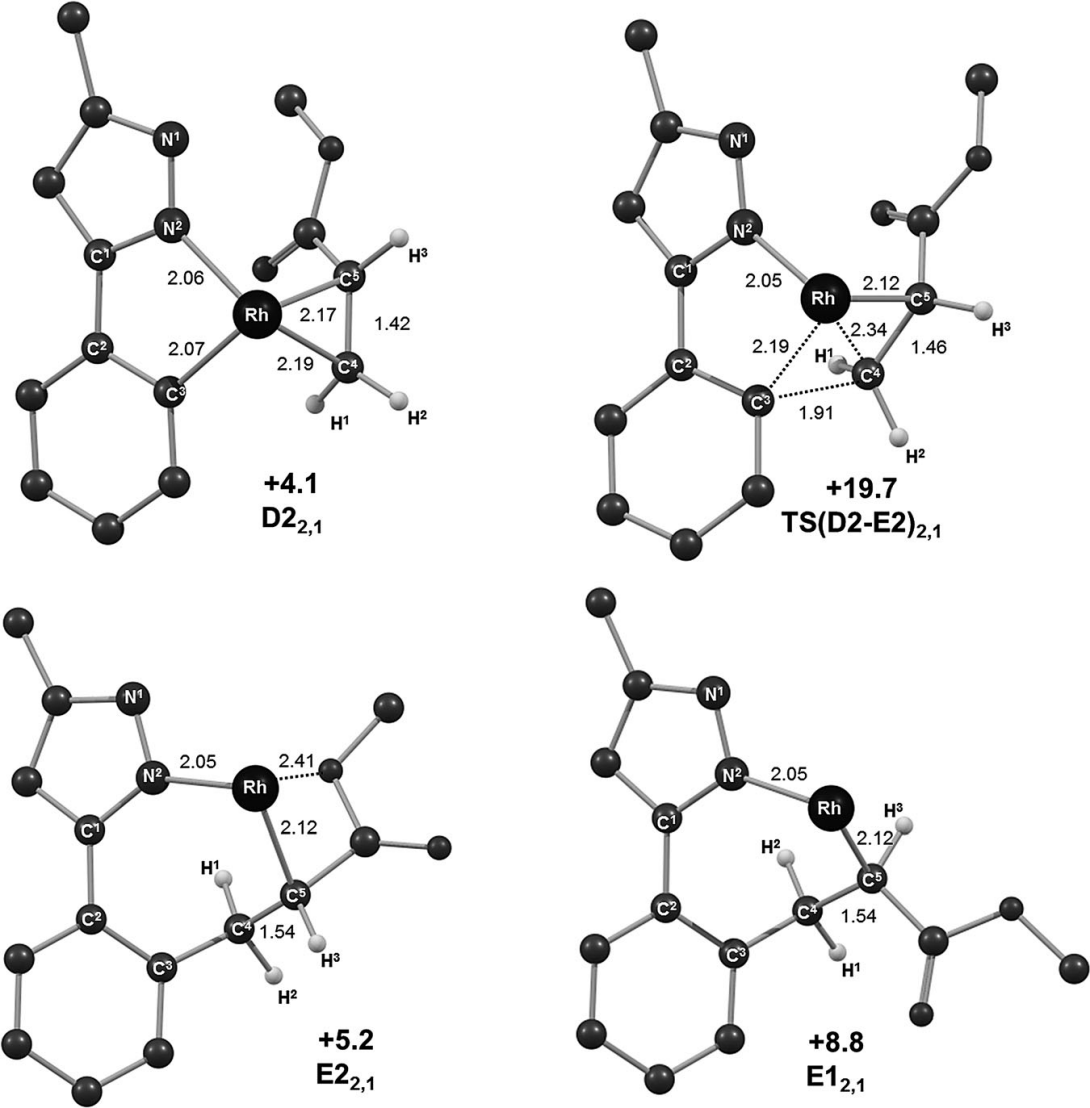
Computed structures for 2,1-insertion starting from D2_2,1_ with relative energies in kcal mol^−1^ and selected distances in Å. The geometry of E1_2,1_ is included for comparison. The Cp* ligand (which lies above the plane of the page) and all hydrogen atoms except those on C4 and C5 have been omitted for clarity.

The computed energy profiles for the four possible migratory insertion steps involving methyl acrylate are shown in Figure 2, which also defines the atom-labelling scheme employed. Selected computed structures are shown in Figure 3. The profiles start with two pairs of alkene adducts, **D1_2,1_**/**D2_2,1_** (2,1 insertion, see Figure 2i) and **D1_1,2_**/**D2_1,2_** (1,2 insertion, Figure 2ii), which all lie within 0.5 kcal mol^−1^ of one another. Starting with **D2_2,1_** (*G*_DCE_=+4.1 kcal mol^−1^), migratory insertion proceeds by initial rotation of the alkene ligand to achieve a near-planar four-centred transition state, **TS(D2-E2)_2,1_** (*G*_DCE_=+19.7 kcal mol^−1^, C5–C4–Rh–C3=7.2°). As this occurs, the new C4⋅⋅⋅C3 bond begins to form (1.91 Å) with elongation of both the Rh–C4 (2.34 Å, cf. 2.19 Å in **D2_2,1_**) and C4–C5 distances (1.46 Å, cf. 1.42 Å in **D2_2,1_**). **TS(D2-E2)_2,1_** leads to **E2_2,1_** (*G*_DCE_=+5.2 kcal mol^−1^) in which the seven-membered metallacycle adopts a twist-boat conformation, characterised by a N2–C1⋅⋅⋅C4–C5 torsion angle of 33°. Also noticeable is a short contact of 2.41 Å between the rhodium and the methoxy oxygen of the ester substituent. Overall the migratory insertion step proceeds with a barrier of 15.6 kcal mol^−1^ and is slightly endergonic (Δ*G*_DCE_=+1.1 kcal mol^−1^).

In **D1_2,1_** the CO_2_Me group is oriented towards the Cp* ring and this forces the alkene to lie near-parallel to the Rh–N2 bond (C5–C4–Rh–N2=19.7°). A greater degree of alkene rotation is therefore required to access **TS(D1-E1)_2,1_**, but despite this the insertion barrier is the same as that from **D2_2,1_** (15.6 kcal mol^−1^). **TS(D1**-**E1)_2,1_** and **TS(D2**-**E2)_2,1_** therefore only differ in energy by 0.1 kcal mol^−1^.[19] In contrast, **E1_2,1_** is 3.6 kcal mol^−1^ higher in energy than **E2_2,1_**. This may reflect the different conformation of **E1_2,1_**, which exhibits a boat-type structure with {RhCp*} and the C2–C3 bond in the prow and stern positions, respectively, and an N2–C1⋅⋅⋅C4–C5 torsion angle of 5°. Furthermore, **E1_2,1_** does not feature a Rh⋅⋅⋅O short contact such as that seen in **E2_2,1_** (see Figure 3). Full details of all computed structures are supplied in the Supporting Information.

Computed profiles for the alternative 1,2-insertions of methyl acrylate from **D1_1,2_** and **D2_1,2_** are summarized in Figure 2ii. Both pathways proceed through the expected four-centred transition states, but these are now significantly higher in energy (**TS(D1-E1)_1,2_**: +26.5 kcal mol^−1^; **TS(D2-E2)_1,2_**: +26.7 kcal mol^−1^). Thus, 1,2-insertion is clearly disfavoured kinetically over 2,1-insertion, consistent with the observation of only 1,2-disubstituted alkenes experimentally. Therefore only pathways derived from the 2,1-migratory insertion will be considered in the following.

#### Reaction with methyl acrylate: Rearrangement/β-H transfer

**Figure 4:**
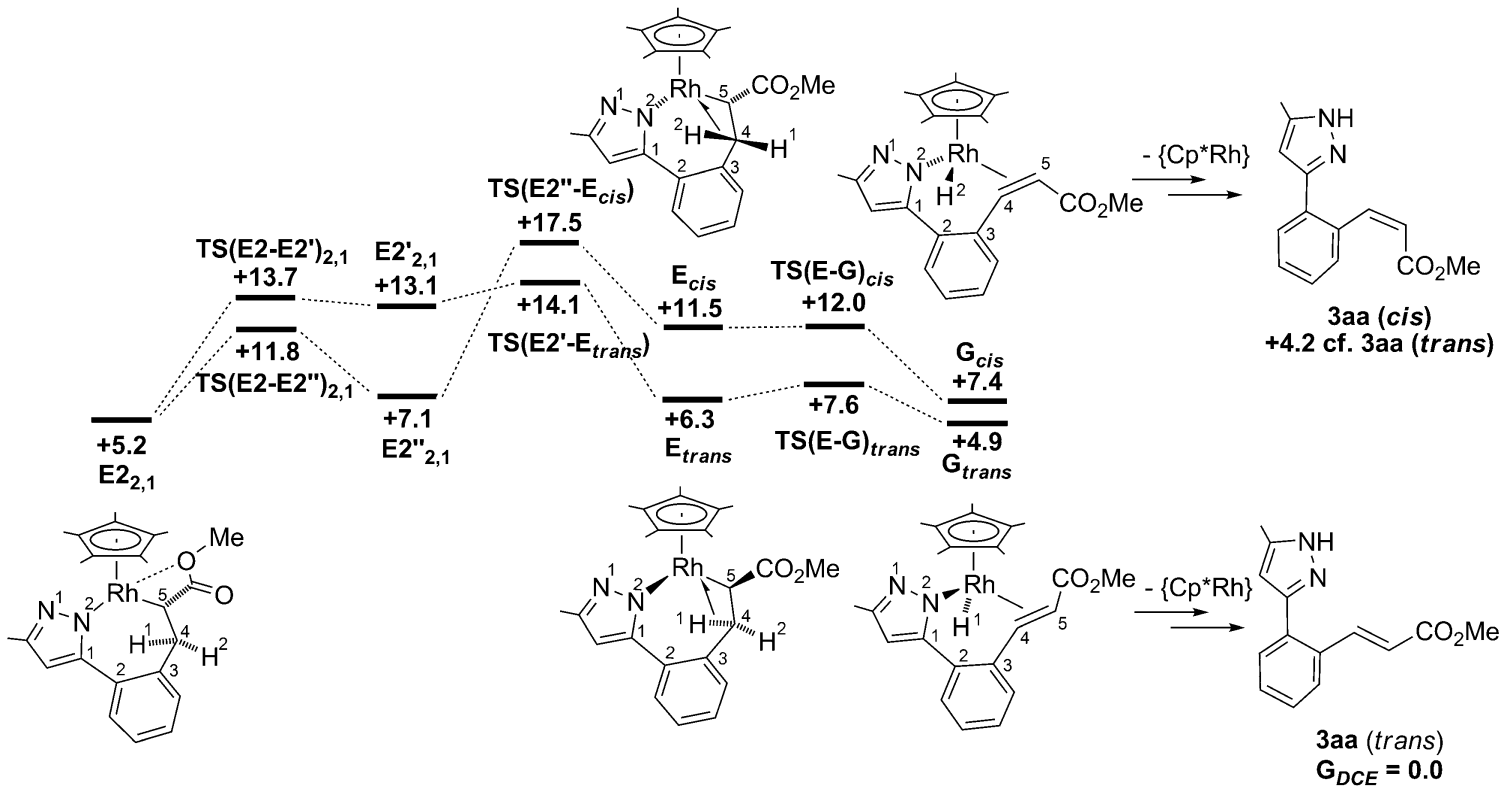
Computed energy profiles (*G*_DCE_, kcal mol^−1^) for β-H transfer from E2_2,1_ formed with methyl acrylate. Energies (*G*_DCE_) are quoted relative to Int(A-B) and free methyl acrylate set to 0.0 kcal mol^−1^, with the exception of the organic products for which *trans* vinyl 3 aa provides the reference energy.

**Figure 5:**
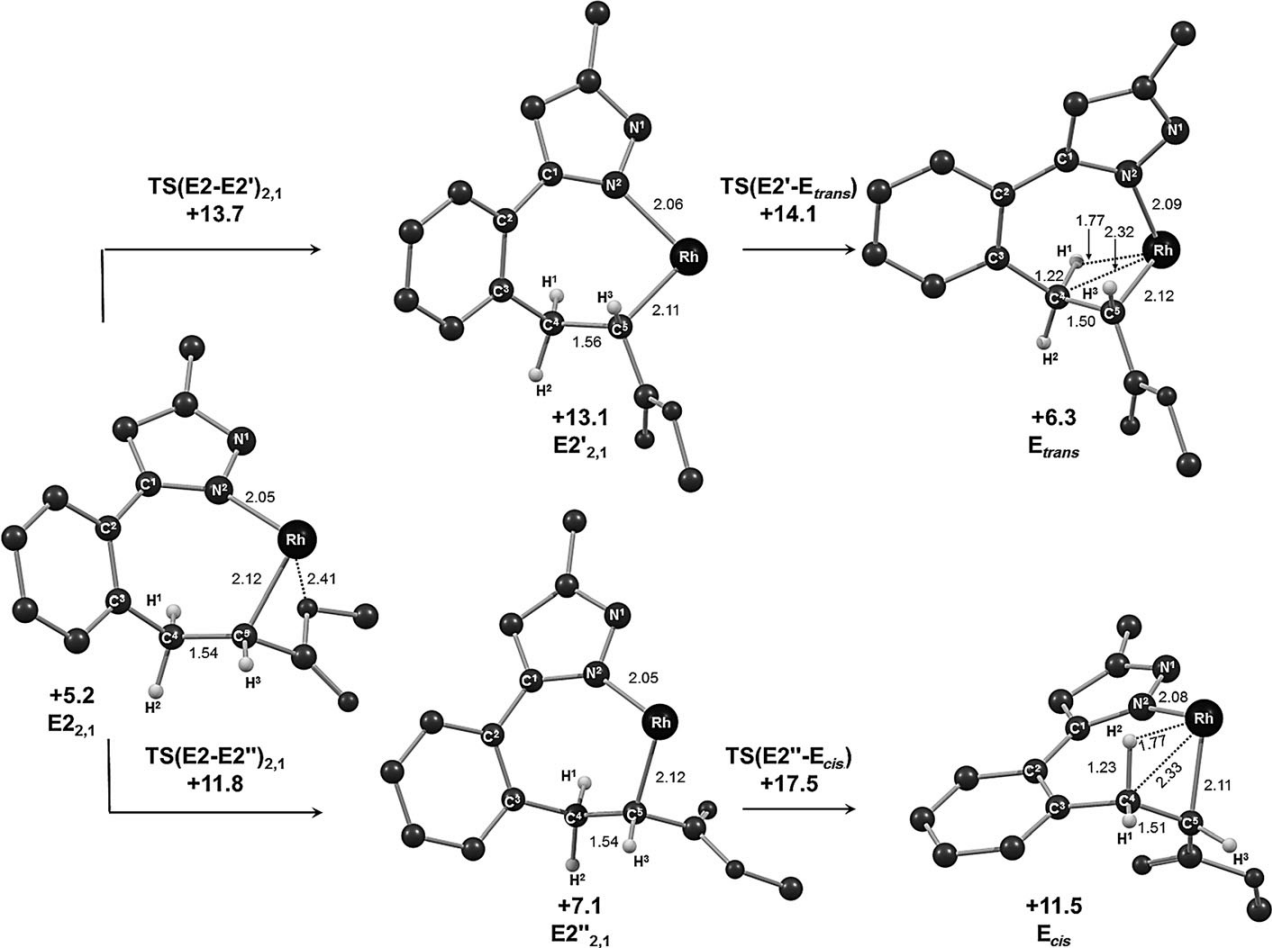
Computed structures for intermediates involved in β-H transfer from E2_2,1_. The Cp* ligand (which lies above the plane of the page) and all hydrogen atoms except those on C4 and C5 have been omitted for clarity.

The stereoselectivity of the vinylation reactions will reflect the ease of β-H transfer in **E1_2,1_** and **E2_2,1_** and so pathways leading to both the *trans* and *cis* isomers of the **3 aa** product were characterised. Along both pathways β-H transfer requires an initial rearrangement to form an agostic intermediate, either **E**_***cis***_ (*G*_DCE_=+11.5 kcal mol^−1^) or **E**_***trans***_ (*G*_DCE_=+6.3 kcal mol^−1^). The seven-membered rhodacycles in **E1_2,1_** and **E2_2,1_** proved to be highly flexible and so allowed pathways linking each of these species to both **E**_***cis***_ and **E**_***trans***_ to be defined. The more accessible routes stem from **E2_2,1_** and both involve two steps (see Figures 4 and 5). To form **E**_***trans***_ initial rotation about the Rh–C5 bond disrupts the Rh⋅⋅⋅OMe interaction in **E2_2,1_** and leads to a new conformer **E2′_2,1_** (*G*_DCE_=+13.1 kcal mol^−1^) in which the rhodacycle exhibits a flattened, twist-boat conformation. The C4–H1 bond can then approach the rhodium centre via **TS(E2′-E**_***trans***_**)** (*G*_DCE_=+14.1 kcal mol^−1^) to give **E**_***trans***_. **E**_***trans***_ exhibits a near-planar arrangement of the Rh, N2 and C1 to C4 centres, whereas the C5 position sits well above this plane to accommodate the approach of the C4–H bond to form the β-agostic interaction.

The first step in forming **E**_***cis***_ from **E2_2,1_** involves the initial rotation of the CO_2_Me substituent to give the conformer **E2′′_2,1_** (*G*_DCE_=+7.1 kcal mol^−1^). This again interrupts the Rh⋅⋅⋅OMe interaction but does not cause any significant change in the conformation of the rhodacycle at this point. In the second step the C3–C4–C5 moiety flips via **TS(E2′′-E**_***cis***_**)** (*G*_DCE_=+17.5 kcal mol^−1^) with rotation of the central {CH_2_} group that permits the C4–H2 bond to approach the rhodium centre in **E**_***cis***_. Both **E**_***cis***_ and **E**_***trans***_ feature strong agostic interactions with elongated C4–H distances of around 1.23 Å and short Rh⋅⋅⋅H contacts of around 1.8 Å. β-H transfer therefore readily occurs with minimal barriers to form **G**_***cis***_ (*G*_DCE_=+7.4 kcal mol^−1^) and **G**_***trans***_ (*G*_DCE_=+4.9 kcal mol^−1^) in which the bound alkene has either a *cis* or *trans* stereochemistry, respectively. **G**_***cis***_ and **G**_***trans***_ (as well as their agostic precursors, **E**_***cis***_ and **E**_***trans***_) are chiral at the rhodium atom and are distinguished by an inversion centre at the rhodium as well as a difference in the nature of the geometric isomer of the alkene (see Figure S3 in the Supporting Information).[20]

Product release from **G**_***trans***_ and **G**_***cis***_ to give the *trans* and *cis* vinyl groups involves a formal N–H reductive elimination and decoordination of the alkene. The former process may be assisted by HOAc or could be coupled to the Cu(OAc)_2_-mediated Rh^I^/Rh^III^ re-oxidation. The higher oxidation state would tend to weaken the alkene binding and so aid substitution by acetate. Given these various factors, and the different order in which these events may occur, we have not considered the product release steps in detail. The computed energies of the organic products show that the *trans* isomer of **3 aa** is favoured thermodynamically over the *cis* isomer by 4.2 kcal mol^−1^.

Considering now the overall pathway from **D1_2,1_** and **D2_2,1_**, both initial migratory insertion processes have very similar barriers (Figure 2), which suggests that a mixture of **E1_2,1_** and **E2_2,1_** would be generated. Both can then undergo rearrangement and β-H transfer to give **G**_***trans***_ and **G**_***cis***_, with these processes being more accessible from **E2_2,1_**. The lower barrier to rearrangement/β-H transfer to form **G**_***trans***_, along with the greater stability of **G**_***trans***_, suggests a preference for the formation of this species and by extension the *trans* alkene product **3 aa**. This is consistent with the experimental results in which only **3 aa** is formed, either as a free species or bound in the dimeric Cu^I^ complex **2 aa**. Interestingly, the high *trans* selectivity (also observed with the other pyrazoles) originates from the rearrangement of **E2_2,1_** to form **E**_***trans***_ rather than in the subsequent β-H transfer step.

#### Reaction with styrene: Migratory insertion

**Figure 6:**
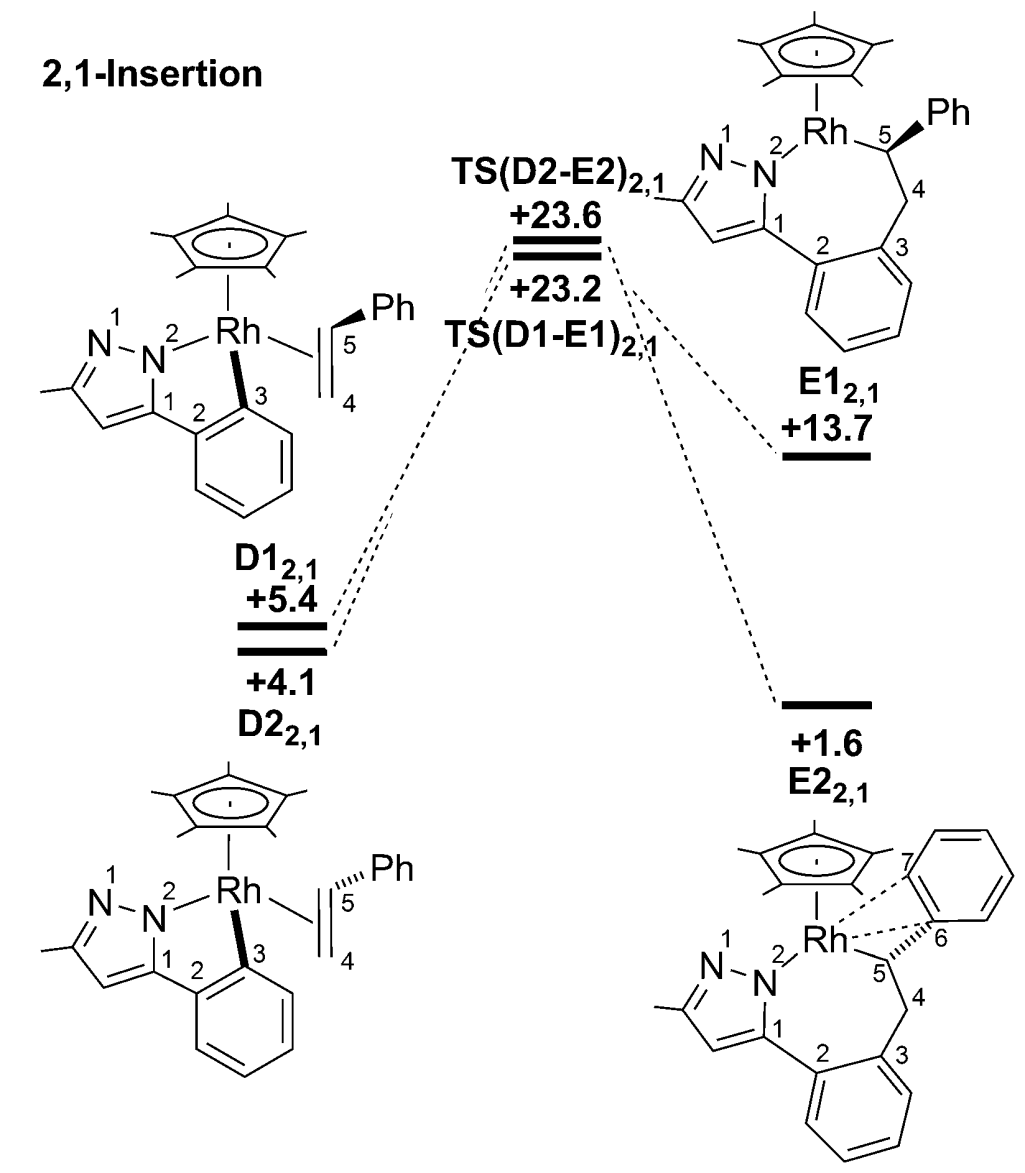
Computed energy profiles (*G*_DCE_, kcal mol^−1^) for the migratory insertion of styrene from adducts D by 2,1-insertion. Energies (*G*_DCE_) are quoted relative to Int(A-B) and free styrene set to 0.0 kcal mol^−1^.

Migratory insertion was computed for all four forms of the styrene adduct **D** (2,1-insertion is shown in Figure 6 with those for 1,2 insertion presented in Figure S4 in the Supporting Information). As with methyl acrylate, a clear preference for 2,1-insertion was computed, with **TS(D2-E2)_2,1_** (*G*_DCE_=+23.6 kcal mol^−1^) and **TS(D1-E1)_2,1_** (*G*_DCE_=+23.2 kcal mol^−1^) lying 3–4 kcal mol^−1^ below the equivalent transition states for 1,2-insertion. The seven-membered rhodacycles are also more stable along the 2,1-insertion pathway, particularly **E2_2,1_** (*G*_DCE_=+1.6 kcal mol^−1^) for which the insertion step from **D2_2,1_** is exergonic. The computed structure of **E2_2,1_** reveals a stabilizing allylic interaction in which the Rh–C5 bond (2.13 Å) is reinforced by interactions with the *ipso* (Rh–C6=2.25 Å) and *ortho* (Rh–C7=2.40 Å) positions of the phenyl substituent. This is similar to the Rh⋅⋅⋅O interaction noted in **E2_2,1_** with methyl acrylate, although the stabilisation is clearly more significant with the phenyl group. No such interaction is possible in **E1_2,1_** (*G*_DCE_=+13.7 kcal mol^−1^) as the phenyl substituent is directed away from the metal centre.

#### Reaction with styrene: Rearrangement/β-H transfer

**Figure 7:**
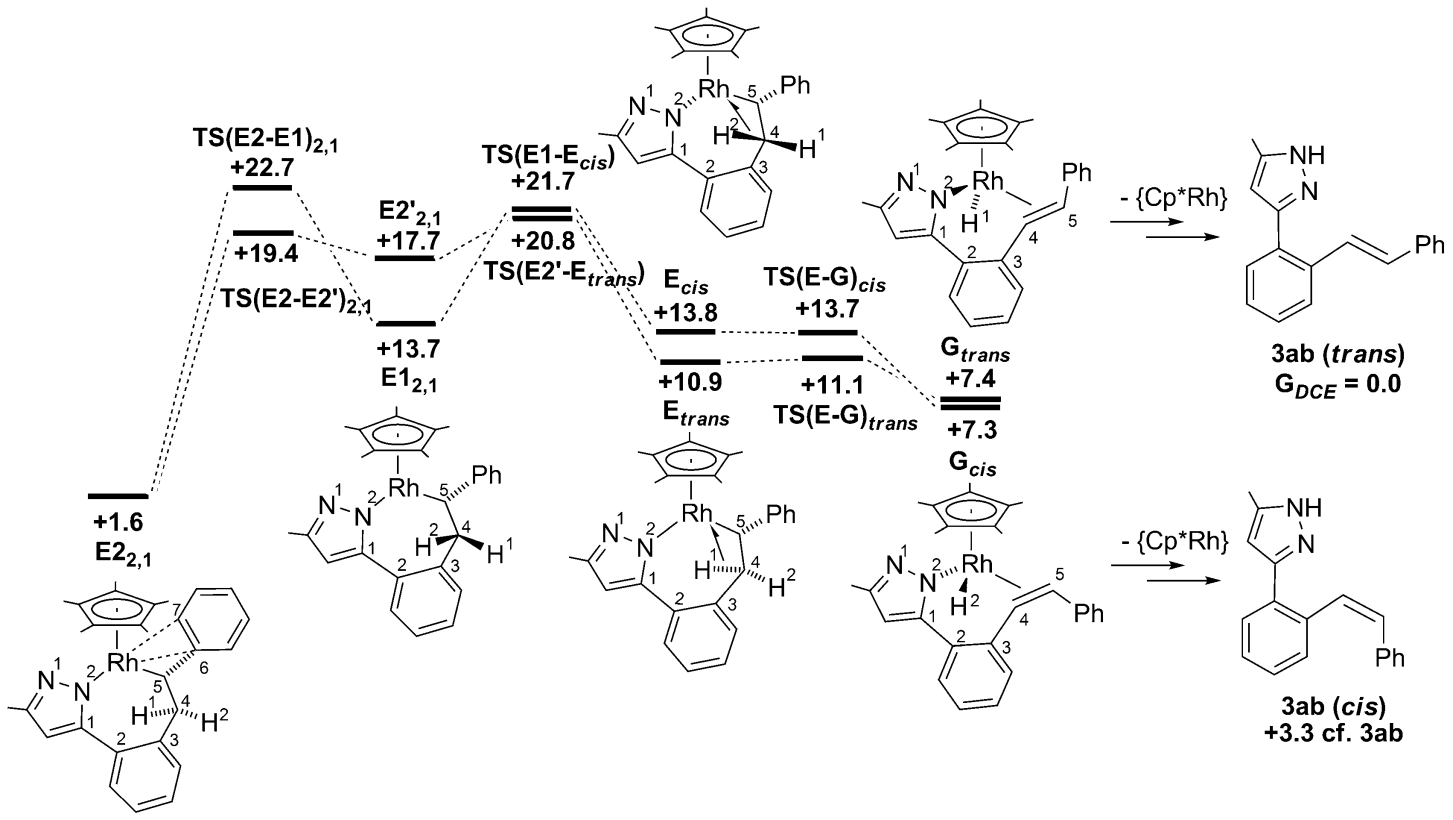
Computed energy profiles (*G*_DCE_, kcal mol^−1^) for β-H transfer from E2_2,1_ formed with styrene. Energies (*G*_DCE_) are quoted relative to Int(A-B) and free styrene set to 0.0 kcal mol^−1^ with the exception of the organic products for which *trans*­ 3 ab provides the reference energy.

The lowest-energy pathways for the formation of **G**_***trans***_ and **G**_***cis***_ are shown in Figure 7. **G**_***trans***_ is accessed directly from **E2_2,1_** in an analogous fashion to that computed with the methyl acrylate system. In this case a significantly larger overall barrier of 19.2 kcal mol^−1^ is computed (cf. 8.9 kcal mol^−1^ with methyl acrylate). This again reflects the greater stability of **E2_2,1_** with the initial rearrangement step to give **E2′_2,1_** having a barrier of 17.8 kcal mol^−1^ before **E**_***trans***_ is accessed via **TS(E2′-E**_***trans***_**)** (+20.8 kcal mol^−1^). The strong agostic interaction in **E**_***trans***_ (C4–H1=1.23 Å; Rh⋅⋅⋅H1=1.76 Å) then allows facile β-H transfer to give **G**_***trans***_ (+7.4 kcal mol^−1^). In contrast to the situation with methyl acrylate, the lowest-energy pathway to **E**_***cis***_ involves the initial rearrangement to **E1_2,1_** via **TS(E2-E1)_2,1_** (*G*_DCE_=+22.7 kcal mol^−1^, see Figure 7). **E**_***cis***_ (*G*_DCE_=+13.8 kcal mol^−1^) is then accessed via **TS(E1-E**_***cis***_**)** (+21.7 kcal mol^−1^),[21] with facile β-H transfer then giving **G**_***cis***_ (+7.3 kcal mol^−1^). **G**_***cis***_ is therefore formed from **E2_2,1_** with an overall barrier of 21.1 kcal mol^−1^.[22]

The extra stability of intermediate **E2_2,1_** in the styrene system has a significant effect on the rearrangement/β-H transfer pathways. This species is the most stable intermediate along the entire pathway from **D** and it makes the rearrangement/β-H transfer process significantly endothermic. The stereoselectivity will therefore be dictated by the barrier to forming **G**_***cis***_ or **G**_***trans***_ from **E2_2,1_** and the calculations indicate this is again determined by the ease of rearrangement of the seven-membered rhodacycles. As for methyl acrylate, this rearrangement is found to be more accessible for the formation of **G**_***trans***_ (Δ*G*^≠^_DCE_=+19.2 kcal mol^−1^) than for **G**_***cis***_ (Δ*G*^≠^_DCE_=+21.1 kcal mol^−1^). Assuming facile product release, **3 ab** would then be formed, consistent with experimental results in which only organic products with a *trans* stereoselectivity were observed.

### Discussion

The rhodium-catalysed vinylation of 3-phenylpyrazoles based on an oxidative C–H coupling strategy has been demonstrated for a range of alkenes, C_2_H_3_Y (Y=CO_2_Me, Ph, C(O)Me). Compared with [RhCl_2_Cp*]_2_, [Rh(MeCN)_3_Cp*][PF_6_]_2_ acts as a particularly efficient catalyst, effecting previously unreported vinylations with both styrene and methyl vinyl ketone. For all three alkenes, insertion occurs regioselectively with the substituted atom being placed next to the metal and leading to linear 1,2-disubstituted vinylation products. The stereoselectivity of the process is very high with the *trans* alkene products being favoured in each case. The final product distribution depends on the ease of aza-Michael cyclisation of the initial vinyl product, which itself reflects the alkene involved. Interestingly, for methyl acrylate the *trans* mono- and divinylation products (**3 aa** and **6 aa**) can undergo an aza-Michael reaction to give fused heterocyclic products (**4 aa** and **5 aa**). Such vinylation Michael cyclisations have been observed previously with pyrazoles[11] and amides,[10] however, here, the vinyl species and the resultant cyclised product have both been isolated for the first time. Moreover, the divinyl product **6 aa** has been shown to cyclise more readily than the monovinyl **3 aa**. Mechanistic experiments indicate that these cyclisations are not metal-catalysed. As the cyclisation of **3 aa** is base-catalysed it can occur when aqueous ammonia is used in the work-up procedure to sequester the copper used in the catalysis. Oxidative C–H coupling with the stronger Michael acceptor methyl vinyl ketone led only to fused heterocyclic products.

DFT calculations have allowed comparison of the possible pathways for the migratory insertion (computed intermediates **D**→**E**) and rearrangement/β-H transfer steps (**E**→**G**) implicated in the formation of monovinylation products from the reaction of 3-phenyl-5-methylpyrazole with both methyl acrylate and styrene. The calculations showed that migratory insertion has a higher barrier than the subsequent rearrangement/β-H transfer. Moreover, the 2,1-insertion mode is kinetically favoured, which reflects the preference for 1,2-disubstituted vinylation products observed experimentally. For methyl acrylate the difference between the alternative 2,1- and 1,2-insertion transition states is around 7 kcal mol^−1^, whereas for styrene this is reduced to 3–4 kcal mol^−1^. The greater discrimination with methyl acrylate is consistent with the results of work on the selectivity of the intermolecular Heck reaction, which also revealed that electronic effects determine the regioselectivity of the reaction.[23] This is supported here by the computed gas-phase enthalpies of the insertion transition states, which also show a preference for 2,1-insertion, that is, this intrinsic electronic preference is not affected by the inclusion of entropy, solvent, dispersion and basis-set effects (see Tables S2 and S3 in the Supporting Information). The calculations highlight a high degree of conformational flexibility in the seven-membered rhodacycles **E1_2,1_** and **E2_2,1_** formed upon the 2,1-insertion of methyl acrylate or styrene. The interconversion of **E1_2,1_** and **E2_2,1_** is therefore possible, whereas the stereoselectivity is ultimately determined by the ease of rhodacycle rearrangement that must occur prior to the β-H transfer step. The flexibility of these rhodacycles means that both the *cis* and *trans* alkene products can be accessed from either insertion intermediate **E1_2,1_** or **E2_2,1_**; however, the calculations correctly indicate a preference for the formation of *trans* vinyl products, as seen experimentally.

The energies of the migratory insertion transition states (methyl acrylate ca. +19.6 kcal mol^−1^; styrene ca. +23.4 kcal mol^−1^) can also be compared with that of the C–H cleavage transition state **TS(B-C)2** (*G*_DCE_=19.5 kcal mol^−1^), which is the highest point of the preceding N–H/C–H activation pathway (see Scheme 6). Thus, for methyl acrylate, migratory insertion will be competitive with C–H activation, and so a similar situation arises to that seen in the reaction of 4-octyne with 3-methyl-5-phenylpyrazole.[6] In contrast, for styrene, the 2,1-insertion transition state is clearly higher in energy and so migratory insertion would correspond to the overall rate-determining step in the cycle.

## Conclusions

We have shown that the Rh^III^-catalysed oxidative coupling of monosubstituted alkenes with 3-phenylpyrazoles gives a range of 1,2-substituted vinylation products depending on the substituents on both the alkene and the 3-phenylpyrazole substrates. The use of aqueous ammonia to remove copper can affect the product distribution by causing base-catalysed cyclisation of the monovinyl product whereas the divinyl product can cyclise without base catalysis. Computational studies have defined the key migratory insertion and rearrangement/β-H transfer steps and correctly model both the regio- and stereoselectivity of the oxidative coupling reactions with methyl acrylate and styrene. The observed *trans* stereoselectivity is linked to the rearrangement of the rhodacycles formed upon migratory insertion.

## Experimental Section

For details of instruments used and the general experimental and computational procedures, see the Supporting Information.

**General procedure for catalysis reactions with rhodium**: The appropriate pyrazole (1 equiv), alkene (1.2 equiv), [Cp*Rh(MeCN)_3_][PF_6_]_2_ (33 mg, 5 mol %), Cu(OAc)_2_**⋅**H_2_O (2.5 equiv) and DCE (10 mL) were added to a Schlenk flask, which was sealed with a screw cap and then transferred to a preheated oil bath and stirred at 83 °C for 16 h. The reaction mixture was cooled to room temperature, diluted with Et_2_O (10 mL) and transferred to a separating funnel to which ammonium hydroxide solution (10 mL, 2 m) was added. The aqueous layer was extracted with Et_2_O (3×10 mL) and the organic layers were combined and dried over MgSO_4_.

**X-ray crystal structure determinations of 2 aa and 4 ba**: A description of the procedure for the collection of crystallographic data for **2 aa** and **4 ab**, including a diagram of the molecular structure and the crystal data of **4 ab**, are given in the Supporting Information.

Crystal data for **2 aa**: C_28_H_26_Cu_2_N_4_O_4_, *M*=609.64, triclinic, *a*=12.007(3), *b*=12.325(4), *c*=20^.^235(6) Å, *α*=96.748(6), *β*=90.416(6), *γ*=115.197(6)°, *V*=2685.2(13 Å^3^, *T*=150(2) K, space group *P*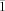, *Z*=2, 21 286 reflections measured, 10 420 independent reflections (*R*_int_=0.1504). The final *R*1 values were 0.0875 (*I*>2*σ*(I)), 0.1767 (all data). The final *wR*(*F*2) values were 0.1719 (*I*>2*σ*(I)), 0.2095 (all data). GOF=0.856.

CCDC 1025819 http://www.ccdc.cam.ac.uk/cgi-bin/catreq.cgi(**2 aa**) and CCDC 1021466 http://www.ccdc.cam.ac.uk/cgi-bin/catreq.cgi(**4 ba**) contain the supplementary crystallographic data for this paper. These data can be obtained free of charge from The Cambridge Crystallographic Data Centre via http://www.ccdc.cam.ac.uk/data_request/cif.

## Supporting Information

As a service to our authors and readers, this journal provides supporting information supplied by the authors. Such materials are peer reviewed and may be re-organized for online delivery, but are not copy-edited or typeset. Technical support issues arising from supporting information (other than missing files) should be addressed to the authors.

miscellaneous_information
