# Supplementary material for: Combined Experimental and Computational Investigations of Rhodium-Catalysed C–H Functionalisation of Pyrazoles with Alkenes
Source: Chemistry. 2014 Dec 17;21(7):3087–96. doi: 10.1002/chem.201405550 (PMC4517174; doi:10.1002/chem.201405550)
Supplement: Supplementary file 1 — miscellaneous_information [file chem0021-3087-sd1.pdf]

CHEMISTRY
A European Journal

Supporting Information

© Copyright Wiley-VCH Verlag GmbH & Co. KGaA, 69451 Weinheim, 2015

Combined Experimental and Computational Investigations of Rhodium-Catalysed C–H Functionalisation of Pyrazoles with Alkenes

Andrés G. Algarra,[a] David L. Davies,*[b] Qudsia Khamker,[b] Stuart A. Macgregor,*[a] Claire L. McMullin,[a] Kuldip Singh,[b] and Barbara Villa-Marcos[b]

chem_201405550_sm_miscellaneous_information.pdf

Supporting Information

Table of Contents

1. Full Experimental Details 2
2. Crystal Structure of **4ba** 7
3. ^{1}H and ^{13}C NMR spectra 9
4. Computational details 43
5. Breakdown of Energy Contributions 43
    a. Octyne 44
    b. Methyl acrylate 45
    c. Styrene 48
6. Functional Testing 51
7. Cartesian Coordinates and calculated energies of all stationary points. 52

1. Full Experimental Details

General experimental procedures

Unless stated otherwise all reactions were carried out under an inert atmosphere of nitrogen work up was carried out in air. ^{1}H, and ^{13}C–{^{1}H} NMR spectra were obtained using a DRX 400 MHz spectrometer. Chemical shifts were recorded in ppm (on δ scale with tetramethylsilane as internal reference), and coupling constants are reported in Hz. FAB mass spectra were obtained on a Kratos concept mass spectrometer using NOBA as matrix. The electrospray (ES) mass spectra were recorded using a micromass Quattra LC mass spectrometer in HPLC grade acetonitrile. Microanalyses were performed by the Elemental Analysis Service (London Metropolitan University). All starting materials were obtained from Aldrich or Alfa Aesar with the exception of [Cp*Rh(MeCN)$_3$][PF$_6$]$_2$,[1] 3-phenyl-5-methyl-1H-pyrazole and 3,5-diphenyl-1H-pyrazole[2] and 3-phenyl-5-(trifluoromethyl)-1H-pyrazole[3] which were prepared according to literature methods.

Reaction of 1a with methyl acrylate (a) (1.2 equiv.)

Following the general procedure, a Schlenk flask was loaded with [Cp*Rh(MeCN)$_3$][PF$_6$]$_2$ (33 mg, 5 mol%), 3-phenyl-5-methyl-1*H*-pyrazole (**1a**, 158 mg, 1.00 mmol), Cu(OAc)$_2$.H$_2$O (500 mg, 2.5 mmol), methyl acrylate (**a**, 103 mg, 1.2 mmol) and DCE (10 ml). The products were purified by column chromatography eluting from 100% dichloromethane to 20% ethyl acetate in hexane to give **2aa** as white solid (123 mg, 41%, 0.41 mmol), **4aa** as yellow oil (40 mg, 17%, 0.17 mmol), **5aa** as a brown oily solid (52 mg, 16%, 0.16 mmol) and **3aa** as yellow oil (34 mg, 14%, 0.14 mmol). On some occasions some **6aa** was also formed. When an alternative work-up was used (celite filtration), the yields were as follows: 54% of **2aa**, <10% **3aa**, <10% **5aa** and 11% **6aa**.

2aa: ^{1}H NMR (400 MHz, CDCl$_3$): δ 2.33 (s, 3H, *Me*), 3.81 (s, 3H, CO$_2$*Me*), 5.06 (d, *J* = 13.7 Hz, 1H, =C*H*(CO$_2$Me)), 6.41 (s, 1H, *Pz-H*), 6.47 (d, *J* = 13.7 Hz, 1H, Ar-*H*C=), 7.19-7.28 (m, 2H, H^3 + H^4), 7.35 (t, *J* = 7.0, 7.4 Hz, 1H, H^2), 7.47 (d, *J* = 7.4 Hz, 1H, H^1), ^{13}C {^{1}H} NMR (100 MHz, CDCl$_3$): δ 13.7 (*Me*), 52.0 (CO$_2$*Me*), 77.8 (=*C*H(CO$_2$Me)), 94.6 (Ar-H*C*=), 102.4 (*C*-H (Pz)), 127.2 (C^1), 127.6 (C^3/C^4), 128.6 (C^3/C^4), 128.9 (C^2), 130.9, 134.4, 150.7, 151.5, 167.8 (*C*O$_2$Me). ESIMS: *m/z* 613 [M+H]$^+$. HRMS (ES): Calcd for $C_{28}H_{27}{}^{65}Cu_2N_4O_4$ [M+H]$^+$ 613.0639, found 613.0588.

3aa: ^{1}H NMR (400 MHz, CDCl$_3$): δ 2.33 (s, 3H, *Me*), 3.77 (s, 3H, CO$_2$*Me*), 6.19 (s, 1H, *Pz-H*), 6.39 (d, *J* = 16.0 Hz, 1H, =C*H*(CO$_2$Me)), 7.36 (td, *J* = 1.6, 7.4 Hz, 1H, H^3), 7.40 (td, *J* = 1.6, 7.4 Hz, 1H, H^2), 7.55 (dd, *J* = 1.6, 7.4 Hz, 1H, H^1), 7.65 (dd, *J* = 1.6, 7.8 Hz, 1H, H^4), 8.13 (d, *J* = 15.7 Hz, 1H, Ar-*H*C=), ^{13}C {^{1}H} NMR (125 MHz, CDCl$_3$): δ 11.5 (*Me*), 51.7 (CO$_2$*Me*), 105.9 (*C*-H (Pz)), 118.9 (=*C*H(CO$_2$Me)), 127.0 (C^4), 128.2 (C^3), 129.7 (C^2), 129.9 (C^1), 132.9 (2C), 133.6, 144.3 (Ar-H*C*=), 167.5 (*C*O$_2$Me). (1 quaternary C not observed). ESIMS: *m/z* 243 [M+H]$^+$, 211 [M-H-OMe]$^+$, 169 [M-CO$_2$Me-Me]$^+$.

4aa: ^{1}H NMR (400 MHz, CDCl$_3$): δ 2.36 (s, 3H, *Me*), 2.77 (dd, *J* = 7.8, 16.0 Hz, 1H, C*H$_2$*), 3.30 (dd, *J* = 3.1, 16.4 Hz, 1H, C*H$_2$*), 3.77 (s, 3H, CO$_2$*Me*), 5.49 (br dd, *J* = 3.1 Hz, 1H, CH$_2$C*H*), 6.16 (broad s, 1H, *Pz-H*), 7.30 (t, *J* = 7.4 Hz, 1H, H^2/H^3), 7.39 (t, *J* = 7.4 Hz, 1H, H^2/H^3), 7.45 (d, *J* = 7.4 Hz, 1H, H^1/H^4), 7.53 (d, *J* = 7.4 Hz, 1H, H^1/H^4), ^{13}C {^{1}H} NMR (125 MHz, CDCl$_3$): δ 14.5 (*Me*), 38.7 (*C*H$_2$),

52.1 (CO_2*Me*), 58.8 (CH_2*C*H), 120.4 (*C*[1]/*C*[4]), 123.5 (*C*[1]/*C*[4]), 127.3 (*C*[2]/*C*[3]), 128.7 (*C*[2]/*C*[3]), 144.2, 170.7 (*C*O_2Me) (2 quaternary C not observed and pyrazole CH not observed in normal ^{13}C spectrum). ESIMS: *m/z* 243 $[M+H]^+$. HRMS (ES): Calcd for $C_{14}H_{15}N_2O_2$ $[M+H]^+$ 243.1134, found 243.1140.

5aa: ^{1}H NMR (400 MHz, $CDCl_3$): δ 2.38 (s, 3H, *Me*), 2.70 (dd, *J* = 8.6, 16.4 Hz, 1H, C*H_2*), 3.22 (dd, *J* = 4.7, 16.4 Hz, 1H, C*H_2*), 3.76 (s, 3H, CO_2*Me*), 3.85 (s, 3H, CO_2*Me*), 5.42 (dd, *J* = 4.7, 8.2 Hz, 1H, CH_2C*H*), 6.36 (s, 1H, *Pz-H*), 6.52 (d, *J* = 16.0 Hz, 1H, =C*H*(CO_2Me)), 7.30 (t, *J* = 7.4, 7.8 Hz, 1H, H^2), 7.46 (d, *J* = 7.4 Hz, 1H, H^3), 7.60 (d, *J* = 7.8 Hz, 1H, H^1), 8.04 (d, *J* = 16.0 Hz, 1H, Ar-*H*C=), ^{13}C {^{1}H} NMR (100 MHz, $CDCl_3$): δ 11.4 (*Me*), 38.6 (*C*H_2), 51.9 (CO_2*Me*), 52.1 (CO_2*Me*), 58.7 (CH_2*C*H), 99.5 (*C*-H (Pz)), 119.9 (=*C*H(CO_2Me)), 124.8 (C^3), 125.7 (C^1), 127.7 (C^2), 130.9, 140.4 (Ar-H*C*=), 144.9, 167.0 (*C*O_2Me), 170.5 (*C*O_2Me) (3 quaternary C not observed). ESIMS: *m/z* 327 $[M+H]^+$. HRMS (ES): Calcd for $C_{18}H_{19}N_2O_4$ $[M+H]^+$ 327.1345, found 327.1338. In some ^{1}H NMR spectra, signals for the CH_2CH protons are broad.

6aa: ^{1}H NMR (400 MHz, $CDCl_3$): δ 2.25 (s, 3H, *Me*), 3.65 (s, 6H, CO_2*Me*), 5.98 (s, 1H, *Pz-H*), 6.26 (d, *J* = 15.7 Hz, 2H, 2 x =C*H*(CO_2Me)), 7.33 (t, *J* = 7.8 Hz, 1H, H^2), 7.55-7.61 (m, 4H, 2 x Ar-*H*C= + H^1 + H^3), ^{13}C {^{1}H} NMR (100 MHz, $CDCl_3$): δ 13.3 (*Me*), 50.6 (CO_2*Me*), 106.9 (*C*-H (Pz)), 118.8 (=*C*H(CO_2Me)), 124.7 (C^2), 126.7 (Ar), 126.8 (Ar), 139.4 (Ar-*C*H=), 169.5 (*C*O_2Me). (**6aa** converts into **5aa** in solution, difficult to do full assignment). FAB MS: *m/z* 327 $[M+H]^+$, 295 $[M-OMe]^+$, 267 $[M-CO_2Me]^+$, 207 $[M-2(CO_2Me)]^+$, 154 $[M-2(CH=CH(CO_2Me))]^+$. HRMS (ES): Calcd for $C_{18}H_{19}N_2O_4$ $[M+H]^+$ 327.1345, found 327.1338.

Reaction of 1a with methyl acrylate (a) (2.2 equiv.)

Following the general procedure, a Schlenk flask was loaded with $[Cp^*Rh(MeCN)_3][PF_6]_2$ (33 mg, 5 mol%), 3-phenyl-5-methyl-1*H*-pyrazole (**1a**, 158 mg, 1.00 mmol), $Cu(OAc)_2.H_2O$ (500 mg, 2.5 mmol), methyl acrylate (**a**, 189 mg, 2.2 mmol) and DCE (10 ml). The product was purified by column chromatography eluting with 5 % ethyl acetate in hexane to give **5aa** as a brown solid (280 mg, 86%, 0.86 mmol).

Reaction of 1b with methyl acrylate (a)

Following the general procedure, a Schlenk flask was loaded with $[Cp^*Rh(MeCN)_3][PF_6]_2$ (33 mg, 5 mol%), 3,5-diphenyl-1*H*-pyrazole (**1b**, 220 mg, 1.00 mmol), $Cu(OAc)_2.H_2O$ (500 mg, 2.5 mmol), methyl acrylate (**a**, 103 mg, 1.2 mmol) and DCE (10 ml). The products were purified by column chromatography eluting with 30% ethyl acetate in petroleum ether (40-60 °C) to give **3ba** as a pale yellow oil (107 mg, 35%, 0.35 mmol), **6ba** as a yellow oil (impure) and **4ba** as a yellow solid (impure). Compounds **6ba** and **4ba** were purified further by tlc.

3ba: ^{1}H NMR (400 MHz, $CDCl_3$): δ 3.75 (s, 3H, CO_2*Me*), 6.43 (d, *J* = 16.0 Hz, 1H, =C*H*(CO_2Me)), 6.70 (s, 1H, *Pz-H*), 7.33-7.37 (m, 3H, H^2 + H^3 + H^7), 7.42 (td, *J* = 2.0, 7.0 Hz, 2H, H^6), 7.60 (dd, *J* = 1.2, 7.0 Hz, 1H, H^1), 7.67 (dd, *J* = 2.0, 7.4 Hz, 1H, H^4), 7.70 (d, *J* = 7.0 Hz, 2H, H^5), 8.11 (d, *J* = 15.7 Hz, 1H, Ar-*H*C=), ^{13}C {^{1}H} NMR (100 MHz, $CDCl_3$): δ 50.5 (CO_2*Me*), 102.8 (*C*-H (Pz)), 118.4 (=*C*H(CO_2Me)), 124.5 (C^5), 126.0 (C^4), 127.1, 127.2, 127.4, 127.7 (Ar), 127.7 (C^6), 128.5 (Ar), 128.8 (Ar), 131.1, 131.9, 142.5 (Ar-H*C*=), 166.1 (*C*O_2Me) (3 C not observed, possibly quaternary). ESIMS:

m/z 305 $[M+H]^+$, 274 $[M-OMe]^+$. HRMS (ES): Calcd for $C_{19}H_{17}N_2O_2$ $[M+H]^+$ 305.1290, found 305.1283.

4ba: This product was obtained by preparative TLC eluting with 30 % ethyl acetate in petroleum ether (40-60 °C) to give **4ba** as a pale yellow solid (80 mg, 4%, 0.04 mmol). ^{1}H NMR (400 MHz, $CDCl_3$): δ 2.76 (dd, *J* = 8.6, 16.4 Hz, 1H, CH_2), 3.29 (dd, *J* = 5.1, 16.4 Hz, 1H, CH_2), 3.71 (s, 3H, *Me*), 5.55 (dd, *J* = 5.1, 8.6 Hz, 1H, CH_2C*H*), 6.60 (s, 1H, *Pz-H*), 7.22-7.27 (m, 2H, H^3 + H^7), 7.32-7.37 (m, 3H, H^2 + H^6), 7.41 (d, *J* = 7.4 Hz, 1H, H^4), 7.52 (d, *J* = 7.4 Hz, 1H, H^1), 7.77 (dd, *J* = 1.6, 7.4 Hz, 2H, H^5), ^{13}C {^{1}H} NMR (100 MHz, $CDCl_3$): δ 37.4 (*C*H_2), 50.7 (*Me*), 57.8 (CH_2*C*H), 92.3 (*C*-H (Pz)), 119.1 (C^1), 122.1 (C^4), 124.2 (C^5), 126.2 (C^3/C^7), 126.3 (C^3/C^7), 127.2 (C^6), 127.3 (C^2), 128.9, 132.6, 142.7, 144.8, 154.9, 169.9 (*C*=O). HRMS (ES): Calcd for $C_{19}H_{17}N_2O_2$ $[M+H]^+$ 305.1290, found 305.1280. The product was recrystallised from dichloromethane/hexane to give **4ba** as clear needles.

6ba: This product was purified by preparative TLC eluting with 30 % ethyl acetate in petroleum ether (40-60 °C) to give **6ba** as yellow oil (80 mg, 15%, 0.15 mmol). ^{1}H NMR (400 MHz, $CDCl_3$): δ 3.71 (s, 6H, *Me*), 6.37 (d, *J* = 16.0 Hz, 2H, =C*H*(CO_2Me)), 6.53 (br s, 1H, *Pz-H*), 7.32-7.43 (m, 4H, H^2 + H^4 + H^5), 7.54 (d, *J* = 7.0 Hz, 2H, H^3), 7.62 (dd, *J* = 1.2, 7.4 Hz, 2H, H^1), 8.06 (d, *J* = 16.0 Hz, 2H, Ar-*H*C=), 10.27 (br s, 1H, N*H*), ^{13}C {^{1}H} NMR (100 MHz, $CDCl_3$): δ 50.6 (*Me*), 118.4, 118.6 (=*C*H(CO_2Me)), 124.6 (C^1), 126.2, 127.2, 127.5, 127.6 (C^4), 127.8, 128.7, 128.9 (C^3), 132.0, 142.6, 166.2 (*C*O_2Me) (Pyrazole CH not observed in normal ^{13}C spectrum but shows a correlation in the HMQC spectrum at δ 107.6). HRMS (ES): Calcd for $C_{23}H_{21}N_2O_4$ $[M+H]^+$ 389.1501, found 389.1494.

Reaction of 1c with methyl acrylate (a)

Following the general procedure, a Schlenk flask was loaded with [Cp*Rh(MeCN)$_3$][PF_6]$_2$ (16 mg, 5 mol%), 3-phenyl-5-(trifluoromethyl)-1H-pyrazole (**1c**, 212 mg, 1.00 mmol), Cu(OAc)$_2$.H_2O (500 mg, 2.5 mmol), methyl acrylate (**a**, 103 mg, 1.2 mmol) and DCE (10 ml). The crude ^{1}H NMR spectrum showed the presence of two main products in an 8:1 ratio. The products were purified by column chromatography eluting from 100% dichloromethane to 10% ethyl acetate in dichloromethane to give **3ca** as a yellow oil (215 mg, 73%, 0.73 mmol) and **2ca** as a white solid (34 mg, 9%, 0.09 mmol).

2ca: ^{1}H NMR (400 MHz, $CDCl_3$): δ 3.82 (s, 3H, CO_2*Me*), 5.26 (d, *J* = 14.1 Hz, 1H, =C*H*(CO_2Me)), 6.71 (d, *J* = 14.1 Hz, 1H, Ar-*H*C=), 6.92 (s, 1H, *Pz-H*), 7.31-7.35 (m, 2H, H^3 + H^4), 7.42 (td, *J* = 2.0, 7.8, 8.2 Hz, 1H, H^2), 7.51 (br d, *J* = 7.4 Hz, 1H, H^1), ^{13}C {^{1}H} NMR (125 MHz, CD_2Cl_2): δ 52.3 (CO_2*Me*), 82.8 (=*C*H(CO_2Me)), 99.5 (Ar-H*C*=), 103.1 (*C*-H (Pz)), 121.7 (q, *J* = 263.6 Hz, *C*F_3), 128.2 (C^1), 128.4 (C^3/C^4), 129.1 (C^3/C^4), 129.8 (C^2), 131.3, 133.2, 144.3 (q, *J* = 35.8 Hz, *C*-CF_3), 151.4, 168.1 (*C*=O), ^{19}F {^{1}H} NMR (376 MHz, $CDCl_3$): δ -59.8 (C*F_3*).

3ca: ^{1}H NMR (400 MHz, $CDCl_3$): δ 3.75 (s, 3H, CO_2*Me*), 6.42 (d, *J* = 16.0 Hz, 1H, =C*H*(CO_2Me)), 6.64 (s, 1H, *Pz-H*), 7.47-7.49 (m, 3H, H^1 + H^2 + H^3), 7.69 (m, 1H, H^4), 7.81 (d, *J* = 16.0 Hz, 1H, Ar-*H*C=), 11.52 (br s, 1H, N*H*), ^{13}C {^{1}H} NMR (125 MHz, $CDCl_3$): δ 52.0 (CO_2*Me*), 105.1 (*C*-H (Pz)), 121.0 (=*C*H(CO_2Me)), 121.2 (q, *J* = 270.6 Hz, *C*F_3), 127.5 (C^4), 128.8, 129.8 (C^2 + C^3), 130.3 (C^1), 133.2, 142.0 (Ar-H*C*=), 167.1 (*C*=O), (2 quaternary C not observed); ^{19}F {^{1}H} NMR (376 MHz, $CDCl_3$): δ -62.0 (C*F_3*). ESIMS: *m/z* 297 $[M+H]^+$. HRMS (ES): Calcd for $C_{14}H_{12}N_2F_3O_2$ $[M+H]^+$ 297.0851, found 297.0858.

Reaction of 1a with styrene (b)

Following the general procedure, a Schlenk flask was loaded with $[Cp^*Rh(MeCN)_3][PF_6]_2$ (33 mg, 5 mol%), 3-phenyl-5-methyl-1*H*-pyrazole (**1a**, 158 mg, 1.00 mmol), $Cu(OAc)_2.H_2O$ (500 mg, 2.5 mmol), styrene (**b**, 125 mg, 1.2 mmol) and DCE (10 ml). The products were purified by column chromatography eluting with 50% ethyl acetate in hexane to give **3ab** as yellow oil (157 mg, 60%, 0.60 mmol).

3ab: ^{1}H NMR (400 MHz, $CDCl_3$): δ 2.35 (s, 3H, *Me*), 6.23 (br s, 1H, *Pz-H*), 7.06 (d, 1H, *J* = 16.0 Hz, C=C*H*), 7.32-7.40 (m, 6H, *Ar-H*), 7.44-7.51 (m, 3H, 2 x *Ar-H* + *H*C=CH), 7.70 (dd, *J* = 7.4, 8.6 Hz, 1H, *Ar-H*), 10.05 (br s, 1H, N*H*), ^{13}C {^{1}H} NMR (125 MHz, $CDCl_3$): δ 11.9 (*Me* (Pz)), 100.6 (*C*-H (Pz)), 126.1, 126.7 (Ar), 127.6, 127.7 (Ar), 128.2, 128.3, 128.7 (Ar), 129.1, 129.5, 130.0 (C=*C*H), 131.7, 135.7, 136.0, 136.8, 137.6. ESIMS: *m/z* 261 $[M+H]^+$. HRMS (ES): Calcd for $C_{18}H_{17}N_2$ $[M+H]^+$ 261.1392, found 261.1391.

Reaction of 1b with styrene (b)

Following the general procedure, a Schlenk flask was loaded with $[Cp^*Rh(MeCN)_3][PF_6]_2$ (33 mg, 5 mol%), 3,5-diphenyl-1*H*-pyrazole (**1b**, 220 mg, 1.00 mmol), $Cu(OAc)_2.H_2O$ (500 mg, 2.5 mmol), styrene (**b**, 125 mg, 1.2 mmol) and DCE (10 ml). The product was purified by column chromatography eluting with 100% dichloromethane and 30% ethyl acetate in hexane to give **3bb** as a pale yellow solid (287 mg, 89%, 0.89 mmol).

3bb: ^{1}H NMR (400 MHz, $CDCl_3$): δ 6.68 (br s, 1H, *Pz-H*), 7.03 (d, *J* = 16.0 Hz, 1H, C=C*H*), 7.18 (m, 1H, *Ar-H*), 7.24-7.31 (m, 5H, H^2 + H^3 + C=C*H*, *Ar-H*), 7.34-7.40 (m, 5H, *Ar-H*), 7.45 (br d, *J* = 7.4 Hz, 1H, H^1), 7.66 (d, *J* = 7.4 Hz, 1H, H^4), 7.71 (br d, *J* = 7.8 Hz, 2H, H^5), 10.40 (br s, 1H, N*H*), ^{13}C {^{1}H} NMR (125 MHz, $CDCl_3$): δ 103.7 (*C*-H (Pz)), 125.7 (C^5), 126.6 (Ar), 126.7 (C^4), 126.9, 127.8 (Ar), 127.9 (Ar), 128.1, 128.2 (Ar), 128.5, 128.8, 128.9 (Ar), 129.0 (Ar), 129.4 (C^1), 131.0 (C=*C*H), 136.1, 137.2 (1 C not observed, possibly quaternary). ESIMS: *m/z* 323 $[M+H]^+$. HRMS (ES): Calcd for $C_{23}H_{19}N_2$ $[M+H]^+$ 323.1548, found 323.1545.

Reaction of 1a with methyl vinyl ketone (c) (1.2 equiv.)

Following the general procedure, a Schlenk flask was loaded with $[Cp^*Rh(MeCN)_3][PF_6]_2$ (33 mg, 5 mol%), 3-phenyl-5-methyl-1*H*-pyrazole (**1a**, 158 mg, 1.00 mmol), $Cu(OAc)_2.H_2O$ (500 mg, 2.5 mmol), methyl vinyl ketone (**c**, 84 mg, 1.2 mmol) and DCE (10 ml). The crude ^{1}H NMR spectrum showed the presence of two products in a 4:1 ratio. The products were purified by column chromatography eluting with 100% dichloromethane to give **4ac** as a yellow oil (158 mg, 70%, 0.70 mmol) and **7ac** as a yellow oil (18 mg, 8%, 0.08 mmol).

4ac: ^{1}H NMR (400 MHz, $CDCl_3$): δ 2.22 (s, 3H, CO*Me*), 2.36 (s, 3H, *Me*), 2.86 (dd, *J* = 8.6, 18.0 Hz, 1H, CH_2), 3.47 (dd, *J* = 3.9, 18.0 Hz, 1H, CH_2), 5.55 (dd, *J* = 3.9, 8.6 Hz, 1H, CH_2C*H*), 6.14 (s, 1H, *Pz-H*), 7.27 (t, *J* = 7.4 Hz, 1H, H^3), 7.37 (t, *J* = 7.4 Hz, 1H, H^2), 7.44 (d, *J* = 7.4 Hz, 1H, H^4), 7.52 (d, *J* = 7.4 Hz, 1H, H^1), ^{13}C {^{1}H} NMR (100 MHz, $CDCl_3$): δ 14.4 (*Me*), 30.4 (CO*Me*), 47.9 (*C*H_2), 58.2 (CH_2*C*H), 96.2 (*C*-H (Pz)), 120.3 (C^1), 123.9, 127.3 (C^4), 128.5 (C^3), 129.5, 130.4 (C^2), 144.9, 153.4,

205.3 (C=O). ESIMS: *m/z* 227 $[M+H]^+$. HRMS (ES): Calcd for $C_{14}H_{15}N_2O$ $[M+H]^+$ 227.1184, found 227.1185.

7ac: ^{1}H NMR (400 MHz, $CDCl_3$): δ 2.13 (s, 3H, CO(*Me*)), 2.30 (s, 3H, *Me*), 3.06 (t, *J* = 6.7 Hz, 2H, C*H$_2$*(C=O)), 4.24 (t, *J* = 6.7 Hz, 2H, NC*H$_2$*), 6.25 (s, 1H, *Pz-H*), 7.24 (tt, *J* = 1.2, 7.4 Hz, 1H, H^3), 7.34 (td, *J* = 1.2, 7.4 Hz, 2H, H^2), 7.73 (dd, *J* = 1.2, 7.4 Hz, 2H, H^1), ^{13}C {^{1}H} NMR (100 MHz, $CDCl_3$): δ 11.1 (*Me*), 30.3 (CO*Me*), 43.0 (CH_2), 43.1 (CH_2), 102.5 (*C*-H (Pz)), 125.4 (C^1), 127.4 (C^3), 128.5 (C^2), 133.8, 139.8, 150.4, 206.3 (C=O). ESIMS: *m/z* 229 $[M+H]^+$. HRMS (ES): Calcd for $C_{14}H_{17}N_2O$ $[M+H]^+$ 229.1341, found 229.1337.

Reaction of 1a with methyl vinyl ketone (c) (2.2 equiv.)

Following the general procedure, a Schlenk flask was loaded with $[Cp^*Rh(MeCN)_3][PF_6]_2$ (33 mg, 5 mol%), 3-phenyl-5-methyl-1*H*-pyrazole (**1a**, 158 mg, 1.00 mmol), $Cu(OAc)_2.H_2O$ (500 mg, 2.5 mmol), methyl vinyl ketone (**c**, 154 mg, 2.2 mmol) and DCE (10 ml). The crude ^{1}H NMR spectrum showed the presence of two products in a 1.4:1 ratio. The products were purified by column chromatography eluting with 50 % ethyl acetate in petroleum ether (40-60 °C) to give **8ac** as a yellow oil (137 mg, 46%, 0.46 mmol) and **4ac** as a yellow oil (90 mg, 40%, 0.40 mmol) (see above for characterisation).

8ac: ^{1}H NMR (400 MHz, $CDCl_3$): δ 2.16 (s, 3H, CO*Me*), 2.21 (s, 3H, CO*Me*), 2.36 (s, 3H, *Me(Pz)*), 2.81 (t, *J* = 7.4, 7.8 Hz, 2H, C*H$_2$*), 2.87 (dd, *J* = 8.6, 17.6 Hz, 1H, C*H$_2$*CH), 3.09 (t, *J* = 7.0, 8.2 Hz, 2H, C*H$_2$*), 3.45 (dd, *J* = 4.3, 17.6 Hz, 1H, C*H$_2$*CH), 5.55 (dd, *J* = 4.3, 8.6 Hz, 1H, CH_2C*H*), 6.11 (s, 1H, *Pz-H*), 7.17-7.22 (m, 2H, H^1 + H^2), 7.29 (dd, *J* = 2.0, 7.0 Hz, 1H, H^3), ^{13}C {^{1}H} NMR (125 MHz, $CDCl_3$): δ 14.4 (*Me(Pz)*), 27.2 (CH_2), 30.1 (CO*Me*), 30.5 (CO*Me*), 43.5 (CH_2), 48.0 (*C*H$_2$CH), 58.2 (CH_2*C*H), 97.8 (*C*-H (Pz)), 121.7 (C^3), 127.7 (C^1/C^2), 128.7 (C^1/C^2), 129.2, 134.1, 144.8, 145.1, 153.5, 205.3 (C=O), 207.4 (C=O). ESIMS: *m/z* 297 $[M+H]^+$. HRMS (ES): Calcd for $C_{18}H_{21}N_2O_2$ $[M+H]^+$ 297.1603, found 297.1597.

Reaction of 1a with methyl vinyl ketone (c)

Following the general procedure, a Schlenk flask was loaded with 3-phenyl-5-methyl-1*H*-pyrazole (**1a**, 158 mg, 1.00 mmol), methyl vinyl ketone (**c**, 84 mg, 1.2 mmol) and DCE (10 ml). The product was purified by column chromatography eluting from 10% ethyl acetate in hexane to give **7ac** as a white solid (214 mg, 94%, 0.94 mmol).

Reaction of 1b with methyl vinyl ketone (c)

Following the general procedure, a Schlenk flask was loaded with $[Cp^*Rh(MeCN)_3][PF_6]_2$ (33 mg, 5 mol%), 3,5-diphenyl-1*H*-pyrazole (**1b**, 220 mg, 1.00 mmol), $Cu(OAc)_2.H_2O$ (500 mg, 2.5 mmol), methyl vinyl ketone (**c**, 84 mg, 1.2 mmol) and DCE (10 ml). The product was purified by column chromatography eluting from 1% ethyl acetate in dichloromethane to give **4bc** as a white solid (266 mg, 92%, 0.92 mmol).

4bc: ^{1}H NMR (400 MHz, $CDCl_3$): δ 2.16 (s, 3H, *Me*), 2.83 (dd, *J* = 9.0, 18.0 Hz, 1H, C*H$_2$*), 3.46 (dd, *J* = 3.9. 18.0 Hz, 1H, C*H$_2$*), 5.59 (dd, *J* = 3.9, 9.0 Hz, 1H, CH_2C*H*), 6.58 (s, 1H, *Pz-H*), 7.21-7.25 (m, 2H, H^3 + H^7), 7.31-7.35 (m, 3H, H^2 + H^6), 7.40 (dd, *J* = 0.8, 7.8 Hz, 1H, H^4), 7.50 (d, *J* = 7.4 Hz, 1H, H^1),

7.76 (dd, J = 1.6, 8.6 Hz, 2H, H^5), ^{13}C {^{1}H} NMR (100 MHz, $CDCl_3$): δ 29.5 (*Me*), 47.0 (CH_2), 57.7 (CH_2CH), 92.7 (*C*-H (Pz)), 119.4 (C^1), 123.0 (C^4), 124.5 (C^5), 126.6 (C^3/C^7), 126.7 (C^3/C^7), 127.6 (C^2), 127.6 (C^6), 129.1, 133.0, 143.7, 145.2, 155.2, 204.3 (*C*=O). HRMS (ES): Calcd for $C_{19}H_{17}N_2O$ $[M+H]^+$ 289.1341, found 289.1336.

Conversion of 2ba to 4ba

An NMR tube was loaded with **2ba** (30 mg, 0.10 mmol) dissolved in $CDCl_3$. To this was added ^{t}BuOK (11 mg, 0.10 mmol) and the tube was shaken. Monitoring by ^{1}H NMR spectroscopy showed complete conversion to **4ba** almost immediately.

2. Crystal Structure of 4ba

Data were collected on a Bruker Apex 2000 CCD diffractometer using graphite monochromated Mo-K_α radiation, λ = 0.7107 Å. The data were corrected for Lorentz and polarisation effects and empirical absorption corrections were applied. The structure was solved by direct methods and with structure refinement on F^2 employed SHELXTL version 6.10.[4] Hydrogen atoms were included in calculated positions (C—H = 0.95 – 1.00 Å) riding on the bonded atom with isotropic displacement parameters set to 1.5U_{eq} (C) for methyl hydrogen atoms and 1.2U_{eq} (C) for all other H atoms. All non-hydrogen atoms were refined with anisotropic displacement parameters without positional restraints. Figures were drawn using the program ORTEP.[5] Crystal data for.**4ba** $C_{19}H_{16}N_2O_2$, M = 304.34, Triclinic, a = 9.9460(18) Å, b = 13.007(2) Å, c = 13.248(2) Å, α = 114.451(4)°, β = 98.994(4)°, γ = 95.003(4)°, V = 1518.9(5) Å^3, T = 150(2) K, space group P-1, Z = 4, 8088 reflections measured, 5068 independent reflections (Rint = 0.0595). The final R1 values were 0.0689 (I > 2σ(I)) 0.1175 (all data). The final wR(F2) values were 0.1461 (I > 2σ(I)), 0.1707 (all data). GOF = 0.932.

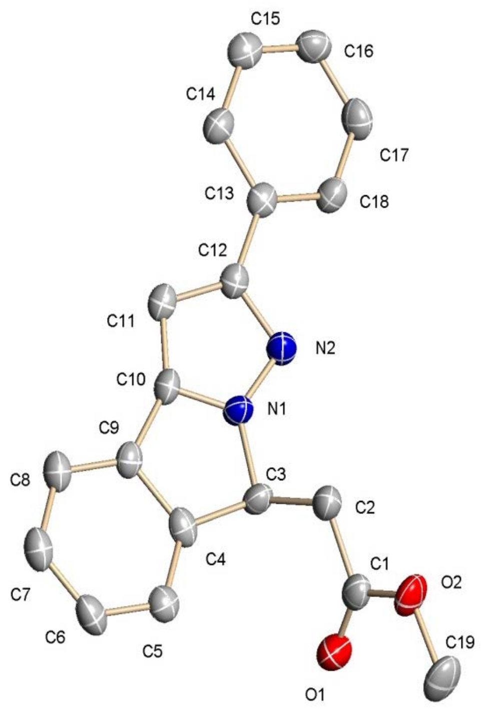

Figure S1. Molecular structure of **4ba** showing one of the two unique molecules present in the unit cell. Hydrogen atoms have been omitted for clarity.

References

[1] C. White, S. J. Thompson, P. M. Maitlis, *J. Chem. Soc., Dalton Trans.* **1977**, 1654.

[2] F. Texierboullet, B. Klein, J. Hamelin, *Synthesis* **1986**, 409.

[3] S. Guillou, F. J. Bonhomme, M. S. Ermolenko, Y. L. Janin, *Tetrahedron* **2011**, *67*, 8451.

[4] Bruker, Version 6.10 ed., Bruker Inc, Madison, Wisconsin, USA, **1998-2000**.

[5] L. J. Farrugia, *J. Appl. Cryst.* **1997**, *30*, 565.

3. ^{1}H and ^{13}C NMR spectra

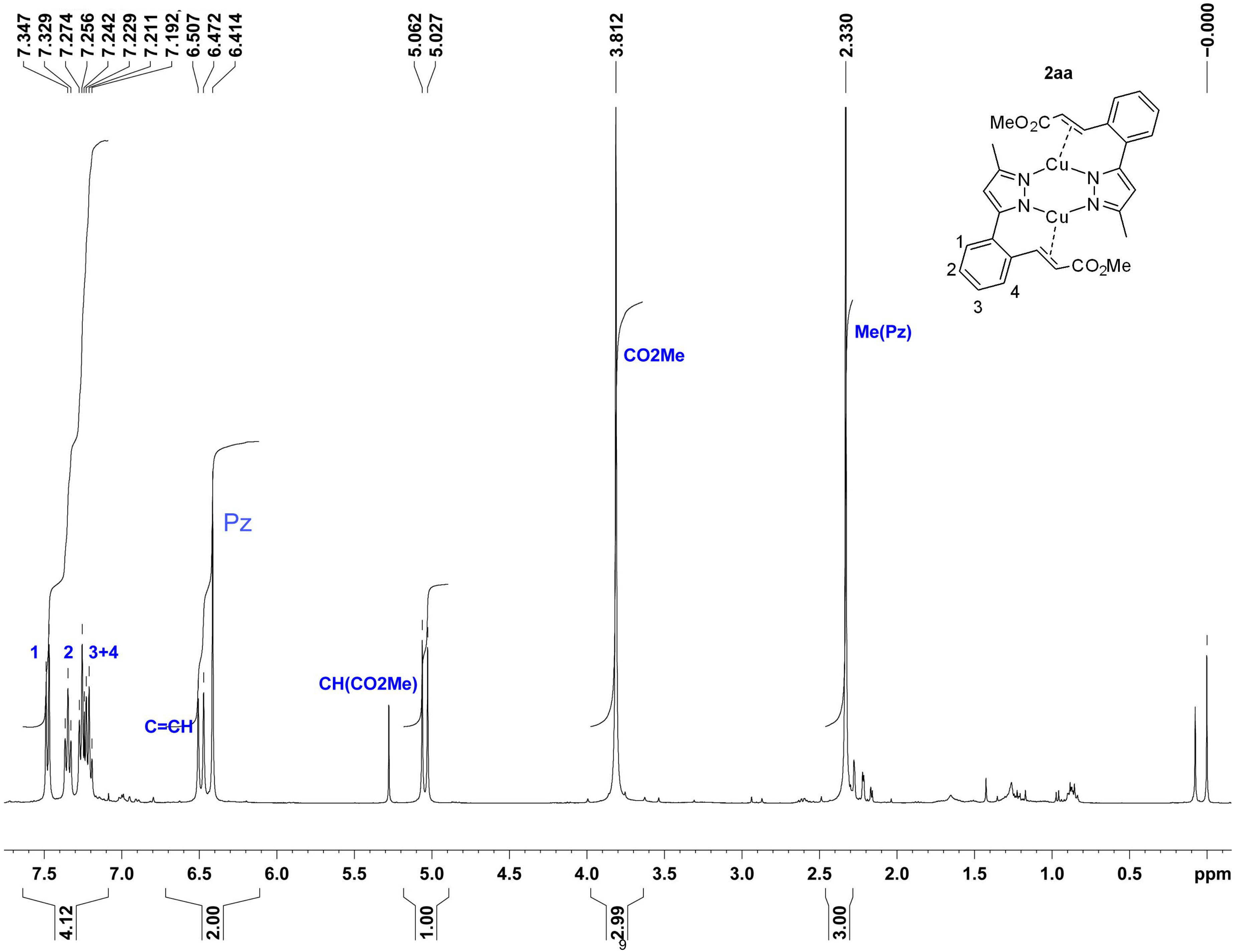

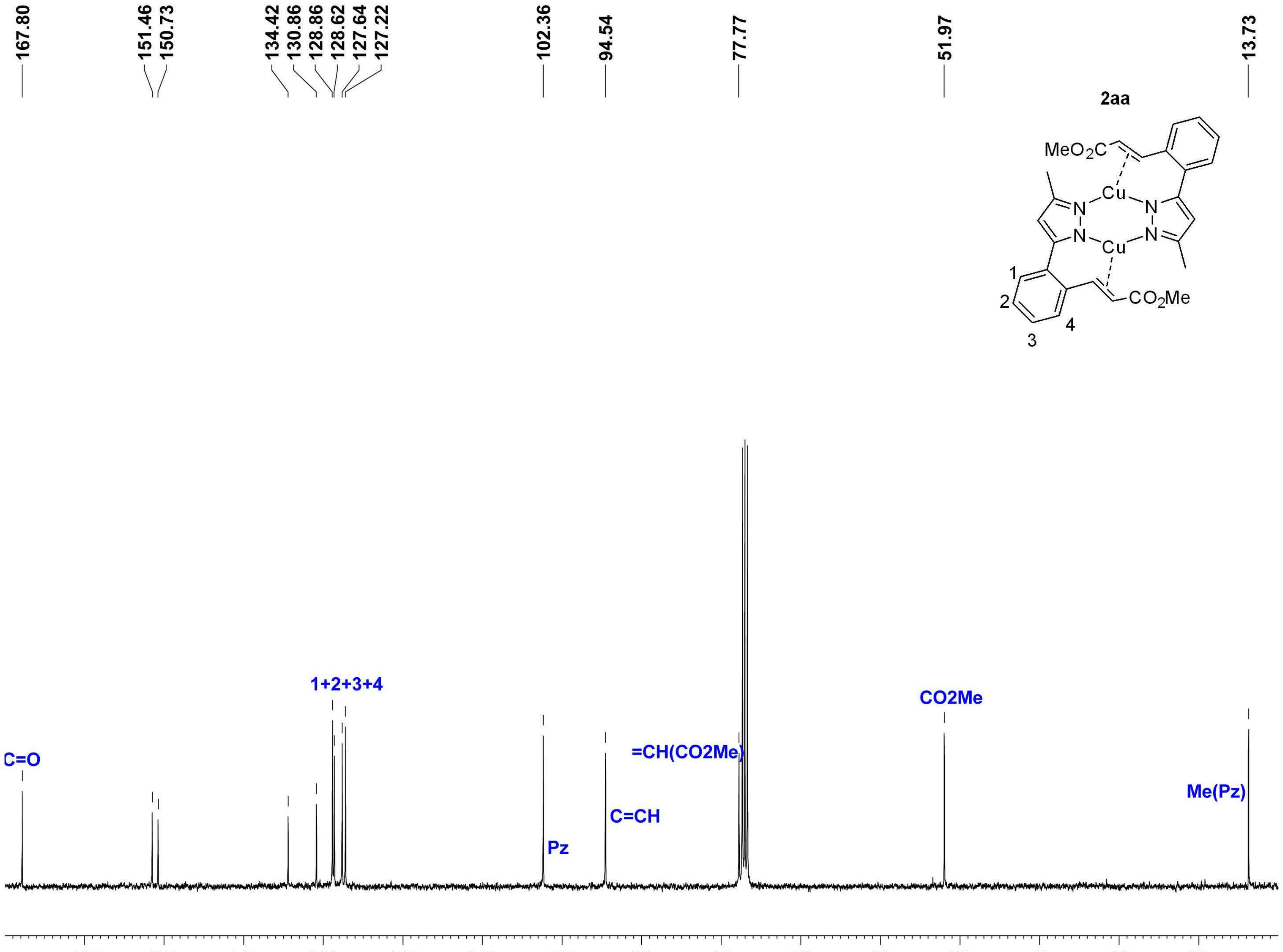

167.80
151.46
150.73
134.42
130.86
128.86
128.62
127.64
127.22
102.36
94.54
77.77
51.97
13.73
2aa
MeO2C
Cu
N
CO2Me
1
2
3
4
C=O
1+2+3+4
Pz
C=CH
=CH(CO2Me)
CO2Me
Me(Pz)
160
150
140
130
120
110
100
90
80
70
60
50
40
30
20
ppm

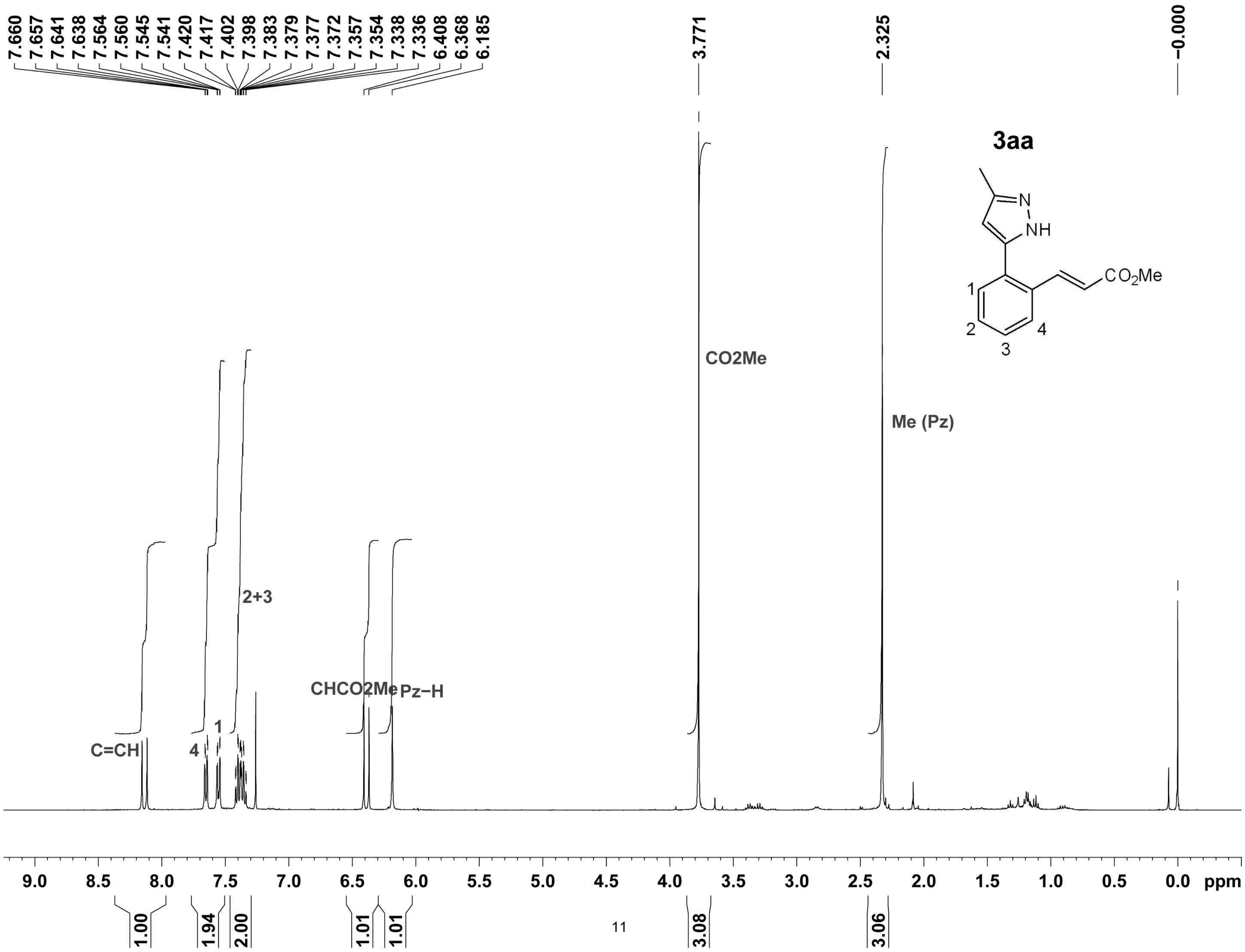
7.660
7.657
7.641
7.638
7.564
7.560
7.545
7.541
7.420
7.417
7.402
7.398
7.383
7.379
7.377
7.372
7.357
7.354
7.338
7.336
6.408
6.368
6.185
3.771
2.325
-0.000
3aa
N
NH
CO_2Me
1
2
3
4
CO2Me
Me (Pz)
2+3
CHCO2Me
Pz−H
C=CH
4
1
9.0
8.5
8.0
7.5
7.0
6.5
6.0
5.5
5.0
4.5
4.0
3.5
3.0
2.5
2.0
1.5
1.0
0.5
0.0
ppm
1.00
1.94
2.00
1.01
1.01
3.08
3.06

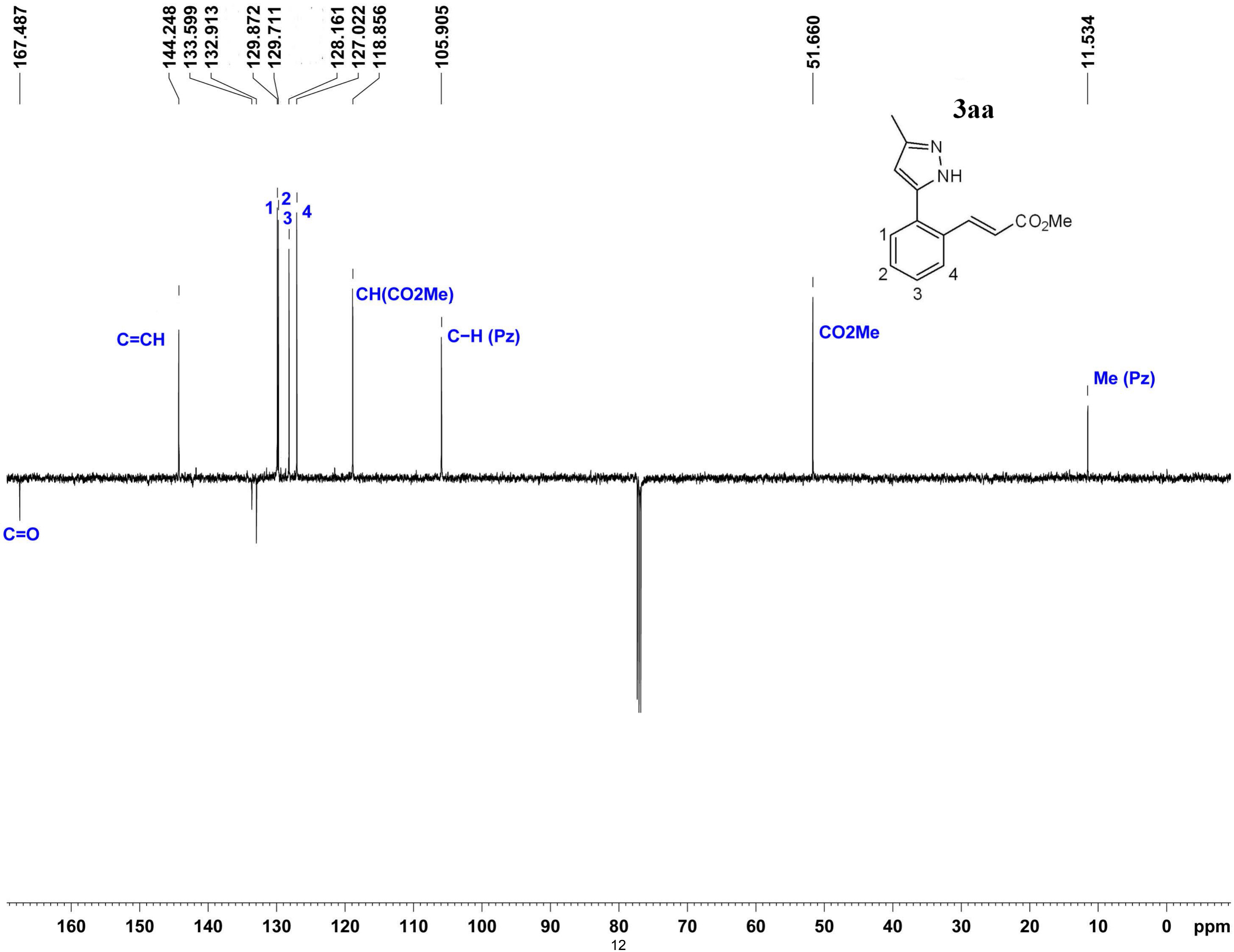
167.487
144.248
133.599
132.913
129.872
129.711
128.161
127.022
118.856
105.905
51.660
11.534
3aa
N
NH
CO2Me
1
2
3
4
C=CH
CH(CO2Me)
C−H (Pz)
CO2Me
Me (Pz)
C=O
160
150
140
130
120
110
100
90
80
70
60
50
40
30
20
10
0
ppm

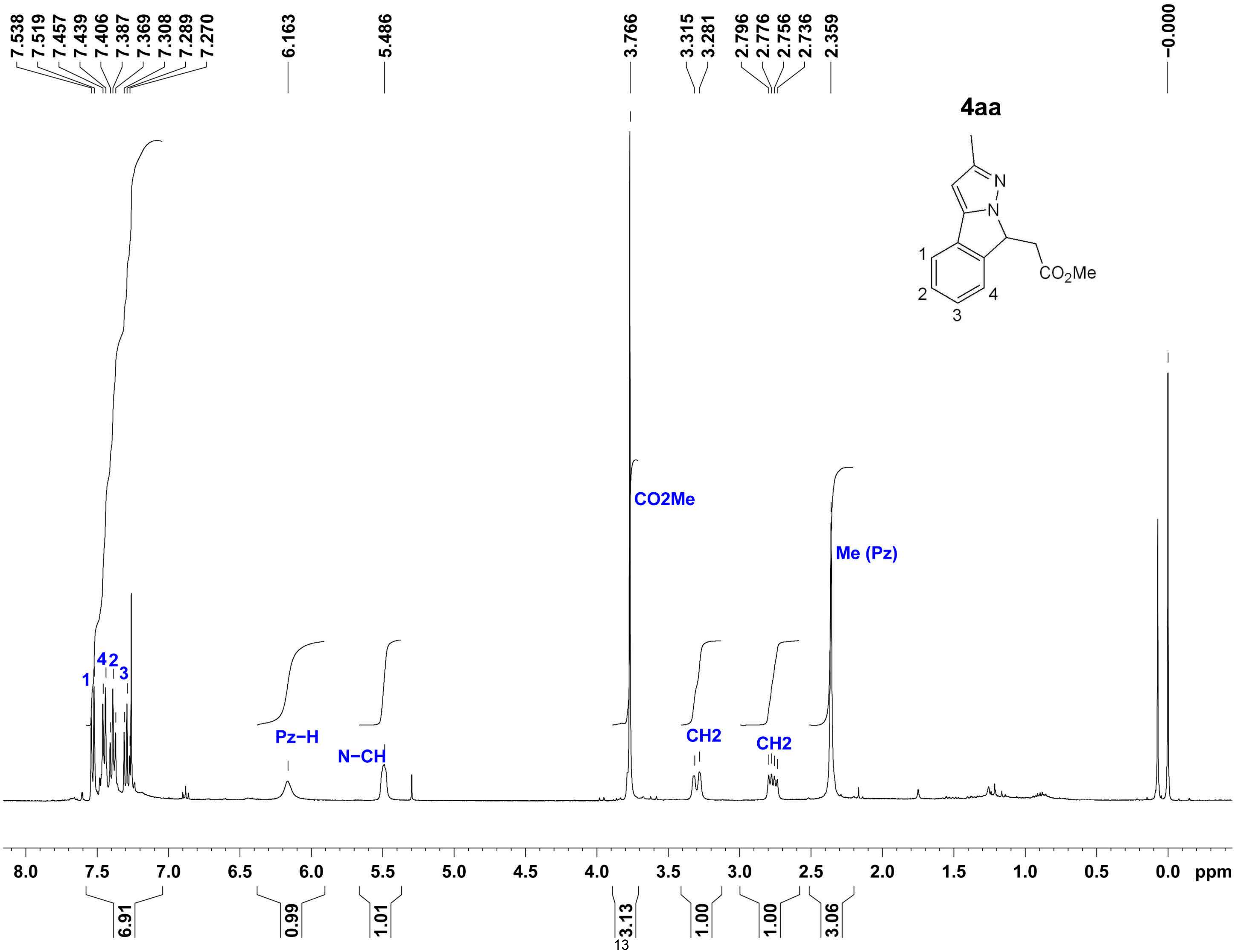
7.538
7.519
7.457
7.439
7.406
7.387
7.369
7.308
7.289
7.270
6.163
5.486
3.766
3.315
3.281
2.796
2.776
2.756
2.736
2.359
-0.000
4aa
N
N
1
2
3
4
CO_2Me
CO2Me
Me (Pz)
1
4
2
3
Pz−H
N−CH
CH2
CH2
8.0
7.5
7.0
6.5
6.0
5.5
5.0
4.5
4.0
3.5
3.0
2.5
2.0
1.5
1.0
0.5
0.0
ppm
6.91
0.99
1.01
3.13
1.00
1.00
3.06

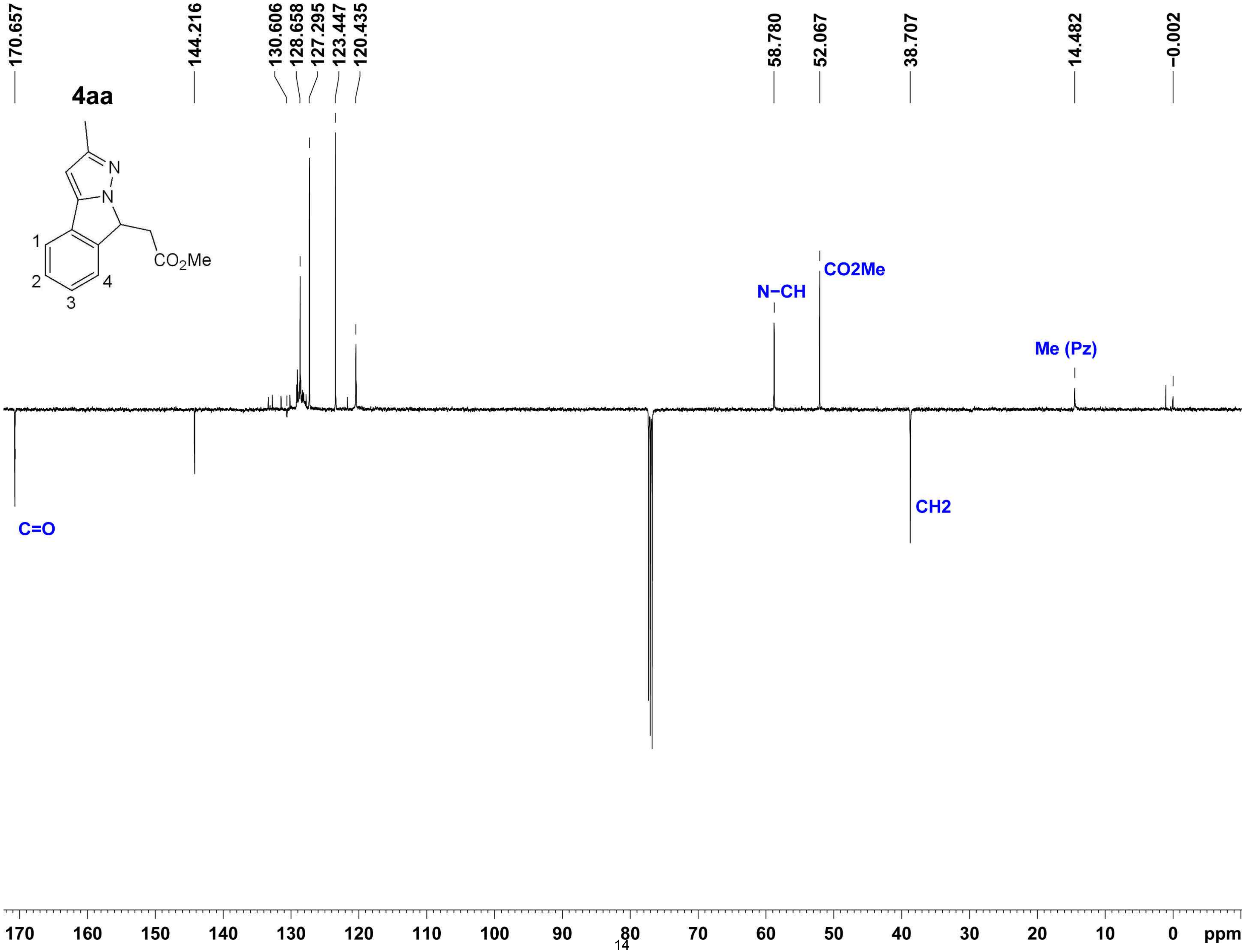

170.657
144.216
130.606
128.658
127.295
123.447
120.435
58.780
52.067
38.707
14.482
-0.002
4aa
N
N
1
2
3
4
CO2Me
N-CH
CO2Me
Me (Pz)
C=O
CH2
170
160
150
140
130
120
110
100
90
80
70
60
50
40
30
20
10
0
ppm

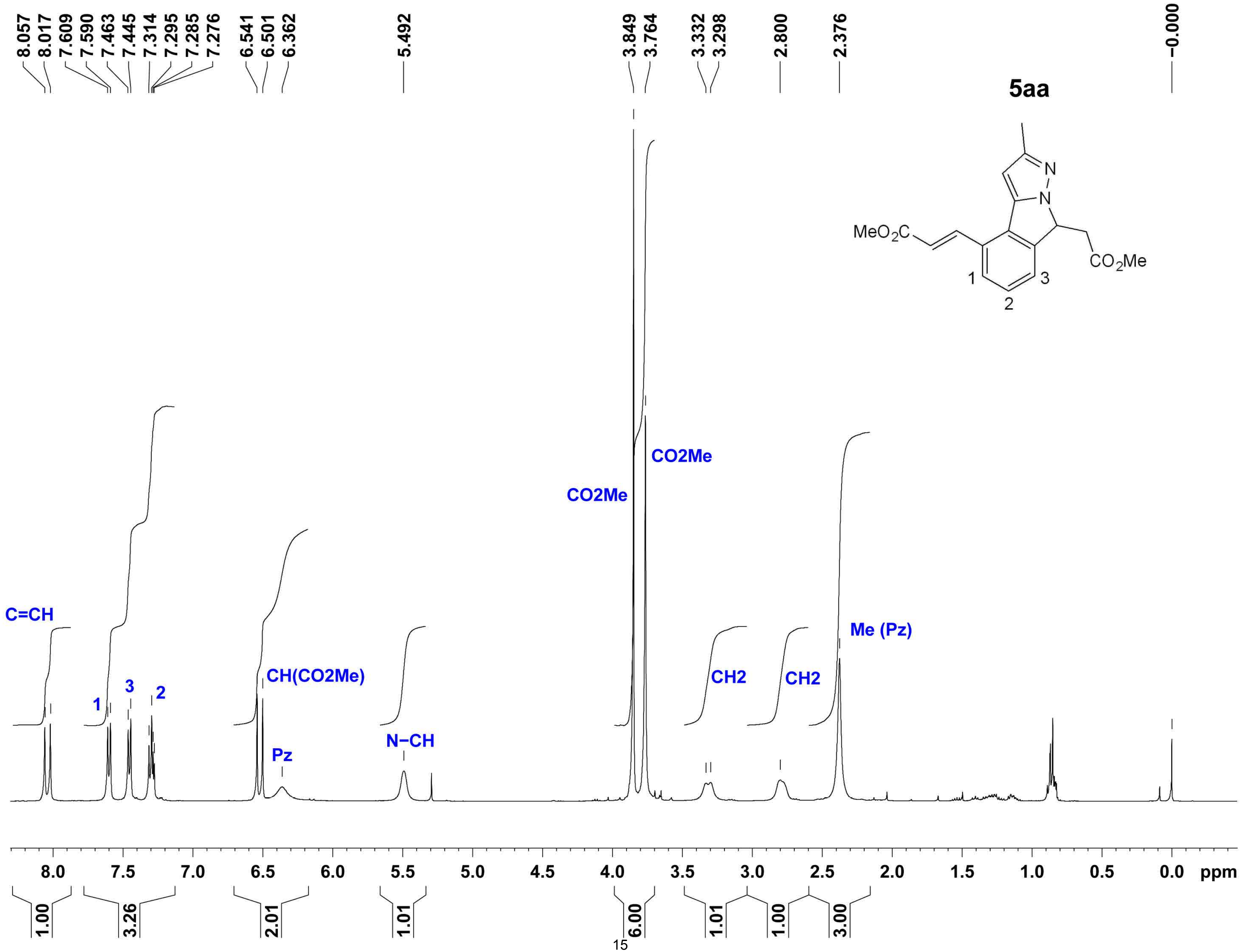

8.057
8.017
7.609
7.590
7.463
7.445
7.314
7.295
7.285
7.276
6.541
6.501
6.362
5.492
3.849
3.764
3.332
3.298
2.800
2.376
-0.000
5aa
MeO2C
CO2Me
N
N
1
2
3
C=CH
CH(CO2Me)
Pz
N−CH
CO2Me
CO2Me
CH2
CH2
Me (Pz)
8.0
7.5
7.0
6.5
6.0
5.5
5.0
4.5
4.0
3.5
3.0
2.5
2.0
1.5
1.0
0.5
0.0
ppm
1.00
3.26
2.01
1.01
6.00
1.01
1.00
3.00

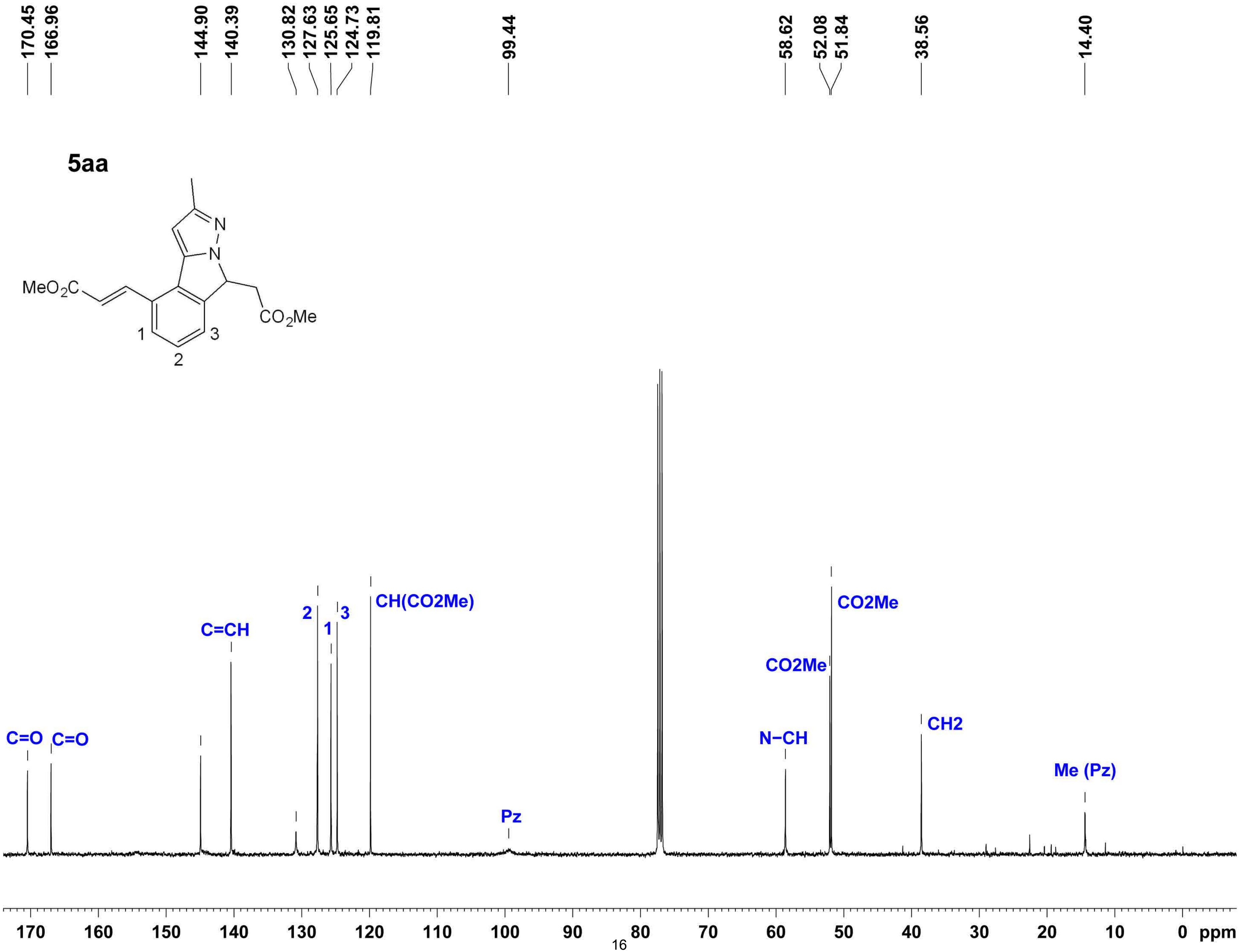
170.45
166.96
144.90
140.39
130.82
127.63
125.65
124.73
119.81
99.44
58.62
52.08
51.84
38.56
14.40
5aa
N
N
MeO2C
CO2Me
1
2
3
C=O
C=O
C=CH
2
1
3
CH(CO2Me)
Pz
N−CH
CO2Me
CO2Me
CH2
Me (Pz)
170 160 150 140 130 120 110 100 90 80 70 60 50 40 30 20 10 0 ppm

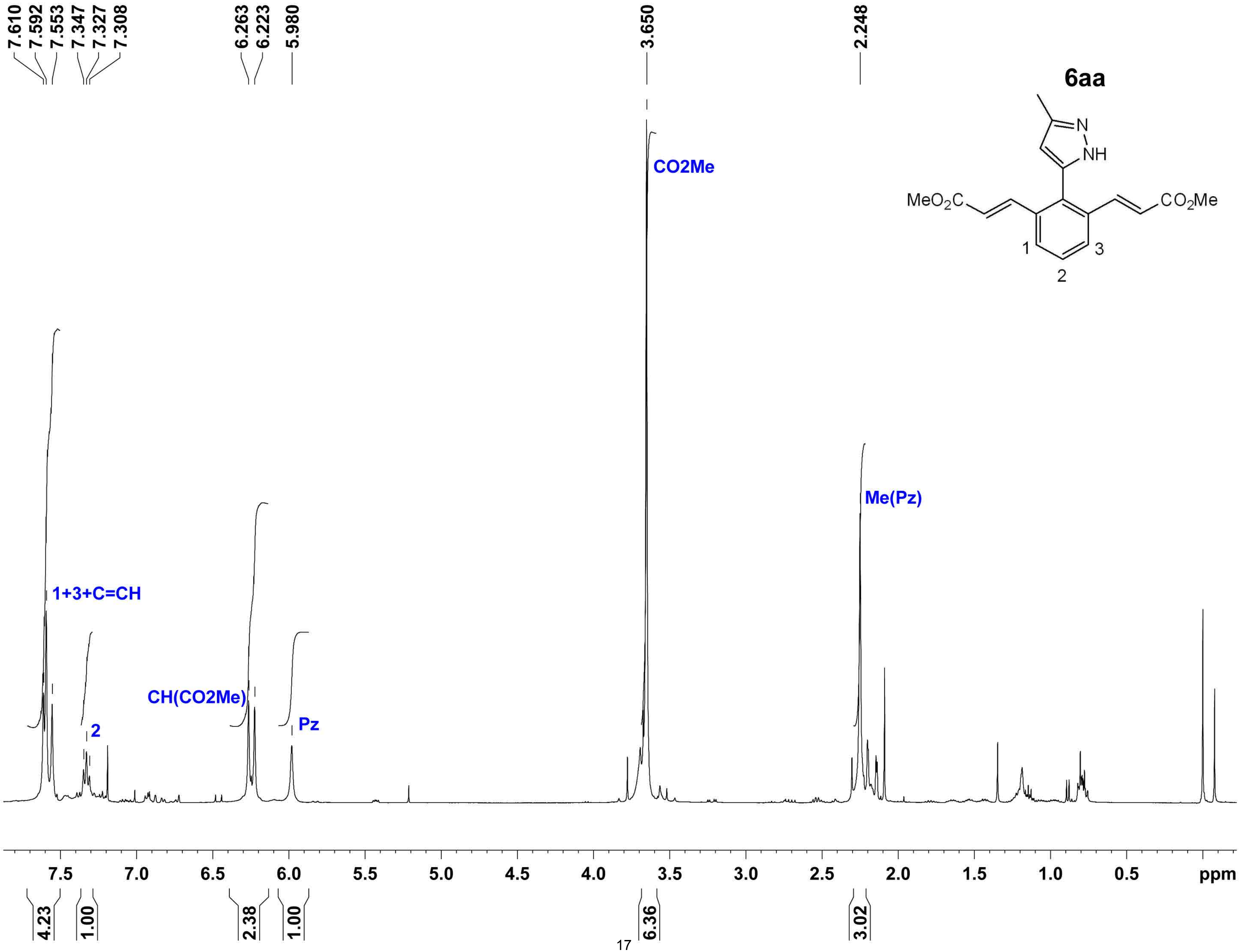
7.610
7.592
7.553
7.347
7.327
7.308
6.263
6.223
5.980
3.650
2.248
6aa
N
NH
MeO2C
CO2Me
1
2
3
CO2Me
Me(Pz)
1+3+C=CH
CH(CO2Me)
2
Pz
7.5
7.0
6.5
6.0
5.5
5.0
4.5
4.0
3.5
3.0
2.5
2.0
1.5
1.0
0.5
ppm
4.23
1.00
2.38
1.00
6.36
3.02

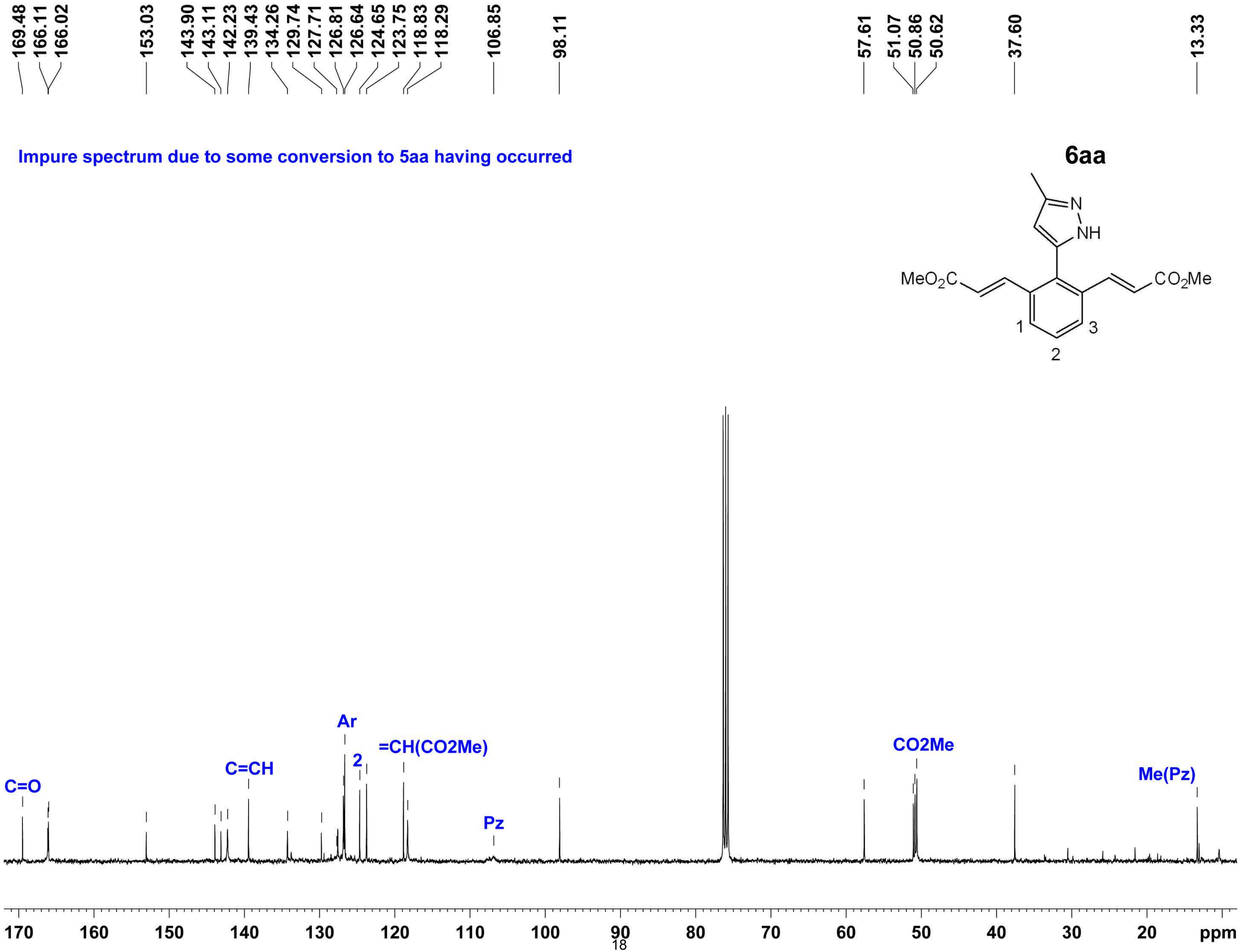
169.48
166.11
166.02
153.03
143.90
143.11
142.23
139.43
134.26
129.74
127.71
126.81
126.64
124.65
123.75
118.83
118.29
106.85
98.11
57.61
51.07
50.86
50.62
37.60
13.33
Impure spectrum due to some conversion to 5aa having occurred
6aa
N
NH
MeO_2C
CO_2Me
1
2
3
C=O
C=CH
Ar
2
=CH(CO2Me)
Pz
CO2Me
Me(Pz)
170
160
150
140
130
120
110
100
90
80
70
60
50
40
30
20
ppm

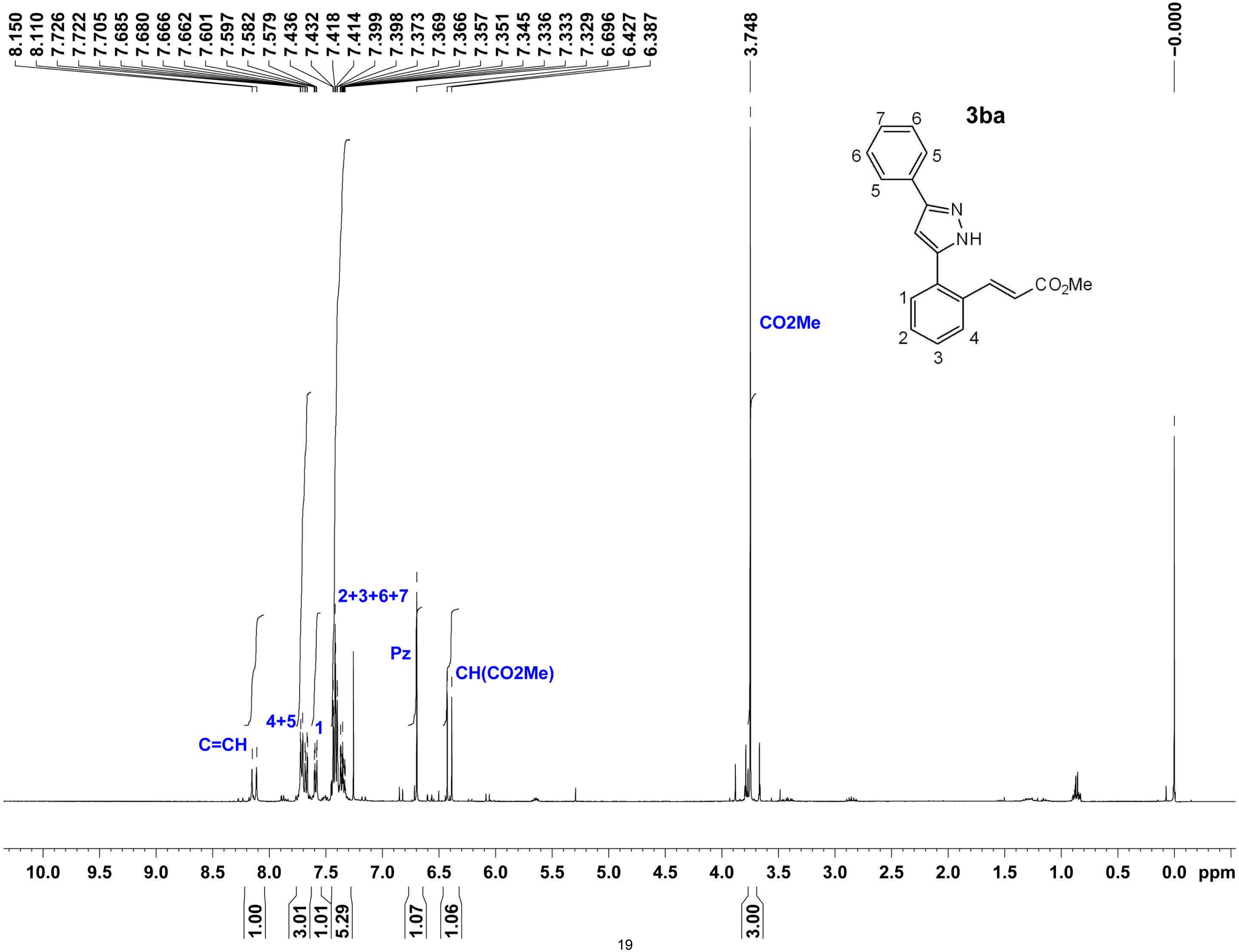
8.150
8.110
7.726
7.722
7.705
7.685
7.680
7.666
7.662
7.601
7.597
7.582
7.579
7.436
7.432
7.418
7.414
7.399
7.398
7.373
7.369
7.366
7.357
7.351
7.345
7.336
7.333
7.329
6.696
6.427
6.387
3.748
-0.000
3ba
CO2Me
C=CH
4+5
1
2+3+6+7
Pz
CH(CO2Me)
N
NH
CO_2Me
10.0
9.5
9.0
8.5
8.0
7.5
7.0
6.5
6.0
5.5
5.0
4.5
4.0
3.5
3.0
2.5
2.0
1.5
1.0
0.5
0.0
ppm
1.00
3.01
1.01
5.29
1.07
1.06
3.00

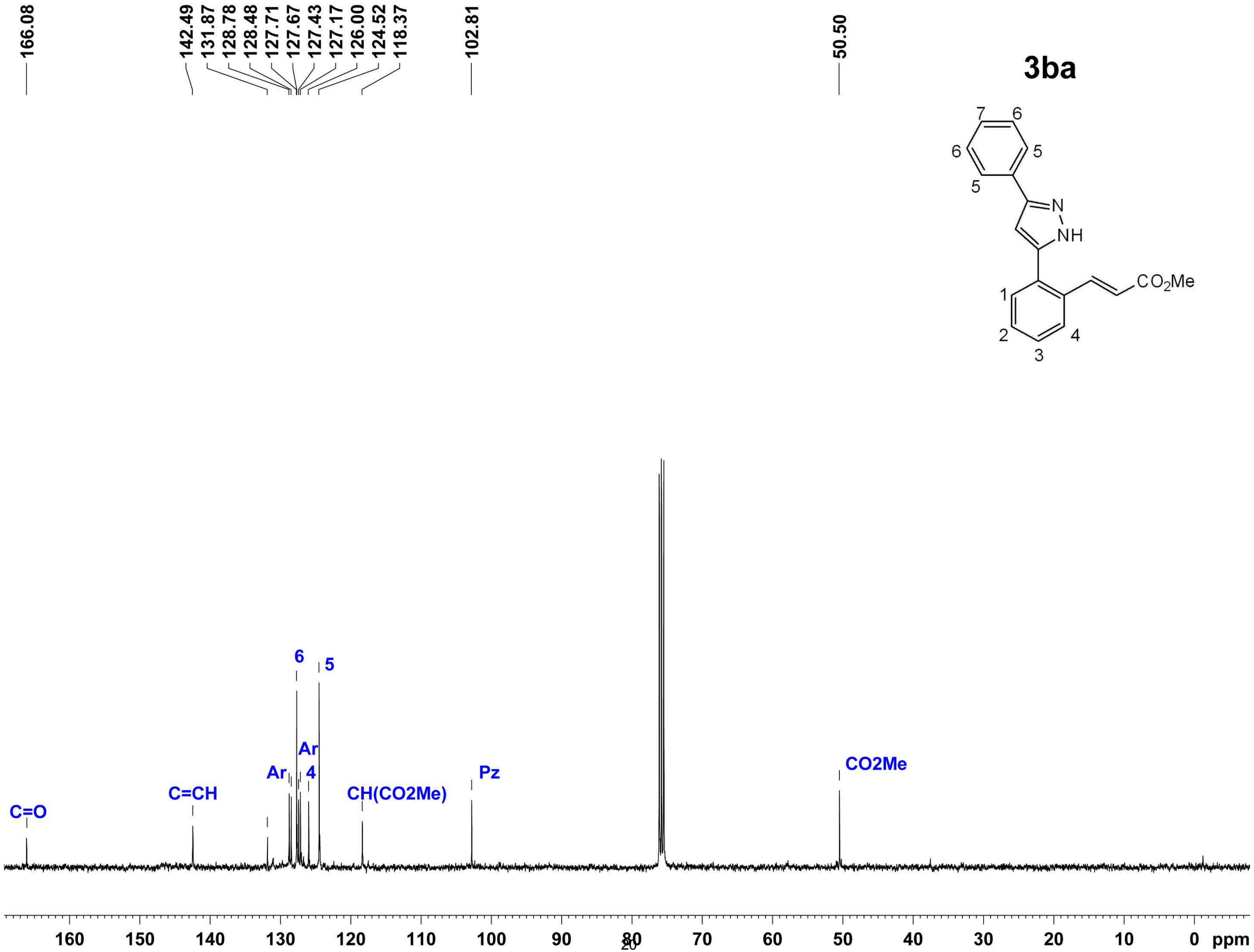

166.08
142.49
131.87
128.78
128.48
127.71
127.67
127.43
127.17
126.00
124.52
118.37
102.81
50.50
3ba
N
NH
CO_2Me
C=O
C=CH
Ar
6
Ar
4
5
CH(CO2Me)
Pz
CO2Me
160
150
140
130
120
110
100
90
80
70
60
50
40
30
20
10
0
ppm

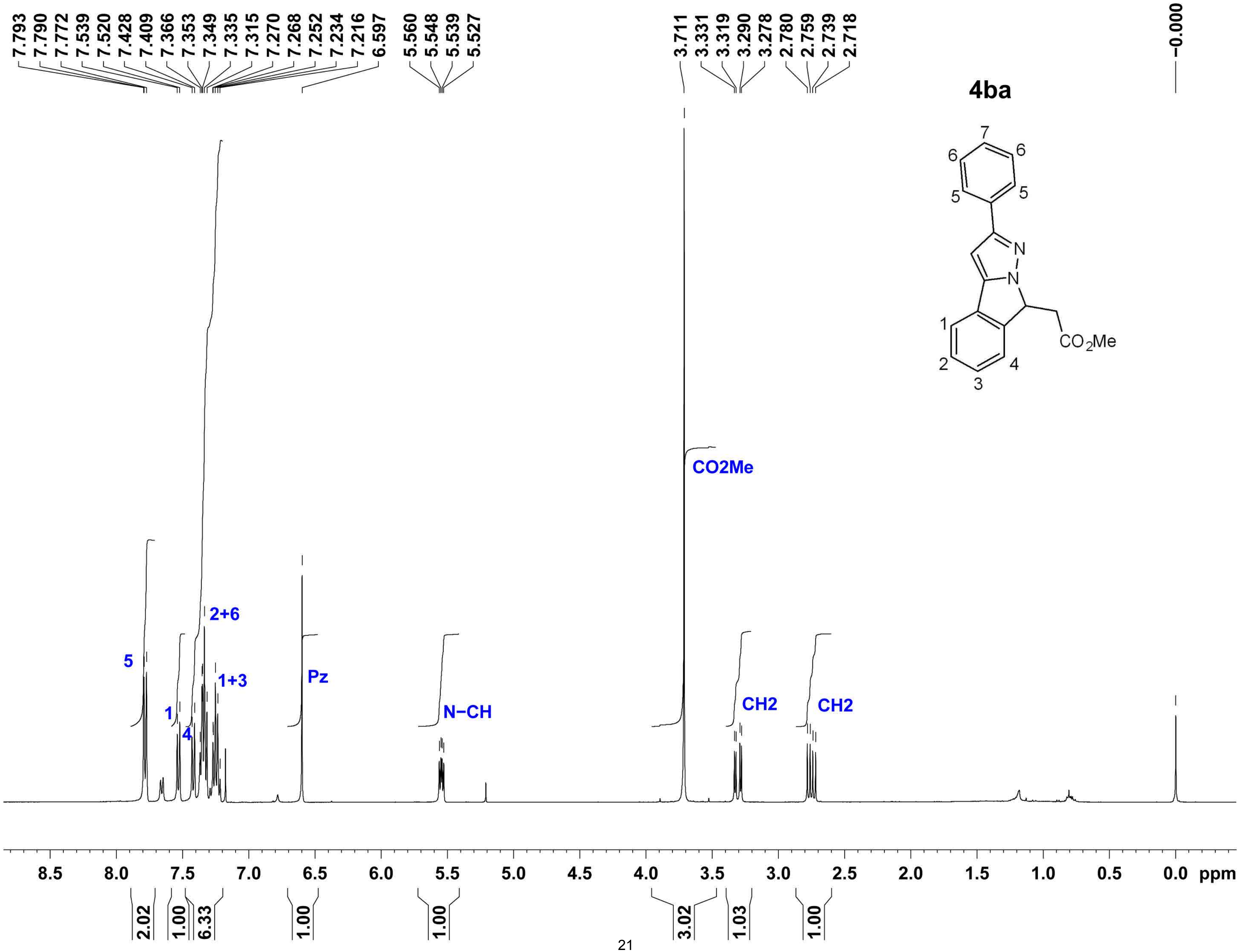

7.793
7.790
7.772
7.539
7.520
7.428
7.409
7.366
7.353
7.349
7.335
7.315
7.270
7.268
7.252
7.234
7.216
6.597
5.560
5.548
5.539
5.527
3.711
3.331
3.319
3.290
3.278
2.780
2.759
2.739
2.718
-0.000
4ba
5
1
4
2+6
1+3
Pz
N−CH
CO2Me
CH2
CH2
8.5
8.0
7.5
7.0
6.5
6.0
5.5
5.0
4.5
4.0
3.5
3.0
2.5
2.0
1.5
1.0
0.5
0.0 ppm
2.02
1.00
6.33
1.00
1.00
3.02
1.03
1.00

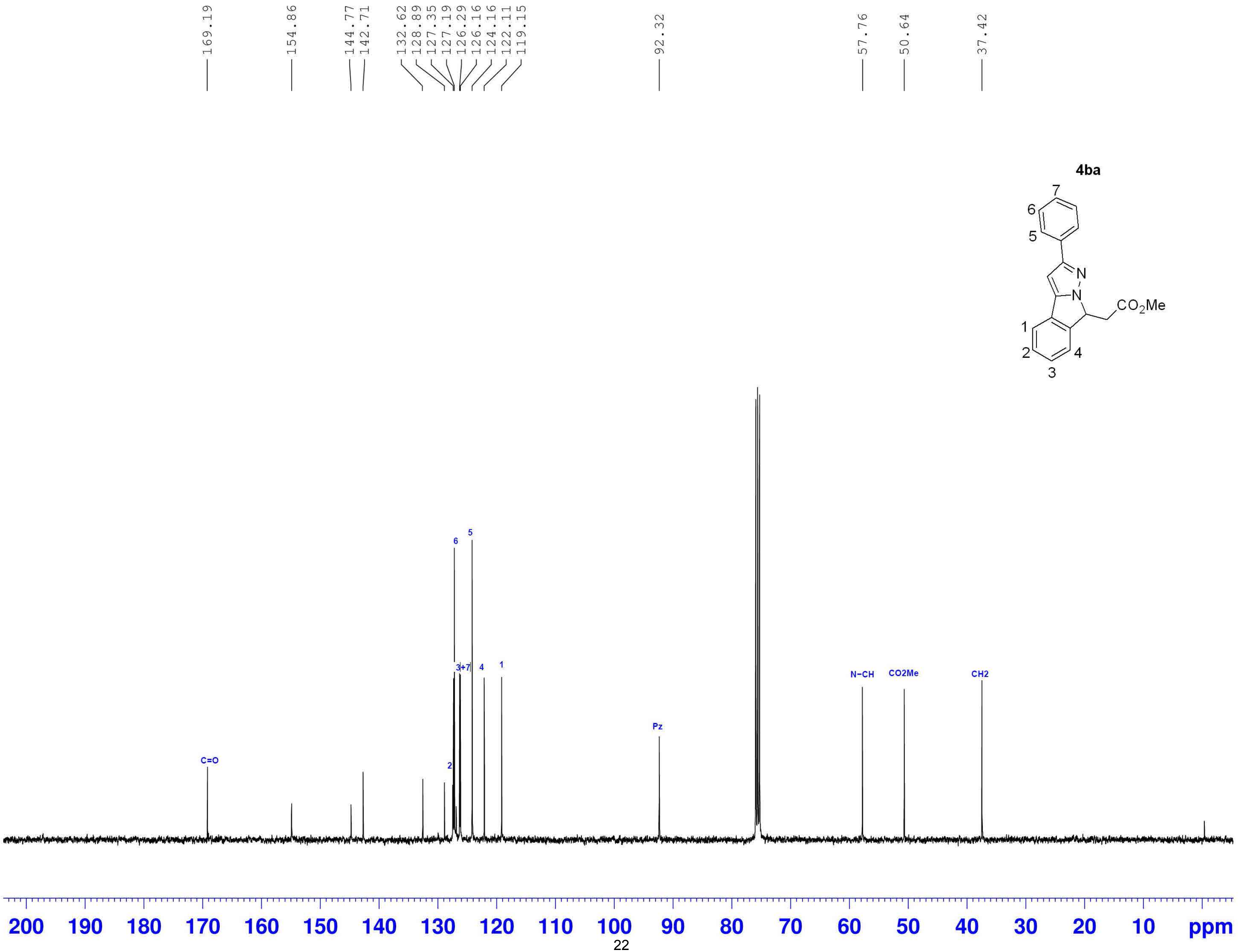
169.19
154.86
144.77
142.71
132.62
128.89
127.35
127.19
126.29
126.16
124.16
122.11
119.15
92.32
57.76
50.64
37.42
4ba
CO2Me
C=O
6
5
3+7
4
1
2
Pz
N−CH
CO2Me
CH2
200
190
180
170
160
150
140
130
120
110
100
90
80
70
60
50
40
30
20
10
ppm

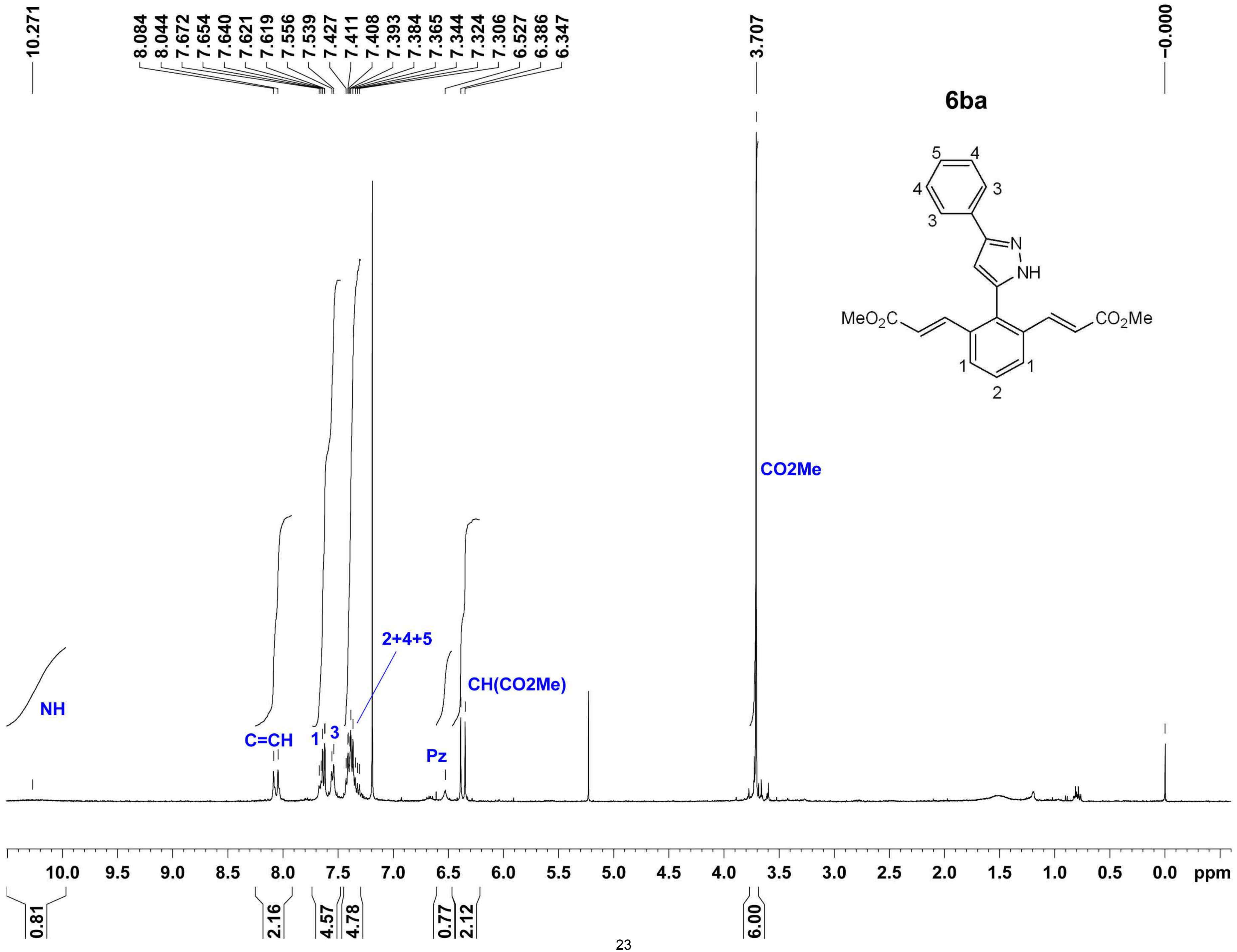

10.271
8.084
8.044
7.672
7.654
7.640
7.621
7.619
7.556
7.539
7.427
7.411
7.408
7.393
7.384
7.365
7.344
7.324
7.306
6.527
6.386
6.347
3.707
-0.000
6ba
MeO2C
CO2Me
NH
N
NH
C=CH
1
3
2+4+5
Pz
CH(CO2Me)
CO2Me
ppm
0.81
2.16
4.57
4.78
0.77
2.12
6.00

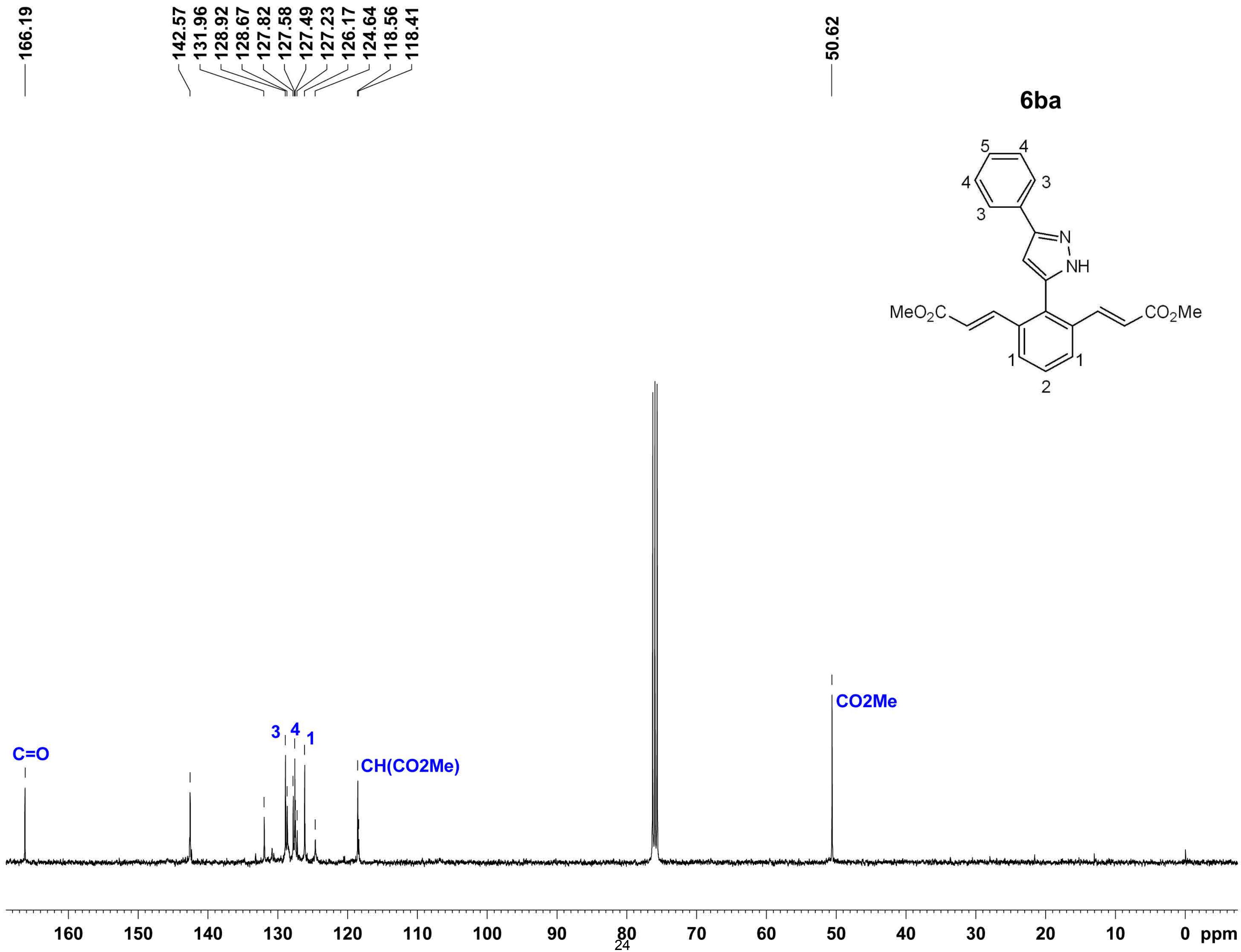
166.19
142.57
131.96
128.92
128.67
127.82
127.58
127.49
127.23
126.17
124.64
118.56
118.41
50.62
6ba
MeO2C
CO2Me
NH
N
C=O
3
4
1
CH(CO2Me)
CO2Me
160
150
140
130
120
110
100
90
80
70
60
50
40
30
20
10
0 ppm

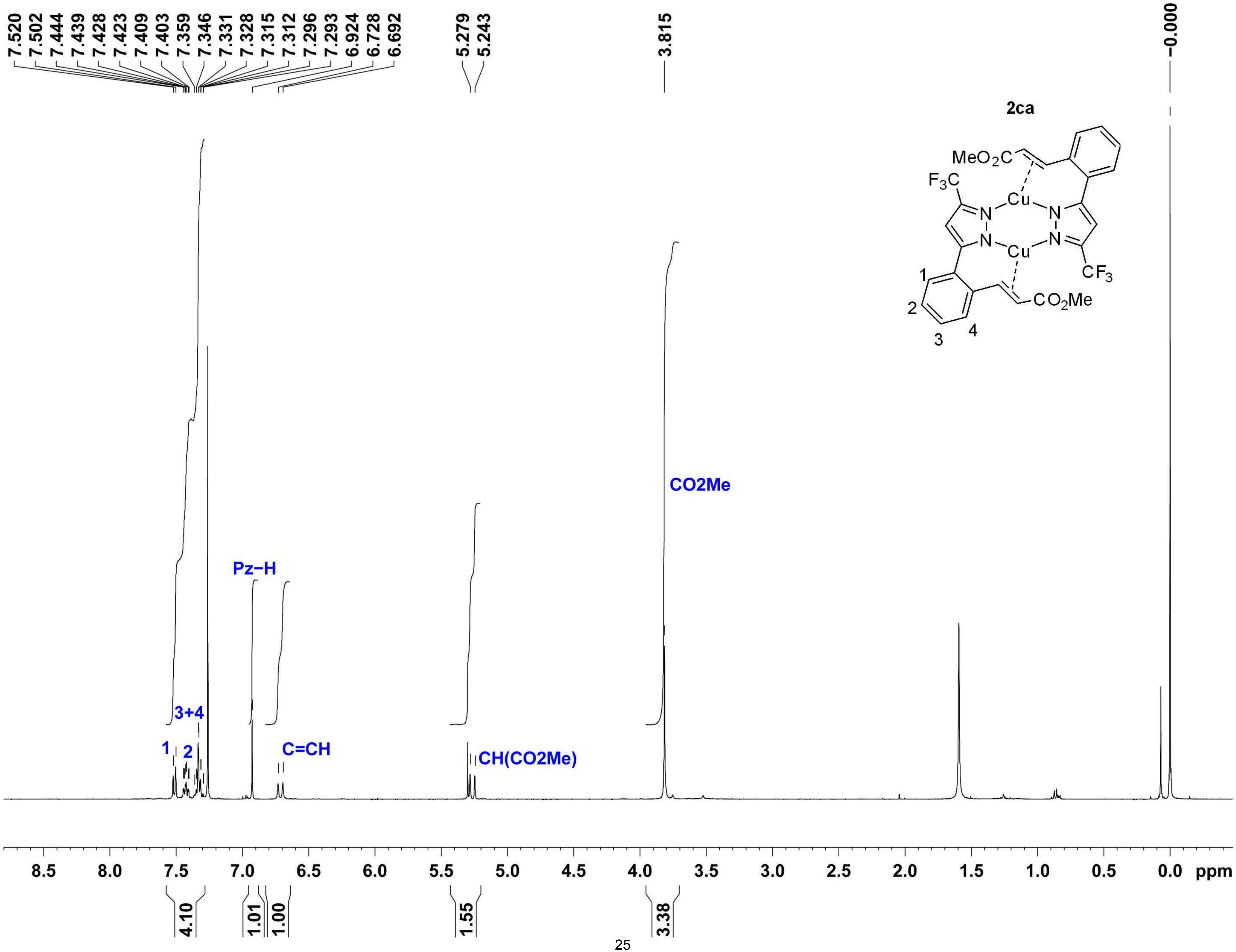
7.520
7.502
7.444
7.439
7.428
7.423
7.409
7.403
7.359
7.346
7.331
7.328
7.315
7.312
7.296
7.293
6.924
6.728
6.692
5.279
5.243
3.815
-0.000
2ca
CO2Me
Pz−H
3+4
1
2
C=CH
CH(CO2Me)
ppm
4.10
1.01
1.00
1.55
3.38

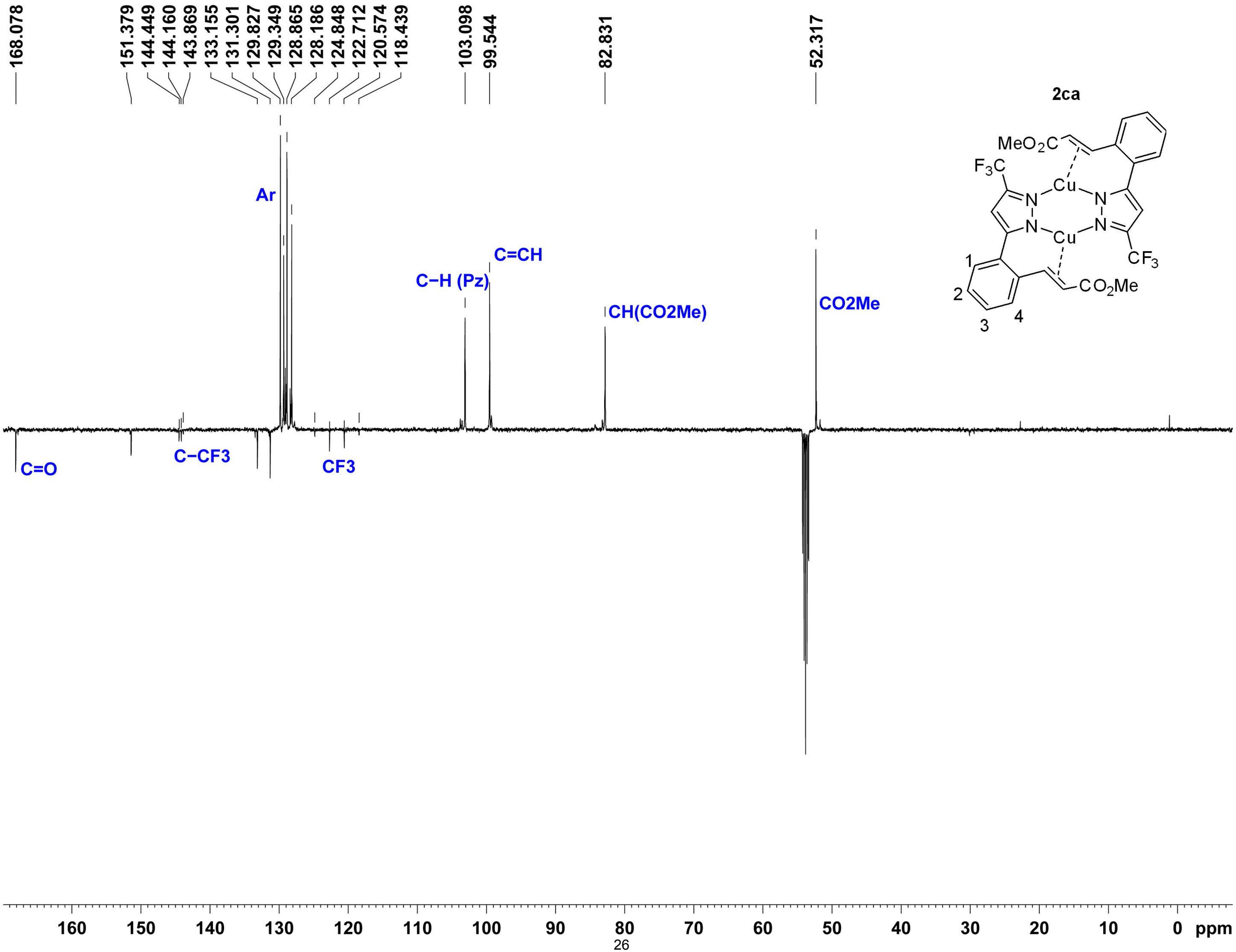

168.078
151.379
144.449
144.160
143.869
133.155
131.301
129.827
129.349
128.865
128.186
124.848
122.712
120.574
118.439
103.098
99.544
82.831
52.317
2ca
Ar
C=CH
C−H (Pz)
CH(CO2Me)
CO2Me
C=O
C−CF3
CF3
160
150
140
130
120
110
100
90
80
70
60
50
40
30
20
10
0
ppm

2ca

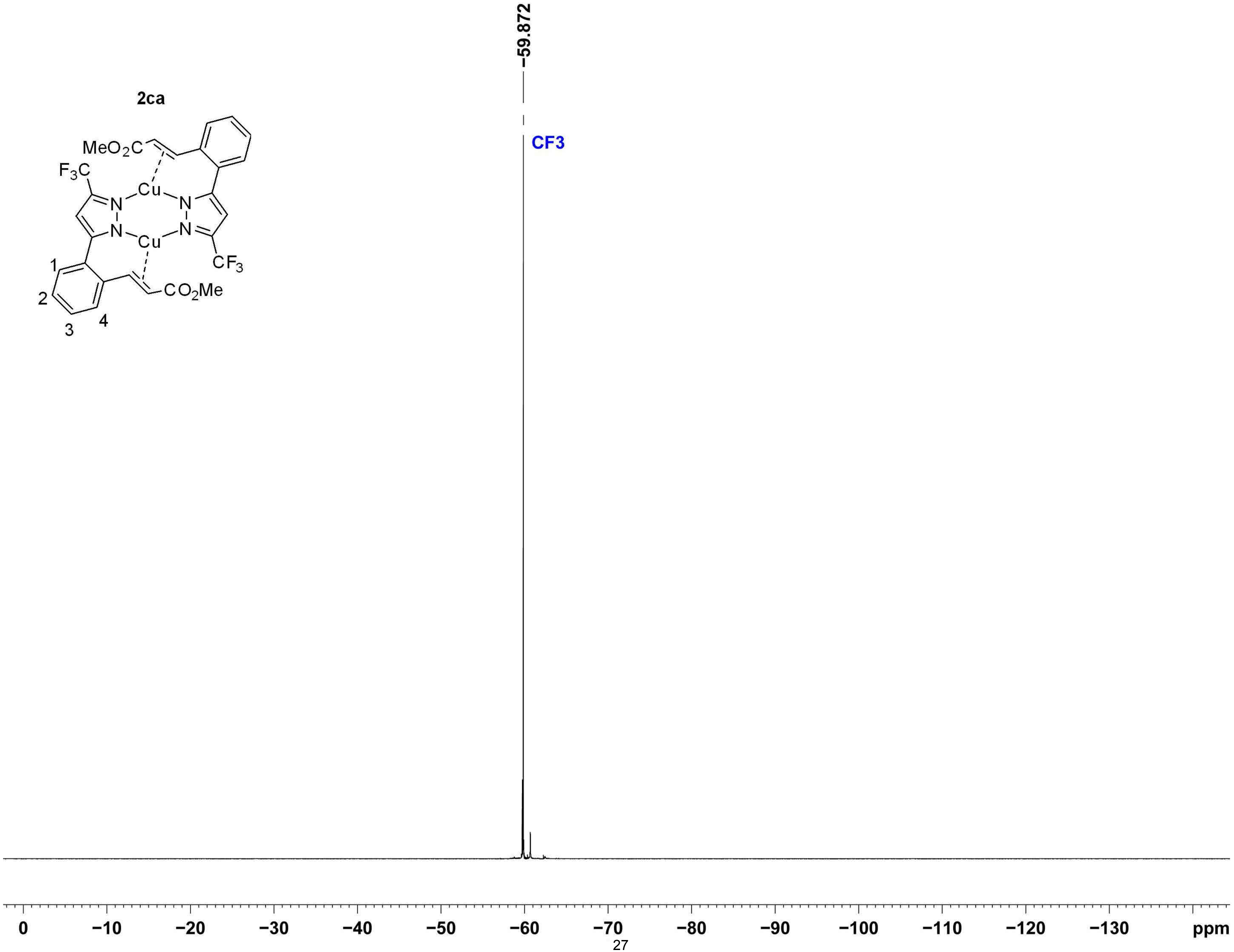
MeO2C
F3C
Cu
N
N
Cu
N
N
CF3
CO2Me
1
2
3
4
−59.872
CF3
0
−10
−20
−30
−40
−50
−60
−70
−80
−90
−100
−110
−120
−130
ppm

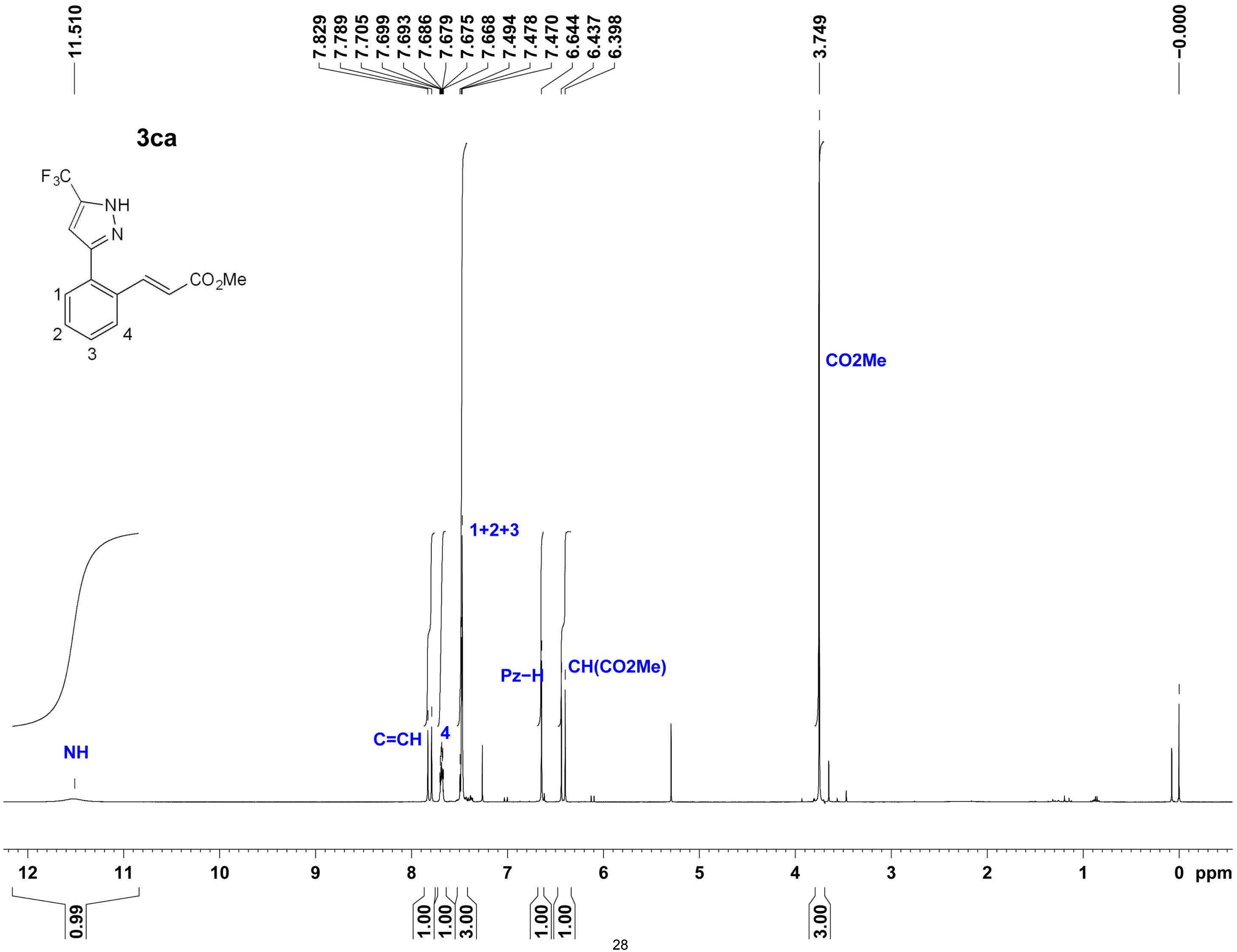

11.510
7.829
7.789
7.705
7.699
7.693
7.686
7.679
7.675
7.668
7.494
7.478
7.470
6.644
6.437
6.398
3.749
-0.000
3ca
F3C
NH
N
CO2Me
1
2
3
4
CO2Me
1+2+3
Pz−H
CH(CO2Me)
C=CH
4
NH
12
11
10
9
8
7
6
5
4
3
2
1
0 ppm
0.99
1.00
1.00
3.00
1.00
1.00
3.00

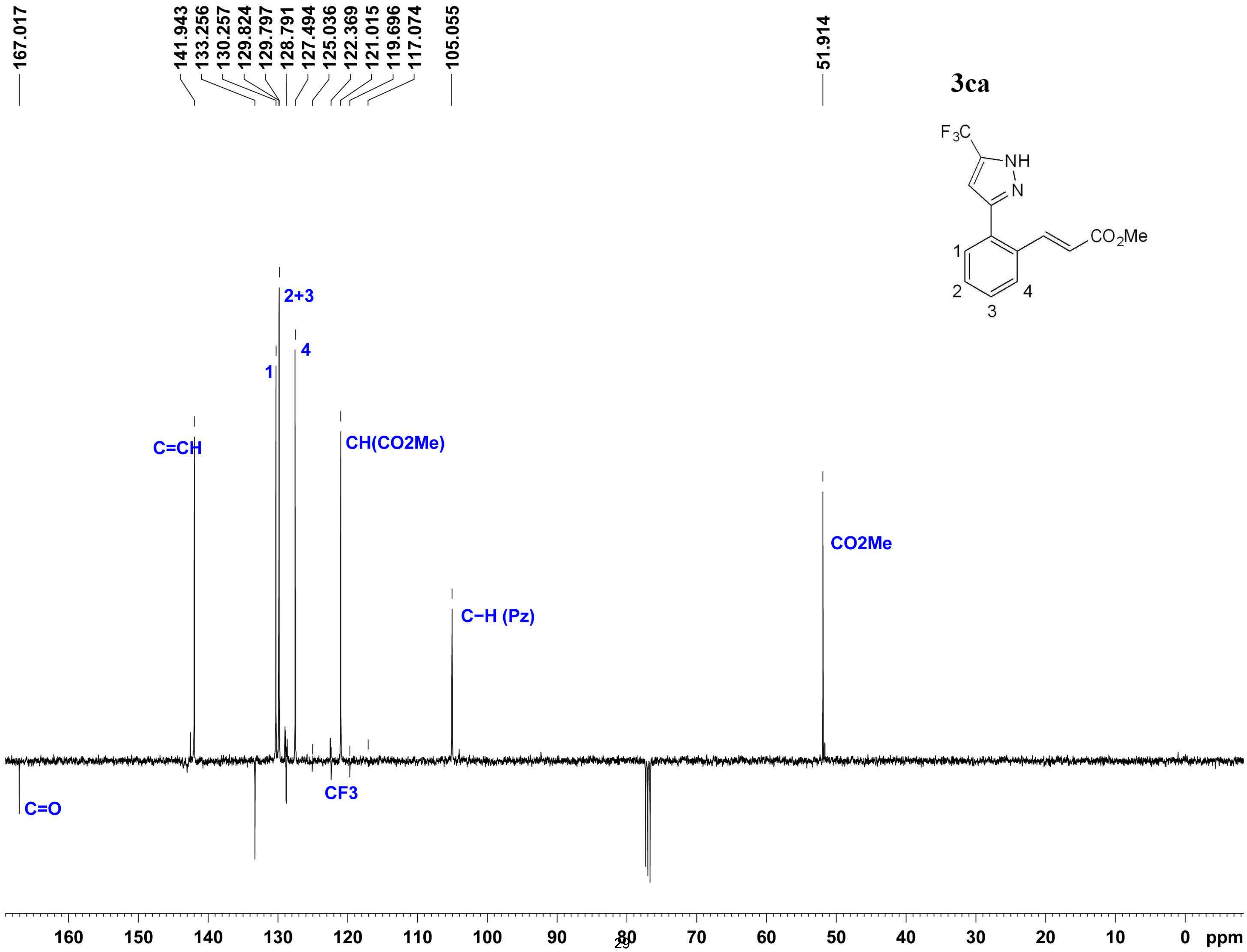

167.017
141.943
133.256
130.257
129.824
129.797
128.791
127.494
125.036
122.369
121.015
119.696
117.074
105.055
51.914
3ca
F_3C
NH
N
CO_2Me
1
2
3
4
2+3
4
1
C=CH
CH(CO2Me)
CO2Me
C−H (Pz)
C=O
CF3
160
150
140
130
120
110
100
90
80
70
60
50
40
30
20
10
0
ppm

3ca

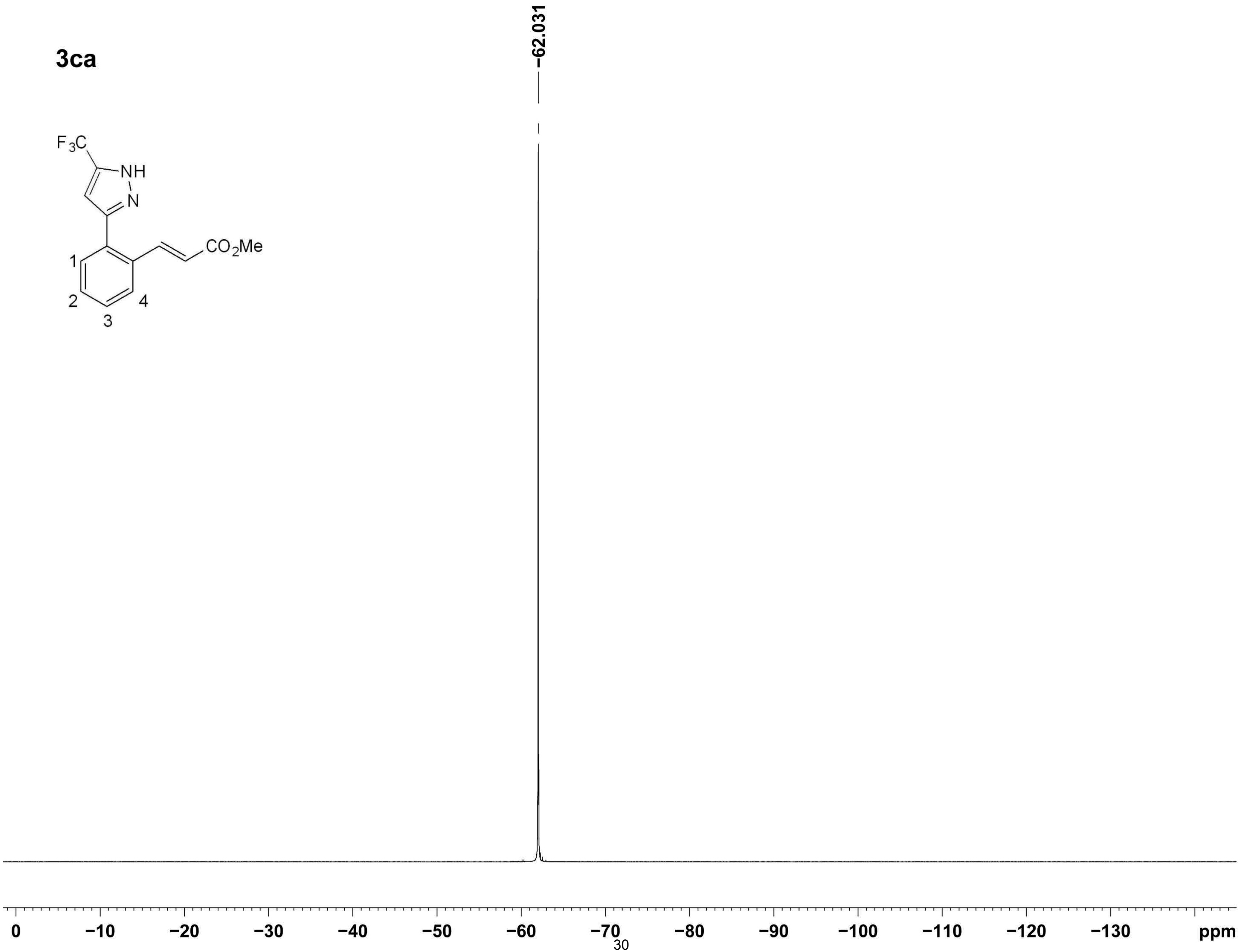
F3C
NH
N
CO2Me
1
2
3
4
−62.031
0
−10
−20
−30
−40
−50
−60
−70
−80
−90
−100
−110
−120
−130
ppm

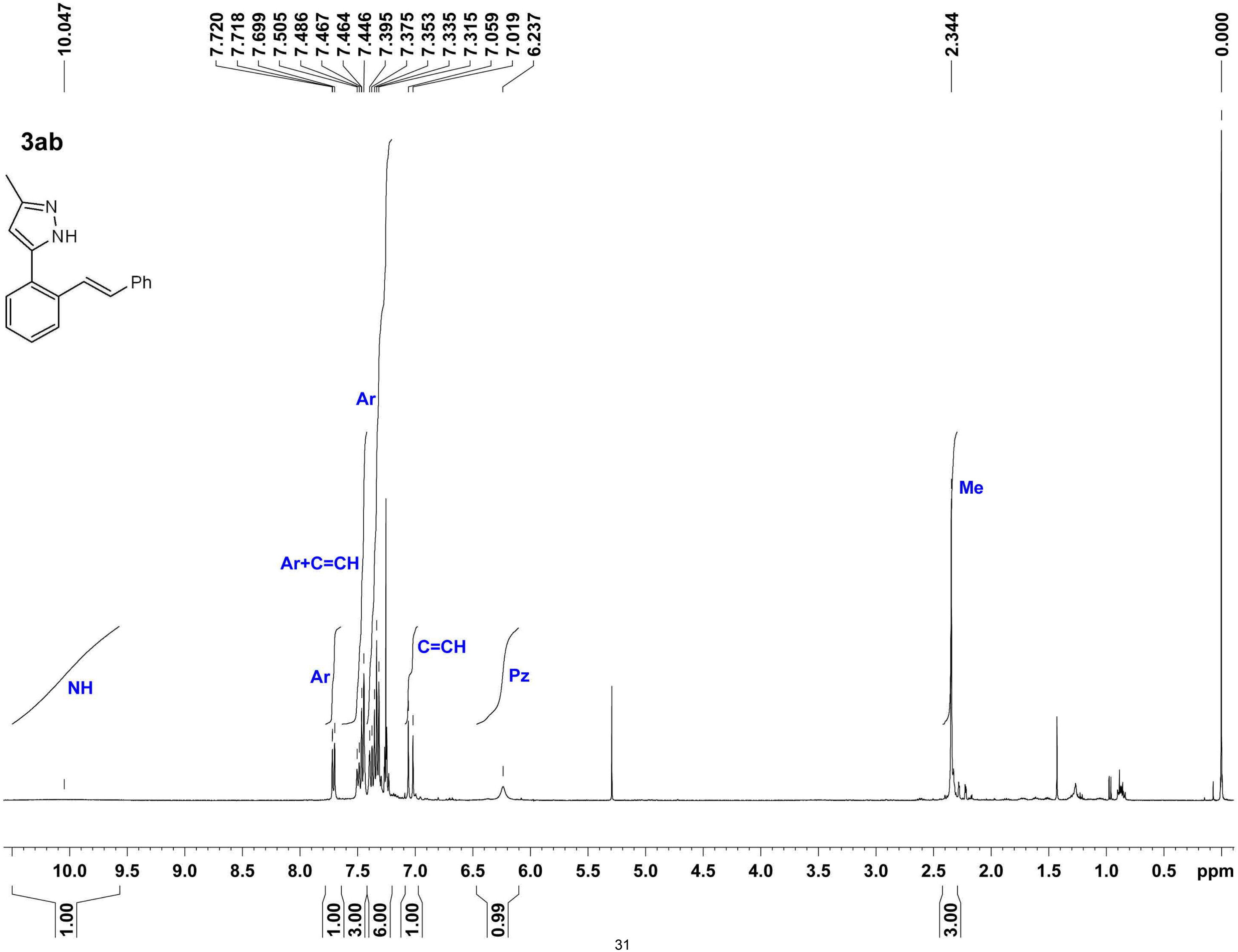

10.047
7.720
7.718
7.699
7.505
7.486
7.467
7.464
7.446
7.395
7.375
7.353
7.335
7.315
7.059
7.019
6.237
2.344
0.000
3ab
N
NH
Ph
Ar
Ar+C=CH
Ar
C=CH
Pz
NH
Me
10.0
9.5
9.0
8.5
8.0
7.5
7.0
6.5
6.0
5.5
5.0
4.5
4.0
3.5
3.0
2.5
2.0
1.5
1.0
0.5
ppm
1.00
1.00
3.00
6.00
1.00
0.99
3.00

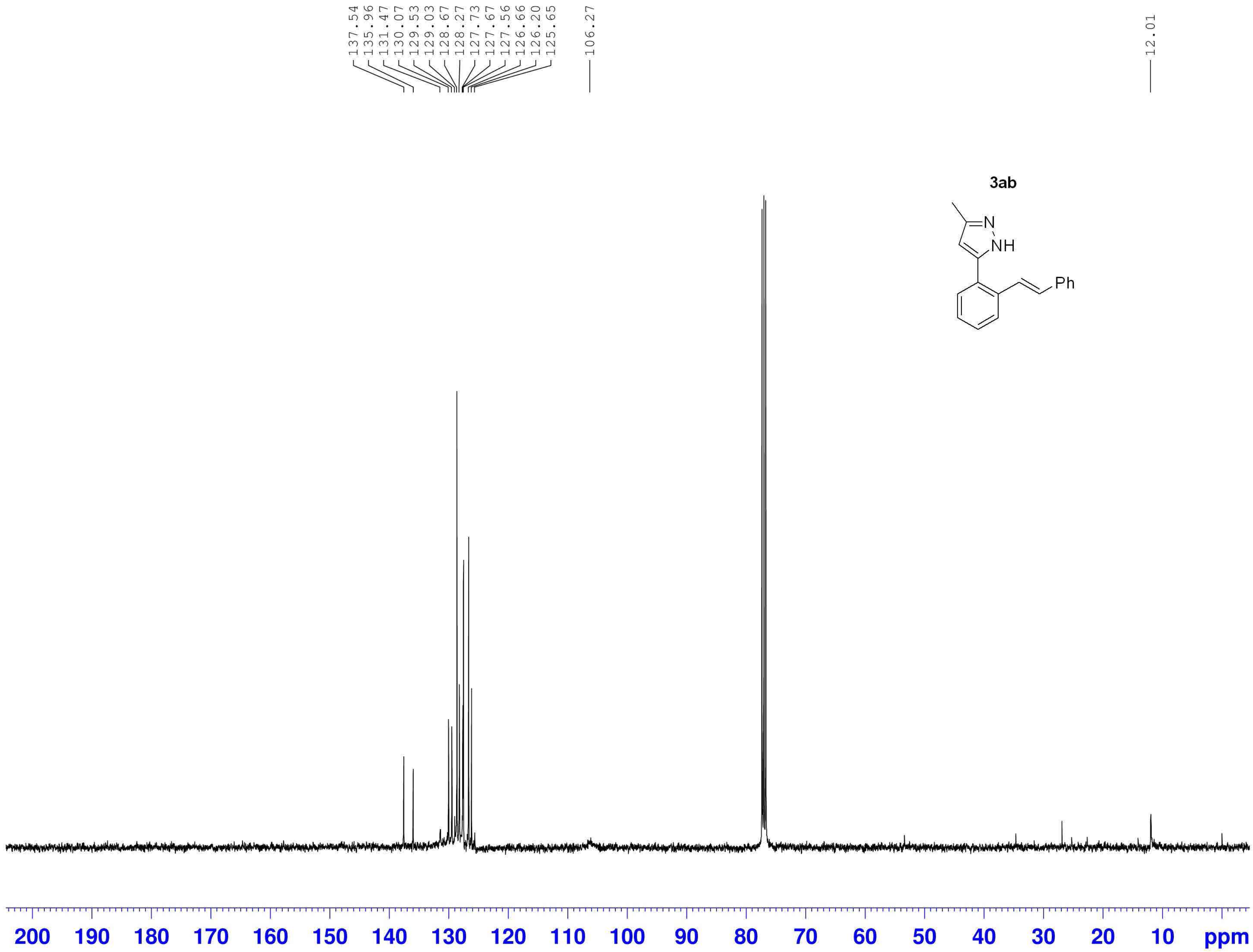
137.54
135.96
131.47
130.07
129.53
129.03
128.67
128.27
127.73
127.67
127.56
126.66
126.20
125.65
106.27
12.01
3ab
N
NH
Ph
200 190 180 170 160 150 140 130 120 110 100 90 80 70 60 50 40 30 20 10 ppm

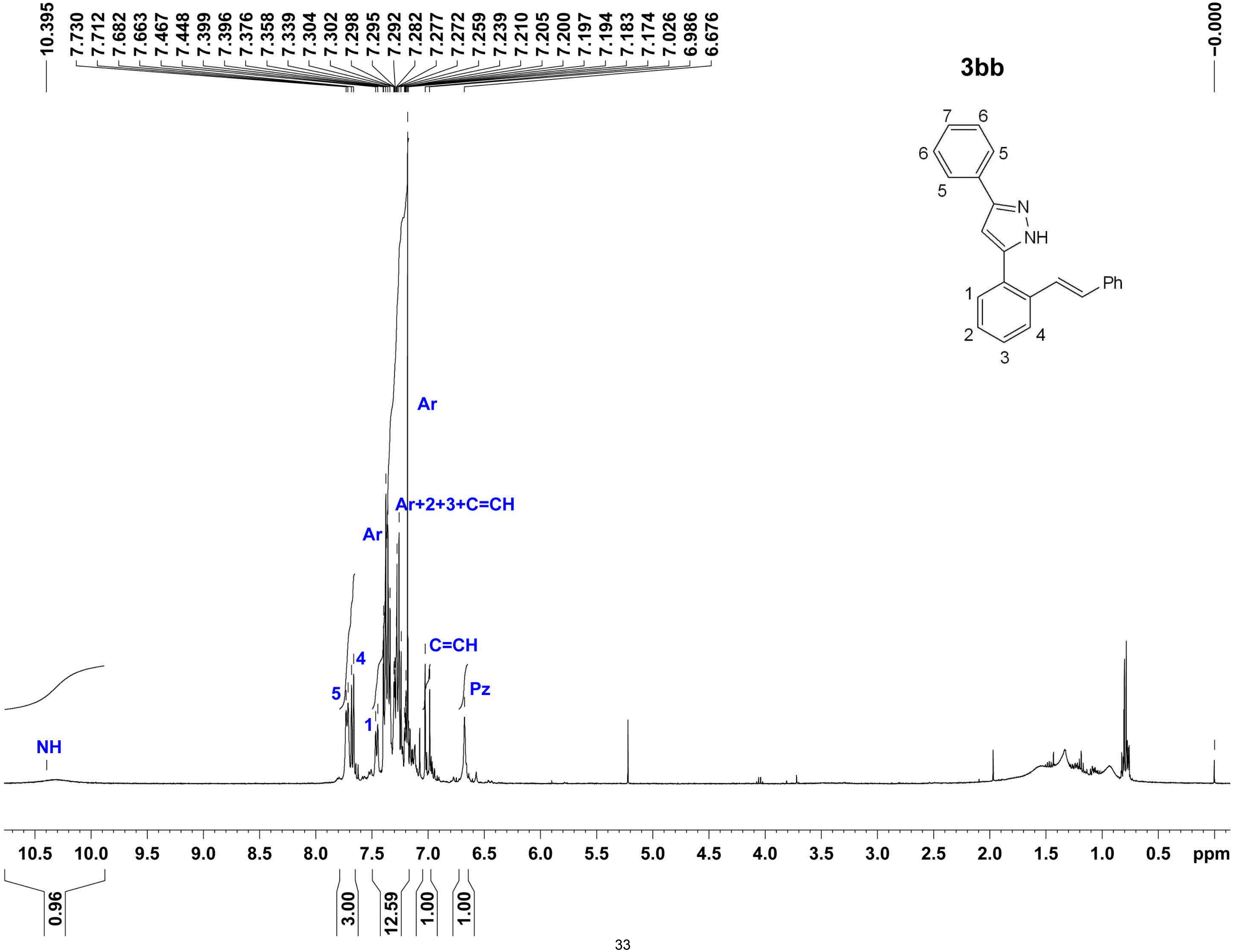
10.395
7.730
7.712
7.682
7.663
7.467
7.448
7.399
7.396
7.376
7.358
7.339
7.304
7.302
7.298
7.295
7.292
7.282
7.277
7.272
7.259
7.239
7.210
7.205
7.200
7.197
7.194
7.183
7.174
7.026
6.986
6.676
-0.000
Ar
Ar+2+3+C=CH
Ar
4
5
1
C=CH
Pz
NH
10.5 10.0 9.5 9.0 8.5 8.0 7.5 7.0 6.5 6.0 5.5 5.0 4.5 4.0 3.5 3.0 2.5 2.0 1.5 1.0 0.5 ppm
0.96
3.00
12.59
1.00
1.00
7
6
6
5
5
N
NH
Ph
1
2
3
4

3bb

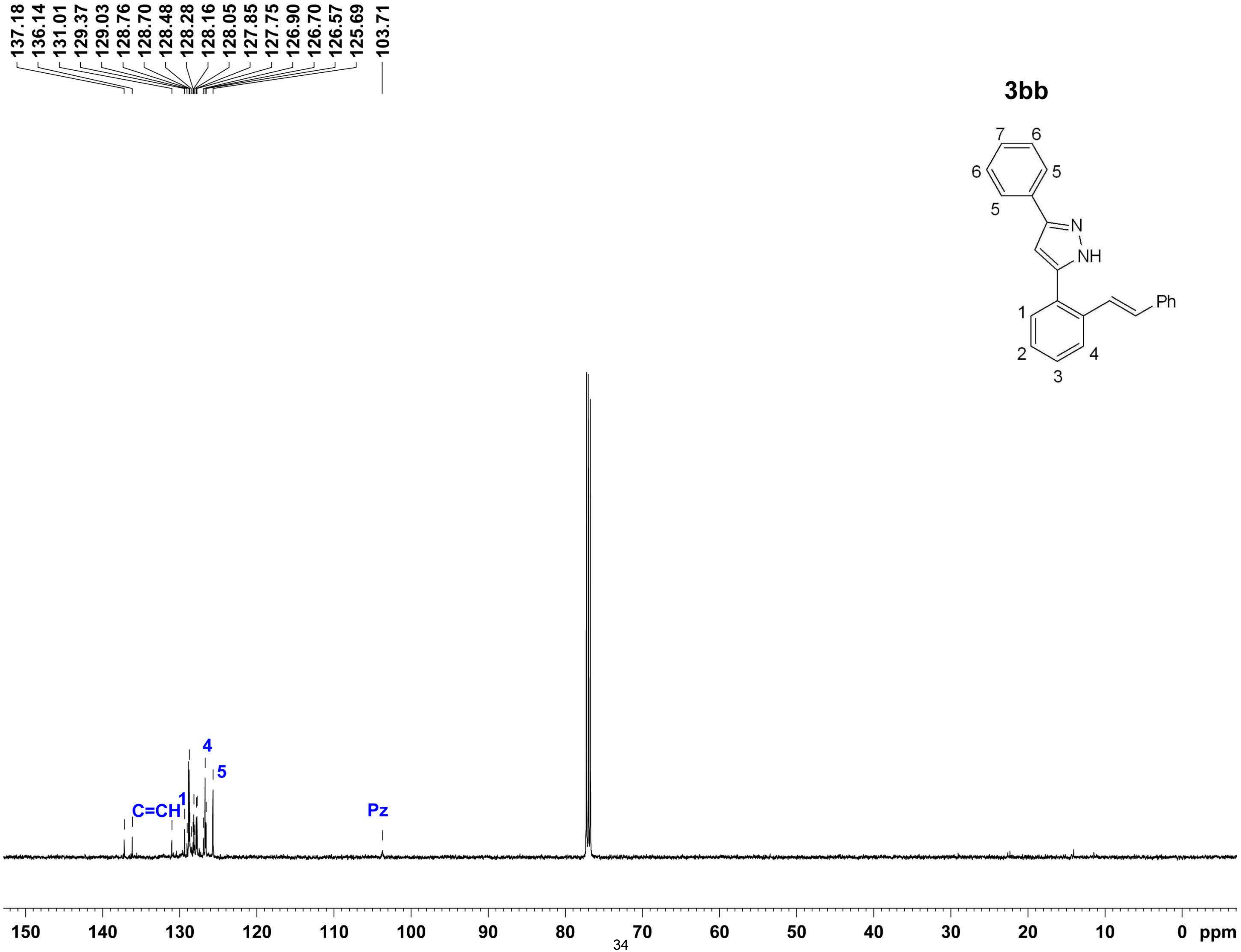

137.18
136.14
131.01
129.37
129.03
128.76
128.70
128.48
128.28
128.16
128.05
127.85
127.75
126.90
126.70
126.57
125.69
103.71
C=CH
1
4
5
Pz
150
140
130
120
110
100
90
80
70
60
50
40
30
20
10
0
ppm

3bb

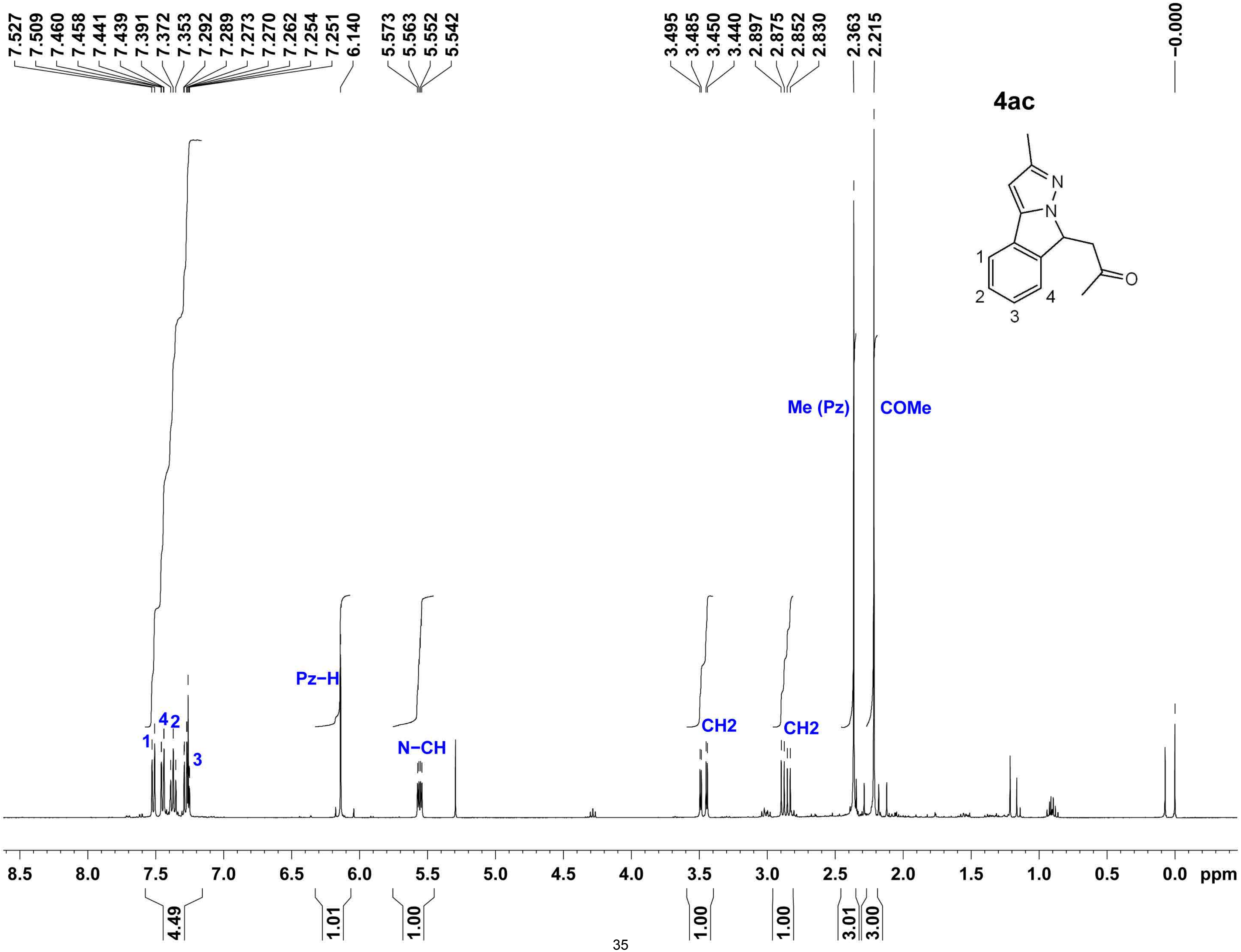
7.527
7.509
7.460
7.458
7.441
7.439
7.391
7.372
7.353
7.292
7.289
7.273
7.270
7.262
7.254
7.251
6.140
5.573
5.563
5.552
5.542
3.495
3.485
3.450
3.440
2.897
2.875
2.852
2.830
2.363
2.215
-0.000
4ac
N
N
O
1
2
3
4
Me (Pz)
COMe
Pz−H
N−CH
CH2
CH2
8.5
8.0
7.5
7.0
6.5
6.0
5.5
5.0
4.5
4.0
3.5
3.0
2.5
2.0
1.5
1.0
0.5
0.0
ppm
4.49
1.01
1.00
1.00
1.00
3.01
3.00

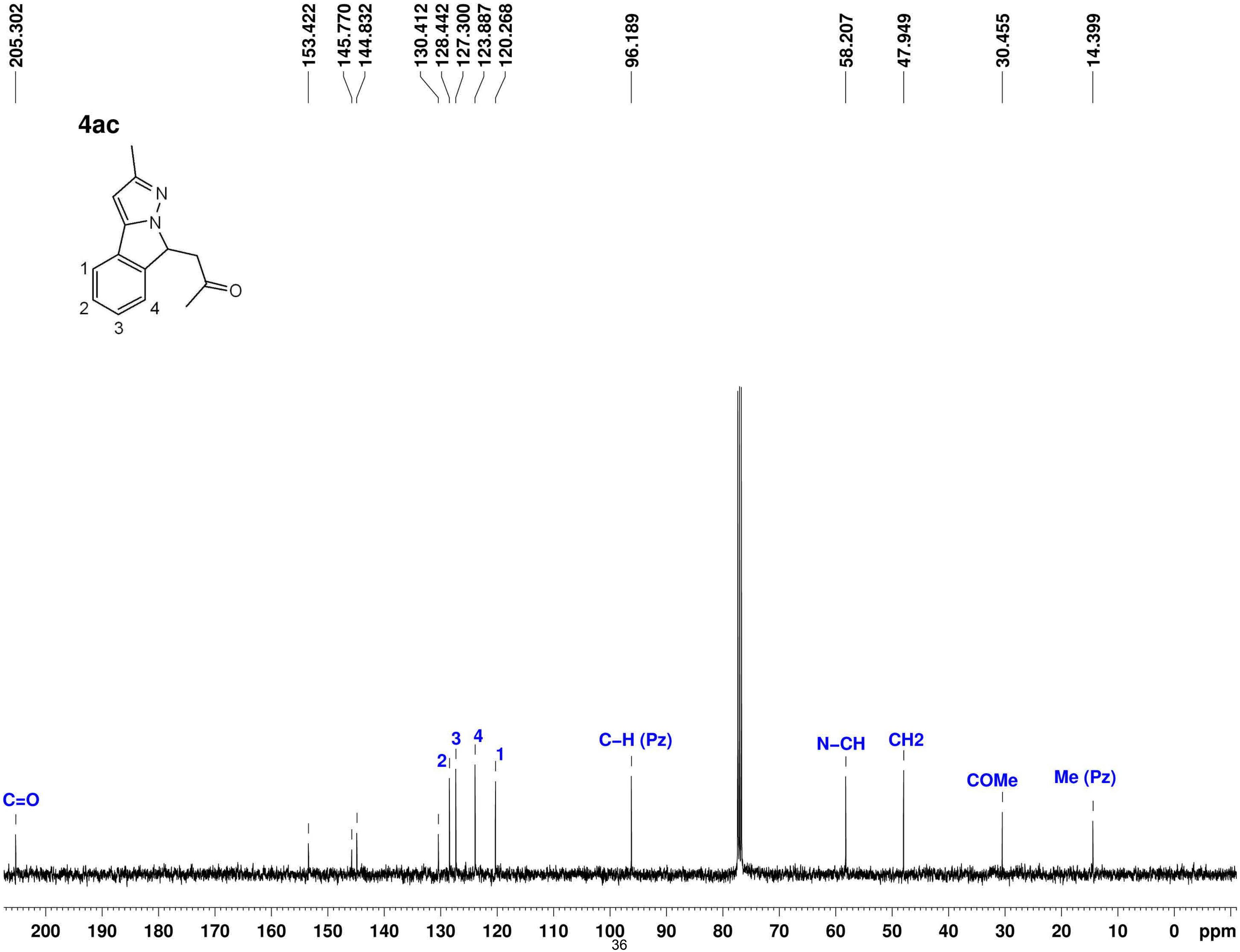
205.302
153.422
145.770
144.832
130.412
128.442
127.300
123.887
120.268
96.189
58.207
47.949
30.455
14.399
4ac
N
N
O
1
2
3
4
C=O
2
3
4
1
C–H (Pz)
N–CH
CH2
COMe
Me (Pz)
200 190 180 170 160 150 140 130 120 110 100 90 80 70 60 50 40 30 20 10 0 ppm

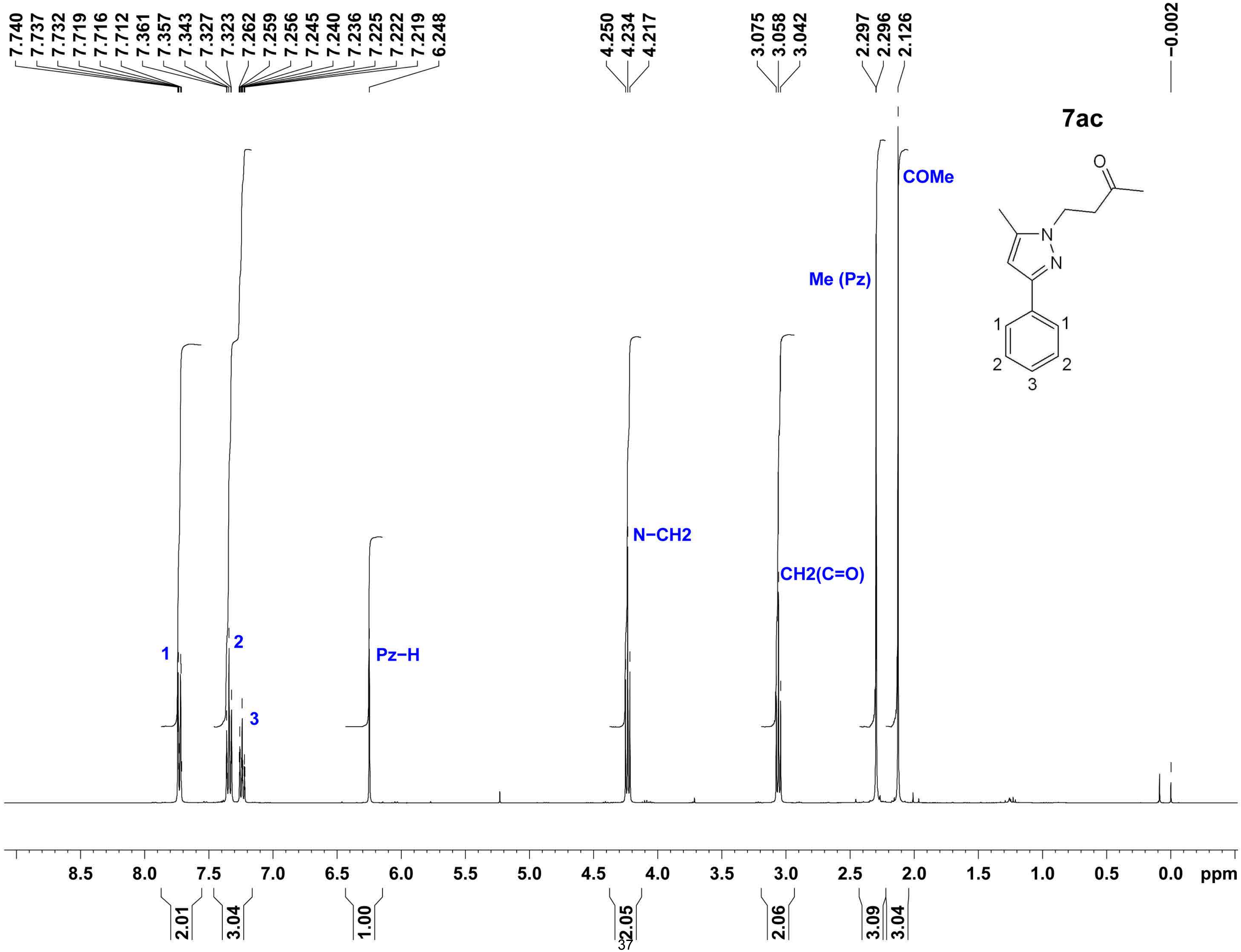
7.740
7.737
7.732
7.719
7.716
7.712
7.361
7.357
7.343
7.327
7.323
7.262
7.259
7.256
7.245
7.240
7.236
7.225
7.222
7.219
6.248
4.250
4.234
4.217
3.075
3.058
3.042
2.297
2.296
2.126
-0.002
7ac
COMe
Me (Pz)
N-CH2
CH2(C=O)
Pz-H
1
2
3
8.5
8.0
7.5
7.0
6.5
6.0
5.5
5.0
4.5
4.0
3.5
3.0
2.5
2.0
1.5
1.0
0.5
0.0
ppm
2.01
3.04
1.00
2.05
2.06
3.09
3.04

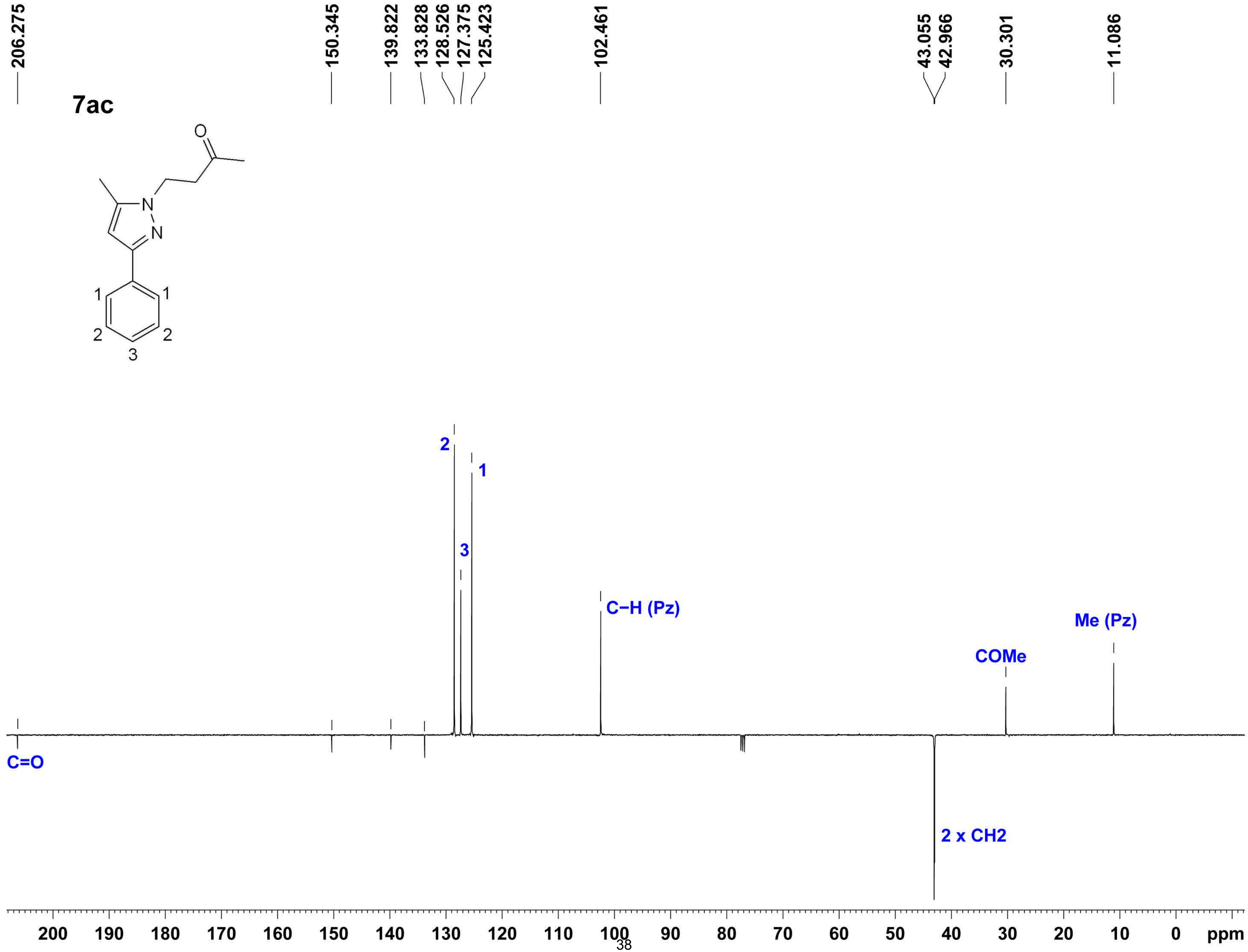
206.275
150.345
139.822
133.828
128.526
127.375
125.423
102.461
43.055
42.966
30.301
11.086
7ac
O
N
N
1
1
2
2
3
2
1
3
C−H (Pz)
Me (Pz)
COMe
C=O
2 x CH2
200
190
180
170
160
150
140
130
120
110
100
90
80
70
60
50
40
30
20
10
0
ppm

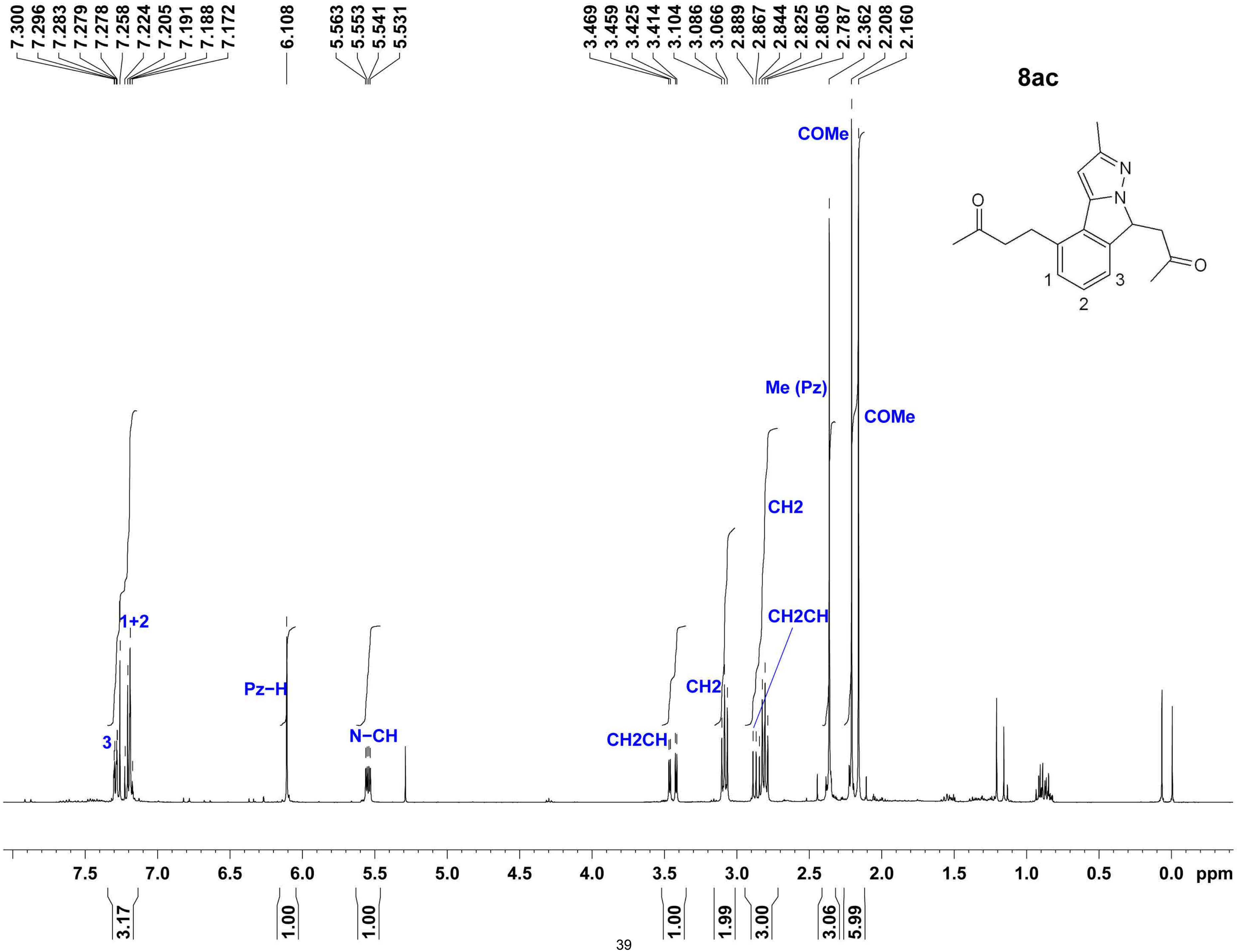
7.300
7.296
7.283
7.279
7.278
7.258
7.224
7.205
7.191
7.188
7.172
6.108
5.563
5.553
5.541
5.531
3.469
3.459
3.425
3.414
3.104
3.086
3.066
2.889
2.867
2.844
2.825
2.805
2.787
2.362
2.208
2.160
8ac
COMe
Me (Pz)
COMe
CH2
CH2CH
1+2
3
Pz−H
N−CH
CH2CH
CH2
7.5
7.0
6.5
6.0
5.5
5.0
4.5
4.0
3.5
3.0
2.5
2.0
1.5
1.0
0.5
0.0
ppm
3.17
1.00
1.00
1.00
1.99
3.00
3.06
5.99

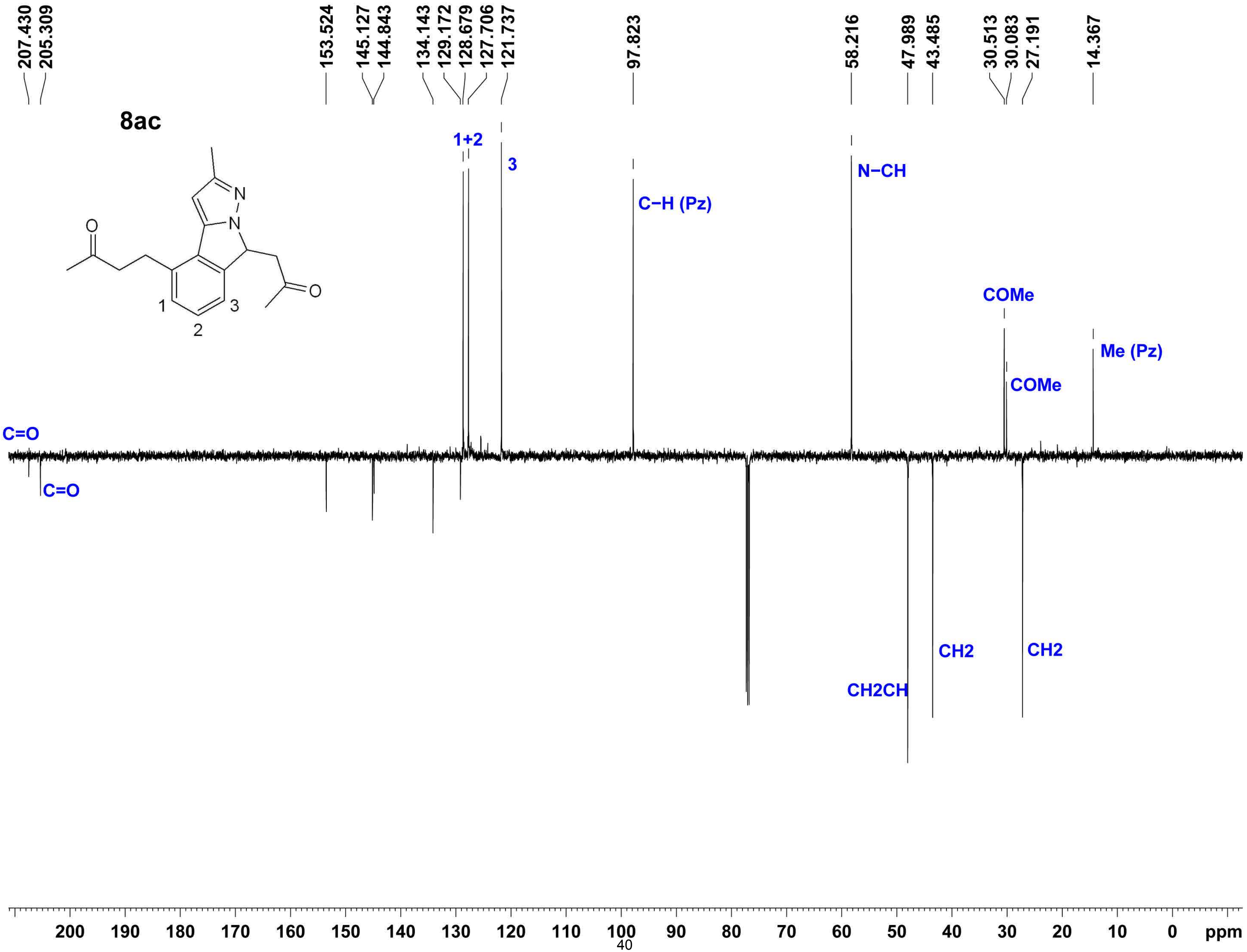
207.430
205.309
153.524
145.127
144.843
134.143
129.172
128.679
127.706
121.737
97.823
58.216
47.989
43.485
30.513
30.083
27.191
14.367
8ac
N
N
O
O
1
2
3
1+2
3
C−H (Pz)
N−CH
COMe
Me (Pz)
COMe
C=O
C=O
CH2
CH2
CH2CH
200
190
180
170
160
150
140
130
120
110
100
90
80
70
60
50
40
30
20
10
0
ppm

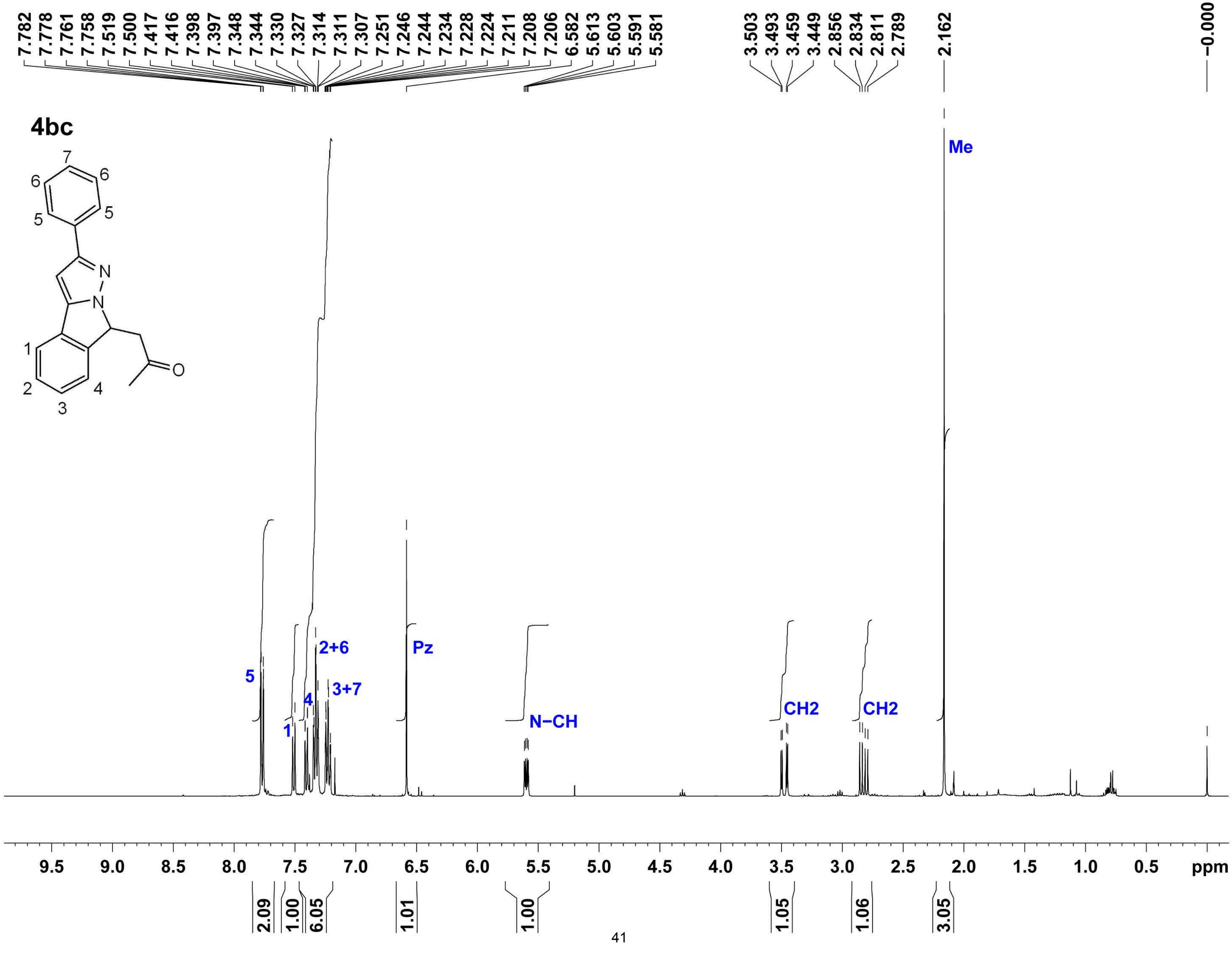
7.782
7.778
7.761
7.758
7.519
7.500
7.417
7.416
7.398
7.397
7.348
7.344
7.330
7.327
7.314
7.311
7.307
7.251
7.246
7.244
7.234
7.228
7.224
7.211
7.208
7.206
6.582
5.613
5.603
5.591
5.581
3.503
3.493
3.459
3.449
2.856
2.834
2.811
2.789
2.162
-0.000
4bc
Me
5
1
4
2+6
3+7
Pz
N−CH
CH2
CH2
ppm
2.09
1.00
6.05
1.01
1.00
1.05
1.06
3.05

4bc

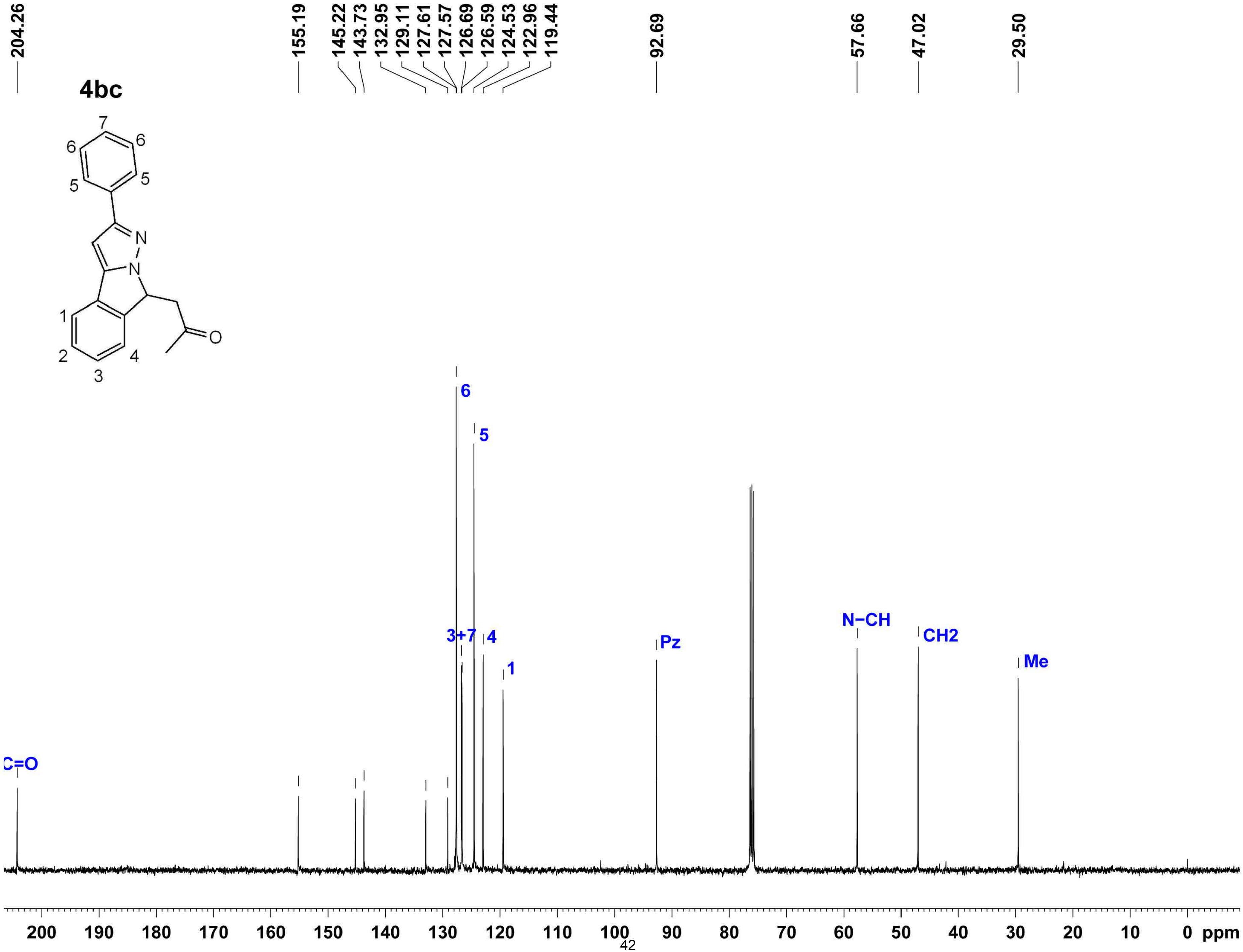
7
6
6
5
5
N
N
1
2
3
4
O
204.26
155.19
145.22
143.73
132.95
129.11
127.61
127.57
126.69
126.59
124.53
122.96
119.44
92.69
57.66
47.02
29.50
6
5
3+7
4
1
Pz
N−CH
CH2
Me
C=O
200
190
180
170
160
150
140
130
120
110
100
90
80
70
60
50
40
30
20
10
0
ppm

4. Computational details

DFT calculations were run with Gaussian 03 (Revision D.01) and Gaussian 09 (Revision A.02). Geometry optimisations were performed with Gaussian 03 and the BP86 functional and employed a smaller basis set, BS1, in which the Rh centre was described with the Stuttgart RECPs and associated basis sets and 6-31G** basis sets were used for all other atoms. The 'grid=ultrafine' option was used throughout. All stationary points were fully characterized via analytical frequency calculations as either minima (all positive eigenvalues) or transition states (one negative eigenvalue). IRC calculations and subsequent geometry optimizations were used to confirm the minima linked by each transition state. For both alkenes under consideration different possible orientations of the CO_2Me or Ph substituent were considered and the lowest energy structures are reported for each stationary point. Free energies (298.15 K, 1 atm) reported in the text, G_{DCE}, include corrections for dichloroethane solvent (ε = 10.125, PCM method, run with Gaussian 09), dispersion (Grimme's D3 parameter set[1]) and basis set effects using cc-pVTZ-PP on Rh and 6-311++G** on all other atoms (denoted BS2). Details of these different energy contributions for each reaction under consideration are given in Tables S1-3 (Supporting Information). In addition the 2,1 migratory insertion transition states (**TS(D1-E1)**$_{\mathbf{2,1}}$ and **TS(D2-E2)**$_{2,1}$) were reoptimised with a range of functionals and in all cases these were found to lie within 0.8 kcal/mol of one another (see Table S4, Supporting Information).

[1] S. Grimme, J. Antony, S. Ehrlich, H. Krieg, *J. Chem. Phys.* **2010**, *132*.

5. Breakdown of Energy Contributions

The following tables detail the evolution of the relative energies as the successive corrections to the initial SCF energy are included. Terms used are:

**ΔE_{BS1}**	SCF energy computed with the BP86 functional with BS1
**ΔH_{BS1}**	Enthalpy at 0 K with BS1
**ΔG_{BS1}**	Free energy at 298.15 K and 1 atm with BS1
**$\Delta G_{BS1/DCE}$**	Free energy corrected for dichloroethane solvent with BS1
**$\Delta G_{BS1/DCE+D3}$**	Free energy corrected for dichloroethane solvent and dispersion effects with BS1
**ΔE_{BS2}**	SCF energy corrected for basis effects with BS2
**ΔG_{final}**	Free energy corrected for basis set (BS2), dispersion effects and dichloroethane solvent

In each case the final data used in the main article is highlighted in bold.

a) Octyne

Table S1 – Computed relative energies (kcal/mol) for the reaction of 3-phenyl-5-methylpyrazole (**1a**) at [Rh(OAc)$_2$Cp*] and octyne. Data in bold are those used in the main text. All energies are quoted relative to **Int(A-B)** at 0.0 kcal/mol.

	ΔE_{BS1}	ΔH_{BS1}	ΔG_{BS1}	$\Delta G_{BS1/DCE}$	$\Delta G_{BS1/DCE+D3}$	ΔE_{BS2}	ΔG_{final}
**A**	-2.9	-2.5	-4.3	-4.2	+7.4	-2.1	**+8.3**
**TS(A-B)1**	+11.7	+11.4	+10.9	+11.0	+14.2	+12.3	**+14.9**
**Int(A-B)**	0.0	0.0	0.0	0.0	0.0	0.0	**0.0**
**TS(A-B)2**	+10.0	+8.2	+9.2	+11.0	+12.4	+10.9	**+13.3**
**B·HOAc**	-0.1	-0.5	-0.0	+0.1	+3.6	+0.3	**+4.0**
**B**	+16.4	+15.3	+3.4	+1.0	+11.9	+13.0	**+8.5**
**TS(B-C)1**	+26.4	+24.5	+12.2	+9.3	+20.6	+22.4	**+16.5**
**Int(B-C)**	+25.8	+24.1	+11.4	+9.0	+19.0	+21.7	**+14.9**
**TS(B-C)2**	+28.8	+24.5	+13.2	+11.9	+22.2	+26.1	**+19.5**
**C1**	+21.2	+19.8	+8.2	+7.6	+18.5	+18.5	**+15.7**
**TS(C1-C2)**	+22.2	+20.8	+9.8	+9.6	+20.3	+19.4	**+17.4**
**C2**	+11.5	+10.7	-0.5	-0.9	+9.2	+7.8	**+5.5**
**D**	+18.3	+17.2	+7.9	+6.9	+8.9	+14.1	**+4.7**
**TS(D-E)**	+30.9	+29.4	+21.3	+20.8	+23.1	+27.2	**+19.4**
**E**	+13.0	+12.9	+3.6	+2.0	+6.3	+10.0	**+3.3**
**TS(E-F)**	+21.2	+20.3	+12.6	+12.5	+16.6	+18.4	**+13.9**
**F**	-8.7	-7.6	-14.8	-14.6	-10.9	-9.6	**-11.8**

b) Methyl acrylate

Table S2a – Computed relative energies (kcal/mol) for the reaction of 3-phenyl-5-methylpyrazole (**1a**) at [Rh(OAc)$_2$Cp*] and methyl acrylate (**a**) via 2,1-insertion. Data in bold are those used in the main text. All energies are quoted relative to **Int(A-B)** at 0.0 kcal/mol; see Table S1 for data on **A** to **C2**.

	ΔE_{BS1}	ΔH_{BS1}	ΔG_{BS1}	$\Delta G_{BS1/DCE}$	$\Delta G_{BS1/DCE+D3}$	ΔE_{BS2}	ΔG_{final}
**D1$_{2,1}$**	+14.3	+14.1	+4.1	+3.5	+7.6	+10.6	**+4.0**
**TS(D1-E1)$_{2,1}$**	+27.9	+27.7	+19.0	+18.6	+23.3	+24.3	**+19.6**
**E1$_{2,1}$**	+18.5	+18.7	+8.5	+6.4	+13.1	+14.2	**+8.8**
**TS(E1-F1)$_{2,1}$**	+64.1	+63.0	+54.6	+54.2	+60.1	+62.4	**+58.4**
**F1$_{2,1}$**	+29.7	+30.1	+20.3	+20.6	+26.8	+27.1	**+24.1**
**D2$_{2,1}$**	+17.7	+17.2	+7.0	+3.9	+7.3	+14.5	**+4.1**
**TS(D2-E2)$_{2,1}$**	+30.5	+29.8	+20.5	+18.6	+23.7	+26.6	**+19.7**
**E2$_{2,1}$**	+15.1	+15.3	+4.9	+3.1	+8.5	+11.8	**+5.2**
**TS(E2-F2)$_{2,1}$**	+63.1	+61.8	+51.9	+51.2	+63.1	+62.3	**+62.3**
**F2$_{2,1}$**	+30.1	+30.6	+20.3	+19.7	+27.0	+26.9	**+23.8**
**TS(E1-E1′)$_{2,1}$**	+20.1	+20.3	+11.2	+9.5	+15.9	+16.2	**+12.0**
**E1′$_{2,1}$**	+18.3	+18.8	+8.8	+8.1	+12.3	+15.5	**+9.5**
**TS(E1′-E$_{trans}$)**	+20.9	+21.2	+12.4	+10.5	+19.3	+17.1	**+15.4**
**TS(E2-E2′)$_{2,1}$**	+19.3	+19.6	+11.0	+9.2	+17.8	+15.2	**+13.7**
**E2′$_{2,1}$**	+18.4	+19.1	+9.6	+8.5	+16.9	+14.5	**+13.1**
**TS(E2′-E$_{trans}$)$_{2,1}$**	+19.6	+19.7	+10.7	+9.5	+18.4	+15.4	**+14.1**
**E$_{trans}$ / E$_{trans}$′**	+11.1	+11.2	+2.4	+1.7	+10.3	+7.1	**+6.3**
**TS(E-G)$_{trans}$ / TS(E-G)$_{trans}$′**	+13.5	+11.9	+3.3	+3.0	+11.3	+9.9	**+7.6**
**G$_{trans}$ / G$_{trans}$′**	+9.4	+8.7	-0.0	-0.3	+7.8	+6.5	**+4.9**
**TS(E1-E1″)$_{2,1}$**	+21.1	+21.4	+12.2	+9.4	+17.8	+17.6	**+14.2**
**TS(E2-E1″)$_{2,1}$**	+26.6	+26.5	+16.7	+13.7	+23.7	+22.5	**+19.6**
**E1″$_{2,1}$**	+21.0	+21.4	+11.3	+8.6	+16.9	+17.4	**+13.3**
**TS(E1″-E$_{cis}$)**	+25.5	+25.3	+16.2	+13.0	+22.3	+21.8	**+18.6**
**TS(E2-E2″)$_{2,1}$**	+15.2	+15.3	+5.8	+3.8	+15.1	+12.0	**+11.8**
**E2″$_{2,1}$**	+14.1	+14.5	+4.6	+3.2	+11.3	+10.3	**+7.6**
**TS(E2″-E$_{cis}$)**	+25.3	+25.0	+15.6	+12.3	+21.4	+21.4	**+17.5**
**E$_{cis}$ / E$_{cis}$′**	+19.7	+19.2	+9.9	+7.0	+15.8	+15.3	**+11.5**
**TS(E-G)$_{cis}$ / TS(E-G)$_{cis}$′**	+21.2	+19.3	+10.2	+8.9	+16.1	+17.1	**+12.0**
**G$_{cis}$ / G$_{cis}$′**	+15.6	+14.6	+5.2	+4.2	+10.5	+12.5	**+7.4**

Table S2b – Computed relative energies (kcal/mol) for the reaction of 3-phenyl-5-methylpyrazole (**1a**) at [Rh(OAc)$_2$Cp*] and methyl acrylate (**a**) via 1,2-insertion. Data in bold are those used in the main text. All energies are quoted relative to **Int(A-B)** at 0.0 kcal/mol; see Table S1 for data on **A** to **C2**.

	**ΔE$_{BS1}$**	**ΔH$_{BS1}$**	**ΔG$_{BS1}$**	**ΔG$_{BS1/DCE}$**	**ΔG$_{BS1/DCE+D3}$**	**ΔE$_{BS2}$**	**ΔG$_{final}$**
**D1$_{1,2}$**	+12.7	+12.9	+3.6	+3.3	+7.1	+9.3	**+3.6**
**TS(D1-E1)$_{1,2}$**	+34.2	+33.6	+24.5	+24.5	+30.1	+30.6	**+26.5**
**E1$_{1,2}$**	+22.2	+22.1	+10.6	+8.2	+19.8	+16.9	**+14.6**
**TS(E1-F1)$_{1,2}$**	+73.5	+72.1	+62.0	+62.3	+70.1	+71.3	**+67.9**
**F1$_{1,2}$**	+28.9	+29.4	+19.5	+19.7	+25.4	+26.6	**+23.2**
**TS(E1-E$_{gem}$)$_{1,2}$**	+27.9	+27.6	+17.9	+16.3	+27.1	+23.9	**+23.2**
**D2$_{1,2}$**	+17.4	+17.0	+7.0	+5.1	+8.0	+13.3	**+3.9**
**TS(D2-E2)$_{1,2}$**	+37.3	+36.4	+27.2	+25.6	+31.1	+33.0	**+26.7**
**E2$_{1,2}$**	+27.5	+27.8	+16.7	+13.8	+25.5	+22.5	**+20.5**
**TS(E2-F2)$_{1,2}$**	+72.0	+70.4	+60.3	+59.5	+67.2	+69.3	**+64.5**
**F2$_{1,2}$**	+28.1	+28.6	+18.0	+17.6	+24.8	+25.1	**+21.8**
**TS(E2-E$_{gem}$)$_{1,2}$**	+29.3	+29.3	+20.2	+18.2	+29.2	+25.0	**+24.9**
**E$_{gem}$ / E$_{gem}$′**	+24.0	+23.2	+13.6	+12.7	+20.5	+20.1	**+16.7**
**TS(E-G)$_{gem}$ / TS(E-G)$_{gem}$′**	+24.6	+22.5	+13.3	+12.7	+19.7	+21.1	**+16.6**
**G$_{gem}$ / G$_{gem}$′**	+15.5	+14.7	+6.3	+5.6	+11.6	+13.3	**+9.5**

Table S2d – Computed relative energies (kcal/mol) for the overall reaction of 3-phenyl-5-methylpyrazole (**1a**) at [Rh(OAc)$_2$Cp*] and methyl acrylate (**a**) to give organic compounds. Data in bold are those used in the main text. All energies are quoted relative to **3aa$_{trans}$** at 0.0 kcal/mol.

	**ΔE$_{BS1}$**	**ΔH$_{BS1}$**	**ΔG$_{BS1}$**	**ΔG$_{BS1/DCE}$**	**ΔG$_{BS1/DCE+D3}$**	**ΔE$_{BS2}$**	**ΔG$_{final}$**
**3aa$_{trans}$**	0.0	0.0	0.0	0.0	0.0	0.0	**0.0**
**3aa$_{cis}$**	+6.5	+6.5	+6.4	+6.2	+4.9	+6.4	**+4.8**
**4aa**	-8.7	-7.7	-7.2	-4.7	-6.6	-6.8	**-4.7**

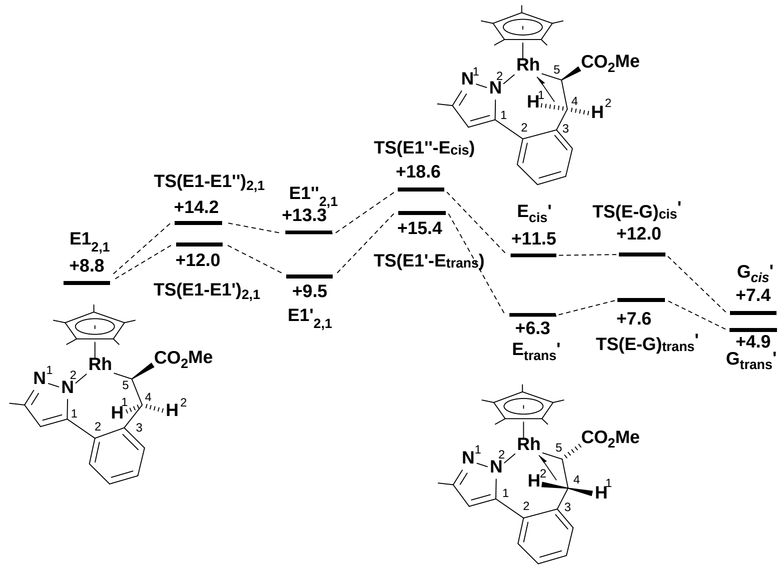

Figure S2. Computed energy profiles (G_{DCE}, kcal/mol) for β-H transfer from **E1$_{2,1}$** formed with methyl acrylate. Energies (G_{DCE}) are quoted relative to **Int(A-B)** and free methyl acrylate set to 0.0 kcal/mol.

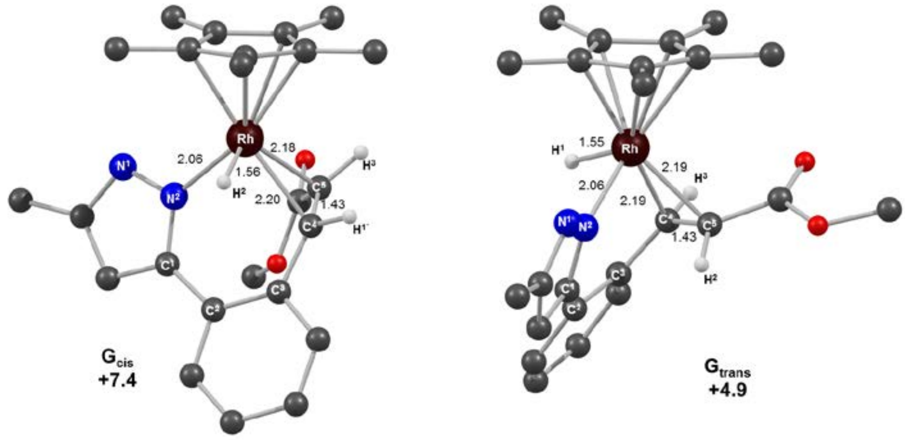

Figure S3. Computed structures of G_{cis} and G_{trans} with relative energies in kcal/mol and selected distances in Å. H atoms except those on C^4 and C^5 are omitted for clarity.

c) Styrene

Table S3a – Computed relative energies (kcal/mol) for the reaction of 3-phenyl-5-methylpyrazole (**1a**) at [Rh(OAc)$_2$Cp*] and styrene (**b**) via 2,1-insertion. Data in bold are those used in the main text. All energies are quoted relative to **Int(A-B)** at 0.0 kcal/mol; see Table S1 for data on **A** to **C2**.

	ΔE_{BS1}	ΔH_{BS1}	ΔG_{BS1}	$\Delta G_{BS1/DCE}$	$\Delta G_{BS1/DCE+D3}$	ΔE_{BS2}	ΔG_{final}
2,1-insertion							
**D2$_{2,1}$**	+20.6	+20.0	+9.8	+7.6	+9.2	+15.6	**+4.1**
**TS(D2-E2)$_{2,1}$**	+36.2	+35.6	+26.9	+25.5	+28.4	+31.4	**+23.6**
**E2$_{2,1}$**	+13.2	+13.8	+5.4	+4.7	+5.3	+9.4	**+1.6**
**TS(E2-F2)$_{2,1}$**	+72.6	+70.7	+60.9	+60.2	+65.8	+69.1	**+62.4**
**F2$_{2,1}$**	+32.2	+32.7	+22.8	+22.6	+27.8	+28.4	**+24.0**
**TS(E2-E2′)$_{2,1}$**	+32.6	+32.6	+22.1	+20.0	+27.3	+28.0	**+22.7**
**E2′$_{2,1}$**	+26.6	+26.9	+17.2	+16.4	+22.8	+21.5	**+17.7**
**TS(E2′-E$_{trans}$)**	+28.1	+28.2	+19.9	+18.8	+25.9	+23.0	**+20.8**
**E$_{trans}$ / E$_{trans}$′**	+18.6	+18.6	+10.2	+9.1	+15.9	+13.5	**+10.9**
**TS(E-G)$_{trans}$ / TS(E-G)$_{trans}$′**	+20.4	+18.7	+10.2	+9.3	+15.9	+15.7	**+11.1**
**G$_{trans}$ / G$_{trans}$′**	+15.4	+14.7	+6.0	+5.2	+11.3	+11.4	**+7.4**
**D1$_{2,1}$**	+18.6	+18.4	+9.2	+8.8	+10.1	+13.9	**+5.4**
**TS(D1-E1)$_{2,1}$**	+34.5	+34.1	+25.8	+25.2	+28.0	+29.8	**+23.2**
**E1$_{2,1}$**	+24.5	+25.0	+15.6	+14.4	+18.4	+19.8	**+13.7**
**TS(E1-F1)$_{2,1}$**	+73.9	+72.8	+64.2	+64.7	+66.7	+71.2	**+64.1**
**F1$_{2,1}$**	+33.0	+33.4	+24.0	+23.7	+26.8	+29.7	**+23.4**
**TS(E1-E2″)$_{2,1}$**	+27.0	+27.4	+19.2	+17.9	+22.5	+22.4	**+17.8**
**E2″$_{2,1}$**	+26.2	+26.5	+17.1	+16.5	+19.2	+22.2	**+15.2**
**TS(E2″-E$_{trans}$)**	+28.8	+29.2	+20.5	+19.0	+25.9	+23.9	**+21.0**
**TS(E1-E$_{cis}$) / TS(E1-E$_{cis}$)′**	+30.8	+30.6	+21.6	+19.7	+26.4	+26.2	**+21.7**
**E$_{cis}$ / E$_{cis}$′**	+25.7	+25.0	+15.8	+14.1	+19.4	+20.1	**+13.8**
**TS(E-G)$_{cis}$ / TS(E-G)$_{cis}$′**	+26.7	+24.6	+15.8	+14.2	+19.2	+21.2	**+13.7**
**G$_{cis}$ / G$_{cis}$′**	+20.1	+18.9	+9.7	+7.9	+12.1	+15.4	**+7.3**

Table S3b – Computed relative energies (kcal/mol) for the reaction of 3-phenyl-5-methylpyrazole (**1a**) at [Rh(OAc)$_2$Cp*] and styrene (**b**) via 1,2-insertion. Data in bold are those used in the main text. All energies are quoted relative to **Int(A-B)** at 0.0 kcal/mol; see Table S1 for data on **A** to **C2**.

	**ΔE$_{BS1}$**	**ΔH$_{BS1}$**	**ΔG$_{BS1}$**	**ΔG$_{BS1/DCE}$**	**ΔG$_{BS1/DCE+D3}$**	**ΔE$_{BS2}$**	**ΔG$_{final}$**
1,2-insertion							
**D1$_{1,2}$**	+19.3	+19.1	+9.2	+8.2	+10.0	+14.4	**+5.2**
**TS(D1-E1)$_{1,2}$**	+39.6	+38.7	+29.0	+28.2	+32.1	+34.7	**+27.2**
**E1$_{1,2}$**	+26.2	+26.1	+15.1	+13.2	+23.5	+20.4	**+17.7**
**TS(E1-F1)$_{1,2}$**	+76.0	+74.7	+64.9	+64.9	+70.5	+73.0	**+67.5**
**F1$_{1,2}$**	+32.7	+33.4	+23.5	+23.7	+27.1	+29.3	**+23.7**
**TS(E1-E$_{gem}$)$_{1,2}$**	+31.3	+31.2	+21.7	+19.8	+29.5	+26.2	**+24.5**
**D2$_{1,2}$**	+21.7	+21.0	+10.3	+8.1	+10.0	+16.6	**+4.9**
**TS(D2-E2)$_{1,2}$**	+39.5	+38.5	+29.0	+27.9	+31.4	+34.4	**+26.3**
**E2$_{1,2}$**	+30.5	+30.8	+19.8	+17.1	+27.2	+25.3	**+22.0**
**TS(E2-F2)$_{1,2}$**	+76.0	+74.5	+64.5	+64.0	+70.2	+72.4	**+66.6**
**F2$_{1,2}$**	+32.1	+32.7	+22.1	+21.9	+27.8	+28.2	**+23.9**
**TS(E2-E$_{gem}$)$_{1,2}$**	+32.9	+33.0	+24.2	+22.2	+31.7	+28.1	**+26.9**
**E$_{gem}$ / E$_{gem}$′**	+26.4	+25.8	+16.3	+15.4	+21.2	+21.4	**+16.2**
**TS(E-G)$_{gem}$ / TS(E-G)$_{gem}$′**	+27.3	+25.2	+16.2	+15.4	+20.2	+22.6	**+15.5**
**G$_{gem}$ / G$_{gem}$′**	+19.6	+18.5	+9.5	+8.9	+12.1	+15.8	**+8.2**

Table S3c – Computed relative energies (kcal/mol) for the overall reaction of 3-phenyl-5-methylpyrazole (**1a**) at [Rh(OAc)$_2$Cp*] and styrene (**b**) to give organic compounds. Data in bold are those used in the main text. All energies are quoted relative to **3ab$_{trans}$** at 0.0 kcal/mol.

	**ΔE$_{BS1}$**	**ΔH$_{BS1}$**	**ΔG$_{BS1}$**	**ΔG$_{BS1/DCE}$**	**ΔG$_{BS1/DCE+D3}$**	**ΔE$_{BS2}$**	**ΔG$_{final}$**
**3ab$_{trans}$**	0.0	0.0	0.0	0.0	0.0	0.0	**0.0**
**3ab$_{cis}$**	+4.9	+5.0	+5.1	+5.5	+3.7	+4.5	**+3.3**
**4ab**	-10.0	-8.8	-6.9	-4.6	-7.3	-8.3	**-5.7**

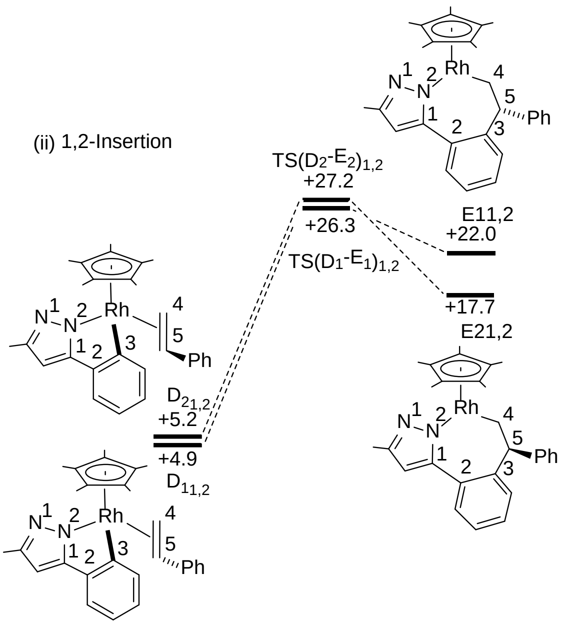

Figure S4. Computed energy profiles (G_{DCE}, kcal/mol) for the migratory insertion of styrene from adducts **D** via 1,2-insertion. Energies (G_{DCE}) are quoted relative to **Int(A-B)** and free styrene set to 0.0 kcal/mol.

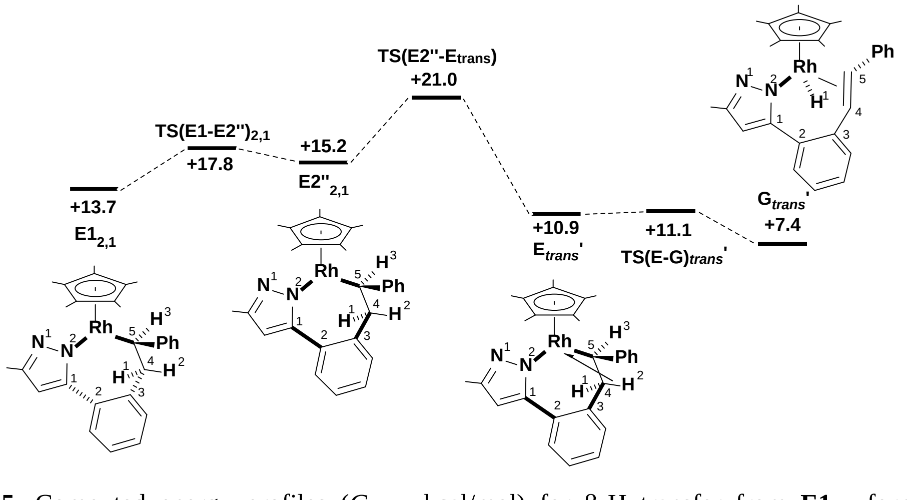

Figure S5. Computed energy profiles (G_{DCE}, kcal/mol) for β-H transfer from $\mathbf{E1_{2,1}}$ formed with styrene. Energies (G_{DCE}) are quoted relative to **Int(A-B)** and free styrene set to 0.0 kcal/mol.

6. Functional Testing

The preference for alkene 2,1-insertion to give either **E1** or **E2** is very close in free energy. Therefore, we assessed these barriers with a range of density functionals to determine if this was dependent on the functional employed. The barrier was computed for both **TS(D1-E1)$_{2,1}$** and **TS(D2-E2)$_{2,1}$**, and the relative free energy barriers from **D1** and **D2** respectively are given in the table below. The free energies include corrections for solvent (DCE) and basis set effects. An additional correction for dispersion effects is included for all functionals with the exception of M06, M06L, B97D and ωB97XD where a treatment of dispersion is included in the original functional, and B3P86 which does not have dispersion corrected parameters available.

Table S4 – Computed **ΔG_{final}** relative energy barriers (kcal/mol) for the 2,1-insertion of methyl acrylate or styrene with 3-phenyl-5-methylpyrazole (**1a**) and [Rh(OAc)$_2$Cp*] for a range of density functionals. The final column gives the difference between these insertion transition states (**$\Delta\Delta G_{2,1}$** = {**TS(D2-E2)$_{2,1}$**} - {**TS(D1-E1)$_{2,1}$**}).

**Substrate**	**Functional**	**ΔG_{final}**		**$\Delta\Delta G_{2,1}$**
		**TS(D2-E2)$_{2,1}$**	**TS(D1-E1)$_{2,1}$**	
Methyl Acrylate				
	BP86	+19.8	+19.7	*0.14*
	B3P86	+17.8	+17.9	*-0.07*
	BLYP	+29.1	+28.8	*0.28*
	B3LYP	+30.1	+30.2	*-0.10*
	PBEPBE	+17.9	+18.2	*-0.36*
	PBE1PBE	+19.5	+19.5	*0.02*
	B3PW91	+20.9	+21.0	*-0.03*
	M06	+19.9	+19.2	*0.72*
	M06L	+26.3	+26.8	*-0.42*
	B97D	+21.2	+21.5	*-0.33*
	ωB97XD	+25.2	+25.0	*0.24*
Styrene				
	BP86	+23.7	+23.3	*0.38*
	B3P86	+24.0	+23.8	*0.18*
	BLYP	+33.2	+33.1	*0.11*
	B3LYP	+35.0	+34.9	*0.12*
	PBEPBE	+22.2	+22.3	*-0.12*
	PBE1PBE	+24.4	+24.3	*0.10*
	B3PW91	+25.4	+25.1	*0.30*
	M06	+24.5	+24.2	*0.28*
	M06L	+31.9	+31.4	*0.48*
	B97D	+24.6	+24.5	*0.04*
	ωB97XD	+28.4	+27.7	*0.76*

7. Cartesian Coordinates and calculated energies of all stationary points.

Cartesian coordinates are shown in Å and energies in atomic units

```
AcOH
SCF (BP86) Energy = -229.088709027
Enthalpy 0K = -229.028638
Energy 298K = -229.023982
Free Energy 298K = -229.056014
Lowest Frequency = 61.5402 cm-1
SCF (DCE) Energy = -229.094077515
SCF (BP86-D3) Energy = -229.092152147
SCF (BS2) Energy = -229.166192709

O  -0.77354 -1.06292  0.00001
H  -1.72202 -0.80692 -0.00000
C  -0.09249  0.12627  0.00002
O  -0.65939  1.20855 -0.00001
C   1.40383 -0.10156 -0.00001
H   1.69808 -0.68620  0.88710
H   1.69804 -0.68627 -0.88708
H   1.92133  0.86608 -0.00005

PrCCPr
SCF (BP86) Energy = -313.222624271
Enthalpy 0K = -313.029163
Energy 298K = -313.018261
Free Energy 298K = -313.067816
Lowest Frequency = 7.1862 cm-1
SCF (DCE) Energy = -313.225015762
SCF (BP86-D3) Energy = -313.237035581
SCF (BS2) Energy = -313.300251185

C  -0.58949 -0.49268  0.16301
C   0.58949 -0.49275 -0.16303
C   2.00991 -0.47810 -0.52284
C  -2.00991 -0.47824  0.52278
H  -2.12650 -0.12045  1.56571
H  -2.40701 -1.51344  0.51693
H   2.12641 -0.12010 -1.56571
H   2.40707 -1.51329 -0.51727
C  -2.87737  0.40142 -0.41309
H  -2.76207  0.03778 -1.45011
H  -2.47992  1.43235 -0.40066
C  -4.35882  0.39833 -0.01068
H  -4.95931  1.02876 -0.68779
H  -4.78124 -0.62183 -0.04158
H  -4.49721  0.78375  1.01521
C   2.87737  0.40138  0.41313
H   2.47996  1.43234  0.40091
H   2.76209  0.03759  1.45011
C   4.35883  0.39830  0.01072
H   4.49720  0.78392 -1.01510
H   4.95934  1.02858  0.68795
H   4.78122 -0.62189  0.04141

-------------------------------------

A
SCF (BP86) Energy = -1454.43511870
Enthalpy 0K = -1453.942446
Energy 298K = -1453.905823
Free Energy 298K = -1454.015356
Lowest Frequency = 10.6160 cm-1
SCF (DCE) Energy = -1454.45056500
SCF (BP86-D3) Energy = -1454.51664452
SCF (BS2) Energy = -1454.45894535

Rh -2.05422 -0.30926  0.18699
O  -2.69998  1.67505  0.11660
C  -1.92128  2.60425 -0.36937
C  -2.52808  4.00775 -0.30634
C  -2.51816 -0.82196 -1.87350
C  -1.08899 -0.96840 -1.65380
C  -0.90508 -1.98147 -0.61042
C  -2.20447 -2.44268 -0.18826
C  -3.21509 -1.70598 -0.96257
O  -0.77376  2.44165 -0.84747
C  -4.69964 -1.89929 -0.85851
C  -3.14975  0.10103 -2.87340
C   0.00088 -0.31465 -2.44870
C   0.42809 -2.43018 -0.09321
C  -2.50143 -3.50400  0.83068
C   4.84466 -0.27226  0.01022
C   4.52845 -1.58063  0.44451
C   6.18311  0.00757 -0.34920
C   5.51504 -2.57294  0.51035
C   7.17072 -0.98463 -0.27607
C   6.84254 -2.28138  0.15229
C   3.98225  2.18683 -0.09228
C   2.69347  2.71952 -0.15330
N   2.49043  0.45206 -0.10580
N   1.84906  1.64491 -0.15316
C   3.80953  0.77397 -0.06228
O  -2.91346 -0.18344  2.19118
C  -1.81081  0.30626  2.63640
C  -1.70056  0.81464  4.05178
O  -0.81147  0.38094  1.82991
H  -3.60667  3.98128 -0.09570
H  -2.02366  4.57530  0.49458
H  -2.33609  4.53568 -1.25364
H  -5.24672 -1.01075 -1.20929
H  -5.02271 -2.76129 -1.47239
H  -5.00441 -2.09484  0.18190
H  -2.52763  0.99538 -3.02879
H  -3.25927 -0.41147 -3.84727
H  -4.14864  0.43004 -2.54817
H   0.95738 -0.32544 -1.90192
H   0.14420 -0.85287 -3.40560
H  -0.24700  0.73480 -2.66838
H   1.12638 -1.57726 -0.00963
H   0.33875 -2.91077  0.89333
H   0.87247 -3.16474 -0.79109
H  -2.72369 -4.46669  0.33319
H  -1.64633 -3.66408  1.50503
H  -3.37312 -3.23408  1.44797
H   3.49915 -1.79870  0.74363
H   6.44553  1.00931 -0.70562
H   5.24904 -3.57928  0.85289
H   8.20089 -0.74512 -0.56184
H   7.61388 -3.05680  0.20829
H   4.91372  2.74837 -0.04493
H   0.81954  1.69085 -0.25820
H  -2.02562  1.86885  4.08035
H  -2.35746  0.23669  4.71885
H  -0.65786  0.76387  4.39798
C   2.17322  4.12623 -0.19314
H   1.12571  4.13086 -0.53637
H   2.20945  4.60212  0.80372
H   2.77179  4.74960 -0.87774

TS(A-B)1
SCF (BP86) Energy = -1454.41191531
Enthalpy 0K = -1453.920339
```

Energy 298K = -1453.884230
Free Energy 298K = -1453.991116
Lowest Frequency = -89.8451 cm^{-1}
SCF (DCE) Energy = -1454.42738336
SCF (BP86-D3) Energy = -1454.50685147
SCF (BS2) Energy = -1454.43603852

```
Rh -1.10232 -0.49433  0.02175
O  -2.20542  0.79936  1.25200
C  -2.45465  2.04343  0.97219
C  -3.36823  2.73485  1.98370
C  -2.40467 -0.37490 -1.70590
C  -1.08504 -0.00835 -2.15576
C  -0.20035 -1.14950 -1.90433
C  -0.96553 -2.20954 -1.30200
C  -2.34218 -1.72760 -1.14405
O  -2.00049  2.69990 -0.00511
C  -3.47869 -2.53274 -0.59108
C  -3.63764  0.46935 -1.83024
C  -0.72118  1.25418 -2.87798
C   1.23684 -1.22888 -2.31454
C  -0.47818 -3.57111 -0.90336
C   3.44212  0.16389  0.07030
C   3.04273 -1.02638  0.72238
C   4.73073  0.22309 -0.50790
C   3.91038 -2.12746  0.77668
C   5.59577 -0.87916 -0.44565
C   5.18788 -2.06191  0.19315
C   2.90678  2.70798  0.01875
C   1.70692  3.41808 -0.02779
N   1.19156  1.20569 -0.06417
N   0.71859  2.47670 -0.08383
C   2.54295  1.33164  0.00677
O  -1.27797 -1.72017  1.72693
C  -0.48149 -1.22401  2.64142
C  -0.64211 -1.80355  4.03985
O   0.33481 -0.31342  2.38409
H  -3.97699  2.01046  2.54366
H  -2.74019  3.28926  2.70256
H  -4.01002  3.46543  1.46793
H  -4.33764 -1.89480 -0.33337
H  -3.81737 -3.28416 -1.32889
H  -3.16404 -3.06219  0.32295
H  -3.40163  1.53147 -1.66484
H  -4.06670  0.35496 -2.84344
H  -4.40774  0.16945 -1.10308
H   0.34438  1.49304 -2.74699
H  -0.91938  1.15013 -3.96210
H  -1.30888  2.10297 -2.49652
H   1.71746 -0.24162 -2.27719
H   1.81565 -1.90349 -1.66675
H   1.29787 -1.60951 -3.35236
H  -0.80906 -4.33285 -1.63373
H   0.62069 -3.60366 -0.85210
H  -0.87333 -3.84862  0.08728
H   2.06178 -1.04859  1.20949
H   5.04586  1.13673 -1.02416
H   3.59389 -3.03919  1.29632
H   6.58943 -0.81592 -0.90275
H   5.86349 -2.92266  0.24278
H   3.90854  3.12696  0.09945
H  -0.32226  2.60439 -0.11051
H  -1.45037 -1.25971  4.55846
H  -0.92322 -2.86745  4.00056
H   0.28761 -1.67387  4.61275
C   1.41865  4.89007  0.00364
H   0.72425  5.18892 -0.80060
H   0.96232  5.19648  0.96189
H   2.35090  5.46166 -0.12223
```

Int(A-B)
SCF (BP86) Energy = -1454.43051130

Enthalpy 0K = -1453.938496
Energy 298K = -1453.902117
Free Energy 298K = -1454.008480
Lowest Frequency = 17.9881 cm^{-1}
SCF (DCE) Energy = -1454.44607926
SCF (BP86-D3) Energy = -1454.53060745
SCF (BS2) Energy = -1454.45568405

```
Rh -0.69627 -0.48975 -0.05370
O  -2.36508  0.40262  0.92791
C  -3.03844  1.43527  0.53951
C  -4.26075  1.75341  1.39725
C  -2.06537 -1.42750 -1.48599
C  -0.90298 -0.95588 -2.19437
C   0.26490 -1.66594 -1.65683
C  -0.19489 -2.55613 -0.61616
C  -1.63726 -2.39438 -0.47707
O  -2.76081  2.19109 -0.43821
C  -2.52202 -3.16031  0.46273
C  -3.48250 -1.01052 -1.74758
C  -0.88924  0.00428 -3.34904
C   1.64943 -1.58590 -2.22944
C   0.64067 -3.45983  0.24013
C   2.94917  0.63858  0.00316
C   2.86642 -0.47167  0.87475
C   4.17042  0.87396 -0.67440
C   3.97365 -1.31692  1.05106
C   5.27286  0.02820 -0.49184
C   5.17984 -1.07506  0.37273
C   1.97613  2.97591 -0.45638
C   0.68480  3.48189 -0.60759
N   0.50694  1.27227 -0.21172
N  -0.15864  2.42624 -0.46764
C   1.83574  1.58563 -0.20488
O  -0.11174 -0.85372  1.92138
C  -0.10307  0.09577  2.85700
C   0.04802 -0.51089  4.26067
O  -0.20223  1.31274  2.68273
H  -4.63447  0.86071  1.91943
H  -3.96470  2.49666  2.15752
H  -5.05084  2.20066  0.77509
H  -3.39348 -2.55862  0.76481
H  -2.89424 -4.09152 -0.00485
H  -1.97430 -3.43237  1.37838
H  -3.54294  0.04803 -2.04275
H  -3.90916 -1.62517 -2.56222
H  -4.11046 -1.15067 -0.85509
H   0.03636  0.60106 -3.36002
H  -0.95063 -0.53750 -4.31204
H  -1.73908  0.70230 -3.29734
H   1.90736 -0.55511 -2.51695
H   2.41286 -1.92951 -1.51672
H   1.71299 -2.21450 -3.13815
H   0.33464 -4.51305  0.10847
H   1.71000 -3.37428 -0.00300
H   0.50885 -3.18862  1.30314
H   1.93237 -0.66749  1.41039
H   4.24437  1.72095 -1.36483
H   3.89488 -2.16618  1.73907
H   6.20519  0.22906 -1.03059
H   6.04055 -1.73620  0.51943
H   2.90544  3.54173 -0.47080
H  -1.23293  2.37126 -0.51028
H  -0.90358 -0.98451  4.55793
H   0.82538 -1.29174  4.27847
H   0.29333  0.28258  4.98119
C   0.18292  4.87660 -0.83523
H  -0.62303  4.90034 -1.58770
H  -0.21970  5.31442  0.09523
H   1.00124  5.52341 -1.18678
```

TS(A-B)2

SCF (BP86) Energy = -1454.41457778
Enthalpy 0K = -1453.925452
Energy 298K = -1453.889692
Free Energy 298K = -1453.993898
Lowest Frequency = -116.5295 cm^{-1}
SCF (DCE) Energy = -1454.42719211
SCF (BP86-D3) Energy = -1454.51245121
SCF (BS2) Energy = -1454.43833909

```
Rh -0.47241 -0.54491 -0.07630
O  -2.89251  0.59958 -0.46840
C  -3.39961  1.54438  0.18740
C  -4.87441  1.52177  0.55290
C  -1.06220 -1.14411 -2.11579
C   0.37030 -1.16351 -1.98880
C   0.72310 -2.10731 -0.92529
C  -0.52150 -2.70271 -0.42669
C  -1.61060 -2.10802 -1.14499
O  -2.72852  2.57478  0.62460
C  -3.07060 -2.38122 -0.97300
C  -1.86951 -0.38922 -3.12770
C   1.34339 -0.40691 -2.84269
C   2.09950 -2.57830 -0.57269
C  -0.59869 -3.74931  0.64550
C   2.94699  0.92130  0.21171
C   3.01589  0.05050  1.32461
C   4.09479  1.06720 -0.60520
C   4.19919 -0.65090  1.60271
C   5.27299  0.35831 -0.32549
C   5.33079 -0.50389  0.78260
C   1.78448  3.15120 -0.29869
C   0.43888  3.54819 -0.34280
N   0.45079  1.36009  0.03351
N  -0.34192  2.45369 -0.15479
C   1.76109  1.76370 -0.04380
O   0.04380 -0.69661  1.98961
C  -1.09221 -0.62031  2.63331
C  -0.99381 -0.57361  4.15261
O  -2.19101 -0.55712  2.03620
H  -5.46091  1.05237 -0.25120
H  -4.98571  0.90357  1.46020
H  -5.25062  2.53277  0.76611
H  -3.59270 -1.42572 -0.78719
H  -3.47980 -2.83392 -1.89519
H  -3.26450 -3.06302 -0.13229
H  -1.29491  0.44629 -3.55629
H  -2.16620 -1.05902 -3.95750
H  -2.77671  0.02198 -2.66030
H   2.28259 -0.20880 -2.30490
H   1.58400 -0.99660 -3.74729
H   0.92609  0.55829 -3.16770
H   2.86710 -1.82540 -0.80100
H   2.17680 -2.82490  0.49711
H   2.33150 -3.49550 -1.14889
H  -1.62039 -3.84262  1.04211
H  -0.28989 -4.73481  0.25050
H   0.06741 -3.49421  1.48561
H   2.13299 -0.06810  1.96020
H   4.05279  1.74680 -1.46369
H   4.23990 -1.31239  2.47551
H   6.14869  0.48361 -0.97169
H   6.25230 -1.05139  1.00761
H   2.67098  3.78070 -0.36140
H  -1.65162  2.47388  0.25321
H  -0.14080 -1.16571  4.51821
H  -0.83791  0.47299  4.46650
H  -1.93040 -0.93302  4.60360
C  -0.17652  4.90669 -0.52939
H  -1.27022  4.85289 -0.41280
H   0.21147  5.62909  0.20941
H   0.04037  5.31589 -1.53229
```

B·HOAc
SCF (BP86) Energy = -1454.43068929
Enthalpy 0K = -1453.939297
Energy 298K = -1453.903102
Free Energy 298K = -1454.008522
Lowest Frequency = 19.9978 cm^{-1}
SCF (DCE) Energy = -1454.44604799
SCF (BP86-D3) Energy = -1454.52528072
SCF (BS2) Energy = -1454.45522133

```
C   1.18039  2.08876 -0.00930
C   0.64377  3.37320 -0.23236
C  -0.75167  3.20366 -0.27016
N  -1.04438  1.88875 -0.09218
N   0.12602  1.20900  0.07890
Rh -0.00745 -0.87544  0.17722
C   0.00455 -3.01677 -0.30646
C  -1.02557 -2.33565 -1.06793
C  -0.39800 -1.31893 -1.90196
C   1.04270 -1.38568 -1.67005
C   1.28167 -2.41499 -0.67869
C  -2.50201 -2.58449 -1.00167
C  -1.10301 -0.44091 -2.89345
C   2.08501 -0.59559 -2.40408
C   2.62233 -2.82255 -0.14463
C  -0.18755 -4.17569  0.63003
C   2.60754  1.73517  0.09943
C   3.54579  2.42203 -0.71070
C   4.91581  2.13674 -0.63345
C   5.38506  1.15331  0.25420
C   4.46696  0.47234  1.07127
C   3.09361  0.75955  1.00248
O  -1.52644 -0.55249  1.68091
C  -0.64369 -0.48695  2.61183
O   0.58034 -0.72935  2.29154
C  -1.02465 -0.09403  4.01443
O  -3.30313  0.68853 -0.62135
C  -4.41354  1.25620 -0.12330
O  -4.44102  2.30865  0.51468
C  -5.66450  0.44089 -0.43397
H  -1.00044  1.00670  4.09073
H  -2.04821 -0.42725  4.24112
H  -0.31124 -0.51003  4.74089
H  -1.19100 -4.16319  1.08330
H  -0.07200 -5.13822  0.09710
H   0.54913 -4.15644  1.44878
H  -3.04828 -1.62686 -0.95188
H  -2.83203 -3.12994 -1.90550
H  -2.77234 -3.18661 -0.12128
H  -0.52274  0.47152 -3.09856
H  -1.24145 -0.97852 -3.85084
H  -2.09165 -0.13376 -2.51738
H   1.72189  0.41415 -2.64839
H   3.00557 -0.48271 -1.81298
H   2.34229 -1.10307 -3.35315
H   2.54351 -3.20769  0.88402
H   3.05163 -3.62523 -0.77353
H   3.32659 -1.97643 -0.13970
H   3.18271  3.17526 -1.41810
H   5.61935  2.68028 -1.27366
H   4.82348 -0.27662  1.78777
H   6.45517  0.92870  0.31592
H   1.20278  4.30617 -0.29465
H   2.39259  0.25116  1.66988
H  -2.41798  1.21369 -0.32395
H  -6.55783  0.99422 -0.11422
H  -5.72525  0.21887 -1.51231
H  -5.62349 -0.52499  0.09816
C  -1.84347  4.21737 -0.46771
H  -2.80032  3.82531 -0.08562
H  -1.60590  5.15475  0.06311
H  -1.97395  4.47250 -1.53592
```

B
SCF (BP86) Energy = -1225.31574798
Enthalpy 0K = -1224.885438
Energy 298K = -1224.854912
Free Energy 298K = -1224.947127
Lowest Frequency = 15.7925 cm^{-1}
SCF (DCE) Energy = -1225.32976697
SCF (BP86-D3) Energy = -1225.39504076
SCF (BS2) Energy = -1225.26874086

Rh	-0.94114	-0.10848	0.15632
O	-1.85740	0.94144	1.81927
C	-1.22706	0.21157	2.66614
C	-1.28123	0.51428	4.14200
C	-2.75664	-0.73522	-0.91824
C	-2.00727	0.29387	-1.66035
C	-0.71187	-0.26434	-2.00033
C	-0.61683	-1.58441	-1.40923
C	-1.90544	-1.87648	-0.75884
O	-0.51507	-0.76173	2.21289
H	-0.46733	1.21754	4.38917
H	-2.23727	0.99254	4.40145
H	-1.13610	-0.40339	4.73108
C	-2.23528	-3.16624	-0.06507
H	-3.10594	-3.06003	0.59992
H	-2.46630	-3.95641	-0.80366
H	-1.38638	-3.51581	0.54425
C	-4.16135	-0.57102	-0.41552
H	-4.30342	0.41983	0.04525
H	-4.88306	-0.66419	-1.24811
H	-4.41441	-1.33179	0.33864
C	-2.50379	1.65312	-2.05923
H	-1.74300	2.41649	-1.82374
H	-2.72663	1.69177	-3.14191
H	-3.42156	1.91852	-1.51261
C	0.34022	0.43487	-2.80962
H	0.39195	1.50353	-2.54670
H	1.33538	-0.00283	-2.64290
H	0.10137	0.35334	-3.88640
C	0.51860	-2.55353	-1.55890
H	0.61894	-3.19418	-0.66932
H	0.34935	-3.21108	-2.43337
H	1.47939	-2.03546	-1.69776
C	2.73711	-0.09831	0.20277
C	3.84422	-0.27877	-0.66524
C	2.43964	-1.13065	1.12552
C	4.62165	-1.44479	-0.61582
H	4.08318	0.50918	-1.38791
C	3.21703	-2.29821	1.16616
C	4.31020	-2.46496	0.29918
H	5.47193	-1.55826	-1.29767
H	2.97349	-3.07961	1.89524
H	4.91650	-3.37620	0.33940
C	2.45689	2.44675	-0.14055
C	1.32069	3.28458	-0.13687
H	3.50285	2.73106	-0.25663
N	0.60101	1.26955	0.30025
N	0.20092	2.55468	0.10519
C	1.96770	1.15486	0.13141
H	1.60221	-1.00510	1.81720
C	1.22937	4.77426	-0.31814
H	0.17338	5.07517	-0.40969
H	1.66123	5.32061	0.54045
H	1.76878	5.11165	-1.22104

TS(B-C)1
SCF (BP86) Energy = -1225.29970710
Enthalpy 0K = -1224.870846
Energy 298K = -1224.840653
Free Energy 298K = -1224.933044
Lowest Frequency = -66.6254 cm^{-1}
SCF (DCE) Energy = -1225.31450552
SCF (BP86-D3) Energy = -1225.37843885
SCF (BS2) Energy = -1225.25378866

Rh	-0.66059	0.06214	-0.00700
O	-1.16730	0.50374	1.98528
C	-1.42142	-0.61506	2.61638
C	-1.79015	-0.44885	4.08677
C	-2.74210	-0.02000	-0.73681
C	-2.01089	1.19641	-1.14138
C	-0.92013	0.78653	-2.03909
C	-0.90151	-0.64875	-2.07356
C	-2.04418	-1.14320	-1.27242
O	-1.33399	-1.74880	2.08500
H	-0.86625	-0.48718	4.68939
H	-2.27587	0.52023	4.27477
H	-2.44024	-1.27671	4.40689
C	-2.39487	-2.58834	-1.07279
H	-2.80373	-2.76012	-0.06584
H	-3.14238	-2.91332	-1.82088
H	-1.50754	-3.22993	-1.18489
C	-3.96620	-0.04249	0.12871
H	-3.88331	0.69393	0.94405
H	-4.86451	0.20742	-0.46561
H	-4.11751	-1.03397	0.58072
C	-2.35158	2.61636	-0.80842
H	-1.43348	3.15403	-0.50953
H	-2.79499	3.12912	-1.68296
H	-3.06780	2.67184	0.02501
C	0.00230	1.74281	-2.73109
H	0.38274	2.48867	-2.01058
H	0.86401	1.22603	-3.17935
H	-0.53704	2.27643	-3.53505
C	0.03961	-1.51859	-2.85507
H	0.36996	-2.38754	-2.26388
H	-0.45326	-1.89613	-3.77081
H	0.94044	-0.96364	-3.15695
C	2.59145	-0.72408	0.28917
C	3.83382	-1.20583	-0.18955
C	1.58788	-1.67681	0.60250
C	4.04975	-2.57807	-0.37357
H	4.62153	-0.48849	-0.44268
C	1.79862	-3.04991	0.39871
C	3.03197	-3.50849	-0.09235
H	5.01817	-2.92541	-0.75031
H	1.00637	-3.75689	0.66699
H	3.20683	-4.57966	-0.23714
C	3.15707	1.83353	0.51316
C	2.28395	2.94097	0.49577
H	4.24202	1.83763	0.61604
N	1.02685	1.17513	0.33548
N	0.98999	2.52253	0.37310
C	2.31660	0.70649	0.40670
H	0.65792	-1.38093	1.12609
C	2.59588	4.40657	0.61439
H	2.94100	4.67262	1.63018
H	3.38821	4.71554	-0.09011
H	1.69217	4.99996	0.40205

Int(B-C)
SCF (BP86) Energy = -1225.30064270
Enthalpy 0K = -1224.871458
Energy 298K = -1224.840638
Free Energy 298K = -1224.934238
Lowest Frequency = 25.4826 cm^{-1}
SCF (DCE) Energy = -1225.31468457
SCF (BP86-D3) Energy = -1225.38134295
SCF (BS2) Energy = -1225.25494052

Rh	0.53258	-0.18262	-0.03846
O	0.94475	-0.56895	1.98259
C	1.67310	0.34510	2.59108

```
C   1.95196  0.00864  4.05721
C   2.58888 -0.53974 -0.75496
C   1.64080 -1.60143 -1.13594
C   0.65671 -1.00942 -2.05253
C   0.92370  0.39979 -2.12921
C   2.13361  0.68581 -1.33097
O   2.07789  1.40994  2.07972
H   1.13509  0.41758  4.67670
H   1.98855 -1.07822  4.22378
H   2.89230  0.48268  4.37562
C   2.78690  2.02713 -1.16261
H   3.03530  2.19463 -0.10215
H   3.71513  2.08904 -1.76069
H   2.12156  2.84014 -1.49123
C   3.80414 -0.71361  0.10591
H   3.69428 -1.57590  0.78129
H   4.69560 -0.88944 -0.52489
H   3.97855  0.18201  0.72076
C   1.70147 -3.05486 -0.77482
H   0.69342 -3.41367 -0.50191
H   2.07369 -3.65650 -1.62571
H   2.36692 -3.22728  0.08453
C  -0.43707 -1.78197 -2.72561
H  -0.96230 -2.41684 -1.98990
H  -1.17672 -1.11669 -3.19605
H  -0.01390 -2.43538 -3.51050
C   0.17473  1.40974 -2.94902
H   0.05747  2.36124 -2.40670
H   0.71671  1.61870 -3.89051
H  -0.83172  1.04875 -3.20907
C  -2.28705  1.24647  0.36230
C  -3.33053  2.09812 -0.07053
C  -1.01014  1.83104  0.60213
C  -3.09659  3.46243 -0.28915
H  -4.32037  1.67259 -0.26547
C  -0.77489  3.19713  0.36578
C  -1.81789  4.01890 -0.08761
H  -3.91894  4.10023 -0.63165
H   0.21413  3.61074  0.58749
H  -1.64772  5.08686 -0.25772
C  -3.53813 -1.07293  0.60997
C  -2.97267 -2.36449  0.55876
H  -4.58622 -0.80629  0.74416
N  -1.31610 -0.97722  0.36386
N  -1.61647 -2.28589  0.39359
C  -2.44033 -0.19731  0.48184
H  -0.23932  1.29181  1.19845
C  -3.64080 -3.70487  0.68507
H  -3.97356 -3.90306  1.72039
H  -4.53079 -3.78077  0.03606
H  -2.93578 -4.50289  0.40241
```

TS(B-C)2
SCF (BP86) Energy = -1225.29594584
Enthalpy 0K = -1224.870825
Energy 298K = -1224.840941
Free Energy 298K = -1224.931423
Lowest Frequency = -605.9897 cm^{-1}
SCF (DCE) Energy = -1225.30819841
SCF (BP86-D3) Energy = -1225.37626833
SCF (BS2) Energy = -1225.24786929

```
Rh  0.58201 -0.12237  0.06961
O   0.68885 -0.98716  2.00711
C   0.07233 -0.44299  2.99480
C   0.12646 -1.17159  4.32691
C   2.75933 -0.49737 -0.23419
C   1.96458 -1.47460 -0.97489
C   1.18304 -0.74124 -1.95440
C   1.44798  0.67397 -1.78530
C   2.45377  0.81892 -0.72569
O  -0.59262  0.64208  2.92530
H  -0.78831 -1.78013  4.42811
H   0.99679 -1.84115  4.37361
H   0.14573 -0.44798  5.15510
C   3.13732  2.10045 -0.33840
H   3.45351  2.09316  0.71701
H   4.04101  2.26219 -0.95659
H   2.47668  2.96832 -0.48679
C   3.72075 -0.84352  0.86540
H   3.24278 -1.50904  1.60320
H   4.60397 -1.36608  0.45500
H   4.07281  0.05499  1.39471
C   1.98324 -2.96760 -0.81159
H   0.95976 -3.37325 -0.87679
H   2.60107 -3.44957 -1.59329
H   2.39456 -3.25438  0.16861
C   0.26177 -1.37978 -2.95110
H  -0.40310 -2.10481 -2.44920
H  -0.36736 -0.63501 -3.46129
H   0.84860 -1.91695 -3.71860
C   0.92553  1.78675 -2.64839
H   0.73809  2.69834 -2.05919
H   1.65262  2.03488 -3.44440
H  -0.02246  1.50641 -3.13250
C  -2.03985  1.36092 -0.03809
C  -2.88800  2.43128 -0.39370
C  -0.71486  1.62558  0.47110
C  -2.43696  3.75380 -0.28690
H  -3.89209  2.22253 -0.77909
C  -0.29663  2.97820  0.56271
C  -1.13907  4.03426  0.18690
H  -3.10200  4.57647 -0.57229
H   0.68566  3.20234  0.99570
H  -0.80272  5.07207  0.28580
C  -3.50685 -0.82785 -0.47740
C  -3.01499 -2.14983 -0.53829
H  -4.53577 -0.49210 -0.60340
N  -1.30355 -0.90125 -0.09660
N  -1.66229 -2.17427 -0.31689
C  -2.37169 -0.04120 -0.19470
H  -0.51904  1.04729  1.62271
C  -3.76224 -3.43376 -0.76809
H  -3.05060 -4.25143 -0.96530
H  -4.36922 -3.72199  0.10991
H  -4.45034 -3.35629 -1.62810
```

C1
SCF (BP86) Energy = -1225.30800003
Enthalpy 0K = -1224.878191
Energy 298K = -1224.847808
Free Energy 298K = -1224.939455
Lowest Frequency = 16.2614 cm^{-1}
SCF (DCE) Energy = -1225.31911555
SCF (BP86-D3) Energy = -1225.38731265
SCF (BS2) Energy = -1225.26006903

```
Rh -0.58137 -0.00253  0.06071
O  -0.32272  0.00509  2.24732
C   0.58533 -0.50033  2.94408
C   0.73109 -0.17081  4.40818
C  -2.71194  0.87005  0.26536
C  -2.03868  1.55954 -0.77598
C  -1.57472  0.54995 -1.74746
C  -2.11133 -0.74642 -1.33551
C  -2.74135 -0.57073 -0.05649
O   1.49879 -1.34128  2.48235
H   1.54479  0.56590  4.52380
H  -0.19979  0.26793  4.79047
H   1.00683 -1.06804  4.98258
C  -3.48021 -1.60964  0.74111
H  -3.36829 -1.43639  1.82380
H  -4.56375 -1.59299  0.51379
H  -3.11158 -2.62484  0.52361
```

```
C  -3.28406  1.45536  1.52424
H  -3.11857  2.54219  1.57551
H  -4.37147  1.26812  1.58891
H  -2.81292  1.00381  2.41490
C  -1.75721  3.03064 -0.87974
H  -0.68027  3.22728 -0.70332
H  -2.02662  3.40853 -1.88181
H  -2.33373  3.60271 -0.13550
C  -0.91092  0.86575 -3.05813
H  -0.18936  1.68928 -2.94161
H  -0.36772 -0.00692 -3.45279
H  -1.65914  1.17122 -3.81467
C  -2.08767 -1.99729 -2.16565
H  -2.23063 -2.90053 -1.55245
H  -2.90525 -1.96695 -2.91033
H  -1.13678 -2.10840 -2.70891
C   2.13035 -0.99649 -0.67215
C   3.14442 -1.90089 -1.05340
C   0.81429 -1.47826 -0.33892
C   2.88333 -3.27883 -1.10143
H   4.13994 -1.51983 -1.30825
C   0.59342 -2.87167 -0.37459
C   1.61420 -3.76896 -0.75215
H   3.67754 -3.97285 -1.39730
H  -0.38800 -3.27274 -0.09338
H   1.41449 -4.84631 -0.76891
C   3.28800  1.42015 -0.68947
C   2.64406  2.63644 -0.36073
H   4.32539  1.27475 -0.98983
N   1.14674  1.08867 -0.13137
N   1.33634  2.41680 -0.02957
C   2.28758  0.44261 -0.53816
H   1.33143 -1.47402  1.49224
C   3.20579  4.03081 -0.32705
H   3.98048  4.14682  0.45311
H   3.67124  4.30988 -1.28941
H   2.40043  4.75147 -0.11288
```

TS(C1-C2)
SCF (BP86) Energy = -1225.30642933
Enthalpy 0K = -1224.876733
Energy 298K = -1224.846982
Free Energy 298K = -1224.936793
Lowest Frequency = -87.6843 cm^{-1}
SCF (DCE) Energy = -1225.31699708
SCF (BP86-D3) Energy = -1225.38604001
SCF (BS2) Energy = -1225.25864462

```
Rh -0.59128 -0.00314  0.05698
O  -0.39020 -0.25015  2.23505
C   0.61311 -0.44624  2.95286
C   0.53470 -0.40099  4.45783
C  -2.71741  0.87780  0.29350
C  -2.00866  1.65663 -0.65711
C  -1.53151  0.73818 -1.70993
C  -2.08805 -0.58555 -1.43842
C  -2.75293 -0.52490 -0.16436
O   1.83395 -0.69494  2.49180
H   1.02151  0.52252  4.81546
H  -0.51396 -0.40609  4.78141
H   1.07904 -1.25262  4.89453
C  -3.52251 -1.62782  0.50809
H  -3.43651 -1.56475  1.60511
H  -4.59962 -1.57723  0.25639
H  -3.15745 -2.62056  0.19935
C  -3.32122  1.34506  1.58664
H  -3.11008  2.40934  1.77127
H  -4.41807  1.20766  1.58159
H  -2.91750  0.76907  2.43743
C  -1.70657  3.12712 -0.61565
H  -0.61683  3.29008 -0.50182
H  -2.04263  3.62078 -1.54530
H  -2.21423  3.61830  0.22962
C  -0.81754  1.17177 -2.95892
H  -0.08658  1.96393 -2.73321
H  -0.27555  0.33324 -3.42301
H  -1.53425  1.56827 -3.70340
C  -2.04105 -1.75833 -2.37468
H  -2.11724 -2.71648 -1.83795
H  -2.88472 -1.70383 -3.08830
H  -1.10553 -1.77640 -2.95423
C   2.06294 -1.11467 -0.69295
C   3.03922 -2.07389 -1.04055
C   0.72696 -1.52682 -0.36320
C   2.71406 -3.43874 -1.05187
H   4.05431 -1.74620 -1.29322
C   0.43956 -2.90299 -0.35097
C   1.42081 -3.85617 -0.69628
H   3.47515 -4.17796 -1.32410
H  -0.56240 -3.24854 -0.06773
H   1.17092 -4.92338 -0.68514
C   3.32445  1.25705 -0.74866
C   2.74609  2.49760 -0.38659
H   4.34274  1.07017 -1.08855
N   1.19169  1.01344 -0.10951
N   1.44432  2.33361 -0.00652
C   2.28904  0.32269 -0.57033
H   1.80921 -0.68595  1.49649
C   3.37102  3.86504 -0.36477
H   4.18384  3.93645  0.38099
H   3.80565  4.13360 -1.34433
H   2.60924  4.61834 -0.10808
```

C2
SCF (BP86) Energy = -1225.32351322
Enthalpy 0K = -1224.892742
Energy 298K = -1224.862515
Free Energy 298K = -1224.953220
Lowest Frequency = 25.1338 cm^{-1}
SCF (DCE) Energy = -1225.33445754
SCF (BP86-D3) Energy = -1225.40401236
SCF (BS2) Energy = -1225.27707824

```
Rh  0.51905  0.03489  0.03285
O   0.00647 -0.72787  1.95215
C  -0.33920 -1.98126  2.03668
C  -0.60595 -2.45369  3.46864
C   2.50659 -1.09212 -0.03928
C   1.82810 -1.34831 -1.26388
C   1.55735 -0.04982 -1.89579
C   2.16243  0.98673 -1.08231
C   2.69739  0.35714  0.10969
O  -0.47213 -2.80587  1.08910
C   3.50650  1.02132  1.18874
C   2.96051 -2.11930  0.95743
C   1.45109 -2.69702 -1.81105
C   0.92426  0.14148 -3.24598
C   2.29908  2.43448 -1.45798
C  -1.83423  1.85590  0.01952
C  -2.59476  3.03201  0.16396
C  -0.46824  1.79009  0.44958
C  -2.02181  4.16361  0.76324
C   0.06309  2.92771  1.08259
C  -0.70010  4.10344  1.23494
C  -3.62658 -0.01614 -0.71449
C  -3.39109 -1.38610 -0.85425
N  -1.42064 -0.39276 -0.48812
N  -2.03280 -1.58001 -0.74285
C  -2.35925  0.57589 -0.45010
C  -4.33195 -2.52537 -1.10640
H  -0.05357 -3.38914  3.65452
H  -0.32497 -1.69345  4.21108
H  -1.67972 -2.68376  3.57597
H  -1.51115 -2.37193 -0.29933
```

H	3.32499	0.55644	2.17123
H	4.59146	0.94983	0.97932
H	3.25859	2.09145	1.27567
H	2.35349	-3.03469	0.89260
H	4.01865	-2.38940	0.78038
H	2.88416	-1.73341	1.98658
H	0.61409	-2.61895	-2.52374
H	2.29811	-3.16428	-2.34809
H	1.13735	-3.37466	-1.00097
H	0.10881	-0.58057	-3.41242
H	0.50092	1.15238	-3.35159
H	1.66763	-0.00120	-4.05308
H	2.23046	3.09420	-0.57929
H	3.27853	2.61082	-1.94109
H	1.51293	2.74582	-2.16305
H	-3.63689	3.05509	-0.17683
H	-2.60898	5.08063	0.87861
H	1.08367	2.90463	1.48291
H	-0.25709	4.97531	1.73075
H	-4.59430	0.47905	-0.76738
H	-5.37259	-2.18702	-0.99019
H	-4.21935	-2.93316	-2.12682
H	-4.16235	-3.35486	-0.39788

D

SCF (BP86) Energy = -1309.44656613
Enthalpy 0K = -1308.882823
Energy 298K = -1308.846265
Free Energy 298K = -1308.951758
Lowest Frequency = 18.3071 cm^{-1}
SCF (DCE) Energy = -1309.45527092
SCF (BP86-D3) Energy = -1309.55106517
SCF (BS2) Energy = -1309.40113573

C	1.52649	-0.66997	1.97028
C	2.81647	-0.51982	2.51361
C	3.58591	0.01712	1.45657
N	2.81820	0.18714	0.33654
N	1.58267	-0.23319	0.67419
Rh	-0.10461	-0.36830	-0.48699
C	-0.15099	1.80078	-0.21224
C	0.95997	2.74292	-0.46142
C	0.81121	4.07529	0.31231
C	1.96406	5.04494	0.01245
C	0.21946	-1.16895	2.36153
C	-0.11091	-1.69815	3.62752
C	-1.40677	-2.17096	3.88194
C	-2.38254	-2.11603	2.87381
C	-2.05934	-1.58336	1.60747
C	-0.76673	-1.11226	1.32906
C	-1.29130	1.37497	0.14597
C	-2.67126	1.52637	0.66312
C	-3.07914	3.00547	0.85893
C	-4.51056	3.14597	1.39780
C	5.04238	0.39097	1.43317
H	3.14799	-0.76435	3.52233
H	1.91110	2.24318	-0.19209
H	1.02168	2.95032	-1.54950
H	-0.15560	4.54616	0.05437
H	0.77440	3.85305	1.39403
H	1.85096	5.98645	0.57608
H	2.93736	4.60264	0.28707
H	2.00488	5.30071	-1.06145
H	0.65498	-1.73864	4.41065
H	-1.65534	-2.58023	4.86711
H	-3.39792	-2.48104	3.06703
H	-2.84176	-1.53058	0.84004
H	-2.76026	0.98091	1.62055
H	-3.38581	1.03369	-0.02642
H	-2.36393	3.48193	1.55344
H	-2.98260	3.53986	-0.10444
H	-4.62094	2.64350	2.37473
H	-4.78400	4.20589	1.53348
H	-5.24616	2.69675	0.70694
H	5.31228	0.78024	0.43835
H	5.27983	1.16968	2.18056
H	5.69463	-0.47447	1.64971
C	-0.41732	-0.19765	-2.77403
C	0.88948	-0.72539	-2.52504
C	0.72265	-1.98287	-1.79142
C	-0.69136	-2.24096	-1.64798
C	-1.40358	-1.10939	-2.19788
C	-0.76559	1.06347	-3.51257
C	2.21872	-0.15906	-2.93940
C	1.85398	-2.88933	-1.39664
C	-1.30704	-3.50020	-1.10994
C	-2.89364	-0.99528	-2.37022
H	0.13699	1.61140	-3.82404
H	-1.35381	0.83754	-4.42041
H	-1.37021	1.74031	-2.88285
H	2.85925	-0.00528	-2.05069
H	2.73808	-0.84458	-3.63404
H	2.10321	0.81063	-3.44932
H	1.55313	-3.59284	-0.60508
H	2.19922	-3.48214	-2.26472
H	2.70751	-2.30107	-1.02288
H	-1.50584	-4.20858	-1.93621
H	-0.64211	-3.99893	-0.38850
H	-2.26135	-3.30495	-0.59682
H	-3.21866	0.05765	-2.38276
H	-3.21892	-1.45181	-3.32486
H	-3.43690	-1.50916	-1.56055

TS(D-E)

SCF (BP86) Energy = -1309.42644448
Enthalpy 0K = -1308.863430
Energy 298K = -1308.827793
Free Energy 298K = -1308.930395
Lowest Frequency = -296.9066 cm^{-1}
SCF (DCE) Energy = -1309.43440443
SCF (BP86-D3) Energy = -1309.53035305
SCF (BS2) Energy = -1309.38025528

C	0.77884	1.10365	-1.95459
C	0.80794	2.30059	-2.70022
C	-0.35541	2.99262	-2.30241
N	-1.05630	2.27001	-1.37011
N	-0.35893	1.13599	-1.18717
Rh	-0.70091	-0.39253	0.15101
C	0.51031	0.87990	1.24140
C	0.20092	2.19591	1.84538
C	1.42655	2.92556	2.44244
C	1.05660	4.29683	3.02720
C	1.53948	-0.12010	-1.82832
C	2.50289	-0.56712	-2.75719
C	3.05513	-1.85141	-2.65465
C	2.63347	-2.71573	-1.62481
C	1.70846	-2.27111	-0.66885
C	1.17335	-0.95731	-0.71018
C	-2.31851	-0.91067	1.67487
C	-2.87615	-0.17056	0.57536
C	-2.69685	-0.98218	-0.64253
C	-2.03892	-2.20252	-0.27954
C	-1.74602	-2.13563	1.14628
C	-2.34014	-0.52373	3.12810
C	-3.60395	1.14447	0.61353
C	-3.20693	-0.57815	-1.99655
C	-1.71003	-3.35833	-1.18144
C	-1.15194	-3.24937	1.96537
C	1.37027	-0.07248	1.00323
C	2.58081	-0.65742	1.68608
C	3.92111	-0.24685	1.03460
C	5.12845	-0.85085	1.76766
H	2.77943	0.08622	-3.59240

H   3.79267 -2.19019 -3.38978
H   1.42023 -2.94354 0.14837
H   3.03380 -3.73380 -1.56177
H   1.56129 2.62058 -3.41949
C -0.87761 4.32397 -2.76689
H   2.50856 -1.75996 1.69664
H   2.56832 -0.32966 2.74353
H -0.57546 2.06691 2.62681
H -0.27113 2.82353 1.06021
H   3.99187 0.85636 1.03361
H   3.92096 -0.56377 -0.02244
H   5.15724 -0.54033 2.82766
H   5.09781 -1.95483 1.74488
H   6.07701 -0.53458 1.30193
H   1.88542 2.29432 3.22618
H   2.18741 3.04542 1.64996
H   0.31574 4.20025 3.84095
H   1.94196 4.80872 3.44073
H   0.61906 4.95323 2.25476
H -1.94084 -3.94324 2.31461
H -0.43298 -3.84430 1.37914
H -0.63160 -2.86786 2.85866
H -1.37651 -0.73708 3.62037
H -2.55368 0.54893 3.25752
H -3.12123 -1.08568 3.67374
H -4.67899 1.00990 0.39016
H -3.52562 1.61844 1.60518
H -3.17295 1.83984 -0.12943
H -2.75336 -1.18007 -2.79929
H -4.30430 -0.70545 -2.05320
H -2.97572 0.48225 -2.19346
H -1.85079 -3.09183 -2.24037
H -0.66148 -3.68207 -1.06208
H -2.35832 -4.22752 -0.96371
H -1.74578 4.61840 -2.15578
H -0.11217 5.11569 -2.68275
H -1.20107 4.29654 -3.82372

E

SCF (BP86) Energy = -1309.45496814
Enthalpy 0K = -1308.889719
Energy 298K = -1308.853877
Free Energy 298K = -1308.958561
Lowest Frequency = 13.0824 cm^{-1}
SCF (DCE) Energy = -1309.46476732
SCF (BP86-D3) Energy = -1309.55566104
SCF (BS2) Energy = -1309.40758342

C -1.18138 -0.27044 1.87062
C -1.52429 0.26160 3.12692
C -0.53227 1.23674 3.38649
N   0.37289 1.27916 2.36845
N -0.03315 0.35475 1.46555
Rh 0.92615 -0.17623 -0.25371
C -0.64923 0.74260 -1.13844
C -0.56237 2.14120 -1.70334
C -0.88128 3.22651 -0.64391
C -0.69672 4.65292 -1.18264
C -1.69603 -1.40049 1.07708
C -2.00016 -2.61429 1.73827
C -2.40821 -3.75316 1.03470
C -2.49943 -3.70393 -0.36742
C -2.22408 -2.50491 -1.03648
C -1.83869 -1.32641 -0.34887
C -1.77582 -0.02462 -1.11382
C -3.07573 0.33733 -1.83563
C -4.23162 0.69604 -0.87231
C -5.54240 1.00662 -1.61147
C   2.56036 0.14062 -1.70423
C   2.79815 0.78187 -0.40301
C   3.05740 -0.28520 0.59672
C   2.81754 -1.52356 -0.03298
C   2.45678 -1.26244 -1.44714
C   2.53788 0.80815 -3.04975
C   3.01606 2.24040 -0.13467
C   3.39498 -0.00987 2.03016
C   2.87173 -2.89262 0.58237
C   2.18136 -2.33783 -2.45955
H   1.65944 -1.93691 -3.34213
H   3.12560 -2.80073 -2.80543
H   1.55848 -3.14075 -2.03254
H   2.28832 1.87755 -2.97185
H   3.53252 0.73813 -3.52964
H   1.80503 0.34106 -3.72706
H   2.57564 2.51382 0.83861
H   4.09809 2.47472 -0.10428
H   2.55484 2.86976 -0.91166
H   2.60310 0.61899 2.48460
H   3.47625 -0.93947 2.61463
H   4.35567 0.53070 2.11045
H   1.96776 -3.47828 0.34308
H   3.74093 -3.46283 0.20385
H   2.95316 -2.83933 1.67870
H -1.88999 -2.64740 2.82759
H -2.63806 -4.67829 1.57410
H -2.32983 -2.46268 -2.12662
H -2.79760 -4.59174 -0.93562
H -2.37981 -0.00844 3.74581
C -0.38728 2.15758 4.56609
H -3.40166 -0.51575 -2.46392
H -2.90787 1.18063 -2.52862
H -1.25297 2.27060 -2.56231
H   0.45000 2.33253 -2.10264
H -1.92170 3.08577 -0.29767
H -0.24438 3.06134 0.24359
H -0.94015 5.40752 -0.41531
H   0.34629 4.83062 -1.50312
H -1.34581 4.84320 -2.05662
H -4.38871 -0.14111 -0.16746
H -3.92937 1.56280 -0.25594
H -5.88080 0.13998 -2.20738
H -6.35172 1.26327 -0.90660
H -5.42142 1.85797 -2.30525
H   0.51131 2.78302 4.44220
H -1.25789 2.82965 4.67345
H -0.28913 1.60047 5.51549

TS(E-F)

SCF (BP86) Energy = -1309.44197228
Enthalpy 0K = -1308.877922
Energy 298K = -1308.842814
Free Energy 298K = -1308.944235
Lowest Frequency = -120.0877 cm^{-1}
SCF (DCE) Energy = -1309.44928171
SCF (BP86-D3) Energy = -1309.54306254
SCF (BS2) Energy = -1309.39420078

C   0.98980 2.20696 -0.53256
C   0.75491 3.58543 -0.41550
C -0.61877 3.69792 -0.09236
N -1.19674 2.46995 -0.01149
N -0.21951 1.57045 -0.30288
Rh -0.64034 -0.50772 -0.20637
C   0.35283 0.13118 1.26207
C   0.20813 0.82292 2.56743
C -1.22721 0.97914 3.09842
C -1.27691 1.72383 4.44053
C   2.17815 1.44522 -0.89606
C   3.13374 2.01660 -1.76348
C   4.24983 1.29527 -2.20244
C   4.40462 -0.03800 -1.78770
C   3.47301 -0.61735 -0.91754
C   2.35610 0.10451 -0.42057
C   1.43542 -0.52151 0.56335

C 1.94280 -1.76958 1.31627
C 3.15319 -1.52913 2.24743
C 3.61790 -2.81609 2.94709
C -2.51149 -1.79910 0.06737
C -2.88429 -0.66583 -0.71631
C -2.09369 -0.70413 -1.95901
C -1.27584 -1.87336 -1.95823
C -1.45579 -2.51086 -0.65756
C -3.13593 -2.24880 1.36058
C -3.92157 0.37499 -0.40041
C -2.21541 0.31948 -3.05429
C -0.39209 -2.36904 -3.06972
C -0.85632 -3.83046 -0.25139
H -0.78544 -3.92614 0.84433
H -1.47149 -4.67459 -0.61828
H 0.15691 -3.95680 -0.66632
H -3.57156 -1.40374 1.91672
H -3.94687 -2.97904 1.17332
H -2.40148 -2.73994 2.01949
H -3.47081 1.38323 -0.40881
H -4.74812 0.35355 -1.13567
H -4.35868 0.21232 0.59778
H -2.25606 1.33836 -2.63538
H -1.36487 0.27222 -3.75241
H -3.14096 0.16040 -3.63912
H 0.53058 -2.83084 -2.68103
H -0.90836 -3.13030 -3.68617
H -0.09002 -1.54924 -3.74107
H 2.96508 3.03884 -2.11863
H 4.97619 1.75866 -2.87768
H 3.62694 -1.65383 -0.60453
H 5.25808 -0.63021 -2.13525
H 1.48376 4.38770 -0.52547
C -1.44177 4.93602 0.12380
H 1.10955 -2.18495 1.91273
H 2.19055 -2.56170 0.58217
H 0.69746 1.81678 2.46799
H 0.84856 0.27262 3.29120
H -1.67802 -0.02497 3.20152
H -1.81398 1.51555 2.33289
H -2.31355 1.82444 4.80350
H -0.70032 1.19583 5.22171
H -0.85674 2.74119 4.34897
H 2.88182 -0.77118 3.00718
H 3.98604 -1.09184 1.66920
H 3.93339 -3.57866 2.21270
H 4.47345 -2.62470 3.61663
H 2.80890 -3.25850 3.55580
H -2.43780 4.66395 0.50721
H -0.96277 5.61782 0.84831
H -1.58050 5.50506 -0.81348

F
SCF (BP86) Energy = -1309.48949239
Enthalpy 0K = -1308.922380
Energy 298K = -1308.887350
Free Energy 298K = -1308.987778
Lowest Frequency = 14.2501 cm^{-1}
SCF (DCE) Energy = -1309.49649904
SCF (BP86-D3) Energy = -1309.59124089
SCF (BS2) Energy = -1309.43876103

C 1.34914 0.25491 -0.26520
N 1.89804 -1.06250 0.00090
C 1.23372 -1.91698 0.83387
C 0.02070 -1.37427 1.45921
C -0.00106 0.06454 1.78381
C 0.73402 0.94868 0.88234
C -0.81388 0.50074 2.89295
C -1.63629 -0.38689 3.56122
C -1.69879 -1.76235 3.17539
C -0.88092 -2.24077 2.16689
C 1.98452 -3.09420 0.86700
C 3.08714 -2.84450 -0.00180
N 3.02991 -1.59853 -0.53019
Rh -0.79607 0.02713 -0.26001
C -2.60493 1.27496 -1.02921
C -1.64513 0.93222 -2.07603
C -1.65269 -0.52107 -2.25817
C -2.51588 -1.05771 -1.24864
C -3.11308 0.05442 -0.49740
C -1.00237 1.91784 -3.01225
C -0.95691 -1.28002 -3.35516
C -2.86771 -2.50529 -1.04296
C -4.12764 -0.10519 0.60076
C -2.99524 2.67111 -0.62809
C 0.87784 2.43053 1.17180
C 1.92165 2.73720 2.27454
C 2.07490 4.24229 2.53963
C 2.07278 1.00073 -1.37240
C 3.50883 1.48388 -1.05103
C 4.15565 2.19256 -2.25074
H -2.14298 3.36725 -0.69954
H -3.79764 3.06978 -1.27872
H -3.36591 2.70631 0.40946
H -0.07881 1.51522 -3.45725
H -1.68809 2.16819 -3.84473
H -0.74981 2.86187 -2.50136
H -0.74274 -2.31784 -3.05288
H -1.57399 -1.31996 -4.27407
H 0.00489 -0.81319 -3.62397
H -2.09874 -3.17252 -1.46395
H -2.97219 -2.74369 0.02885
H -3.83003 -2.75134 -1.53173
H -4.27653 0.83754 1.15102
H -5.11118 -0.41483 0.19834
H -3.81367 -0.86812 1.33341
H -0.86560 -3.30906 1.92365
H -2.36559 -2.44537 3.71193
H -0.77231 1.54993 3.20216
H -2.24851 -0.03409 4.39804
H 1.78005 -3.99444 1.44413
C 4.22320 -3.75856 -0.36349
H -0.10002 2.85139 1.47486
H 1.16346 2.96166 0.24634
H 2.11988 0.33233 -2.25226
H 1.45597 1.86715 -1.66758
H 2.89617 2.30448 1.98198
H 1.62797 2.21866 3.20642
H 2.81612 4.43844 3.33272
H 2.40736 4.77663 1.63185
H 1.11774 4.69337 2.85740
H 3.48286 2.17082 -0.18478
H 4.11286 0.61052 -0.75478
H 3.56958 3.07504 -2.56685
H 5.17576 2.53868 -2.01200
H 4.22937 1.51449 -3.11957
H 4.89069 -3.25595 -1.08048
H 4.81923 -4.04048 0.52297
H 3.86252 -4.69539 -0.82468

Methyl Acrylate (Y = C(O)OMe)

C_2H_3COOMe (a)
SCF Energy = -306.466039947
Enthalpy 0K = -306.373579
Energy 298K = -306.366735
Free Energy 298K = -306.404403
Lowest Frequency = 85.3951 cm^{-1}
SCF (DCE) Energy = -306.470441841
SCF (BP86-D3) Energy = -306.473475227
SCF (BS2) Energy = -306.558965002

C	1.49353	0.39029	0.00001
C	2.18971	-0.76122	-0.00002
H	3.28421	-0.76225	-0.00007
H	1.99152	1.36520	-0.00001
H	1.68316	-1.73053	0.00003
C	0.00928	0.48774	0.00003
O	-0.60438	1.55059	-0.00001
O	-0.60077	-0.73660	0.00004
C	-2.04425	-0.67717	-0.00002
H	-2.41300	-0.14883	-0.89418
H	-2.38115	-1.72283	-0.00108
H	-2.41314	-0.15062	0.89514

2,1-insertion

$D1_{2,1}$
SCF Energy = -1302.69637197
Enthalpy 0K = -1302.232310
Energy 298K = -1302.200395
Free Energy 298K = -1302.294393
Lowest Frequency = 15.4108 cm^{-1}
SCF (DCE) Energy = -1302.70651699
SCF (BP86-D3) Energy = -1302.79045137
SCF (BS2) Energy = -1302.66530295

C	0.11688	2.43413	0.28737
C	1.01934	3.39145	0.78453
C	2.11072	2.63237	1.26991
N	1.89593	1.29559	1.08045
N	0.68726	1.20563	0.49664
C	3.37987	3.10497	1.92320
Rh	-0.34950	-0.46348	-0.11705
C	0.76905	-0.16856	-1.96919
C	2.16248	-0.68711	-1.87014
O	2.50820	-1.86286	-1.73493
C	-1.18614	2.37416	-0.35503
C	-2.00508	3.48272	-0.65880
C	-3.25171	3.29567	-1.27480
C	-3.68992	2.00162	-1.59652
C	-2.87481	0.88723	-1.30185
C	-1.62924	1.05406	-0.67784
C	-1.57465	-2.28616	0.42709
C	-0.17093	-2.72781	0.48252
C	0.50247	-1.95714	1.46975
C	-0.48410	-1.05015	2.06558
C	-1.77163	-1.29913	1.46206
C	-2.66699	-2.94350	-0.37058
C	0.42713	-3.84961	-0.31834
C	1.94519	-2.04892	1.87588
C	-0.19578	-0.13083	3.21425
C	-3.08278	-0.69379	1.87486
C	-0.33603	-1.02539	-2.21013
O	3.04901	0.33972	-1.98685
H	-1.65938	4.49214	-0.40821
H	-3.87997	4.16226	-1.50701
H	-3.22805	-0.11630	-1.57061
H	-4.66058	1.85149	-2.08260
H	0.90449	4.47487	0.79350
H	0.69026	0.89588	-2.21131
H	-0.14885	-2.09990	-2.31176
H	-1.22036	-0.63174	-2.71950
H	-3.61575	-1.36821	2.57092
H	-2.93726	0.27090	2.38438
H	-3.73714	-0.51124	1.00799
H	-3.04802	-3.83986	0.15443
H	-3.51974	-2.26220	-0.51937
H	-2.31568	-3.26723	-1.36326
H	0.71832	-4.67861	0.35192
H	-0.29136	-4.25457	-1.04847
H	1.32559	-3.50990	-0.86138
H	2.52537	-2.62100	1.13600
H	2.38139	-1.03647	1.94508
H	2.05158	-2.54461	2.85928
H	-0.14827	-0.71129	4.15474
H	0.77341	0.37387	3.06831
H	-0.97331	0.63962	3.32825
H	3.18134	3.67420	2.84931
H	4.01072	2.23983	2.18250
H	3.96280	3.76511	1.25576
C	4.43713	-0.03970	-1.85946
H	5.00942	0.87504	-2.06403
H	4.64092	-0.39978	-0.83854
H	4.69304	-0.83260	-2.58037

$TS(D1\text{-}E1)_{2,1}$
SCF Energy = -1302.67466331
Enthalpy 0K = -1302.210617
Energy 298K = -1302.179624
Free Energy 298K = -1302.270613
Lowest Frequency = -261.5425 cm^{-1}
SCF (DCE) Energy = -1302.68451736
SCF (BP86-D3) Energy = -1302.76787125
SCF (BS2) Energy = -1302.64361335

C	-2.46134	0.76372	-0.74777
C	-3.36906	1.84010	-0.81556
C	-2.63733	2.94933	-0.33871
N	-1.36229	2.57962	0.00176
N	-1.27619	1.26575	-0.26139
C	-3.06443	4.38446	-0.20321
Rh	0.22587	-0.07261	0.17651
C	1.07052	0.56361	-1.67567
C	2.52013	0.29749	-1.82478
O	3.07239	-0.80542	-1.88017
C	-2.46125	-0.66670	-0.98931
C	-3.61420	-1.45482	-1.17994
C	-3.51505	-2.84763	-1.31863
C	-2.25667	-3.47480	-1.26441
C	-1.09502	-2.70370	-1.09549
C	-1.16732	-1.29899	-0.96260
C	1.49127	-1.43314	1.47294
C	2.05739	-0.10102	1.51194
C	1.05066	0.80772	2.01109
C	-0.12682	0.00304	2.37035
C	0.15641	-1.36835	2.06101
C	2.22710	-2.68130	1.06951
C	3.48302	0.24182	1.19396
C	1.17990	2.28535	2.24918
C	-1.36832	0.56560	3.00067
C	-0.73164	-2.54713	2.33266
C	0.15983	-0.50628	-2.09901
O	3.21849	1.47530	-1.94430
H	-4.59605	-0.96865	-1.19244
H	-4.42060	-3.44566	-1.46524
H	-0.11296	-3.19125	-1.11190
H	-2.17618	-4.56174	-1.37409
H	-4.39994	1.82216	-1.16812
H	0.77436	1.60069	-1.86326
H	0.66793	-1.41552	-2.43802
H	-0.63576	-0.16454	-2.77036

H	-0.35840	-3.11552	3.20468
H	-1.76363	-2.23306	2.55135
H	-0.77137	-3.23444	1.47178
H	2.90033	-3.02571	1.87778
H	1.52715	-3.50372	0.85224
H	2.83668	-2.50422	0.16837
H	4.10599	0.10595	2.09799
H	3.88520	-0.41242	0.40484
H	3.58880	1.28752	0.86624
H	2.10549	2.68174	1.80322
H	0.32858	2.82097	1.79053
H	1.19972	2.51108	3.33195
H	-1.20498	0.75012	4.07895
H	-1.64028	1.52397	2.52858
H	-2.22429	-0.11915	2.89943
H	-4.02327	4.47643	0.33668
H	-2.29922	4.95103	0.35084
H	-3.19687	4.87054	-1.18721
C	4.62853	1.31673	-2.21314
H	5.01112	2.33382	-2.37590
H	5.14558	0.84589	-1.36101
H	4.78721	0.69360	-3.10805

E1$_{2,1}$

SCF (BP86) Energy = -1302.68966442
Enthalpy 0K = -1302.224997
Energy 298K = -1302.193296
Free Energy 298K = -1302.287276
Lowest Frequency = 17.8013 cm^{-1}
SCF (DCE) Energy = -1302.70225810
SCF (BP86-D3) Energy = -1302.77971693
SCF (BS2) Energy = -1302.65957142

Rh	-0.41853	-0.62078	-0.13854
C	-2.11657	-0.78284	1.27052
C	-2.18416	-1.85664	0.32818
C	-0.96939	-2.69802	0.49132
C	-0.16453	-2.13096	1.51680
C	-0.80726	-0.87649	1.92968
C	2.27802	1.68754	-0.00730
C	3.11636	2.20082	1.00996
C	1.21023	2.48820	-0.49699
C	2.89770	3.47346	1.55350
H	3.93870	1.57657	1.37677
C	0.99525	3.75759	0.07168
C	1.82358	4.25162	1.09067
H	3.55870	3.85283	2.34047
H	0.15862	4.35888	-0.29835
H	1.63700	5.24450	1.51414
C	3.75488	-0.29826	-0.81703
C	3.40860	-1.62371	-1.18034
H	4.74636	0.15300	-0.78885
N	1.53439	-0.59541	-0.73250
N	2.06686	-1.80262	-1.09729
C	2.53702	0.33684	-0.53519
C	0.37709	2.01014	-1.67021
C	-0.80315	1.05296	-1.36361
H	-0.99978	0.41891	-2.25775
H	1.04578	1.48034	-2.37081
C	-0.31238	0.06284	2.98683
H	-0.66489	-0.26558	3.98430
H	0.78738	0.10162	3.01040
H	-0.68416	1.08430	2.81280
C	1.15331	-2.66695	1.99421
H	1.67664	-1.94049	2.63431
H	1.00562	-3.59100	2.58272
H	1.80537	-2.89922	1.13459
C	-3.18324	0.21632	1.61112
H	-3.56068	0.03272	2.63413
H	-2.81003	1.25339	1.56875
H	-4.03487	0.13625	0.92015
C	-3.32414	-2.15765	-0.59893
H	-4.07225	-2.80342	-0.10083
H	-3.81854	-1.23008	-0.92498
H	-2.97635	-2.68752	-1.50011
C	-0.61315	-3.92041	-0.30209
H	-1.30758	-4.08130	-1.14085
H	0.40689	-3.80647	-0.71145
H	-0.63719	-4.82327	0.33595
H	-0.01403	2.89713	-2.20397
C	-2.05016	1.78389	-1.01767
C	4.29868	-2.74274	-1.64189
H	4.73121	-2.53793	-2.63821
H	5.14399	-2.90200	-0.94916
H	3.72258	-3.67936	-1.71063
O	-3.18361	1.17225	-1.54504
O	-2.13859	2.83243	-0.37783
C	-4.39800	1.93148	-1.36390
H	-5.18553	1.35508	-1.87053
H	-4.64044	2.06001	-0.29600
H	-4.30338	2.93181	-1.81716

TS(E1-F1)$_{2,1}$

SCF Energy = -1302.61705904
Enthalpy 0K = -1302.154316
Energy 298K = -1302.123417
Free Energy 298K = -1302.213861
Lowest Frequency = -331.9510 cm^{-1}
SCF (DCE) Energy = -1302.62903522
SCF (BP86-D3) Energy = -1302.70823251
SCF (BS2) Energy = -1302.58297805

C	-1.92101	1.44497	0.24725
C	-2.08650	2.82617	0.34005
C	-0.99398	3.38161	-0.38566
N	-0.20628	2.40873	-0.88899
N	-0.74753	1.21537	-0.46557
C	-0.67199	4.82831	-0.63336
Rh	0.80772	-0.26285	-0.00818
C	-0.88932	-0.17249	-1.89058
C	-0.14522	-1.40133	-1.57132
O	-0.71741	-1.89339	-0.26584
C	-2.85577	0.34082	0.49933
C	-3.54526	0.15276	1.71077
C	-4.48677	-0.88227	1.83488
C	-4.74418	-1.72880	0.74540
C	-4.06148	-1.54057	-0.47007
C	-3.11601	-0.51390	-0.60481
C	-2.41549	-0.20913	-1.91913
C	2.99253	-0.63185	-0.41754
C	2.73101	0.78087	-0.27551
C	2.31809	1.03514	1.12526
C	2.24587	-0.20345	1.80593
C	2.57964	-1.25286	0.82119
C	3.48233	-1.35978	-1.63502
C	3.03472	1.85810	-1.27657
C	2.06884	2.40327	1.68895
C	1.92832	-0.44049	3.25542
C	2.73282	-2.71221	1.14719
O	0.48028	-2.16072	-2.34420
H	-3.33446	0.81790	2.55499
H	-5.01878	-1.02553	2.78118
H	-4.27567	-2.19262	-1.32499
H	-5.48092	-2.53391	0.83676
H	-2.92212	3.35221	0.80102
H	-0.42396	0.39518	-2.70282
H	-2.77515	0.76669	-2.29828
H	3.72802	-2.90091	1.59429
H	1.98101	-3.05196	1.87843
H	2.64667	-3.33882	0.24597
H	1.30459	-1.33964	3.39440
H	2.84940	-0.58787	3.85303
H	1.38242	0.41087	3.69269
H	3.87374	-0.65735	-2.38802

```
H   4.29241 -2.06635 -1.38138
H   2.65251 -1.93034 -2.10355
H   3.94315  2.42016 -0.98542
H   3.20510  1.43469 -2.27922
H   2.18888  2.56112 -1.34571
H   3.02390  2.93543  1.86105
H   1.47106  3.00589  0.98638
H   1.53132  2.35760  2.64941
H  -2.68851 -0.97096 -2.67664
H  -1.54681  5.37206 -1.03104
H  -0.36445  5.34454  0.29421
H   0.14912  4.91327 -1.36225
C  -0.48652 -3.28728  0.01332
H  -0.23358 -3.38650  1.07882
H  -1.42250 -3.83362 -0.19692
H   0.32197 -3.67220 -0.62815
```

F1$_{2,1}$

SCF Energy = -1302.67177815
Enthalpy 0K = -1302.206721
Energy 298K = -1302.175180
Free Energy 298K = -1302.268490
Lowest Frequency = 18.1326 cm^{-1}
SCF (DCE) Energy = -1302.68059276
SCF (BP86-D3) Energy = -1302.76258296
SCF (BS2) Energy = -1302.63906003

```
C   2.07185 -1.37220 -0.42407
N   1.40122 -0.09459 -0.81862
C   0.41836 -0.14820 -1.88058
C  -0.61859 -1.13744 -1.66950
C  -0.41121 -1.99099 -0.47945
C   0.99739 -2.41334 -0.04276
C  -1.49106 -2.90360 -0.16973
C  -2.62522 -2.99872 -0.95196
C  -2.76092 -2.21212 -2.14553
C  -1.78238 -1.30632 -2.50175
C   0.74990  0.88628 -2.77556
C   1.88255  1.52274 -2.21050
N   2.31237  0.94410 -1.06841
Rh -0.64743  0.10337  0.12513
C  -2.27634  0.32115  1.57250
C  -1.04343  0.63790  2.26900
C  -0.46007  1.75867  1.56665
C  -1.40025  2.22916  0.53903
C  -2.51411  1.34119  0.53814
C  -0.48832 -0.03809  3.49299
C   0.85079  2.42409  1.87886
C  -1.18106  3.43372 -0.33216
C  -3.74055  1.40212 -0.32804
C  -3.27836 -0.72798  1.96397
C   2.61421  2.72469 -2.74197
C   3.02460 -1.07289  0.74211
O   2.67943 -0.76785  1.87572
O   4.31546 -1.24683  0.35935
H  -1.87407 -0.70721 -3.41426
H  -3.64549 -2.33868 -2.77847
H  -1.36956 -3.56325  0.69893
H  -3.41679 -3.70590 -0.68184
H   0.21149  1.16874 -3.67811
H   1.43467  1.83518  2.60164
H   1.46620  2.52948  0.96714
H   0.68917  3.43407  2.30074
H  -1.29900  4.37463  0.23869
H  -0.16514  3.43541 -0.76353
H  -1.89614  3.46114 -1.17023
H  -4.62865  1.71888  0.25144
H  -3.61269  2.11556 -1.15810
H  -3.96755  0.41560 -0.76933
H  -4.12401 -0.27422  2.51533
H  -3.68982 -1.24318  1.07959
H  -2.82660 -1.49355  2.61447
H  -0.73554  0.52311  4.41532
H  -0.90153 -1.05387  3.60771
H   0.60722 -0.13589  3.43167
H   1.27449 -3.38501 -0.49885
H   0.99138 -2.54949  1.05220
H   2.67499 -1.70384 -1.28725
H   2.04178  3.65487 -2.57716
H   3.58752  2.82759 -2.23801
H   2.78372  2.63343 -3.82804
C   5.28922 -0.97857  1.39687
H   6.26370 -1.22445  0.95420
H   5.25181  0.08317  1.68782
H   5.09247 -1.60045  2.28469
```

D2$_{2,1}$

SCF Energy = -1302.69085257
Enthalpy 0K = -1302.227301
Energy 298K = -1302.195305
Free Energy 298K = -1302.289635
Lowest Frequency = 14.2802 cm^{-1}
SCF (DCE) Energy = -1302.70508761
SCF (BP86-D3) Energy = -1302.78639997
SCF (BS2) Energy = -1302.65978021

```
Rh -0.65039 -0.23250 -0.22383
C  -2.87534 -0.68507 -0.06558
C  -2.17295 -1.97090 -0.11310
C  -1.31258 -2.05911  1.02496
C  -1.47443 -0.81854  1.78993
C  -2.48174 -0.00765  1.14606
C   0.84744  2.27486  0.33807
C   1.10965  3.65869  0.41958
C  -0.35080  1.81741 -0.28502
C   0.20258  4.58492 -0.11602
H   2.03192  4.00380  0.90053
C  -1.24015  2.75443 -0.83033
C  -0.97092  4.13694 -0.74269
H   0.41673  5.65711 -0.05203
H  -2.15112  2.41985 -1.34252
H  -1.67634  4.85580 -1.17476
C   2.91185  1.07104  1.55005
C   3.06146 -0.32719  1.71226
H   3.58550  1.85791  1.88771
N   1.21301 -0.04317  0.63012
N   2.01228 -0.99610  1.14512
C   1.70147  1.21784  0.85038
C  -0.70066 -0.03440 -2.38631
C   0.31356 -0.96629 -2.05052
H  -1.67522 -0.38778 -2.74119
H  -0.40060  0.96387 -2.71606
H   0.12330 -2.04491 -2.07998
C   1.75172 -0.58199 -2.18475
C  -0.79195 -0.53635  3.09653
H  -1.31724 -1.05237  3.92209
H   0.24945 -0.89542  3.07141
H  -0.77754  0.54060  3.32293
C  -3.06833  1.26773  1.67705
H  -4.01591  1.05883  2.20807
H  -2.38492  1.76050  2.38474
H  -3.27974  1.98709  0.87055
C  -3.98949 -0.26609 -0.98394
H  -4.95760 -0.66892 -0.63058
H  -4.08523  0.83038 -1.02992
H  -3.84009 -0.63811 -2.01075
C  -2.39728 -3.02682 -1.15877
H  -3.38404 -3.50585 -1.02081
H  -2.38364 -2.60830 -2.18038
H  -1.63225 -3.81664 -1.10776
C  -0.41048 -3.19061  1.42692
H  -0.41267 -3.99699  0.67678
H   0.62714 -2.82040  1.53494
H  -0.73385 -3.62488  2.39068
```

```
C   4.16012 -1.09412  2.39635
H   5.15183 -0.86462  1.96685
H   4.21557 -0.86308  3.47589
H   3.98318 -2.17612  2.28812
O   2.54505 -1.68364 -2.03545
O   2.17715  0.54148 -2.42602
C   3.96166 -1.39814 -2.06003
H   4.23246 -0.77284 -1.19427
H   4.45939 -2.37577 -2.00183
H   4.23778 -0.87141 -2.98765
```

TS(D2-E2)$_{2,1}$

SCF Energy = -1302.67049251
Enthalpy 0K = -1302.207207
Energy 298K = -1302.175934
Free Energy 298K = -1302.268265
Lowest Frequency = -274.9621 cm^{-1}
SCF (DCE) Energy = -1302.68263738
SCF (BP86-D3) Energy = -1302.76303226
SCF (BS2) Energy = -1302.63994297

```
Rh -0.32985 -0.53083 -0.08453
C  -1.78116 -2.23991  0.20751
C  -0.42507 -2.73899  0.30201
C   0.23599 -2.05138  1.38927
C  -0.75621 -1.16324  2.01545
C  -1.99107 -1.29338  1.30158
C  -0.91442  2.39538 -0.33143
C  -1.75031  3.53134 -0.29264
C  -1.40034  1.17771 -0.93083
C  -3.06022  3.47650 -0.78886
H  -1.37313  4.45284  0.16440
C  -2.73245  1.14692 -1.40568
C  -3.56106  2.27675 -1.32921
H  -3.69597  4.36720 -0.74731
H  -3.10786  0.23618 -1.88904
H  -4.58580  2.22829 -1.71314
C   1.33427  3.19304  0.86355
C   2.34297  2.34505  1.36469
H   1.29787  4.28130  0.90119
N   0.87222  1.04057  0.45722
N   2.04056  1.03077  1.11585
C   0.39409  2.31707  0.28391
C  -0.27449  0.36623 -2.25002
C   0.71708 -0.65727 -1.91998
H  -1.06478  0.05449 -2.94042
H   0.18632  1.32834 -2.50748
H   0.54417 -1.68024 -2.27727
C   2.15543 -0.26594 -1.90837
C  -0.47348 -0.31399  3.22071
H  -0.45432 -0.93474  4.13588
H   0.50928  0.17703  3.12099
H  -1.23461  0.46968  3.35641
C  -3.29340 -0.61722  1.61995
H  -4.02578 -1.34625  2.01337
H  -3.16190  0.16958  2.37831
H  -3.73350 -0.14348  0.72571
C  -2.85681 -2.75167 -0.71152
H  -3.38010 -3.61810 -0.26338
H  -3.61611 -1.97842 -0.91001
H  -2.44710 -3.08181 -1.67992
C   0.18327 -3.81971 -0.54820
H   0.09225 -4.80032 -0.04510
H  -0.32260 -3.90510 -1.52383
H   1.25236 -3.63173 -0.73474
C   1.63489 -2.25789  1.89522
H   2.22754 -2.86389  1.19294
H   2.14182 -1.28275  2.01148
H   1.62821 -2.77493  2.87342
C   3.62923  2.70304  2.05514
H   4.38162  3.09333  1.34521
H   3.47532  3.47753  2.82647
H   4.05797  1.81135  2.54026
O   2.94855 -1.35647 -1.63952
O   2.60799  0.85017 -2.13670
C   4.35830 -1.04584 -1.55950
H   4.54364 -0.33719 -0.73699
H   4.86144 -2.00432 -1.36928
H   4.71498 -0.60106 -2.50272
```

E2$_{2,1}$

SCF (BP86) Energy = -1302.69508362
Enthalpy 0K = -1302.230385
Energy 298K = -1302.198568
Free Energy 298K = -1302.292999
Lowest Frequency = 14.1220 cm^{-1}
SCF (DCE) Energy = -1302.70723446
SCF (BP86-D3) Energy = -1302.78711043
SCF (BS2) Energy = -1302.66346189

```
C   2.12640  1.14350  0.36330
C   2.61308  2.45538  0.51784
C   1.46514  3.25456  0.71356
N   0.34341  2.48739  0.66006
N   0.75416  1.20434  0.47248
C   1.36446  4.72948  0.98656
Rh -0.77299 -0.08457 -0.00345
O  -1.18963 -0.11930  2.37333
C  -1.13624 -1.49062  1.97030
O  -2.04951 -2.27090  2.22584
C   2.88063 -0.09615  0.12047
C   3.98035 -0.06805 -0.77059
C   4.75125 -1.21181 -1.01545
C   4.43415 -2.41717 -0.36823
C   3.35591 -2.45408  0.52919
C   2.57482 -1.31272  0.79357
C   1.44638 -1.38181  1.80136
C   0.08467 -1.64713  1.14390
C  -0.69001 -0.49254 -2.08365
C  -1.72569 -1.35914 -1.51495
C  -2.74570 -0.50885 -0.96954
C  -2.41692  0.89118 -1.29614
C  -1.18008  0.89876 -1.99427
C   0.51770 -0.94121 -2.85443
C  -1.74303 -2.86158 -1.51900
C  -3.99879 -0.98169 -0.28688
C  -3.24038  2.09962 -0.95558
C  -0.47124  2.11714 -2.51005
H   4.21528  0.87351 -1.27902
H   5.59518 -1.16291 -1.71208
H   3.12353 -3.38526  1.06094
H   5.02927 -3.31848 -0.54979
H   3.65862  2.76263  0.53469
H   1.39583 -0.44291  2.37756
H   0.06348 -2.61757  0.62559
H  -1.05686  2.59301 -3.31716
H  -0.32918  2.85030 -1.69674
H   0.52099  1.86461 -2.91259
H  -3.95893  2.33309 -1.76442
H  -3.82295  1.94809 -0.03254
H  -2.59583  2.97933 -0.80380
H   0.29394 -0.98459 -3.93788
H   1.36610 -0.25531 -2.70702
H   0.84783 -1.94133 -2.53490
H  -2.36728 -3.23867 -2.35052
H  -0.73170 -3.27741 -1.65045
H  -2.15660 -3.26165 -0.57863
H  -3.78591 -1.79743  0.42334
H  -4.48702 -0.16498  0.26851
H  -4.72796 -1.35519 -1.03030
H   1.65769 -2.20202  2.51763
H   1.74681  4.99026  1.99047
H   1.94453  5.32053  0.25583
H   0.31136  5.04989  0.93478
```

C -2.37478 0.26936 3.10456
H -2.35854 -0.19501 4.10394
H -2.32165 1.36301 3.18597
H -3.28768 -0.04922 2.57752

TS(E2-F2)$_{2,1}$
SCF Energy = -1302.61865778
Enthalpy 0K = -1302.156165
Energy 298K = -1302.124790
Free Energy 298K = -1302.218134
Lowest Frequency = -345.7140 cm^{-1}
SCF (DCE) Energy = -1302.62903522
SCF (BP86-D3) Energy = -1302.70039238
SCF (BS2) Energy = -1302.58297805

Rh 1.03116 -0.10115 -0.32036
C   1.32894 -2.13024 0.40517
C   1.80863 -2.09139 -0.96516
C   2.83645 -1.07870 -1.00919
C   3.11719 -0.63372 0.37807
C   2.21329 -1.30347 1.24549
C -2.82266 -0.64040 0.25419
C -3.42682 -1.88765 0.51222
C -3.26310 0.13518 -0.85751
C -4.43911 -2.37574 -0.32870
H -3.09244 -2.47391 1.37447
C -4.26759 -0.37067 -1.69455
C -4.85808 -1.62105 -1.43529
H -4.89760 -3.34827 -0.12083
H -4.60320 0.22603 -2.55091
H -5.64469 -2.00043 -2.09570
C -1.95632 0.26207 2.52438
C -0.99527 1.28845 2.77398
H -2.70636 -0.13081 3.21010
N -0.78310 0.75484 0.66875
N -0.32082 1.61500 1.65852
C -1.84869 -0.01486 1.16157
C -2.60945 1.49315 -1.07933
C -1.10949 1.30893 -1.03676
H -2.93302 2.21570 -0.30355
H -0.77354 0.38012 -1.56740
C   2.12566 -1.18364 2.74003
H   2.64000 -2.02476 3.24552
H   2.58515 -0.24797 3.09712
H   1.07716 -1.18692 3.08061
C   4.20082 0.33286 0.76252
H   5.18959 -0.16493 0.80107
H   4.27796 1.15757 0.03476
H   4.01115 0.77882 1.75204
C   0.27064 -3.05692 0.93895
H   0.71401 -4.01277 1.27991
H -0.25471 -2.60709 1.79742
H -0.48453 -3.29095 0.17146
C   1.33721 -2.95166 -2.10446
H   1.91199 -3.89816 -2.15987
H   0.27348 -3.22055 -1.99652
H   1.45346 -2.44070 -3.07461
C   3.65066 -0.68697 -2.21187
H   3.09238 -0.85489 -3.14717
H   3.92919 0.37882 -2.17686
H   4.58770 -1.27456 -2.27019
H -2.90478 1.90892 -2.05977
C -0.08571 2.29835 -1.24458
C -0.69985 1.98951 4.06965
H -1.62099 2.39408 4.52497
H -0.24589 1.30472 4.80815
H   0.00035 2.82063 3.89422
O   1.11710 1.88220 -1.12274
O -0.41864 3.59725 -1.43309
C   0.70115 4.50490 -1.52189
H   1.32543 4.27229 -2.40137
H   0.25844 5.50525 -1.62375
H   1.32119 4.44400 -0.61305

F2$_{2,1}$
SCF Energy = -1302.67114114
Enthalpy 0K = -1302.206010
Energy 298K = -1302.174428
Free Energy 298K = -1302.268583
Lowest Frequency = 15.1263 cm^{-1}
SCF (DCE) Energy = -1302.68133820
SCF (BP86-D3) Energy = -1302.76011804
SCF (BS2) Energy = -1302.63932474

C   2.09621 -0.55248 0.91851
N   1.43111 0.34198 -0.06817
C   0.94706 -0.14410 -1.33809
C   0.11681 -1.32900 -1.23819
C   0.09256 -1.93410 0.11103
C   1.37100 -1.92171 0.95523
C -0.79222 -3.06915 0.27114
C -1.54396 -3.57070 -0.77295
C -1.43966 -3.00675 -2.08990
C -0.62438 -1.91788 -2.32404
C   1.30835 0.82438 -2.28964
C   1.97990 1.83627 -1.56185
N   2.09118 1.56477 -0.24242
Rh -0.80983 0.05904 0.15204
C -2.87179 -0.00085 0.86455
C -2.09716 0.70270 1.87744
C -1.53296 1.85612 1.22392
C -2.07545 1.94999 -0.14239
C -2.90758 0.81576 -0.35713
C -1.97669 0.33022 3.33017
C -0.60423 2.87445 1.82507
C -1.78003 3.07144 -1.09789
C -3.69164 0.46552 -1.59000
C -3.71089 -1.22798 1.08411
C   2.55773 3.11789 -2.09610
C   3.57288 -0.71795 0.52470
O   3.96459 -1.13663 -0.55486
O   4.38844 -0.36932 1.55698
H -0.52670 -1.49199 -3.32843
H -2.00537 -3.45323 -2.91435
H -0.82263 -3.56048 1.25215
H -2.20110 -4.43136 -0.60835
H   1.07706 0.81295 -3.35282
H -0.28872 2.58360 2.83952
H   0.30759 2.98741 1.20957
H -1.09148 3.86497 1.89422
H -2.29899 4.00348 -0.80265
H -0.69932 3.29217 -1.13118
H -2.09900 2.82297 -2.12291
H -4.77966 0.57846 -1.42244
H -3.41657 1.11151 -2.43930
H -3.51005 -0.58061 -1.89457
H -4.77530 -0.95568 1.21879
H -3.64467 -1.91961 0.22743
H -3.38990 -1.78057 1.98128
H -2.80487 0.75713 3.92957
H -2.00068 -0.76308 3.47026
H -1.03292 0.69712 3.76595
H   2.05805 -2.70548 0.58071
H   1.12109 -2.17760 1.99872
H   2.03754 -0.02850 1.88365
H   3.20775 2.92178 -2.96575
H   1.76789 3.81425 -2.42909
H   3.15262 3.61714 -1.31606
C   5.80135 -0.51076 1.27176
H   6.31719 -0.24457 2.20409
H   6.03600 -1.54536 0.97438
H   6.09611 0.16868 0.45633

TS(E1-E1′)$_{2,1}$

SCF (BP86) Energy = -1302.68712793
Enthalpy 0K = -1302.222316
Energy 298K = -1302.191434
Free Energy 298K = -1302.282984
Lowest Frequency = -27.2357 cm^{-1}
SCF (DCE) Energy = -1302.69910920
SCF (BP86-D3) Energy = -1302.77751000
SCF (BS2) Energy = -1302.65653126

```
Rh -0.22650 -0.46414  0.08092
C  -2.01545 -1.01461  1.26378
C  -1.54724 -2.10726  0.43437
C  -0.20560 -2.51745  0.92071
C   0.17021 -1.63856  1.98110
C  -0.91261 -0.66608  2.13847
C   1.93274  1.87572 -0.08748
C   2.62488  2.50363  0.97374
C   0.69792  2.42987 -0.54081
C   2.09752  3.63703  1.60705
H   3.57940  2.07758  1.30191
C   0.17319  3.55651  0.12566
C   0.85645  4.15466  1.19436
H   2.64951  4.11201  2.42562
H  -0.78407  3.96210 -0.21828
H   0.43279  5.03418  1.69120
C   3.74222  0.22855 -1.08729
C   3.58158 -1.11906 -1.49280
H   4.64847  0.83365 -1.10687
N   1.61550 -0.43681 -0.85409
N   2.29002 -1.51240 -1.32929
C   2.45800  0.63703 -0.69220
C   0.01627  1.88232 -1.78928
C  -0.93308  0.67453 -1.58279
H  -0.86184 -0.03551 -2.42286
H   0.80354  1.56800 -2.49479
C  -0.91316  0.47271  3.11420
H  -1.16444  0.10544  4.12775
H   0.07601  0.95559  3.17008
H  -1.65274  1.23803  2.83481
C   1.45442 -1.67267  2.75987
H   1.75671 -0.66255  3.07969
H   1.35082 -2.29164  3.67135
H   2.27421 -2.09035  2.15539
C  -3.39302 -0.41665  1.30067
H  -3.89982 -0.67203  2.24956
H  -3.37152  0.68220  1.20732
H  -4.00870 -0.80713  0.47740
C  -2.33776 -2.82195 -0.61900
H  -2.98346 -3.59490 -0.15909
H  -2.97503 -2.11441 -1.17240
H  -1.67427 -3.32219 -1.34160
C   0.62066 -3.62859  0.35252
H  -0.00953 -4.39684 -0.12323
H   1.31965 -3.21472 -0.41113
H   1.22337 -4.10997  1.14011
H  -0.54371  2.70861 -2.26414
C  -2.34534  1.09883 -1.40214
C   4.59757 -2.06737 -2.06589
H   4.95073 -1.74095 -3.06109
H   5.48833 -2.15364 -1.41834
H   4.15362 -3.06941 -2.17820
O  -3.23795  0.19269 -1.96587
O  -2.74883  2.13373 -0.86651
C  -4.61177  0.63603 -1.96359
H  -5.17684 -0.15122 -2.48310
H  -4.99346  0.77129 -0.93814
H  -4.71136  1.59537 -2.49715
```

E1′$_{2,1}$
SCF (BP86) Energy = -1302.68993414
Enthalpy 0K = -1302.224802
Energy 298K = -1302.193117
Free Energy 298K = -1302.286784
Lowest Frequency = 21.8953 cm^{-1}
SCF (DCE) Energy = -1302.70026616
SCF (BP86-D3) Energy = -1302.78387987
SCF (BS2) Energy = -1302.65754740

```
Rh -0.18213 -0.20028  0.21357
C  -1.83019 -0.91015  1.49354
C  -0.64558 -1.77095  1.55653
C   0.42649 -1.01177  2.23441
C  -0.04791  0.30860  2.44908
C  -1.43750  0.39149  1.95011
C   1.86481  1.58046 -0.85348
C   2.57278  2.60172 -0.18006
C   0.49490  1.80981 -1.23591
C   1.93647  3.79549  0.18194
H   3.61974  2.42653  0.08912
C  -0.13348  3.01019 -0.81657
C   0.56854  3.98880 -0.10315
H   2.50108  4.57370  0.70734
H  -1.17963  3.16730 -1.10227
H   0.06895  4.91608  0.19640
C   3.67228 -0.33306 -1.36711
C   3.45702 -1.72442 -1.22605
H   4.58414  0.17581 -1.67878
N   1.56246 -0.78066 -0.75287
N   2.17205 -1.97911 -0.82894
C   2.42618  0.24086 -1.05731
C  -0.19617  0.93926 -2.30865
C  -1.00332 -0.24459 -1.73943
H  -0.75113 -1.21025 -2.19807
H   0.59084  0.54504 -2.97193
C  -2.31947  1.60476  2.04579
H  -2.87696  1.61707  3.00194
H  -1.72416  2.53023  1.99347
H  -3.04701  1.63025  1.21914
C   0.69482  1.45701  3.06730
H   0.68364  2.34485  2.41105
H   0.23095  1.74894  4.02796
H   1.74675  1.19928  3.26310
C  -3.21555 -1.34557  1.12141
H  -3.70976 -1.80834  1.99652
H  -3.82910 -0.48861  0.80281
H  -3.20790 -2.08190  0.30351
C  -0.56097 -3.22615  1.20214
H  -0.60547 -3.85690  2.11109
H  -1.38806 -3.52434  0.53929
H   0.38888 -3.43330  0.67952
C   1.77608 -1.58278  2.55499
H   1.69610 -2.34511  3.35182
H   2.20724 -2.06773  1.66070
H   2.47809 -0.80641  2.89642
H  -0.84918  1.60297 -2.90201
C  -2.46777 -0.00270 -1.75286
C   4.40607 -2.86186 -1.48285
H   4.58285 -3.01780 -2.56311
H   5.39022 -2.68564 -1.01436
H   3.98929 -3.79646 -1.07457
O  -3.17059 -1.15517 -2.05463
O  -3.04497  1.07262 -1.56100
C  -4.59608 -0.97226 -2.18343
H  -4.98815 -1.94188 -2.52171
H  -5.05458 -0.69140 -1.22039
H  -4.82326 -0.18392 -2.91910
```

TS(E1-E1″)$_{2,1}$
SCF (BP86) Energy = -1302.68552250
Enthalpy 0K = -1302.220783
Energy 298K = -1302.189890
Free Energy 298K = -1302.281389
Lowest Frequency = -20.5110 cm^{-1}
SCF (DCE) Energy = -1302.69924696

SCF (BP86-D3) Energy = -1302.77286312
SCF (BS2) Energy = -1302.65423460

```
C  -1.78661  1.80424 -0.03274
C  -1.94625  3.18375  0.13976
C  -0.63612  3.72118  0.18867
N   0.27181  2.73668  0.06571
N  -0.41221  1.54872 -0.07966
C  -0.20890  5.15309  0.33628
Rh  0.87160 -0.02942 -0.13935
C  -0.54563 -1.36489 -0.96768
C  -0.94443 -2.22748  0.19336
O  -0.45597 -3.52834  0.05025
C  -2.89962  0.83779 -0.08404
C  -4.05061  1.13658  0.68579
C  -5.20125  0.34221  0.62944
C  -5.22567 -0.78329 -0.20752
C  -4.08713 -1.09889 -0.96158
C  -2.91931 -0.31446 -0.91153
C  -1.72633 -0.72191 -1.74433
C   2.57456 -1.38713 -0.43340
C   2.78145 -0.12786 -1.13126
C   2.99619  0.93748 -0.13292
C   2.72923  0.38293  1.13915
C   2.42991 -1.05856  0.96186
C   2.56566 -2.75953 -1.04386
C   2.98175  0.04636 -2.60759
C   3.34958  2.36099 -0.43749
C   2.74579  1.11163  2.45079
C   2.19787 -2.02950  2.08123
O  -1.60287 -1.91083  1.18047
H  -4.02227  2.00389  1.35299
H  -6.07237  0.59986  1.24124
H  -4.09668 -1.98054 -1.61418
H  -6.11889 -1.41412 -0.26840
H  -2.89077  3.72428  0.18710
H   0.03576 -1.97562 -1.67780
H  -1.33935  0.14923 -2.30423
H   3.15951 -2.31955  2.54715
H   1.56351 -1.59178  2.86876
H   1.70738 -2.94383  1.71514
H   1.98930  0.70687  3.14251
H   3.73153  1.01486  2.94494
H   2.53206  2.18149  2.30740
H   3.54480 -3.25158 -0.89515
H   1.78298 -3.39738 -0.60304
H   2.38384 -2.71410 -2.12986
H   4.06063  0.01344 -2.85553
H   2.48091 -0.74789 -3.18212
H   2.58934  1.01745 -2.94866
H   3.82530  2.45580 -1.42667
H   2.42082  2.96588 -0.41616
H   4.04528  2.76255  0.31816
H  -2.07363 -1.45203 -2.50268
H  -0.57278  5.77363 -0.50239
H  -0.60596  5.60055  1.26493
H   0.89017  5.22248  0.36270
C  -0.83257 -4.41546  1.12701
H  -0.43239 -5.40181  0.85135
H  -0.40734 -4.08094  2.08848
H  -1.92839 -4.45727  1.23234
```

E1″$_{2,1}$
SCF (BP86) Energy = -1302.68566859
Enthalpy 0K = -1302.220740
Energy 298K = -1302.189079
Free Energy 298K = -1302.282902
Lowest Frequency = 18.1465 cm^{-1}
SCF (DCE) Energy = -1302.69912645
SCF (BP86-D3) Energy = -1302.77310018
SCF (BS2) Energy = -1302.65453270

```
C   1.81072  1.74625  0.01426
C   1.97897  3.11959 -0.19530
C   0.67377  3.65574 -0.32644
N  -0.24167  2.67812 -0.20917
N   0.43340  1.49419  0.01298
C   0.25906  5.08088 -0.55283
Rh -0.88879 -0.04165  0.16805
C   0.53544 -1.42583  0.89692
C   0.95881 -2.20084 -0.31368
O   0.39717 -3.48180 -0.31337
C   2.92443  0.78596  0.14009
C   4.11370  1.09257 -0.56689
C   5.26764  0.31041 -0.44816
C   5.25808 -0.81424  0.38960
C   4.08243 -1.14046  1.07922
C   2.91048 -0.36861  0.96593
C   1.68265 -0.79917  1.73504
C  -2.61497 -1.39893  0.36648
C  -2.77093 -0.23442  1.21289
C  -2.95894  0.95315  0.35271
C  -2.77099  0.54755 -0.98938
C  -2.49726 -0.90728 -0.98812
C  -2.61478 -2.83663  0.80197
C  -2.92553 -0.23377  2.70504
C  -3.24335  2.34924  0.81701
C  -2.80391  1.45100 -2.18697
C  -2.30695 -1.75031 -2.21349
O   1.68354 -1.83773 -1.23554
H   4.11372  1.95568 -1.23973
H   6.16706  0.57647 -1.01363
H   4.06388 -2.02379  1.72926
H   6.15212 -1.43724  0.49915
H   2.92434  3.66006 -0.21706
H  -0.04963 -2.09083  1.55347
H   1.27404  0.05691  2.30345
H  -3.28608 -1.99569 -2.66873
H  -1.70657 -1.22563 -2.97391
H  -1.79946 -2.69496 -1.96687
H  -2.36423  0.96710 -3.07301
H  -3.84323  1.73374 -2.43998
H  -2.23221  2.37032 -1.97673
H  -3.58751 -3.30855  0.56991
H  -1.81730 -3.41200  0.30483
H  -2.45872 -2.92550  1.88909
H  -3.99751 -0.25128  2.98180
H  -2.44527 -1.11200  3.16311
H  -2.48077  0.67012  3.15118
H  -3.40695  2.39396  1.90485
H  -2.38154  2.99209  0.55574
H  -4.14515  2.74775  0.31884
H   2.00109 -1.54599  2.48957
H   0.58933  5.73548  0.27371
H   0.69781  5.48743 -1.48153
H  -0.83759  5.15148 -0.62997
C   0.76766 -4.27930 -1.45910
H   0.30748 -5.26456 -1.29537
H   0.39602 -3.83035 -2.39606
H   1.86299 -4.37103 -1.53377
```

TS(E2-E2′)$_{2,1}$
SCF (BP86) Energy = -1302.68835420
Enthalpy 0K = -1302.223514
Energy 298K = -1302.192752
Free Energy 298K = -1302.283395
Lowest Frequency = -12.3557 cm^{-1}
SCF (DCE) Energy = -1302.70035824
SCF (BP86-D3) Energy = -1302.77540140
SCF (BS2) Energy = -1302.65801673

```
Rh -0.62471 -0.34949  0.07931
C  -1.87492 -0.86038  1.74752
C  -2.68751 -0.06338  0.84159
```

C -2.71295 -0.76424 -0.41620
C -2.05283 -2.07806 -0.22873
C -1.56559 -2.14556 1.10424
C   3.02836 0.64190 0.10493
C   4.17133 0.71874 0.93835
C   2.45435 1.84397 -0.38505
C   4.74247 1.94971 1.28140
H   4.60066 -0.21167 1.32477
C   3.04137 3.07404 -0.02809
C   4.17602 3.13838 0.79309
H   5.62413 1.98087 1.93046
H   2.59734 3.99858 -0.41635
H   4.61393 4.10881 1.05002
C   3.35060 -1.80133 -0.56908
C   2.45198 -2.85469 -0.86904
H   4.43963 -1.82177 -0.59118
N   1.21491 -1.09458 -0.41572
N   1.17828 -2.42698 -0.75728
C   2.54552 -0.69170 -0.29077
C   1.20937 1.82301 -1.23858
C -0.05127 1.66409 -0.33488
H   1.25869 1.01894 -1.99215
C -0.82433 -3.30014 1.71113
H -1.51310 -4.13916 1.92335
H -0.04445 -3.65177 1.01481
H -0.33969 -3.01780 2.65889
C -1.90223 -3.15380 -1.25931
H -2.39844 -4.08230 -0.92265
H -2.33984 -2.86263 -2.22610
H -0.82354 -3.35840 -1.39833
C -1.55977 -0.50361 3.16951
H -2.32028 -0.93540 3.84897
H -0.57751 -0.89704 3.47599
H -1.55613 0.58735 3.31710
C -3.46059 1.16958 1.20946
H -4.32079 0.89375 1.84830
H -2.84874 1.90458 1.75440
H -3.85906 1.66997 0.31462
C -3.42419 -0.32554 -1.66039
H -3.30262 0.75523 -1.83340
H -3.02968 -0.84420 -2.54772
H -4.50425 -0.55719 -1.58604
H   1.14624 2.77476 -1.79941
C   2.75597 -4.26396 -1.28953
H   3.27733 -4.29338 -2.26331
H   3.40849 -4.77353 -0.55839
H   1.82339 -4.84247 -1.38529
C -1.18355 2.45068 -0.90838
O -1.68531 3.36611 0.00549
O -1.62066 2.39717 -2.05937
H   0.17644 2.06298 0.67145
C -2.70815 4.23568 -0.52331
H -2.94705 4.93922 0.28687
H -2.34080 4.77537 -1.41109
H -3.60972 3.66969 -0.81463

E2′$_{2,1}$

SCF (BP86) Energy = -1302.68979554
Enthalpy 0K = -1302.224334
Energy 298K = -1302.192992
Free Energy 298K = -1302.285601
Lowest Frequency = 16.7967 cm^{-1}
SCF (DCE) Energy = -1302.70071218
SCF (BP86-D3) Energy = -1302.77701886
SCF (BS2) Energy = -1302.65909737

Rh -0.76665 0.22761 -0.06907
C -2.61039 0.38677 -1.21708
C -2.81930 -0.59797 -0.17222
C -2.57494 0.07098 1.08367
C -2.37773 1.51406 0.80271
C -2.44003 1.70888 -0.60543
C   3.15385 0.04653 -0.09439
C   4.38949 0.12490 -0.78543
C   2.83910 -1.16012 0.58961
C   5.30268 -0.93581 -0.78265
H   4.62128 1.03812 -1.34279
C   3.77113 -2.21506 0.58806
C   4.99873 -2.11347 -0.08203
H   6.24636 -0.84342 -1.33072
H   3.51815 -3.13632 1.12608
H   5.70481 -2.95050 -0.06695
C   2.85454 2.56582 -0.02075
C   1.74121 3.43588 0.04893
H   3.91045 2.83293 -0.00798
N   0.92962 1.38877 0.00709
N   0.59831 2.72423 0.06141
C   2.32574 1.26909 -0.04186
C   1.48744 -1.34414 1.23536
C   0.42046 -1.51339 0.11008
H   1.25532 -0.48791 1.89255
C -2.29952 3.02087 -1.31807
H -3.14290 3.69170 -1.07102
H -1.35662 3.50468 -1.00738
H -2.28435 2.88995 -2.41138
C -2.14935 2.59562 1.81372
H -3.02441 3.27046 1.85905
H -1.98423 2.18647 2.82219
H -1.26323 3.18472 1.51846
C -2.70607 0.12344 -2.69033
H -3.74111 0.29512 -3.04416
H -2.04312 0.79242 -3.26164
H -2.43476 -0.91638 -2.92926
C -3.33496 -1.99395 -0.35591
H -4.44126 -1.98323 -0.37330
H -2.98628 -2.43437 -1.30251
H -3.01378 -2.65164 0.46635
C -2.65199 -0.56551 2.43875
H -2.21647 -1.57881 2.41618
H -2.10575 0.02562 3.19057
H -3.70495 -0.64313 2.77209
H   1.48683 -2.24140 1.87862
C   1.71753 4.93595 0.11520
H   2.28117 5.31190 0.98767
H   2.17285 5.38963 -0.78341
H   0.68002 5.29785 0.19444
C -0.35746 -2.76901 0.27332
O -0.54460 -3.41811 -0.93551
O -0.77548 -3.25230 1.33093
H   0.91331 -1.53440 -0.88005
C -1.14670 -4.72515 -0.83140
H -1.08944 -5.15593 -1.84128
H -0.59575 -5.35141 -0.11123
H -2.19832 -4.66388 -0.50489

TS(E2-E2″)$_{2,1}$

SCF (BP86) Energy = -1302.69484478
Enthalpy 0K = -1302.230323
Energy 298K = -1302.199268
Free Energy 298K = -1302.291548
Lowest Frequency = -38.9993 cm^{-1}
SCF (DCE) Energy = -1302.70726719
SCF (BP86-D3) Energy = -1302.78568813
SCF (BS2) Energy = -1302.66321748

C   2.16728 1.07377 0.47008
C   2.67757 2.35848 0.72724
C   1.54187 3.17407 0.93735
N   0.40549 2.44379 0.79378
N   0.79196 1.16098 0.54153
C   1.46968 4.62943 1.30551
Rh -0.75341 -0.03909 -0.05451
C   0.04684 -1.72099 0.94578
H   0.02360 -2.61742 0.30781

C   2.90114 -0.15667 0.13374
C   4.01989 -0.06947 -0.72958
C   4.77297 -1.20181 -1.06557
C   4.41762 -2.45503 -0.54077
C   3.31875 -2.55204 0.32636
C   2.55484 -1.42363 0.68155
C   1.40214 -1.56227 1.65245
C -1.87079 -1.14938 -1.58870
C -2.79004 -0.25042 -0.95236
C -2.36098 1.13805 -1.21309
C -1.15378 1.08731 -1.95698
C -0.78628 -0.33510 -2.14523
C -2.01279 -2.64360 -1.66472
C -4.04406 -0.65978 -0.23445
C -3.05548 2.38930 -0.75924
C -0.36413 2.27283 -2.42898
C   0.35813 -0.83056 -2.97946
H   4.28368 0.90973 -1.14393
H   5.63243 -1.10643 -1.73795
H   3.05455 -3.52368 0.76204
H   4.99819 -3.34839 -0.79447
H   3.72853 2.63946 0.79202
C -1.17088 -1.69086 1.79731
H   1.36331 -0.68629 2.32103
H   0.09740 -0.79165 -4.05494
H   1.26141 -0.22040 -2.82473
H   0.61595 -1.87093 -2.72983
H   0.61022 1.97090 -2.84128
H -0.91577 2.81527 -3.21820
H -0.17836 2.96597 -1.59020
H -2.76179 -2.92459 -2.42820
H -1.06181 -3.12501 -1.94197
H -2.33590 -3.06198 -0.69650
H -4.85341 -0.86472 -0.96069
H -3.87999 -1.57035 0.36380
H -4.40156 0.13555 0.43900
H -3.71261 2.19908 0.10410
H -2.31804 3.15045 -0.45793
H -3.68279 2.81268 -1.56665
H   1.57706 -2.45565 2.28640
H   1.81262 4.80955 2.34083
H   2.10029 5.24874 0.64336
H   0.42972 4.98566 1.22951
O -1.18539 -0.49060 2.53360
O -2.10108 -2.49788 1.85949
C -2.34151 -0.30393 3.37824
H -2.22529 0.69660 3.81594
H -3.27418 -0.37199 2.79588
H -2.36204 -1.07270 4.16801

E2″$_{2,1}$
SCF (BP86) Energy = -1302.69667785
Enthalpy 0K = -1302.231546
Energy 298K = -1302.199976
Free Energy 298K = -1302.293458
Lowest Frequency = 21.7376 cm^{-1}
SCF (DCE) Energy = -1302.70828840
SCF (BP86-D3) Energy = -1302.78426496
SCF (BS2) Energy = -1302.66580271

C   2.28967 0.77826 0.70752
C   2.90685 1.89014 1.29321
C   1.84628 2.75908 1.65818
N   0.65674 2.23089 1.29137
N   0.92017 1.00300 0.73655
C   1.90911 4.07648 2.37676
Rh -0.66025 0.13469 -0.19857
C -0.09088 -1.84130 0.38195
H -0.12346 -2.42556 -0.55324
C   2.92578 -0.37179 0.04042
C   4.07005 -0.14074 -0.75986
C   4.72840 -1.19247 -1.40992
C   4.24736 -2.50454 -1.27202
C   3.12074 -2.74550 -0.47136
C   2.45119 -1.70208 0.19685
C   1.27075 -2.00460 1.09008
C -2.19169 -0.36178 -1.70905
C -2.82070 0.33110 -0.62147
C -2.25567 1.70122 -0.54170
C -1.26484 1.83116 -1.54866
C -1.15102 0.52614 -2.22696
C -2.54749 -1.72867 -2.22060
C -3.95689 -0.18775 0.20645
C -2.61799 2.76390 0.45296
C -0.43759 3.05586 -1.81079
C -0.23933 0.23161 -3.37955
H   4.42827 0.88795 -0.87545
H   5.61081 -0.98734 -2.02557
H   2.75806 -3.77210 -0.33739
H   4.75206 -3.33640 -1.77496
H   3.97539 2.03220 1.45266
C -1.25559 -2.26779 1.21422
H   1.29887 -1.35478 1.98045
H -0.69773 0.58024 -4.32559
H   0.72962 0.74234 -3.26620
H -0.04546 -0.84749 -3.47532
H   0.42494 2.83208 -2.45719
H -1.04151 3.83423 -2.31304
H -0.05627 3.46782 -0.86118
H -3.45329 -1.68165 -2.85360
H -1.73620 -2.14957 -2.83589
H -2.73340 -2.42064 -1.38228
H -4.91496 -0.02798 -0.32466
H -3.84156 -1.26606 0.40080
H -4.02306 0.33622 1.17331
H -3.35839 2.40515 1.18407
H -1.70810 3.07448 0.99925
H -3.04270 3.64894 -0.05531
H   1.36367 -3.04793 1.45413
H   2.24173 3.95468 3.42366
H   2.61799 4.77029 1.89105
H   0.91333 4.54744 2.39077
O -1.09320 -1.94362 2.54373
O -2.27132 -2.85725 0.81975
C -2.17513 -2.35272 3.40452
H -1.85122 -2.09756 4.42318
H -3.10463 -1.81458 3.15434
H -2.36200 -3.43513 3.31467

TS(E2-E1″)$_{2,1}$
SCF (BP86) Energy = -1302.67676545
Enthalpy 0K = -1302.212510
Energy 298K = -1302.181305
Free Energy 298K = -1302.274231
Lowest Frequency = -21.8153 cm^{-1}
SCF (DCE) Energy = -1302.69083658
SCF (BP86-D3) Energy = -1302.76145095
SCF (BS2) Energy = -1302.64644609

Rh -0.91910 -0.09845 -0.26495
C -2.34817 -0.13510 -1.86382
C -2.67840 -1.22537 -0.95154
C -2.95629 -0.62950 0.32473
C -2.94197 0.84571 0.15994
C -2.60296 1.14438 -1.18435
C   2.97395 0.34202 -0.41560
C   4.27693 0.89991 -0.55002
C   2.80458 -1.01732 -0.81971
C   5.38246 0.17578 -0.99887
H   4.41817 1.95561 -0.30873
C   3.94214 -1.73030 -1.26441
C   5.21963 -1.16958 -1.35214
H   6.35921 0.66512 -1.07492
H   3.80550 -2.77914 -1.55459

H   6.06589 -1.77187 -1.69876
C   2.26599 2.48667 0.78356
C   1.02882 3.13517 1.01326
H   3.24892 2.85977 1.06377
N   0.57245 1.21408 0.05393
N   0.01673 2.37981 0.55456
C   1.96376 1.27029 0.15153
C   1.50273 -1.79957 -0.95420
C   0.36505 -1.73144 0.07749
H   1.79640 -2.86507 -1.04810
H -0.30341 -2.59139 -0.09638
C -2.44204 2.52235 -1.75839
H -3.41871 3.03470 -1.83746
H -1.78239 3.12199 -1.10710
H -1.99723 2.49368 -2.76525
C -3.20660 1.86216 1.22917
H -4.10474 2.45889 0.98512
H -3.36698 1.39283 2.21174
H -2.33886 2.54192 1.30043
C -1.98602 -0.28575 -3.31162
H -2.89504 -0.23116 -3.94167
H -1.30323 0.51369 -3.64022
H -1.49975 -1.25389 -3.50844
C -2.73422 -2.68661 -1.29503
H -3.72892 -2.95664 -1.69603
H -1.98549 -2.95627 -2.05796
H -2.55242 -3.31684 -0.40968
C -3.36003 -1.35148 1.57569
H -2.87597 -2.33685 1.64393
H -3.07454 -0.78237 2.47401
H -4.45749 -1.49531 1.60138
H   1.06688 -1.54527 -1.94215
C   0.77937 -1.76500 1.51332
C   0.76936 4.45924 1.67187
H   1.06269 4.44677 2.73709
H   1.34107 5.27105 1.18816
H -0.30215 4.70894 1.61487
C   0.09542 -2.36878 3.70394
H -0.75464 -2.87172 4.18747
H   1.02905 -2.92332 3.89381
H   0.21401 -1.34573 4.09902
O -0.21486 -2.34552 2.29590
O   1.82594 -1.36149 2.00927

TS(E2′-E_{trans})
SCF Energy = -1302.68785221
Enthalpy 0K = -1302.223385
Energy 298K = -1302.192549
Free Energy 298K = -1302.283820
Lowest Frequency = -64.6787 cm^{-1}
SCF (DCE) Energy = -1302.69900264
SCF (BP86-D3) Energy = -1302.77437803
SCF (BS2) Energy = -1302.65768919

Rh -0.76179 0.21608 0.14569
C -2.68226 0.05117 -0.82322
C -2.80626 -0.56441 0.47636
C -2.49853 0.46378 1.45961
C -2.33226 1.74584 0.79050
C -2.40421 1.48946 -0.61784
C   3.15236 -0.00357 0.04418
C   4.51200 0.02917 -0.36555
C   2.70431 -1.16651 0.73441
C   5.40460 -1.00550 -0.06965
H   4.86686 0.88652 -0.94432
C   3.61841 -2.19265 1.04169
C   4.96310 -2.12386 0.65439
H   6.44357 -0.93854 -0.40961
H   3.25072 -3.07314 1.58137
H   5.64988 -2.94148 0.89670
C   2.88398 2.48413 -0.43081
C   1.78580 3.35287 -0.57977
H   3.93894 2.75325 -0.43055
N   0.94747 1.35218 -0.21578
N   0.63203 2.66219 -0.45027
C   2.33920 1.20517 -0.21169
C   1.24788 -1.37128 1.06761
C   0.40127 -1.49449 -0.22197
H   0.89589 -0.52338 1.71103
C -2.26069 2.52717 -1.68563
H -3.06376 3.28122 -1.59222
H -1.28351 3.03015 -1.55553
H -2.31159 2.09251 -2.69531
C -2.08327 3.08366 1.42292
H -2.97059 3.73784 1.33105
H -1.85067 2.98748 2.49539
H -1.22904 3.57363 0.92520
C -2.94537 -0.60598 -2.14513
H -4.03291 -0.64928 -2.34663
H -2.47233 -0.04787 -2.96793
H -2.54135 -1.63014 -2.16462
C -3.28560 -1.95506 0.78058
H -4.27404 -1.91584 1.27476
H -3.39878 -2.54243 -0.14322
H -2.59054 -2.51101 1.43012
C -2.47397 0.24029 2.94318
H -2.19246 -0.79605 3.18691
H -1.76973 0.92209 3.44575
H -3.47829 0.42431 3.37074
H   1.09255 -2.27661 1.67932
C   1.77219 4.83305 -0.83585
H   2.30856 5.38879 -0.04603
H   2.25772 5.08539 -1.79568
H   0.73489 5.20301 -0.87066
C -0.32155 -2.78315 -0.32557
O -0.65121 -3.08541 -1.63656
O -0.60738 -3.54835 0.60036
H   0.98029 -1.30056 -1.13668
C -1.26208 -4.38166 -1.80905
H -1.43756 -4.48326 -2.88944
H -0.59159 -5.18030 -1.45155
H -2.21271 -4.45622 -1.25469

E_{trans}
SCF Energy = -1302.70139671
Enthalpy 0K = -1302.236849
Energy 298K = -1302.205811
Free Energy 298K = -1302.297082
Lowest Frequency = 31.9645 cm^{-1}
SCF (DCE) Energy = -1302.71162671
SCF (BP86-D3) Energy = -1302.78846925
SCF (BS2) Energy = -1302.67091729

C -2.39722 0.87566 0.42279
C -3.05029 1.91057 1.13220
C -2.03866 2.81121 1.49836
N -0.83518 2.36736 1.04330
N -1.05129 1.20083 0.39216
C -2.13243 4.09834 2.26896
Rh 0.62178 0.19102 -0.33487
C -0.18730 -1.41248 0.79094
C   0.76965 -2.45983 1.20192
O   0.92104 -2.47739 2.56698
C -3.03101 -0.29285 -0.20732
C -4.44098 -0.27707 -0.37929
C -5.13496 -1.34559 -0.95303
C -4.43723 -2.47964 -1.40043
C -3.04469 -2.51259 -1.26183
C -2.33635 -1.45255 -0.66172
C -0.83967 -1.59356 -0.55228
C   2.81732 0.10049 0.01154
C   2.54536 -0.21365 -1.37012
C   1.94965 0.97389 -1.99829
C   1.81255 1.98453 -0.99891

C	2.30265	1.43411	0.27564
C	3.57027	-0.76996	0.97442
C	2.93000	-1.48831	-2.07015
C	1.60731	1.10633	-3.45667
C	1.27812	3.37484	-1.16130
C	2.38137	2.20793	1.55699
O	1.37784	-3.24173	0.46109
H	-4.99601	0.61251	-0.06915
H	-6.22304	-1.28668	-1.06300
H	-2.48251	-3.38728	-1.61030
H	-4.96724	-3.32169	-1.85659
H	-4.11025	1.97626	1.37347
H	2.74624	-2.36203	-1.42388
H	2.35162	-1.62663	-2.99793
H	4.00092	-1.47793	-2.34783
H	1.25305	0.15352	-3.88150
H	0.82240	1.86070	-3.62070
H	2.49733	1.41415	-4.03738
H	0.82429	3.52652	-2.15299
H	0.51328	3.56366	-0.38573
H	2.08973	4.11609	-1.03934
H	3.12562	3.02305	1.47989
H	1.39054	2.64897	1.77078
H	2.66829	1.56194	2.40148
H	4.65570	-0.57397	0.88868
H	3.27385	-0.57193	2.01630
H	3.40100	-1.83753	0.76195
H	-0.47516	-0.75125	-1.36038
H	-0.46178	-2.50495	-1.04423
H	-0.82077	-1.04373	1.60382
H	-1.12349	4.50314	2.44878
H	-2.71435	4.86454	1.72506
H	-2.62189	3.95334	3.24832
C	1.80071	-3.50786	3.06455
H	1.74521	-3.43585	4.15959
H	1.47011	-4.50241	2.72367
H	2.83658	-3.34626	2.72349

TS(E-G)$_{trans}$

SCF Energy = -1302.69754292
Enthalpy 0K = -1302.235674
Energy 298K = -1302.204820
Free Energy 298K = -1302.295655
Lowest Frequency = -576.7817 cm^{-1}
SCF (DCE) Energy = -1302.70721535
SCF (BP86-D3) Energy = -1302.78489145
SCF (BS2) Energy = -1302.66655286

C	-2.43155	0.78004	0.48498
C	-3.11158	1.77926	1.21937
C	-2.12778	2.71868	1.56752
N	-0.91720	2.32941	1.07697
N	-1.10561	1.16051	0.42742
C	-2.25347	3.99431	2.35268
Rh	0.54274	0.18937	-0.37977
C	-0.12500	-1.41460	0.89834
C	0.92942	-2.36121	1.32066
O	1.09006	-2.32463	2.68275
C	-2.99886	-0.41046	-0.15735
C	-4.40078	-0.46546	-0.36803
C	-5.01638	-1.55568	-0.98952
C	-4.24155	-2.63491	-1.44720
C	-2.85473	-2.60008	-1.26203
C	-2.22348	-1.52154	-0.60696
C	-0.74276	-1.62049	-0.40906
C	2.80377	0.24132	-0.04660
C	2.50472	-0.17930	-1.40410
C	1.83002	0.92720	-2.07236
C	1.65133	1.98445	-1.11272
C	2.25135	1.55587	0.15601
C	3.63151	-0.53893	0.93246
C	2.96335	-1.46378	-2.03903
C	1.46180	0.98185	-3.53050
C	1.03713	3.33407	-1.33941
C	2.33155	2.40906	1.38718
O	1.58642	-3.11650	0.59652
H	-5.00982	0.38717	-0.05398
H	-6.10216	-1.55518	-1.13266
H	-2.23633	-3.43565	-1.61109
H	-4.70980	-3.48910	-1.94630
H	-4.16773	1.79599	1.48548
H	2.81483	-2.31435	-1.35402
H	2.40439	-1.67389	-2.96509
H	4.03626	-1.41340	-2.30455
H	1.20977	-0.01456	-3.92599
H	0.59862	1.64233	-3.70550
H	2.30929	1.37091	-4.12558
H	0.49484	3.38044	-2.29661
H	0.32985	3.55900	-0.52164
H	1.81858	4.11679	-1.35170
H	2.99223	3.28057	1.22110
H	1.31936	2.77461	1.64088
H	2.72580	1.84295	2.24577
H	4.70437	-0.32198	0.77160
H	3.38778	-0.27454	1.97319
H	3.48572	-1.62308	0.80268
H	-0.49344	-0.45081	-1.42215
H	-0.27836	-2.43439	-0.98377
H	-0.73458	-1.01088	1.71166
H	-1.26231	4.46061	2.47166
H	-2.91549	4.72391	1.85214
H	-2.66951	3.81579	3.36025
C	2.04043	-3.27772	3.20582
H	1.98268	-3.17769	4.29841
H	1.77789	-4.30174	2.89455
H	3.06113	-3.05374	2.85507

G$_{trans}$

SCF (BP86) Energy = -1302.70422939
Enthalpy 0K = -1302.240791
Energy 298K = -1302.209679
Free Energy 298K = -1302.300869
Lowest Frequency = 32.6743 cm^{-1}
SCF (DCE) Energy = -1302.71384460
SCF (BP86-D3) Energy = -1302.79200745
SCF (BS2) Energy = -1302.67195316

C	-2.43847	0.71937	0.55012
C	-3.12269	1.70453	1.29797
C	-2.15641	2.68113	1.59820
N	-0.95176	2.32702	1.06608
N	-1.13212	1.14289	0.44795
C	-2.29465	3.95919	2.37728
Rh	0.44610	0.18438	-0.45878
C	-0.08321	-1.36521	0.99837
C	1.03163	-2.20835	1.49088
O	1.72008	-2.99788	0.83851
C	-2.96469	-0.48814	-0.09527
C	-4.35729	-0.56922	-0.34229
C	-4.93203	-1.66039	-1.00229
C	-4.11727	-2.70870	-1.46118
C	-2.73826	-2.65091	-1.22889
C	-2.14263	-1.57451	-0.53330
C	-0.68459	-1.67701	-0.26569
C	2.78064	0.32271	-0.06322
C	2.49013	-0.26840	-1.36928
C	1.79197	0.72136	-2.15237
C	1.55895	1.88258	-1.30084
C	2.21400	1.63446	-0.01775
C	3.64648	-0.31610	0.98305
C	3.00439	-1.59613	-1.85266
C	1.47081	0.62268	-3.62013
C	0.92330	3.18855	-1.68606
C	2.26897	2.62158	1.10962

O   1.18860 -2.03932 2.84301
H -4.99125 0.26665 -0.03103
H -6.01298 -1.68181 -1.17663
H -2.09420 -3.47006 -1.57034
H -4.55018 -3.56119 -1.99437
H -4.16955 1.69106 1.59917
H   2.83848 -2.37999 -1.09592
H   2.49931 -1.90563 -2.78132
H   4.08897 -1.54287 -2.06548
H   1.23371 -0.41024 -3.91810
H   0.61372 1.25745 -3.89201
H   2.33958 0.95435 -4.21908
H   0.32495 3.09340 -2.60558
H   0.25958 3.53428 -0.87575
H   1.69378 3.96300 -1.86067
H   2.80840 3.53653 0.80299
H   1.23660 2.90131 1.39638
H   2.78114 2.20326 1.99012
H   3.53484 -1.41191 0.97665
H   4.70992 -0.08983 0.77681
H   3.41429 0.05687 1.99253
H -0.58719 0.14563 -1.61997
H -0.67846 -0.89465 1.78557
H -1.33454 4.49958 2.38458
H -3.05930 4.62625 1.94056
H -2.58717 3.77178 3.42615
H -0.17924 -2.47018 -0.83271
C   2.16629 -2.90979 3.45365
H   2.09396 -2.71768 4.53289
H   1.94197 -3.96511 3.22967
H   3.18198 -2.68060 3.09211

TS(E1″-E$_{cis}$)
SCF Energy = -1302.67856799
Enthalpy 0K = -1302.214416
Energy 298K = -1302.183488
Free Energy 298K = -1302.274988
Lowest Frequency = -50.9603 cm^{-1}
SCF (DCE) Energy = -1302.69300543
SCF (BP86-D3) Energy = -1302.76441709
SCF (BS2) Energy = -1302.64748624

C -1.92032 1.47737 0.05833
C -2.17694 2.80261 0.45799
C -0.94092 3.33172 0.87398
N   0.02837 2.40354 0.72417
N -0.54424 1.27484 0.21330
C -0.62669 4.69420 1.42303
Rh 0.90220 -0.09507 -0.33032
C -0.45501 -1.67385 -0.23726
C -1.04130 -1.87243 1.11637
O -0.28338 -2.81864 1.81342
C -2.94880 0.54411 -0.44255
C -4.30502 0.90173 -0.22269
C -5.37189 0.13585 -0.70015
C -5.11938 -1.03832 -1.42451
C -3.78987 -1.42271 -1.63552
C -2.70477 -0.66822 -1.14789
C -1.31837 -1.19551 -1.42573
C   2.67159 -1.33925 -0.10910
C   2.70859 -0.71686 -1.40275
C   2.89903 0.74119 -1.22047
C   2.93575 1.01500 0.16292
C   2.65119 -0.25175 0.87060
C   2.70212 -2.80825 0.20414
C   2.73894 -1.41325 -2.73392
C   3.06031 1.72502 -2.34384
C   3.12720 2.33373 0.83823
C   2.63193 -0.41104 2.36158
O -2.00861 -1.33169 1.63705
H -4.52535 1.79619 0.36503
H -6.39997 0.45552 -0.49891
H -3.57562 -2.34704 -2.18597
H -5.94105 -1.65162 -1.80846
H -3.13090 3.32554 0.42502
H   0.12255 -2.57151 -0.50550
H -0.74882 -0.39763 -1.98022
H   3.66278 -0.50833 2.75553
H   2.16598 0.46309 2.84278
H   2.06824 -1.31048 2.65308
H   3.78327 2.23392 1.71997
H   3.57173 3.07587 0.15663
H   2.13082 2.70347 1.15845
H   3.71294 -3.10536 0.54031
H   1.98139 -3.06780 0.99670
H   2.46007 -3.41641 -0.68250
H   3.78112 -1.53604 -3.08617
H   2.28530 -2.41515 -2.68022
H   2.19932 -0.83650 -3.50310
H   2.32981 1.54026 -3.14912
H   2.91688 2.75871 -1.99544
H   4.06828 1.64967 -2.79417
H -1.38486 -2.02088 -2.16001
H -0.96239 5.49544 0.74094
H -1.12288 4.86712 2.39522
H   0.45946 4.80385 1.57271
C -0.74257 -3.04461 3.16244
H -0.08874 -3.82904 3.57053
H -0.66673 -2.12489 3.76655
H -1.79344 -3.37657 3.16939

E$_{cis}$
SCF Energy = -1302.68778659
Enthalpy 0K = -1302.224079
Energy 298K = -1302.192775
Free Energy 298K = -1302.285080
Lowest Frequency = 28.3466 cm^{-1}
SCF (DCE) Energy = -1302.70160062
SCF (BP86-D3) Energy = -1302.77443598
SCF (BS2) Energy = -1302.65782633

C -1.96745 1.07955 -0.59696
C -2.41905 2.41917 -0.63633
C -1.28318 3.20644 -0.40007
N -0.19214 2.40938 -0.23622
N -0.60703 1.12629 -0.35599
C -1.15278 4.70120 -0.30926
Rh 0.85762 -0.34137 -0.24109
C -0.29715 -1.33528 1.21197
C -1.00660 -0.54857 2.24854
O -2.12291 -0.04707 2.20166
C -2.78495 -0.12789 -0.76523
C -4.11632 0.02878 -1.23221
C -4.98741 -1.05163 -1.39145
C -4.55051 -2.35484 -1.10455
C -3.23299 -2.54050 -0.67134
C -2.35248 -1.45658 -0.47974
C -0.94297 -1.80581 -0.06535
C   2.86116 -0.62186 0.64407
C   2.76609 -1.43967 -0.54210
C   2.60963 -0.54932 -1.70039
C   2.56560 0.79416 -1.22657
C   2.66923 0.76170 0.24266
C   3.14299 -1.09241 2.04404
C   2.95606 -2.93050 -0.61811
C   2.56848 -0.99706 -3.13555
C   2.44026 2.05273 -2.02883
C   2.72568 1.97795 1.11659
O -0.19891 -0.45254 3.37284
H -4.46753 1.03286 -1.48404
H -6.00700 -0.87583 -1.75076
H -2.87080 -3.55412 -0.45903
H -5.21852 -3.21310 -1.22772
H -3.44300 2.76580 -0.76584

```
H   2.67180 -3.42692  0.32369
H   2.35447 -3.37524 -1.42797
H   4.01397 -3.18467 -0.81881
H   2.04031 -1.95761 -3.24946
H   2.06171 -0.25735 -3.77420
H   3.59275 -1.13705 -3.52995
H   2.23610  1.84453 -3.09065
H   1.61364  2.66108 -1.61709
H   3.37267  2.64432 -1.96853
H   3.65640  2.54866  0.93624
H   1.85995  2.62549  0.88486
H   2.69046  1.70809  2.18347
H   4.19877 -0.89761  2.30895
H   2.50257 -0.57764  2.77766
H   2.97027 -2.17509  2.15190
H  -0.32053 -1.39469 -1.04220
H  -0.76057 -2.88523 -0.20589
H  -0.11301  4.97402 -0.06634
H  -1.42272  5.19859 -1.25852
H  -1.80700  5.12207  0.47508
H   0.37239 -2.07386  1.67374
C  -0.78450  0.31987  4.44174
H  -0.06022  0.27824  5.26770
H  -0.95136  1.36295  4.12608
H  -1.75100 -0.10943  4.75288
```

TS(E-G)$_{cis}$

SCF Energy = -1302.68538870
Enthalpy 0K = -1302.223941
Energy 298K = -1302.192892
Free Energy 298K = -1302.284649
Lowest Frequency = -581.8323 cm^{-1}
SCF (DCE) Energy = -1302.69666416
SCF (BP86-D3) Energy = -1302.77449169
SCF (BS2) Energy = -1302.65504304

```
Rh  0.89426 -0.36215 -0.15495
C   2.89575 -0.36126  0.88727
C   2.87831 -1.38637 -0.14344
C   2.73213 -0.72127 -1.43484
C   2.59414  0.68728 -1.19534
C   2.68477  0.91491  0.25372
C  -2.66748 -0.39138 -0.78099
C  -3.97760 -0.41087 -1.32599
C  -2.14017 -1.62772 -0.30266
C  -4.74434 -1.57721 -1.38755
H  -4.39054  0.51994 -1.72489
C  -2.91465 -2.80333 -0.40165
C  -4.21441 -2.79092 -0.91953
H  -5.75169 -1.54151 -1.81588
H  -2.48300 -3.74680 -0.04549
H  -4.79765 -3.71573 -0.96926
C  -2.47728  2.18129 -0.94474
C  -1.38976  3.05468 -0.78541
H  -3.51462  2.44791 -1.14281
N  -0.60521  1.03818 -0.47779
N  -0.26048  2.34519 -0.50813
C  -1.94888  0.88475 -0.74239
C   3.14999 -2.85306  0.05758
H   2.79589 -3.20369  1.04062
H   2.65620 -3.46506 -0.71486
H   4.23403 -3.06743  0.00417
C   2.79612 -1.39538 -2.77865
H   2.37836 -2.41422 -2.74551
H   2.23975 -0.82873 -3.54107
H   3.84505 -1.47857 -3.12041
C   2.45133  1.78225 -2.20974
H   2.24201  1.38428 -3.21496
H   1.62269  2.44954 -1.91224
H   3.37943  2.38134 -2.26582
C   2.65707  2.26076  0.91426
H   3.56270  2.84267  0.65861
H   1.76766  2.81716  0.56643
H   2.60596  2.16693  2.00998
C   3.11013 -0.57220  2.36221
H   4.08589 -0.15365  2.66937
H   2.31965 -0.08511  2.95879
H   3.11582 -1.64296  2.62125
C  -0.75947 -1.80333  0.26522
H  -0.06821 -1.34788 -1.00976
H  -0.43280 -2.84997  0.17051
C  -0.21126 -1.17937  1.47556
C  -1.33942  4.55404 -0.88205
H  -0.32637  4.91197 -0.63759
H  -1.59084  4.91125 -1.89723
H  -2.04819  5.03512 -0.18452
C  -0.80410 -0.19284  2.41515
H   0.51917 -1.79316  2.01680
O  -0.17785  0.25110  3.38164
O  -2.08926  0.14927  2.13935
C  -2.63792  1.17766  2.99495
H  -2.58687  0.87273  4.05232
H  -2.08265  2.11979  2.86344
H  -3.68004  1.29764  2.67043
```

G$_{cis}$

SCF Energy = -1302.69430058
Enthalpy 0K = -1302.231410
Energy 298K = -1302.200038
Free Energy 298K = -1302.292649
Lowest Frequency = 24.0785 cm^{-1}
SCF (DCE) Energy = -1302.70509395
SCF (BP86-D3) Energy = -1302.78485063
SCF (BS2) Energy = -1302.66238508

```
Rh  0.84515 -0.38664 -0.21476
C   2.87369 -0.10218  0.93946
C   2.85527 -1.34080  0.15746
C   2.72496 -0.98305 -1.23559
C   2.56290  0.46436 -1.30964
C   2.69912  0.99759  0.04496
C  -2.64209 -0.54071 -0.67341
C  -3.93234 -0.68512 -1.24136
C  -2.04038 -1.69351 -0.07988
C  -4.61936 -1.90263 -1.23450
H  -4.38981  0.18498 -1.72175
C  -2.73725 -2.92317 -0.11761
C  -4.01716 -3.03915 -0.67127
H  -5.61475 -1.96791 -1.68660
H  -2.25778 -3.80644  0.32176
H  -4.53200 -4.00523 -0.67085
C  -2.56484  2.02805 -1.05360
C  -1.49182  2.93746 -1.03293
H  -3.61693  2.24900 -1.22978
N  -0.65021  0.98373 -0.56377
N  -0.33173  2.28356 -0.74589
C  -1.99358  0.76990 -0.75547
C   3.11828 -2.72062  0.69728
H   2.62853 -2.88103  1.67246
H   2.75334 -3.49816  0.00764
H   4.20143 -2.88949  0.84610
C   2.85558 -1.92037 -2.40654
H   2.45837 -2.92135 -2.17661
H   2.31934 -1.54096 -3.28962
H   3.92046 -2.03636 -2.68254
C   2.45005  1.31412 -2.54343
H   2.16248  0.71739 -3.42314
H   1.68440  2.09274 -2.38809
H   3.41259  1.81072 -2.76914
C   2.65757  2.45257  0.40045
H   3.47450  3.00161 -0.10275
H   1.69069  2.87631  0.06452
H   2.75383  2.60344  1.48644
C   3.08567  0.00514  2.42578
```

H   4.01478 0.56400 2.63935
H   2.24629 0.52861 2.91664
H   3.18600 -0.98840 2.89100
C -0.69974 -1.74054 0.57205
H   0.16220 -1.04247 -1.45635
C -0.17100 -0.93100 1.63078
C -1.48669 4.42157 -1.27275
H -0.45987 4.81165 -1.18659
H -1.86566 4.67683 -2.27870
H -2.11707 4.96042 -0.54250
H -0.27831 -2.75474 0.55056
C -0.76419 0.20545 2.39326
H   0.62831 -1.38778 2.22591
O -2.08882 0.38811 2.17043
O -0.11168 0.87970 3.19382
C -2.66647 1.52892 2.84770
H -2.22823 2.46144 2.45888
H -3.73998 1.48553 2.62147
H -2.48839 1.46744 3.93290

TS(E1′-E_{trans})
SCF (BP86) Energy = -1302.68579385
Enthalpy 0K = -1302.220986
Energy 298K = -1302.190166
Free Energy 298K = -1302.281086
Lowest Frequency = -34.1046 cm^{-1}
SCF (DCE) Energy = -1302.69801107
SCF (BP86-D3) Energy = -1302.77250955
SCF (BS2) Energy = -1302.65508807

Rh 0.57078 0.20722 0.31867
C   2.76281 0.18595 0.50142
C   2.22420 1.55121 0.31212
C   1.46833 1.92318 1.50428
C   1.38769 0.75285 2.31984
C   2.20625 -0.32155 1.71437
C -2.93457 -0.52793 -0.09201
C -4.29474 -0.36936 0.29912
C -2.30617 -1.77856 0.17525
C -5.02340 -1.37990 0.92700
H -4.76592 0.60174 0.11996
C -3.06167 -2.77980 0.83208
C -4.39561 -2.60439 1.20777
H -6.06695 -1.20718 1.21070
H -2.57254 -3.74175 1.03220
H -4.93788 -3.41241 1.71001
C -2.98082 1.66170 -1.46698
C -2.02926 2.69437 -1.63915
H -4.00479 1.63181 -1.83810
N -1.04978 1.13243 -0.46667
N -0.86753 2.36875 -1.02493
C -2.32424 0.66672 -0.72082
C -0.89577 -2.23121 -0.20379
C   0.04195 -1.33080 -1.03148
H -0.46499 -0.87326 -1.89240
H -1.02292 -3.17777 -0.77299
C   2.46081 -1.67946 2.30475
H   3.41405 -1.69410 2.86679
H   1.66152 -1.96881 3.00581
H   2.51189 -2.44398 1.51180
C   0.62837 0.61029 3.60586
H   0.12001 -0.36622 3.66536
H   1.31736 0.67506 4.46926
H -0.13351 1.39642 3.71710
C   3.79664 -0.47579 -0.35712
H   4.80864 -0.23017 0.01711
H   3.68053 -1.57088 -0.34858
H   3.73237 -0.13067 -1.40089
C   2.49884 2.47444 -0.83328
H   3.18721 3.28295 -0.52097
H   2.95172 1.94348 -1.68422
H   1.54723 2.92791 -1.16611

C   0.80555 3.25326 1.70924
H   1.55876 4.05339 1.82884
H   0.17572 3.49330 0.83247
H   0.16693 3.25541 2.60622
H -0.37225 -2.55787 0.71581
C   1.23362 -2.09130 -1.48883
C -2.16300 3.99552 -2.37829
H -2.39176 3.83587 -3.44725
H -2.97384 4.62105 -1.96330
H -1.22189 4.56422 -2.31131
O   1.65168 -1.67690 -2.73673
O   1.81116 -3.00173 -0.88294
C   2.74183 -2.43933 -3.29481
H   2.86402 -2.06688 -4.32173
H   3.67347 -2.28986 -2.72411
H   2.50503 -3.51571 -3.29842

E_{trans}′
SCF (BP86) Energy = -1302.70139684
Enthalpy 0K = -1302.236848
Energy 298K = -1302.205810
Free Energy 298K = -1302.297080
Lowest Frequency = 31.9559 cm^{-1}
SCF (DCE) Energy = -1302.71162559
SCF (BP86-D3) Energy = -1302.78847096
SCF (BS2) Energy = -1302.67091710

Rh -0.62183 0.19108 -0.33477
C -2.81729 0.10094 0.01176
C -2.30246 1.43458 0.27550
C -1.81230 1.98460 -0.99919
C -1.94959 0.97371 -1.99830
C -2.54547 -0.21358 -1.36984
C   3.03104 -0.29312 -0.20716
C   4.44104 -0.27753 -0.37894
C   2.33631 -1.45276 -0.66163
C   5.13497 -1.34617 -0.95250
H   4.99615 0.61198 -0.06876
C   3.04462 -2.51292 -1.26157
C   4.43717 -2.48016 -1.39996
H   6.22308 -1.28739 -1.06232
H   2.48237 -3.38755 -1.61009
H   4.96715 -3.32230 -1.85598
C   3.05048 1.91057 1.13189
C   2.03893 2.81130 1.49801
H   4.11047 1.97630 1.37301
N   1.05136 1.20069 0.39234
N   0.83537 2.36737 1.04323
C   2.39730 0.87552 0.42278
C   0.83959 -1.59357 -0.55240
C   0.18725 -1.41270 0.79084
H   0.82069 -1.04404 1.60378
H   0.46165 -2.50485 -1.04454
C -2.93042 -1.48836 -2.06949
H -4.00153 -1.47811 -2.34647
H -2.35263 -1.62671 -2.99763
H -2.74615 -2.36198 -1.42322
C -1.60698 1.10570 -3.45666
H -1.25345 0.15253 -3.88131
H -2.49667 1.41417 -4.03753
H -0.82143 1.85940 -3.62069
C -3.57037 -0.76912 0.97491
H -4.65573 -0.57270 0.88941
H -3.40155 -1.83678 0.76249
H -3.27358 -0.57114 2.01669
C -2.38089 2.20863 1.55672
H -3.12478 3.02407 1.47947
H -2.66813 1.56290 2.40130
H -1.38986 2.64928 1.77045
C -1.27762 3.37478 -1.16196
H -2.08910 4.11621 -1.04023
H -0.51279 3.56370 -0.38642

H -0.82374 3.52610 -2.15369
H   0.47533 -0.75105 -1.36027
C -0.76995 -2.45985 1.20166
C   2.13280 4.09854 2.26842
H   2.62127 3.95347 3.24827
H   2.71568 4.86424 1.72487
H   1.12394 4.50398 2.44725
O -0.92150 -2.47741 2.56673
O -1.37821 -3.24163 0.46076
C -1.80140 -3.50775 3.06419
H -1.74407 -3.43742 4.15925
H -2.83768 -3.34447 2.72514
H -1.47244 -4.50210 2.72121

TS(E-G)$_{trans}$′
SCF (BP86) Energy = -1302.69754296
Enthalpy 0K = -1302.235673
Energy 298K = -1302.204820
Free Energy 298K = -1302.295643
Lowest Frequency = -576.7566 cm^{-1}
SCF (DCE) Energy = -1302.70721657
SCF (BP86-D3) Energy = -1302.78488837
SCF (BS2) Energy = -1302.66655289

Rh -0.54284 0.18942 -0.37974
C -2.80387 0.24119 -0.04651
C -2.25152 1.55574 0.15636
C -1.65156 1.98462 -1.11228
C -1.83019 0.92755 -2.07213
C -2.50480 -0.17915 -1.40410
C   2.99882 -0.41022 -0.15753
C   4.40073 -0.46510 -0.36826
C   2.22347 -1.52126 -0.60727
C   5.01636 -1.55522 -0.98992
H   5.00973 0.38753 -0.05412
C   2.85475 -2.59968 -1.26252
C   4.24156 -2.63441 -1.44774
H   6.10213 -1.55465 -1.13310
H   2.23638 -3.43523 -1.61168
H   4.70985 -3.48851 -1.94698
C   3.11135 1.77906 1.21990
C   2.12749 2.71836 1.56822
H   4.16744 1.79564 1.48628
N   1.10552 1.16065 0.42733
N   0.91698 2.32925 1.07740
C   2.43145 0.78016 0.48495
C   0.74277 -1.62032 -0.40927
C   0.12512 -1.41463 0.89820
H   0.73470 -1.01096 1.71155
H   0.27837 -2.43420 -0.98400
C -2.96330 -1.46354 -2.03928
H -4.03617 -1.41317 -2.30496
H -2.40419 -1.67351 -2.96528
H -2.81487 -2.31421 -1.35437
C -1.46199 0.98254 -3.53027
H -1.20990 -0.01378 -3.92598
H -2.30950 1.37167 -4.12526
H -0.59884 1.64310 -3.70514
C -3.63155 -0.53923 0.93245
H -4.70442 -0.32226 0.77170
H -3.48577 -1.62337 0.80251
H -3.38774 -0.27499 1.97319
C -2.33179 2.40866 1.38771
H -2.99261 3.28011 1.22184
H -2.72591 1.84232 2.24620
H -1.31964 2.77430 1.64141
C -1.03742 3.33433 -1.33865
H -1.81891 4.11701 -1.35065
H -0.33010 3.55906 -0.52086
H -0.49521 3.38099 -2.29588
H   0.49328 -0.45043 -1.42234
C -0.92904 -2.36161 1.32041
C   2.25349 3.99453 2.35244
H   2.68968 3.81924 3.35191
H   2.89837 4.73202 1.84101
H   1.25972 4.45017 2.48972
O -1.08974 -2.32525 2.68249
O -1.58574 -3.11705 0.59616
C -2.03934 -3.27916 3.20548
H -1.98022 -3.18061 4.29814
H -3.06052 -3.05478 2.85638
H -1.77712 -4.30271 2.89244

G$_{trans}$′
SCF (BP86) Energy = -1302.70422939
Enthalpy 0K = -1302.240791
Energy 298K = -1302.209679
Free Energy 298K = -1302.300871
Lowest Frequency = 32.6685 cm^{-1}
SCF (DCE) Energy = -1302.71384480
SCF (BP86-D3) Energy = -1302.79200553
SCF (BS2) Energy = -1302.67195265

Rh -0.44610 0.18440 -0.45869
C -2.78067 0.32293 -0.06310
C -2.21395 1.63464 -0.01772
C -1.55889 1.88262 -1.30082
C -1.79203 0.72137 -2.15229
C -2.49025 -0.26828 -1.36914
C   2.96467 -0.48817 -0.09534
C   4.35727 -0.56929 -0.34234
C   2.14258 -1.57448 -0.53346
C   4.93198 -1.66043 -1.00239
H   4.99126 0.26654 -0.03102
C   2.73819 -2.65086 -1.22910
C   4.11720 -2.70869 -1.46136
H   6.01294 -1.68188 -1.17671
H   2.09410 -3.46996 -1.57061
H   4.55009 -3.56116 -1.99459
C   3.12276 1.70449 1.29792
C   2.15650 2.68106 1.59826
H   4.16963 1.69101 1.59908
N   1.13213 1.14283 0.44805
N   0.95180 2.32695 1.06622
C   2.43849 0.71933 0.55010
C   0.68454 -1.67694 -0.26588
C   0.08319 -1.36531 0.99826
H   0.67853 -0.89486 1.78547
H   0.17915 -2.47003 -0.83299
C -3.00475 -1.59594 -1.85245
H -4.08937 -1.54256 -2.06497
H -2.49995 -1.90542 -2.78127
H -2.83869 -2.37985 -1.09580
C -1.47096 0.62259 -3.62006
H -1.23380 -0.41034 -3.91797
H -2.33979 0.95415 -4.21898
H -0.61393 1.25740 -3.89205
C -3.64652 -0.31582 0.98321
H -4.70999 -0.08987 0.77677
H -3.53461 -1.41161 0.97709
H -3.41456 0.05747 1.99263
C -2.26886 2.62185 1.10957
H -2.80811 3.53686 0.80281
H -2.78118 2.20368 1.99006
H -1.23648 2.90144 1.39641
C -0.92310 3.18850 -1.68614
H -1.69350 3.96297 -1.86105
H -0.25953 3.53433 -0.87577
H -0.32457 3.09316 -2.60552
H   0.58698 0.14600 -1.62007
C -1.03161 -2.20848 1.49076
C   2.29479 3.95914 2.37730
H   2.58810 3.77177 3.42596
H   3.05889 4.62651 1.94010

H   1.33449 4.49917 2.38528
O -1.18838 -2.03972 2.84294
O -1.72021 -2.99783 0.83832
C -2.16607 -2.91019 3.45356
H -2.09276 -2.71916 4.53292
H -3.18193 -2.68001 3.09309
H -1.94261 -3.96543 3.22835

TS(E2″-E_{cis})$_{2,1}$
SCF (BP86) Energy = -1302.67885510
Enthalpy 0K = -1302.214809
Energy 298K = -1302.183762
Free Energy 298K = -1302.275943
Lowest Frequency = -99.3124 cm^{-1}
SCF (DCE) Energy = -1302.69337364
SCF (BP86-D3) Energy = -1302.76503020
SCF (BS2) Energy = -1302.64822694

C   1.96181 0.98931 -0.59104
C   2.39147 2.32358 -0.72176
C   1.22053 3.10751 -0.71468
N   0.12967 2.31472 -0.60377
N   0.57556 1.02294 -0.53042
C   1.07480 4.60044 -0.79707
Rh -0.87679 -0.36084 -0.23111
C   0.29692 -1.15241 1.31016
H -0.40858 -1.81456 1.84126
C   2.83956 -0.19909 -0.64175
C   4.09089 0.00560 -1.28804
C   5.08022 -0.97591 -1.36268
C   4.84343 -2.23335 -0.78914
C   3.60987 -2.46713 -0.17458
C   2.59172 -1.48996 -0.08563
C   1.28886 -2.01096 0.50953
C -2.83381 -0.76130 0.63639
C -2.71022 0.62812 0.15610
C -2.67237 0.61013 -1.31404
C -2.58291 -0.74993 -1.70741
C -2.68902 -1.60913 -0.50679
C -3.10960 -1.16721 2.05570
C -2.79564 1.87307 0.98178
C -2.63018 1.83623 -2.17330
C -2.43822 -1.27656 -3.10622
C -2.76103 -3.11040 -0.53599
H   4.27195 0.97412 -1.76153
H   6.02570 -0.75975 -1.87133
H   3.41622 -3.45273 0.26705
H   5.60163 -3.02256 -0.82374
H   3.41976 2.67795 -0.76707
C   0.86398 -0.19747 2.30404
H   1.55147 -2.89104 1.13271
H -3.78155 -3.45142 -0.79521
H -2.07453 -3.53436 -1.28745
H -2.50288 -3.54893 0.44084
H -1.70358 -2.09773 -3.15387
H -3.40160 -1.67857 -3.47294
H -2.11045 -0.49057 -3.80309
H -4.16768 -0.97391 2.31256
H -2.92262 -2.24164 2.21285
H -2.47351 -0.60657 2.76008
H -3.78722 2.35076 0.86216
H -2.63648 1.65854 2.04898
H -2.01864 2.58090 0.64192
H -3.55383 2.43131 -2.05213
H -1.76722 2.45926 -1.86874
H -2.52717 1.58381 -3.24023
H   0.71295 -2.46462 -0.33465
H   1.45793 4.99748 -1.75458
H   0.01311 4.88216 -0.71039
H   1.63226 5.10868 0.00985
O   2.02670 0.17461 2.40940
O -0.12395 0.24257 3.17547
C   0.34741 1.18994 4.15722
H -0.52188 1.41718 4.79103
H   1.16272 0.75884 4.76099
H   0.71988 2.10590 3.67013

E_{cis}′
SCF (BP86) Energy = -1302.68778666
Enthalpy 0K = -1302.224080
Energy 298K = -1302.192776
Free Energy 298K = -1302.285085
Lowest Frequency = 28.3512 cm^{-1}
SCF (DCE) Energy = -1302.70160065
SCF (BP86-D3) Energy = -1302.77443536
SCF (BS2) Energy = -1302.65782696

C   1.96752 1.07973 -0.59658
C   2.41912 2.41936 -0.63561
C   1.28320 3.20658 -0.39938
N   0.19214 2.40948 -0.23589
N   0.60705 1.12640 -0.35584
C   1.15281 4.70130 -0.30807
Rh -0.85766 -0.34125 -0.24123
C   0.29716 -1.33563 1.21151
H -0.37243 -2.07433 1.67303
C   2.78501 -0.12768 -0.76519
C   4.11643 0.02910 -1.23199
C   4.98748 -1.05129 -1.39155
C   4.55048 -2.35459 -1.10519
C   3.23292 -2.54035 -0.67215
C   2.35246 -1.45645 -0.48020
C   0.94291 -1.80572 -0.06598
C -2.86117 -0.62165 0.64410
C -2.66920 0.76189 0.24267
C -2.56560 0.79434 -1.22655
C -2.60978 -0.54916 -1.70035
C -2.76621 -1.43949 -0.54207
C -3.14296 -1.09227 2.04405
C -2.72559 1.97809 1.11668
C -2.44021 2.05288 -2.02887
C -2.56857 -0.99690 -3.13552
C -2.95630 -2.93031 -0.61798
H   4.46772 1.03325 -1.48341
H   6.00710 -0.87542 -1.75072
H   2.87066 -3.55403 -0.46024
H   5.21846 -3.21285 -1.22862
H   3.44308 2.76601 -0.76494
C   1.00664 -0.54925 2.24832
H   0.76038 -2.88505 -0.20700
H -4.01445 -3.18445 -0.81749
H -2.35563 -3.37498 -1.42856
H -2.67098 -3.42682 0.32346
H -2.04023 -1.95735 -3.24943
H -3.59282 -1.13707 -3.52990
H -2.06195 -0.25710 -3.77417
H -4.19885 -0.89790 2.30883
H -2.96980 -2.17488 2.15196
H -2.50283 -0.57723 2.77773
H -3.65653 2.54858 0.93676
H -2.68985 1.70817 2.18353
H -1.86007 2.62581 0.88469
H -3.37284 2.64419 -1.96918
H -1.61397 2.66150 -1.61680
H -2.23538 1.84462 -3.09055
H   0.32066 -1.39397 -1.04274
H   1.80495 5.12167 0.47829
H   1.42542 5.19914 -1.25632
H   0.11243 4.97415 -0.06781
O   2.12292 -0.04767 2.20153
O   0.19903 -0.45366 3.37270
C   0.78466 0.31835 4.44185
H   0.06075 0.27585 5.26809
H   1.75151 -0.11066 4.75232

H   0.95086 1.36173 4.12680

TS(E-G)$_{cis}$′

SCF (BP86) Energy = -1302.68538863
Enthalpy 0K = -1302.223940
Energy 298K = -1302.192891
Free Energy 298K = -1302.284643
Lowest Frequency = -581.8398 cm^{-1}
SCF (DCE) Energy = -1302.69666403
SCF (BP86-D3) Energy = -1302.77449298
SCF (BS2) Energy = -1302.65504299

```
C  -2.87829 -1.38637 -0.14354
C  -2.89576 -0.36132  0.88721
C  -2.68477  0.91489  0.25373
C  -2.59412  0.68732 -1.19535
C  -2.73210 -0.72121 -1.43491
Rh -0.89426 -0.36216 -0.15494
C   0.75947 -1.80336  0.26524
C   2.14018 -1.62775 -0.30260
C   2.66750 -0.39143 -0.78097
C   3.97762 -0.41093 -1.32596
C   4.74438 -1.57726 -1.38744
C   4.21446 -2.79095 -0.91936
C   2.91469 -2.80336 -0.40151
C   1.94890  0.88470 -0.74241
C   2.47735  2.18125 -0.94458
C   1.38985  3.05465 -0.78519
N   0.26054  2.34517 -0.50799
N   0.60523  1.03815 -0.47784
C   1.33947  4.55398 -0.88225
C  -3.11022 -0.57233  2.36214
C  -2.65708  2.26071  0.91432
C  -2.45126  1.78234 -2.20968
C  -2.79605 -1.39527 -2.77875
C  -3.14998 -2.85307  0.05743
C   0.21122 -1.17938  1.47556
C   0.80402 -0.19280  2.41513
O   0.17776  0.25116  3.38160
O   2.08915  0.14936  2.13930
H  -0.51922 -1.79316  2.01680
H   4.39055  0.51987 -1.72490
H   5.75174 -1.54157 -1.81575
H   2.48305 -3.74682 -0.04531
H   4.79772 -3.71576 -0.96903
H   3.51471  2.44786 -1.14256
H   0.43282 -2.85001  0.17057
H  -4.23405 -3.06738  0.00441
H  -2.65652 -3.46501 -0.71525
H  -2.79553 -3.20382  1.04031
H  -2.37813 -2.41404 -2.74568
H  -3.84500 -1.47862 -3.12045
H  -2.23982 -0.82848 -3.54116
H  -4.08594 -0.15368  2.66928
H  -3.11605 -1.64310  2.62111
H  -2.31972 -0.08535  2.95879
H  -3.56257  2.84274  0.65847
H  -2.60622  2.16683  2.01005
H  -1.76753  2.81702  0.56670
H  -3.37936  2.38142 -2.26579
H  -1.62265  2.44963 -1.91209
H  -2.24186  1.38442 -3.21491
H   0.06823 -1.34796 -1.00968
H   2.05374  5.03495 -0.19033
H   1.58340  4.91065 -1.89949
H   0.32844  4.91256 -0.63047
C   2.63769  1.17787  2.99483
H   3.67948  1.29872  2.66956
H   2.08162  2.11964  2.86408
H   2.58768  0.87255  4.05214
```

G$_{cis}$′

SCF (BP86) Energy = -1302.69430062
Enthalpy 0K = -1302.231408
Energy 298K = -1302.200037
Free Energy 298K = -1302.292641
Lowest Frequency = 24.1043 cm^{-1}
SCF (DCE) Energy = -1302.70509481
SCF (BP86-D3) Energy = -1302.78485119
SCF (BS2) Energy = -1302.66238459

```
C  -2.85519 -1.34076  0.15757
C  -2.87368 -0.10200  0.93936
C  -2.69917  0.99762  0.04468
C  -2.56287  0.46417 -1.30981
C  -2.72489 -0.98326 -1.23553
Rh -0.84507 -0.38663 -0.21488
C   0.69975 -1.74059  0.57183
C   2.04045 -1.69342 -0.07997
C   2.64216 -0.54056 -0.67336
C   3.93247 -0.68487 -1.24121
C   4.61953 -1.90236 -1.23442
C   4.01733 -3.03894 -0.67131
C   2.73738 -2.92306 -0.11775
C   1.99361  0.77005 -0.75535
C   2.56478  2.02819 -1.05368
C   1.49172  2.93755 -1.03301
N   0.33167  2.28361 -0.74586
N   0.65025  0.98383 -0.56357
C   1.48664  4.42177 -1.27204
C  -3.08574  0.00551  2.42566
C  -2.65769  2.45265  0.39995
C  -2.45006  1.31368 -2.54378
C  -2.85549 -1.92075 -2.40635
C  -3.11809 -2.72045  0.69778
C   0.17092 -0.93127  1.63069
C   0.76401  0.20506  2.39339
O   0.11151  0.87896  3.19426
O   2.08856  0.38809  2.17036
H  -0.62844 -1.38816  2.22567
H   4.38995  0.18528 -1.72148
H   5.61496 -1.96756 -1.68644
H   2.25791 -3.80638  0.32151
H   4.53221 -4.00500 -0.67094
H   3.61684  2.24917 -1.23002
H   0.27837 -2.75482  0.55015
H  -4.20109 -2.88871  0.84832
H  -2.75467 -3.49817  0.00755
H  -2.62689 -2.88116  1.67220
H  -2.45826 -2.92169 -2.17631
H  -3.92038 -2.03677 -2.68234
H  -2.31926 -1.54143 -3.28947
H  -4.01422  0.56544  2.63912
H  -3.18727 -0.98794  2.89081
H  -2.24586  0.52804  2.91668
H  -3.47441  3.00165 -0.10363
H  -2.75432  2.60370  1.48588
H  -1.69068  2.87630  0.06426
H  -3.41278  1.80975 -2.76992
H  -1.68483  2.09269 -2.38843
H  -2.16193  0.71687 -3.42325
H  -0.16230 -1.04204 -1.45679
H   2.10786  4.96152 -0.53457
H   1.87634  4.67807 -2.27357
H   0.45826  4.81003 -1.19694
C   2.66608  1.52885  2.84780
H   3.73942  1.48629  2.62059
H   2.22688  2.46134  2.45996
H   2.48901  1.46660  3.93312
```

1,2-insertion

D1$_{1,2}$
SCF Energy = -1302.69882710
Enthalpy 0K = -1302.234207
Energy 298K = -1302.202636
Free Energy 298K = -1302.295065
Lowest Frequency = 22.8035 cm^{-1}
SCF (DCE) Energy = -1302.70867408
SCF (BP86-D3) Energy = -1302.79341789
SCF (BS2) Energy = -1302.66750737

```
C   2.18638  1.50625 -0.30154
C   3.52802  1.65589 -0.69717
C   3.88163  0.39734 -1.23864
N   2.82609 -0.47009 -1.18213
N   1.81860  0.22486 -0.61994
C   5.18703 -0.04546 -1.83952
Rh -0.03205 -0.44687 -0.05716
C  -0.29203 -0.77811 -2.17552
C  -1.02563  0.39213 -1.84857
C  -2.50866  0.35522 -1.76366
O  -3.22441 -0.65019 -1.71635
C   1.12763  2.27738  0.32915
C   1.21843  3.62589  0.73671
C   0.10959  4.26783  1.30785
C  -1.10100  3.57431  1.46482
C  -1.20068  2.22601  1.06016
C  -0.09332  1.56330  0.51540
C  -1.51364 -1.57135  1.30025
C  -0.92755 -2.52853  0.37229
C   0.49879 -2.55929  0.58014
C   0.80161 -1.61863  1.65272
C  -0.44357 -1.04470  2.11773
C  -2.98680 -1.34645  1.48999
C  -1.72780 -3.39865 -0.55352
C   1.51879 -3.41615 -0.11626
C   2.17046 -1.38830  2.22549
C  -0.59883 -0.15692  3.31752
O  -3.02147  1.62150 -1.77595
H   2.16045  4.16850  0.59780
H   0.18744  5.31479  1.62008
H  -2.15910  1.70474  1.16593
H  -1.97454  4.07819  1.89388
H   4.15355  2.54371 -0.61003
H   0.69388 -0.69812 -2.64196
H  -0.59429  1.37856 -2.03877
H  -0.84307 -1.70867 -2.34473
H  -0.79111 -0.77406  4.21535
H   0.30883  0.43731  3.50194
H  -1.43645  0.54747  3.20335
H   2.23676 -0.41678  2.73871
H   2.42042 -2.17849  2.95810
H   2.93079 -1.40497  1.42833
H  -3.44753 -2.20030  2.02248
H  -3.18074 -0.44263  2.08960
H  -3.49874 -1.23881  0.51892
H  -2.26901 -4.16691  0.02976
H  -2.47504 -2.80253 -1.10414
H  -1.08660 -3.92375 -1.27864
H   1.06743 -3.98563 -0.94382
H   2.32326 -2.78466 -0.53609
H   1.96834 -4.14168  0.58631
H   5.13648 -1.11566 -2.09586
H   5.42577  0.51358 -2.76259
H   6.03141  0.10298 -1.14288
C  -4.46396  1.69153 -1.73247
H  -4.70606  2.76089 -1.79655
H  -4.90644  1.13980 -2.57742
H  -4.84909  1.26502 -0.79193
```

TS(D1-E1)$_{1,2}$
SCF Energy = -1302.66458245
Enthalpy 0K = -1302.201137
Energy 298K = -1302.170017
Free Energy 298K = -1302.261874
Lowest Frequency = -257.0523 cm^{-1}
SCF (DCE) Energy = -1302.67378827
SCF (BP86-D3) Energy = -1302.75621691
SCF (BS2) Energy = -1302.63349908

```
C   1.07910  2.27966 -0.00925
C   2.09919  3.21328 -0.28820
C   3.09082  2.46218 -0.95300
N   2.70761  1.15012 -1.07298
N   1.49516  1.06405 -0.50410
C   4.40788  2.91357 -1.52032
Rh  0.32238 -0.59053 -0.16671
C  -0.40120 -0.19060 -2.06501
C  -1.31992  0.70523 -1.31778
C  -2.76448  0.26017 -1.37791
O  -3.17675 -0.88770 -1.23447
C  -0.17903  2.23929  0.70938
C  -0.57121  3.17937  1.68283
C  -1.71692  2.96761  2.46401
C  -2.48278  1.79929  2.29136
C  -2.13470  0.87165  1.29794
C  -0.99902  1.07249  0.47390
C  -0.38201 -2.46383  0.88369
C   0.36323 -2.81495 -0.30389
C   1.69988 -2.27173 -0.17041
C   1.78524 -1.64321  1.16270
C   0.51937 -1.77749  1.80986
C  -1.79432 -2.88650  1.18319
C  -0.16198 -3.64373 -1.44269
C   2.85659 -2.41497 -1.11905
C   3.03286 -1.00190  1.69711
C   0.14812 -1.31410  3.18985
O  -3.57383  1.31771 -1.65660
H   0.05969  4.05805  1.85684
H  -2.00247  3.70440  3.22223
H  -2.76712 -0.00689  1.13255
H  -3.36509  1.62087  2.91556
H   2.11206  4.27720 -0.05310
H   0.33745  0.30403 -2.70627
H  -1.21300  1.75940 -1.60230
H  -0.85225 -1.09311 -2.49609
H  -0.00523 -2.17359  3.86861
H   0.93564 -0.68009  3.62575
H  -0.78475 -0.72382  3.17997
H   2.83099 -0.40347  2.59892
H   3.78258 -1.77183  1.95821
H   3.47852 -0.33715  0.93655
H  -1.83814 -3.96576  1.42387
H  -2.19704 -2.33851  2.04964
H  -2.45850 -2.69668  0.32452
H  -0.06645 -4.72166 -1.21321
H  -1.22807 -3.43632 -1.63031
H   0.39347 -3.45285 -2.37466
H   2.51978 -2.75290 -2.11201
H   3.36642 -1.44465 -1.24950
H   3.59227 -3.15163 -0.74333
H   4.99352  2.03839 -1.84379
H   4.27588  3.57419 -2.39680
H   5.00339  3.47362 -0.77786
C  -4.98125  0.99254 -1.75727
H  -5.48032  1.93557 -2.01695
H  -5.14907  0.23305 -2.53729
H  -5.35686  0.60654 -0.79609
```

E1$_{1,2}$
SCF Energy = -1302.68381851
Enthalpy 0K = -1302.219520
Energy 298K = -1302.187584
Free Energy 298K = -1302.284049
Lowest Frequency = 9.6352 cm^{-1}

SCF (DCE) Energy = -1302.69681441
SCF (BP86-D3) Energy = -1302.76595434
SCF (BS2) Energy = -1302.65527226

```
Rh  0.99449 -0.38895  0.09987
C   2.27502 -1.92401  1.01575
C   2.29118 -2.04964 -0.40927
C   2.81688 -0.77431 -0.95017
C   3.26634  0.05494  0.15906
C   2.84675 -0.60236  1.35450
C  -1.85709  1.53127  0.93403
C  -2.22225  2.29192  2.06848
C  -2.45193  0.25431  0.73695
C  -3.13643  1.79963  3.00884
H  -1.76008  3.27522  2.20728
C  -3.34942 -0.23917  1.70371
C  -3.69191  0.52285  2.83163
H  -3.40414  2.40574  3.88110
H  -3.79398 -1.22784  1.55432
H  -4.39873  0.12014  3.56518
C  -0.80799  3.39565 -0.53780
C   0.37456  3.41580 -1.31988
H  -1.53179  4.19687 -0.39104
N   0.23730  1.40933 -0.46180
N   1.00371  2.21901 -1.25498
C  -0.87998  2.09806 -0.01640
C  -2.08271 -0.53907 -0.51442
C  -0.80129 -1.37545 -0.30590
H  -0.61308 -1.98622 -1.20969
H  -1.90619  0.18302 -1.33018
H  -0.95527 -2.07225  0.54181
C  -3.21736 -1.45724 -0.97063
C   2.98486 -0.08787  2.75714
H   3.82569 -0.58984  3.27239
H   3.17056  0.99651  2.77590
H   2.07383 -0.28712  3.34566
C   3.91356  1.40041  0.01253
H   4.07567  1.88044  0.99035
H   4.89632  1.31264 -0.48571
H   3.26575  2.05860 -0.59602
C   1.85471 -2.96601  2.01321
H   2.72505 -3.55089  2.36700
H   1.38130 -2.50919  2.89761
H   1.13228 -3.67363  1.57666
C   1.92515 -3.26020 -1.21668
H   2.81231 -3.90090 -1.37718
H   1.15846 -3.86881 -0.71127
H   1.53275 -2.98148 -2.20790
C   2.99175 -0.41081 -2.39267
H   2.43295 -1.08741 -3.05710
H   2.63001  0.62064 -2.55259
H   4.06080 -0.45506 -2.67604
C   0.93809  4.52994 -2.15530
H   0.24580  4.82406 -2.96471
H   1.13244  5.43464 -1.55158
H   1.88671  4.21205 -2.61650
O  -3.59004 -2.48620 -0.41518
O  -3.77489 -1.00114 -2.13369
C  -4.85266 -1.82131 -2.64124
H  -5.18384 -1.33124 -3.56711
H  -4.50040 -2.84531 -2.84648
H  -5.67779 -1.87408 -1.91264
```

TS(E1-F1)$_{1,2}$
SCF Energy = -1302.60204466
Enthalpy 0K = -1302.139780
Energy 298K = -1302.108200
Free Energy 298K = -1302.202011
Lowest Frequency = -536.2052 cm^{-1}
SCF (DCE) Energy = -1302.61085946
SCF (BP86-D3) Energy = -1302.69025228
SCF (BS2) Energy = -1302.56864849

```
Rh  0.46718 -0.29140 -0.18064
C   1.10369 -2.41541 -0.13841
C   0.71414 -2.09686 -1.49652
C   1.59625 -1.03924 -1.92363
C   2.66932 -0.87450 -0.90681
C   2.38196 -1.73260  0.16656
C  -0.17291  0.72679  1.73066
C   0.36074  0.27557  2.98391
C  -1.33454  0.03522  1.14791
C  -0.17963 -0.81916  3.63717
H   1.20694  0.82497  3.41072
C  -1.86649 -1.08047  1.88364
C  -1.29925 -1.50434  3.07486
H   0.23870 -1.14956  4.59366
H  -2.74807 -1.58819  1.48375
H  -1.73317 -2.35839  3.60580
C   0.90484  3.19294  1.39045
C   1.22398  3.74101  0.10772
H   1.12147  3.61965  2.36896
N   0.27046  1.85671 -0.27818
N   0.82855  2.92566 -0.90227
C   0.27279  1.98607  1.09705
C  -2.18472  0.82809  0.14081
C  -1.27812  1.12195 -1.06347
H  -1.44332  2.08782 -1.55914
H  -2.52692  1.77401  0.59755
H  -1.26990  0.31251 -1.80814
C  -3.41731  0.11192 -0.40918
C   3.14403 -1.91726  1.44610
H   3.56490 -2.93791  1.52255
H   3.98023 -1.20426  1.52768
H   2.48945 -1.77072  2.32572
C   3.82288  0.07705 -1.05494
H   4.40511  0.15549 -0.12283
H   4.51411 -0.24821 -1.85577
H   3.47358  1.09080 -1.31762
C   0.50835 -3.49415  0.72092
H   1.12285 -4.41408  0.67737
H   0.44942 -3.18207  1.77762
H  -0.51102 -3.75206  0.39380
C  -0.37678 -2.75178 -2.29889
H  -0.01222 -3.66358 -2.81142
H  -1.23083 -3.03955 -1.66477
H  -0.76564 -2.07653 -3.08000
C   1.61827 -0.37911 -3.27615
H   0.63098 -0.42181 -3.76511
H   1.90980  0.68097 -3.19895
H   2.34107 -0.87749 -3.95101
C   1.88543  5.05368 -0.20319
H   1.31046  5.90549  0.20219
H   2.89967  5.10987  0.23107
H   1.96826  5.18006 -1.29369
O  -3.52030 -1.08112 -0.67264
O  -4.41049  1.01926 -0.64646
C  -5.59437  0.46419 -1.26958
H  -6.28710  1.30869 -1.38388
H  -5.34820  0.02656 -2.25049
H  -6.03560 -0.31917 -0.63313
```

F1$_{1,2}$
SCF Energy = -1302.67315814
Enthalpy 0K = -1302.207800
Energy 298K = -1302.176248
Free Energy 298K = -1302.269738
Lowest Frequency = 16.5313 cm^{-1}
SCF (DCE) Energy = -1302.68214205
SCF (BP86-D3) Energy = -1302.76475351
SCF (BS2) Energy = -1302.63981975

```
C   0.64395 -2.51238 -0.44702
N  -0.66898 -1.88360 -0.15259
```

```
C  -1.00732 -1.51698  1.19991
C  -0.00392 -0.69623  1.84523
C   1.25142 -0.53181  1.06771
C   1.78773 -1.75846  0.26645
C   2.23057  0.36004  1.66695
C   2.02159  0.97827  2.88233
C   0.83256  0.72755  3.64659
C  -0.15467 -0.09399  3.14560
C  -2.31473 -1.97915  1.42108
C  -2.70858 -2.57669  0.19543
N  -1.74262 -2.55986 -0.74612
Rh -0.39609  0.36491 -0.01629
C  -0.42359  2.55297 -0.06404
C   0.29908  2.08821 -1.26005
C  -0.61214  1.31650 -2.04109
C  -1.91616  1.32659 -1.34908
C  -1.81712  2.15590 -0.18286
C   1.72068  2.42918 -1.60983
C  -0.33859  0.65144 -3.36128
C  -3.14991  0.64141 -1.86791
C  -2.92442  2.53278  0.76147
C   0.13230  3.46935  0.98870
C  -4.04334 -3.18560 -0.13262
C   2.84652 -1.33644 -0.75297
O   2.63957 -0.99650 -1.91194
O   4.09746 -1.38802 -0.20474
H  -1.06200 -0.30384  3.72260
H   0.71604  1.18756  4.63335
H   3.18709  0.49951  1.15216
H   2.78925  1.64501  3.28995
H  -2.91695 -1.84328  2.31729
H   0.71822  0.35401 -3.44699
H  -0.95037 -0.25769 -3.48151
H  -0.57838  1.32491 -4.20733
H  -3.53741  1.15040 -2.77081
H  -2.93760 -0.40752 -2.13600
H  -3.95370  0.63804 -1.11462
H  -3.38227  3.50248  0.48491
H  -3.72830  1.77834  0.76528
H  -2.55523  2.62865  1.79634
H  -0.05509  4.52976  0.73130
H  -0.32743  3.27590  1.97203
H   1.21894  3.33585  1.10426
H   1.77567  3.39177 -2.15434
H   2.34141  2.52907 -0.70380
H   2.17385  1.65026 -2.24280
H   2.28787 -2.43536  0.98512
H   0.60177 -3.56921 -0.12740
H   0.76424 -2.47065 -1.53964
H  -4.81499 -2.40896 -0.27986
H  -3.97199 -3.77744 -1.05810
H  -4.38986 -3.84112  0.68406
C   5.16383 -0.98777 -1.09963
H   6.09235 -1.13413 -0.53156
H   5.15877 -1.60946 -2.00879
H   5.05057  0.06857 -1.39216
```

TS(E1-E$_{gem}$)$_{1,2}$
SCF (BP86) Energy = -1302.67475082
Enthalpy 0K = -1302.210813
Energy 298K = -1302.179858
Free Energy 298K = -1302.272334
Lowest Frequency = -34.8573 cm^{-1}
SCF (DCE) Energy = -1302.68659134
SCF (BP86-D3) Energy = -1302.75820667
SCF (BS2) Energy = -1302.64421708

```
Rh -0.95558 -0.16423 -0.20171
C  -2.39070 -1.44815 -1.16127
C  -2.27510 -1.86557  0.20691
C  -2.84854 -0.80317  1.07136
C  -3.25508  0.27079  0.25929
C  -2.85231 -0.05467 -1.12803
C   2.56911  1.46742 -0.22404
C   3.67861  2.29647 -0.54003
C   2.79434  0.05880 -0.21781
C   4.95042  1.77994 -0.80371
H   3.52196  3.37695 -0.59853
C   4.07876 -0.44992 -0.48472
C   5.15999  0.39349 -0.77086
H   5.77289  2.46119 -1.04616
H   4.23407 -1.53392 -0.48190
H   6.14675 -0.03083 -0.98259
C   1.21348  3.48623  0.55556
C  -0.15798  3.74286  0.74617
H   2.04273  4.16354  0.75420
N  -0.00530  1.65605  0.07932
N  -0.87815  2.63753  0.45086
C   1.29910  2.15190  0.11123
C   1.61460 -0.88932 -0.04782
C   0.67128 -0.78815 -1.26537
H   0.52008 -1.76500 -1.74860
H   1.07832 -0.54425  0.88244
H   1.02480 -0.04523 -1.99519
C   1.97791 -2.33851  0.29341
C  -3.15422  0.81281 -2.31427
H  -4.22317  0.72828 -2.59242
H  -2.94666  1.87000 -2.08515
H  -2.55307  0.52366 -3.19005
C  -3.87327  1.56990  0.67217
H  -4.73243  1.82225  0.02592
H  -4.22807  1.53572  1.71436
H  -3.10815  2.36745  0.58929
C  -2.14997 -2.28177 -2.38743
H  -3.09028 -2.76235 -2.71715
H  -1.77403 -1.67284 -3.22530
H  -1.41622 -3.08105 -2.19711
C  -1.83434 -3.21881  0.69169
H  -2.68269 -3.93020  0.70000
H  -1.04230 -3.64099  0.05306
H  -1.44280 -3.16523  1.72076
C  -2.95834 -0.90698  2.56551
H  -2.00344 -1.22490  3.01795
H  -3.23710  0.05640  3.01815
H  -3.71917 -1.65647  2.85392
C  -0.83582  5.00347  1.20338
H  -0.46906  5.32867  2.19335
H  -0.66172  5.83973  0.50241
H  -1.92322  4.84237  1.27776
O   1.72068 -3.33059 -0.37772
O   2.59660 -2.40240  1.50906
C   2.97060 -3.73603  1.92712
H   3.45257 -3.61102  2.90606
H   2.08182 -4.38216  2.00956
H   3.66952 -4.18877  1.20543
```

E$_{gem}$
SCF (BP86) Energy = -1302.68094310
Enthalpy 0K = -1302.217799
Energy 298K = -1302.186442
Free Energy 298K = -1302.279217
Lowest Frequency = 25.7317 cm^{-1}
SCF (DCE) Energy = -1302.69160779
SCF (BP86-D3) Energy = -1302.76911662
SCF (BS2) Energy = -1302.65017990

```
Rh -0.80065 -0.10716 -0.12876
C  -2.63358 -1.00130 -0.96757
C  -2.23674 -1.78508  0.17682
C  -2.35039 -0.94316  1.37764
C  -2.75905  0.35408  0.96998
C  -2.88846  0.35132 -0.50225
C   2.62508  1.25457  0.04063
C   3.88491  1.85874  0.30438
```

C	2.62794	-0.15264	-0.20290
C	5.08300	1.14237	0.28977
H	3.91169	2.92590	0.53789
C	3.84317	-0.86501	-0.21290
C	5.07088	-0.23380	0.01612
H	6.02591	1.65982	0.49570
H	3.83237	-1.93930	-0.42326
H	5.99981	-0.81189	-0.01131
C	1.45625	3.55265	0.04799
C	0.10923	3.94123	0.03159
H	2.32555	4.20811	0.04779
N	0.11476	1.75354	-0.00384
N	-0.69023	2.84045	-0.00135
C	1.44444	2.13681	0.02169
C	-1.93621	-3.25883	0.17632
H	-1.40891	-3.56910	-0.74013
H	-1.30757	-3.53733	1.03764
H	-2.86873	-3.85057	0.24521
C	-2.11198	-1.41566	2.78556
H	-1.24787	-2.09724	2.84601
H	-1.91944	-0.57183	3.46588
H	-2.99236	-1.96439	3.17045
C	-3.01791	1.55662	1.82546
H	-2.73088	1.38305	2.87439
H	-2.43702	2.41199	1.43290
H	-4.09049	1.82538	1.80433
C	-3.37397	1.52007	-1.30631
H	-4.43484	1.73957	-1.08061
H	-2.76625	2.40801	-1.05413
H	-3.28792	1.33091	-2.38791
C	-2.80780	-1.50839	-2.37211
H	-3.83429	-1.89392	-2.51880
H	-2.64105	-0.71024	-3.11218
H	-2.10958	-2.33019	-2.59847
C	1.33265	-0.90713	-0.49438
H	0.73796	-0.66099	0.56240
C	0.53295	-0.43910	-1.67618
H	0.22722	-1.23371	-2.36789
H	0.90528	0.46498	-2.17115
C	-0.49267	5.31843	0.04987
H	-1.58336	5.25409	-0.09344
H	-0.30677	5.83872	1.00723
H	-0.07921	5.95557	-0.75177
C	1.44175	-2.43746	-0.40716
O	1.69661	-2.83408	0.87387
O	1.30880	-3.21699	-1.33991
C	1.87681	-4.26138	1.03936
H	2.08365	-4.40736	2.10781
H	0.96787	-4.80572	0.73784
H	2.72168	-4.61727	0.42815

TS(E-G)$_{gem}$

SCF (BP86) Energy = -1302.67990977
Enthalpy 0K = -1302.218874
Energy 298K = -1302.188009
Free Energy 298K = -1302.278896
Lowest Frequency = -472.1368 cm^{-1}
SCF (DCE) Energy = -1302.69020680
SCF (BP86-D3) Energy = -1302.76940008
SCF (BS2) Energy = -1302.64857294

Rh	-0.75076	-0.04464	-0.09950
C	-2.71329	-0.76691	-0.91737
C	-2.34705	-1.60250	0.20779
C	-2.32679	-0.76861	1.40875
C	-2.62657	0.57181	1.01735
C	-2.84925	0.58819	-0.43963
C	2.70645	1.01915	0.04796
C	4.01245	1.48324	0.36054
C	2.55922	-0.37500	-0.22627
C	5.12758	0.64353	0.36299
H	4.13984	2.53764	0.61942
C	3.69528	-1.21047	-0.22111
C	4.97491	-0.71754	0.05687
H	6.11403	1.05126	0.60767
H	3.57923	-2.27332	-0.45363
H	5.83699	-1.39173	0.04253
C	1.76677	3.42543	-0.00633
C	0.45812	3.93024	-0.03523
H	2.69310	3.99741	-0.02938
N	0.27278	1.75353	-0.00730
N	-0.43635	2.90296	-0.03620
C	1.62671	2.01667	0.00944
C	-2.21678	-3.10017	0.17731
H	-1.65881	-3.44851	-0.70720
H	-1.69440	-3.47175	1.07349
H	-3.21449	-3.57789	0.15671
C	-2.09741	-1.26425	2.81102
H	-1.34581	-2.06919	2.84138
H	-1.74500	-0.45568	3.46983
H	-3.03343	-1.66602	3.24302
C	-2.75407	1.78027	1.89545
H	-2.40476	1.57976	2.92035
H	-2.15514	2.60620	1.47021
H	-3.80922	2.10709	1.95194
C	-3.26859	1.80108	-1.21636
H	-4.29290	2.11266	-0.93712
H	-2.57361	2.63186	-0.99644
H	-3.25640	1.61082	-2.30125
C	-2.96813	-1.25415	-2.31698
H	-4.01616	-1.59214	-2.42301
H	-2.79674	-0.45955	-3.05975
H	-2.31905	-2.10661	-2.57467
C	1.21566	-0.98351	-0.60077
H	0.60112	-0.61712	0.64969
C	0.48737	-0.42204	-1.74906
H	0.04067	-1.14676	-2.43841
H	0.90251	0.47814	-2.21324
C	-0.02118	5.35475	-0.05689
H	-1.11311	5.38186	-0.20228
H	0.20817	5.88319	0.88650
H	0.44734	5.93085	-0.87426
C	1.10930	-2.50769	-0.48639
O	1.43526	-2.93927	0.76609
O	0.76587	-3.26255	-1.38820
C	1.43003	-4.37743	0.93138
H	1.72734	-4.55120	1.97407
H	0.42762	-4.78957	0.73632
H	2.14699	-4.84826	0.23955

G$_{gem}$

SCF (BP86) Energy = -1302.69450751
Enthalpy 0K = -1302.231353
Energy 298K = -1302.200409
Free Energy 298K = -1302.290886
Lowest Frequency = 24.7987 cm^{-1}
SCF (DCE) Energy = -1302.70479787
SCF (BP86-D3) Energy = -1302.78556209
SCF (BS2) Energy = -1302.66100685

C	1.97280	1.70740	-0.01083
C	2.36022	3.06417	-0.10857
C	1.15887	3.79324	-0.13127
N	0.09628	2.94429	-0.05095
N	0.60088	1.69411	0.01860
C	0.93870	5.27761	-0.21983
Rh	-0.65285	0.07016	0.04681
C	0.39068	-0.41066	-1.77130
C	1.02289	-1.15800	-0.72765
C	0.53877	-2.58285	-0.59525
O	0.91751	-3.16777	0.58058
C	2.82991	0.51783	0.06074
C	4.18093	0.72273	0.44138
C	5.11186	-0.31697	0.48671

C   4.70666 -1.61702 0.15024
C   3.37256 -1.84882 -0.20210
C   2.40833 -0.81494 -0.24387
C -2.74279 -0.29527 -0.92805
C -2.58025 -1.19248 0.20923
C -2.41268 -0.37935 1.39079
C -2.43783 1.01317 0.98526
C -2.67177 1.05758 -0.45865
C -3.03931 -0.75596 -2.32720
C -2.79266 -2.67820 0.16919
C -2.36103 -0.88516 2.80801
C -2.39487 2.22099 1.87546
C -2.83971 2.31949 -1.25147
O -0.12711 -3.18122 -1.44328
H   4.49026 1.73382 0.72150
H   6.14523 -0.11311 0.78633
H   3.07059 -2.86636 -0.46157
H   5.41853 -2.44864 0.16541
H   3.37395 3.45441 -0.18789
H -2.17782 -3.16125 -0.60950
H -2.54595 -3.14199 1.13749
H -3.85339 -2.90678 -0.04653
H -1.84561 -1.85539 2.87612
H -1.83505 -0.17964 3.46881
H -3.38579 -1.01688 3.20312
H -2.05158 1.96571 2.89013
H -1.70390 2.96842 1.44811
H -3.40020 2.67461 1.96032
H -3.77178 2.84043 -0.96361
H -1.98544 2.99229 -1.04934
H -2.88566 2.11694 -2.33314
H -4.11465 -0.99409 -2.43163
H -2.79532 0.01577 -3.07369
H -2.47649 -1.67100 -2.57552
H   0.20719 -0.24483 1.31286
H -0.29566 -0.94480 -2.43477
H   0.89387 0.46753 -2.18488
H -0.13903 5.49434 -0.29489
H   1.32999 5.80656 0.66813
H   1.43753 5.71326 -1.10370
C   0.55536 -4.56071 0.71046
H   1.03168 -4.89810 1.64115
H -0.53787 -4.67973 0.77419
H   0.92347 -5.14244 -0.14997

D2$_{1,2}$
SCF Energy = -1302.69141234
Enthalpy 0K = -1302.227649
Energy 298K = -1302.195640
Free Energy 298K = -1302.289696
Lowest Frequency = 20.0426 cm^{-1}
SCF (DCE) Energy = -1302.70370532
SCF (BP86-D3) Energy = -1302.78743056
SCF (BS2) Energy = -1302.66101263

Rh 0.50635 -0.36962 -0.18789
C   1.44450 -2.30492 0.59073
C   2.36432 -1.73360 -0.39938
C   2.79353 -0.45301 0.06677
C   2.12805 -0.21200 1.35211
C   1.36097 -1.38716 1.69862
C -1.62878 1.36887 0.94630
C -2.78956 1.72734 1.66372
C -1.30052 -0.00962 0.76931
C -3.62415 0.73530 2.20123
H -3.03315 2.78747 1.79821
C -2.15077 -0.98900 1.29978
C -3.30994 -0.62082 2.01826
H -4.52285 1.02169 2.75823
H -1.94422 -2.05191 1.13363
H -3.96463 -1.40057 2.42344
C -0.50441 3.67927 0.17474
C   0.70041 3.79294 -0.55816
H -1.14862 4.48391 0.52773
N   0.37076 1.67774 -0.27113
N   1.22268 2.55814 -0.82728
C -0.68783 2.29431 0.34058
C   0.13835 -0.22142 -2.30740
C -0.60333 -1.33806 -1.84621
H -0.17955 -2.34547 -1.92720
H -0.37416 0.71759 -2.52837
H   1.09890 -0.36805 -2.81361
C -2.09015 -1.40007 -1.75883
C   0.67027 -1.63536 3.00737
H   1.34926 -2.17351 3.69528
H   0.37246 -0.69355 3.49224
H -0.23813 -2.24431 2.88304
C   0.88260 -3.69914 0.55436
H   1.60276 -4.42036 0.98555
H -0.05099 -3.77506 1.13342
H   0.66479 -4.02885 -0.47401
C   2.82233 -2.43917 -1.64502
H   3.50681 -3.26893 -1.39013
H   1.98096 -2.88050 -2.20711
H   3.36051 -1.75870 -2.32253
C   3.75749 0.49919 -0.58063
H   4.12741 0.10984 -1.54222
H   3.25919 1.47010 -0.76860
H   4.63265 0.67131 0.07200
C   2.35686 0.99854 2.21037
H   3.29378 0.88951 2.78835
H   2.44004 1.90319 1.58757
H   1.53329 1.15024 2.92467
C   1.40830 5.02924 -1.03939
H   1.69411 5.69387 -0.20409
H   2.32479 4.74728 -1.58174
H   0.77624 5.62275 -1.72434
O -2.70618 -2.42815 -1.48506
O -2.68850 -0.21287 -2.05239
C -4.12530 -0.20916 -1.88340
H -4.45733 0.77566 -2.23838
H -4.58731 -1.01682 -2.47307
H -4.37863 -0.34260 -0.81946

TS(D2-E2)$_{1,2}$
SCF Energy = -1302.65968108
Enthalpy 0K = -1302.196638
Energy 298K = -1302.165377
Free Energy 298K = -1302.257528
Lowest Frequency = -257.8935 cm^{-1}
SCF (DCE) Energy = -1302.67152629
SCF (BP86-D3) Energy = -1302.75149784
SCF (BS2) Energy = -1302.62973413

Rh 0.82856 -0.06116 -0.29106
C   2.68843 -1.32187 -0.07019
C   2.94192 -0.25264 -1.01373
C   2.74357 1.00541 -0.33153
C   2.43284 0.69721 1.07723
C   2.41968 -0.72329 1.23656
C -1.80201 -0.27266 1.12686
C -2.67552 -0.77179 2.11588
C -0.90131 -1.18031 0.44977
C -2.64491 -2.12331 2.48574
H -3.35007 -0.07670 2.62822
C -0.88270 -2.53701 0.86316
C -1.72860 -3.00308 1.87776
H -3.32712 -2.49127 3.25933
H -0.22351 -3.24528 0.34747
H -1.69592 -4.05710 2.17305
C -2.31609 2.34441 1.07585
C -1.48944 3.33246 0.50243
H -3.26192 2.48586 1.59817
N -0.50328 1.44127 0.11694

N -0.38257 2.76368 -0.07570
C -1.64738 1.13096 0.81122
C -0.12779 -0.15209 -2.15201
C -0.88560 -1.20707 -1.46967
H -0.50089 -2.22169 -1.62467
H -0.66502 0.76612 -2.40973
H   0.59849 -0.48544 -2.90459
C -2.39877 -1.26318 -1.62973
C   2.18766 -1.49335 2.50539
H   3.11694 -1.98950 2.84165
H   1.84686 -0.83368 3.31811
H   1.41890 -2.27402 2.36983
C   2.87981 -2.79212 -0.32660
H   3.92998 -3.09124 -0.14406
H   2.24697 -3.40272 0.33714
H   2.64019 -3.06212 -1.36817
C   3.39080 -0.42839 -2.43799
H   4.49246 -0.51175 -2.48970
H   2.97282 -1.34320 -2.89005
H   3.09251 0.42595 -3.06588
C   2.92447 2.39370 -0.87713
H   3.02806 2.38305 -1.97380
H   2.04639 3.01647 -0.62760
H   3.82990 2.87091 -0.45668
C   2.21102 1.74724 2.12722
H   3.16996 2.21720 2.41505
H   1.54665 2.53989 1.74233
H   1.75212 1.32714 3.03554
C -1.68540 4.82177 0.44101
H -1.90657 5.24719 1.43581
H -0.77249 5.30246 0.05479
H -2.52142 5.10208 -0.22590
O -3.03428 -2.30938 -1.64451
O -2.94604 -0.03304 -1.80350
C -4.38871 -0.03167 -1.91421
H -4.67001 1.02444 -2.01783
H -4.71009 -0.61225 -2.79367
H -4.84195 -0.47002 -1.01083

E2$_{1,2}$
SCF (BP86) Energy = -1302.67535227
Enthalpy 0K = -1302.210448
Energy 298K = -1302.178645
Free Energy 298K = -1302.274259
Lowest Frequency = 16.1417 cm^{-1}
SCF (DCE) Energy = -1302.68925510
SCF (BP86-D3) Energy = -1302.75730620
SCF (BS2) Energy = -1302.64639402

C -1.15582 1.85924 0.12800
C -1.33156 3.18039 -0.31634
C -0.08231 3.55727 -0.86697
N   0.80922 2.54418 -0.74624
N   0.15473 1.51849 -0.12577
C   0.31789 4.85173 -1.51667
Rh 1.03613 -0.29230 0.03678
C -0.56681 -0.89821 -1.12747
C -1.74188 -1.32176 -0.22076
C -2.91408 -1.76159 -1.12461
O -3.44027 -0.70574 -1.80661
C -2.07394 1.04132 0.95394
C -2.77834 1.75192 1.96311
C -3.64701 1.12364 2.85915
C -3.82150 -0.26726 2.78638
C -3.14045 -0.98500 1.79782
C -2.28079 -0.36581 0.85927
C   2.41766 -1.88028 0.74485
C   2.51644 -1.72178 -0.67250
C   2.90903 -0.31936 -0.91058
C   3.23795 0.29931 0.39070
C   2.88799 -0.63221 1.39370
C   2.04057 -3.13112 1.48745
C   2.32182 -2.77419 -1.72519
C   3.15227 0.34025 -2.23376
C   3.72552 1.70830 0.54444
C   2.95173 -0.43745 2.88113
O -3.31592 -2.91272 -1.24230
H -2.59924 2.82881 2.04405
H -4.16591 1.71304 3.62260
H -3.29525 -2.06810 1.71880
H -4.48103 -0.78808 3.48841
H -2.24330 3.77425 -0.25516
H -0.86520 -0.07531 -1.79443
H -0.27574 -1.76667 -1.74543
H   3.75964 -1.04975 3.32396
H   3.13888 0.61455 3.14424
H   2.00906 -0.74263 3.36704
H   3.83340 1.98809 1.60402
H   4.70773 1.84110 0.05530
H   3.00194 2.40012 0.06998
H   2.94306 -3.69001 1.80162
H   1.46481 -2.90118 2.39913
H   1.43147 -3.80446 0.86413
H   3.28609 -3.25150 -1.98152
H   1.63641 -3.56757 -1.38637
H   1.90619 -2.34742 -2.65246
H   2.67211 -0.21105 -3.05690
H   2.74567 1.36613 -2.21470
H   4.23770 0.39715 -2.44518
H -1.46025 -2.25655 0.29512
H -0.32474 5.09006 -2.38307
H   0.24544 5.70316 -0.81575
H   1.35950 4.78757 -1.86972
C -4.53797 -1.04534 -2.68327
H -4.84102 -0.09967 -3.15277
H -4.21706 -1.77379 -3.44565
H -5.37291 -1.47968 -2.10987

TS(E2-F2)$_{1,2}$
SCF Energy = -1302.60432357
Enthalpy 0K = -1302.142500
Energy 298K = -1302.110854
Free Energy 298K = -1302.204741
Lowest Frequency = -525.3072 cm^{-1}
SCF (DCE) Energy = -1302.61485797
SCF (BP86-D3) Energy = -1302.69264945
SCF (BS2) Energy = -1302.57184667

Rh 0.77073 -0.03732 -0.23113
C   2.63556 -1.24017 -0.06098
C   2.62381 -0.61662 -1.36820
C   2.59983 0.80541 -1.13009
C   2.82838 1.04807 0.31900
C   2.87818 -0.19878 0.96282
C -0.98889 -0.48860 1.11852
C -0.76638 -1.11032 2.39212
C -1.06191 -1.32738 -0.08526
C -0.58790 -2.48007 2.49664
H -0.74324 -0.47082 3.28118
C -0.89936 -2.74458 0.08941
C -0.65085 -3.30457 1.33123
H -0.41651 -2.93682 3.47695
H -1.01042 -3.38971 -0.79053
H -0.53372 -4.38910 1.42725
C -1.84595 2.01890 1.70116
C -1.55224 3.15495 0.88382
H -2.34146 2.00713 2.67111
N -0.76707 1.45662 -0.16901
N -0.89831 2.80419 -0.25290
C -1.33996 0.94014 0.97775
C -1.75062 -0.78252 -1.34152
C -1.01484 0.47689 -1.78224
H -1.66277 1.29457 -2.12089
H -0.20489 0.30028 -2.50374

C   3.05964 -0.49197 2.42342
H   4.00250 -1.03997 2.61163
H   3.08508 0.43166 3.02367
H   2.23672 -1.12245 2.80964
C   2.94812 2.41719 0.92650
H   2.97749 2.37067 2.02687
H   3.86777 2.93061 0.58708
H   2.09286 3.05445 0.64087
C   2.70987 -2.71395 0.22211
H   3.74384 -3.01434 0.47908
H   2.05978 -2.99442 1.06882
H   2.39314 -3.30988 -0.64846
C   2.66344 -1.30547 -2.70495
H   3.70001 -1.40283 -3.08320
H   2.23832 -2.32135 -2.64956
H   2.09365 -0.74947 -3.47004
C   2.61620 1.88906 -2.17460
H   2.16709 1.54691 -3.12185
H   2.05613 2.77677 -1.83870
H   3.65185 2.21013 -2.39965
H -1.63210 -1.54198 -2.14096
C -3.26469 -0.54655 -1.21277
C -1.90318 4.59436 1.13253
H -2.99692 4.74345 1.17867
H -1.48395 4.95538 2.08857
H -1.50512 5.22050 0.31926
O -3.85471 -1.50546 -0.44833
O -3.87413 0.36547 -1.75539
C -5.28798 -1.35858 -0.30707
H -5.53076 -0.39936 0.17707
H -5.78110 -1.39355 -1.29190
H -5.60774 -2.20200 0.31946

F2$_{1,2}$
SCF Energy = -1302.67428568
Enthalpy 0K = -1302.209089
Energy 298K = -1302.177416
Free Energy 298K = -1302.272175
Lowest Frequency = 11.5336 cm^{-1}
SCF (DCE) Energy = -1302.68412334
SCF (BP86-D3) Energy = -1302.76341479
SCF (BS2) Energy = -1302.64233818

Rh 0.69017 -0.27691 -0.10568
C   1.80215 -1.57411 -1.56746
C   2.51168 -0.33951 -1.37033
C   2.96938 -0.26856 0.02956
C   2.49292 -1.42848 0.70470
C   1.70294 -2.20655 -0.25573
C -0.93027 0.21387 1.26475
C -1.02581 -0.36288 2.58169
C -1.46561 -0.50337 0.08492
C -1.53478 -1.63513 2.74163
H -0.69330 0.22492 3.44399
C -1.97530 -1.83686 0.33997
C -1.98588 -2.38882 1.60493
H -1.60420 -2.07451 3.74225
H -2.40274 -2.39133 -0.50369
H -2.37544 -3.40170 1.75347
C   0.32039 2.55541 1.61409
C   0.82151 3.38224 0.57614
H   0.46329 2.67520 2.68619
N -0.21587 1.76156 -0.45281
N   0.49559 2.94826 -0.66045
C -0.33902 1.50246 0.96104
C   2.82625 0.70447 -2.40553
H   2.30994 0.49780 -3.35640
H   2.51032 1.70683 -2.06244
H   3.91204 0.74435 -2.61329
C   3.81775 0.83713 0.59167
H   4.87790 0.72566 0.29295
H   3.47575 1.82212 0.23234
H   3.78214 0.85372 1.69297
C   2.72400 -1.81845 2.13727
H   3.46578 -2.63599 2.21843
H   3.09972 -0.96914 2.73055
H   1.79139 -2.17042 2.61154
C   1.11198 -3.55828 0.03044
H   1.90408 -4.33002 0.07785
H   0.57329 -3.56711 0.99276
H   0.39654 -3.85919 -0.75098
C   1.30989 -2.14369 -2.86992
H   2.05364 -2.83057 -3.31941
H   0.37628 -2.71480 -2.73573
H   1.10774 -1.34997 -3.60764
C -2.14018 0.30181 -1.05738
H -2.14645 -0.34389 -1.95133
C -1.39934 1.62428 -1.34360
H -2.06029 2.49004 -1.16868
H -1.01342 1.66613 -2.37295
C -3.59571 0.58144 -0.66472
C   1.63793 4.63673 0.72199
H   1.16719 5.33048 1.43922
H   2.65384 4.41960 1.09698
H   1.72865 5.14284 -0.25125
O -4.41083 -0.42761 -1.09353
O -3.98955 1.53776 -0.00943
C -5.78724 -0.30664 -0.66106
H -6.22133 0.64364 -1.01041
H -6.31180 -1.16218 -1.10790
H -5.85108 -0.34250 0.43828

TS(E2-E$_{gem}$)$_{1,2}$
SCF (BP86) Energy = -1302.67241209
Enthalpy 0K = -1302.207988
Energy 298K = -1302.177217
Free Energy 298K = -1302.268615
Lowest Frequency = -37.3172 cm^{-1}
SCF (DCE) Energy = -1302.68483269
SCF (BP86-D3) Energy = -1302.75557957
SCF (BS2) Energy = -1302.64246721

C -0.86623 2.23253 -0.17641
C -0.67738 3.53692 -0.67428
C   0.70283 3.64346 -0.94418
N   1.33080 2.48627 -0.62567
N   0.38037 1.62927 -0.14889
C   1.47750 4.80451 -1.50071
Rh 0.93877 -0.31444 0.08091
C -0.67611 -0.67085 -1.12880
C -1.72907 -0.87613 -0.00306
C -2.60608 -2.04486 -0.48086
O -3.48443 -1.64540 -1.44257
C -2.11765 1.69552 0.40561
C -2.98828 2.67615 0.96150
C -4.20030 2.35671 1.57368
C -4.58169 1.01069 1.68326
C -3.74116 0.02865 1.15325
C -2.52934 0.33161 0.49003
C   2.00156 -2.13129 0.78902
C   2.26462 -1.89603 -0.59801
C   2.88546 -0.56314 -0.69003
C   3.16498 -0.08808 0.68294
C   2.58397 -1.01600 1.57416
C   1.37173 -3.35892 1.38658
C   2.01696 -2.83922 -1.73994
C   3.37179 0.12800 -1.92674
C   3.84945 1.20824 0.99222
C   2.54166 -0.93276 3.07334
O -2.48168 -3.20935 -0.11763
H -2.66517 3.72039 0.93199
H -4.83005 3.15241 1.98554
H -4.03658 -1.02299 1.24766
H -5.51564 0.72725 2.17928

H -1.44379 4.29375 -0.83676
H -0.94847 0.15196 -1.80561
H -0.53130 -1.59771 -1.70880
H   3.24023 -1.66098 3.52690
H   2.81857 0.07066 3.43056
H   1.53578 -1.16239 3.46368
H   3.87254 1.40976 2.07460
H   4.89205 1.19968 0.62475
H   3.30838 2.03246 0.48797
H   2.14139 -4.11625 1.63049
H   0.83861 -3.12283 2.32194
H   0.64638 -3.82211 0.69951
H   2.91361 -3.45647 -1.93609
H   1.18183 -3.52506 -1.52465
H   1.77744 -2.29600 -2.66840
H   2.94176 -0.31709 -2.83739
H   3.08247 1.19299 -1.88281
H   4.47466 0.06448 -1.99964
H -1.21890 -1.31225 0.89046
H   1.06440 5.14460 -2.46696
H   1.46306 5.67362 -0.81807
H   2.52845 4.51437 -1.65959
C -4.28141 -2.71181 -2.00701
H -4.93401 -2.22786 -2.74616
H -3.63816 -3.46564 -2.48954
H -4.87995 -3.20724 -1.22559

E_{gem}'
SCF (BP86) Energy = -1302.68094312
Enthalpy 0K = -1302.217797
Energy 298K = -1302.186442
Free Energy 298K = -1302.279212
Lowest Frequency = 25.7455 cm^{-1}
SCF (DCE) Energy = -1302.69160697
SCF (BP86-D3) Energy = -1302.76911510
SCF (BS2) Energy = -1302.65017963

C -1.44144 -2.43762 -0.40673
C -1.33260 -0.90729 -0.49423
C -0.53305 -0.43935 -1.67614
Rh 0.80066 -0.10723 -0.12885
N -0.11500 1.75345 -0.00383
N   0.68993 2.84043 -0.00100
C -0.10959 3.94115 0.03208
C -1.45659 3.55250 0.04827
C -1.44470 2.13667 0.02163
C -2.62528 1.25436 0.04040
C -3.88522 1.85847 0.30378
C -5.08325 1.14200 0.28908
C -5.07096 -0.23423 0.01571
C -3.84316 -0.86537 -0.21298
C -2.62800 -0.15287 -0.20291
C   2.23719 -1.78483 0.17636
C   2.63388 -1.00065 -0.96783
C   2.88840 0.35190 -0.50215
C   2.75894 0.35427 0.97007
C   2.35055 -0.94316 1.37739
C   1.93686 -3.25862 0.17550
C   2.80826 -1.50733 -2.37249
C   3.37372 1.52091 -1.30595
C   3.01747 1.55664 1.82589
C   2.11213 -1.41598 2.78520
H -3.91216 2.92568 0.53707
H -6.02624 1.65943 0.49472
H -3.83222 -1.93971 -0.42305
H -5.99983 -0.81241 -0.01175
H -2.32592 4.20793 0.04815
C   0.49226 5.31838 0.05044
H -0.90541 0.46469 -2.17113
H -0.22728 -1.23402 -2.36775
H   2.99306 -1.96353 3.17050
H   1.91814 -0.57241 3.46544

H   1.24891 -2.09870 2.84524
H   2.73046 1.38269 2.87476
H   4.08998 1.82567 1.80486
H   2.43638 2.41197 1.43353
H   2.86938 -3.85028 0.24507
H   1.30762 -3.53725 1.03634
H   1.41022 -3.56886 -0.74133
H   3.83490 -1.89242 -2.51930
H   2.11037 -2.32936 -2.59902
H   2.64118 -0.70908 -3.11238
H   3.28749 1.33205 -2.38759
H   2.76600 2.40874 -1.05343
H   4.43462 1.74035 -1.08035
H -0.73797 -0.66073 0.56250
H   1.58343 5.25377 -0.08901
H   0.08167 5.95441 -0.75357
H   0.30300 5.84003 1.00639
O -1.69659 -2.83410 0.87427
O -1.30807 -3.21727 -1.33934
C -1.87656 -4.26145 1.03992
H -0.96723 -4.80561 0.73925
H -2.08421 -4.40723 2.10824
H -2.72083 -4.61769 0.42808

TS(E-G)$_{gem}'$
SCF (BP86) Energy = -1302.67990972
Enthalpy 0K = -1302.218874
Energy 298K = -1302.188009
Free Energy 298K = -1302.278894
Lowest Frequency = -472.2142 cm^{-1}
SCF (DCE) Energy = -1302.69020644
SCF (BP86-D3) Energy = -1302.76939962
SCF (BS2) Energy = -1302.64857320

C -1.10938 -2.50766 -0.48641
C -1.21568 -0.98347 -0.60079
C -0.48736 -0.42203 -1.74906
Rh 0.75075 -0.04466 -0.09947
N -0.27275 1.75354 -0.00731
N   0.43640 2.90297 -0.03606
C -0.45804 3.93027 -0.03501
C -1.76670 3.42548 -0.00615
C -1.62667 2.01672 0.00946
C -2.70643 1.01921 0.04797
C -4.01242 1.48331 0.36055
C -5.12756 0.64362 0.36299
C -4.97492 -0.71744 0.05683
C -3.69530 -1.21039 -0.22116
C -2.55922 -0.37493 -0.22629
C   2.34703 -1.60255 0.20771
C   2.71330 -0.76689 -0.91740
C   2.84927 0.58817 -0.43957
C   2.62656 0.57171 1.01741
C   2.32677 -0.76874 1.40872
C   2.21675 -3.10021 0.17712
C   2.96815 -1.25406 -2.31703
C   3.26864 1.80109 -1.21623
C   2.75407 1.78010 1.89559
C   2.09738 -1.26447 2.81095
H -4.13980 2.53771 0.61945
H -6.11401 1.05137 0.60767
H -3.57926 -2.27322 -0.45371
H -5.83701 -1.39162 0.04247
H -2.69303 3.99747 -0.02910
C   0.02134 5.35474 -0.05717
H -0.90246 0.47817 -2.21325
H -0.04065 -1.14674 -2.43840
H   3.03341 -1.66625 3.24294
H   1.74495 -0.45595 3.46981
H   1.34580 -2.06943 2.84125
H   2.40481 1.57951 2.92048
H   3.80922 2.10695 1.95206

H	2.15511	2.60605	1.47043
H	3.21446	-3.57795	0.15674
H	1.69415	-3.47184	1.07315
H	1.65898	-3.44848	-0.70754
H	4.01616	-1.59217	-2.42303
H	2.31899	-2.10642	-2.57483
H	2.79690	-0.45938	-3.05975
H	3.25643	1.61091	-2.30112
H	2.57369	2.63188	-0.99625
H	4.29297	2.11262	-0.93698
H	-0.60111	-0.61712	0.64970
H	1.11431	5.38138	-0.19463
H	-0.44101	5.92863	-0.87964
H	-0.21505	5.88578	0.88297
O	-0.76602	-3.26253	-1.38824
O	-1.43532	-2.93923	0.76607
C	-1.43014	-4.37739	0.93136
H	-1.72736	-4.55115	1.97408
H	-2.14717	-4.84819	0.23960
H	-0.42775	-4.78957	0.73621
H	2.17726	-3.16143	-0.60974
H	4.11467	-0.99364	-2.43195
H	2.47674	-1.67115	-2.57565
H	2.79487	0.01576	-3.07375
H	2.88602	2.11689	-2.33292
H	1.98574	2.99219	-1.04911
H	3.77206	2.84017	-0.96323
H	-0.20705	-0.24484	1.31295
H	0.13957	5.49421	-0.29750
H	-1.43917	5.71395	-1.10188
H	-1.32668	5.80631	0.66970
O	0.12659	-3.18132	-1.44323
O	-0.91788	-3.16763	0.58069
C	-0.55602	-4.56065	0.71055
H	-1.03277	-4.89807	1.64101
H	-0.92385	-5.14221	-0.15011
H	0.53717	-4.67985	0.77472

G_{gem}'

SCF (BP86) Energy = -1302.69450743
Enthalpy 0K = -1302.231354
Energy 298K = -1302.200409
Free Energy 298K = -1302.290887
Lowest Frequency = 24.7919 cm^{-1}
SCF (DCE) Energy = -1302.70479829
SCF (BP86-D3) Energy = -1302.78556109
SCF (BS2) Energy = -1302.66100729

C	-0.53913	-2.58280	-0.59518
C	-1.02302	-1.15788	-0.72760
C	-0.39070	-0.41065	-1.77128
Rh	0.65285	0.07006	0.04678
N	-0.60068	1.69415	0.01867
N	-0.09589	2.94427	-0.05090
C	-1.15835	3.79336	-0.13131
C	-2.35981	3.06446	-0.10869
C	-1.97259	1.70764	-0.01087
C	-2.82986	0.51819	0.06069
C	-4.18087	0.72327	0.44127
C	-5.11193	-0.31631	0.48662
C	-4.70686	-1.61643	0.15025
C	-3.37278	-1.84840	-0.20205
C	-2.40842	-0.81464	-0.24385
C	2.58019	-1.19277	0.20905
C	2.74275	-0.29544	-0.92812
C	2.67187	1.05737	-0.45856
C	2.43790	1.01281	0.98534
C	2.41266	-0.37978	1.39071
C	2.79238	-2.67853	0.16883
C	3.03925	-0.75593	-2.32734
C	2.83998	2.31933	-1.25124
C	2.39510	2.22047	1.87576
C	2.36101	-0.88570	2.80788
H	-4.49008	1.73442	0.72132
H	-6.14529	-0.11231	0.78618
H	-3.07094	-2.86600	-0.46147
H	-5.41882	-2.44796	0.16543
H	-3.37348	3.45484	-0.18811
C	-0.93800	5.27771	-0.21963
H	-0.89378	0.46759	-2.18489
H	0.29550	-0.94494	-2.43479
H	3.38577	-1.01727	3.20304
H	1.83488	-0.18032	3.46870
H	1.84576	-1.85603	2.87589
H	2.05117	1.96516	2.89019
H	3.40062	2.67356	1.96122
H	1.70470	2.96832	1.44824
H	3.85302	-2.90724	-0.04721
H	2.54587	-3.14235	1.13715

Styrene (Y = Ph)

C_2H_3Ph (b)
SCF Energy = -309.639885918
Enthalpy 0K = -309.510361
Energy 298K = -309.503382
Free Energy 298K = -309.541912
Lowest Frequency = 46.6645 cm^{-1}
SCF (DCE) Energy = -309.642765739
SCF (BP86-D3) Energy = -309.650259658
SCF (BS2) Energy = -309.716310495

C   1.96059 -0.53390 -0.00011
C   2.99139 0.33709 0.00019
H   4.02589 -0.01751 0.00019
H   2.19369 -1.60810 -0.00051
H   2.85049 1.42309 0.00049
C   0.51989 -0.22310 -0.00011
C -0.40931 -1.28990 -0.00001
C   0.01169 1.09880 -0.00011
C -1.79091 -1.05150 0.00009
H -0.03411 -2.32020 -0.00001
C -1.36681 1.33840 -0.00001
H   0.70459 1.94660 -0.00031
C -2.27651 0.26510 0.00009
H -2.48891 -1.89560 0.00019
H -1.73721 2.36920 -0.00001
H -3.35461 0.45660 0.00009

2,1-insertion

D2$_{2,1}$
SCF Energy = -1305.86013978
Enthalpy 0K = -1305.359570
Energy 298K = -1305.327306
Free Energy 298K = -1305.422763
Lowest Frequency = 9.6697 cm^{-1}
SCF (BS2) Energy = -1305.81482274
SCF (DCE) Energy = -1305.87131453
SCF (BP86-D3) Energy = -1305.96125227

Rh -0.81838 -0.19476 -0.27304
C -3.04278 -0.48880 -0.66171
C -2.43528 -1.82342 -0.59853
C -1.88084 -2.00486 0.70390
C -2.13734 -0.77550 1.46311
C -2.90921 0.12239 0.63747
C   0.68536 2.15637 0.74235
C   1.02227 3.51099 0.95123
C -0.35876 1.81631 -0.17246
C   0.34273 4.52595 0.26036
H   1.82201 3.76388 1.65684
C -1.01596 2.84421 -0.86625
C -0.67396 4.19672 -0.65015
H   0.61234 5.57428 0.42821
H -1.80305 2.60291 -1.59205
H -1.20036 4.98515 -1.20015
C   2.32180 0.74256 2.32844
C   2.33347 -0.66730 2.43615
H   2.95460 1.45673 2.85445
N   0.80372 -0.19187 0.98652
N   1.39599 -1.22415 1.61004
C   1.31703 1.01530 1.38218
C -0.36397 0.01832 -2.37740
C   0.57073 -0.92993 -1.88192
H -1.24216 -0.33486 -2.93041
H -0.03467 1.02418 -2.65277
H   0.29905 -1.99203 -1.95287
C   2.02587 -0.70764 -1.74159
C   2.86942 -1.83184 -1.59082
C   2.62258 0.57310 -1.81289
C   4.26209 -1.68899 -1.53320
H   2.41947 -2.82879 -1.52189
C   4.01372 0.71620 -1.75075
H   1.99152 1.46271 -1.90109
C   4.84081 -0.41264 -1.61547
H   4.89559 -2.57537 -1.42162
H   4.45617 1.71654 -1.80531
H   5.92880 -0.29608 -1.57063
C -1.77008 -0.58316 2.90612
H -2.50042 -1.09575 3.56024
H -0.77188 -1.00371 3.10854
H -1.75561 0.48188 3.18359
C -3.51429 1.42658 1.06947
H -4.56630 1.27777 1.37722
H -2.96989 1.85913 1.92252
H -3.49868 2.17200 0.25900
C -3.87714 0.03266 -1.79870
H -4.91973 -0.32911 -1.71672
H -3.90943 1.13387 -1.80503
H -3.49555 -0.30111 -2.77776
C -2.47546 -2.83195 -1.71236
H -3.50603 -3.20098 -1.86701
H -2.14114 -2.40429 -2.67435
H -1.83840 -3.70243 -1.49262
C -1.17980 -3.21057 1.26089
H -1.06848 -4.00167 0.50242
H -0.17094 -2.92443 1.61641
H -1.74656 -3.63519 2.10974
C   3.21470 -1.54912 3.27657
H   4.26285 -1.53674 2.92538
H   3.22214 -1.23324 4.33491
H   2.85760 -2.59041 3.22966

TS(D2-E2)$_{2,1}$
SCF Energy = -1305.83522123
Enthalpy 0K = -1305.334712
Energy 298K = -1305.303360
Free Energy 298K = -1305.395523
Lowest Frequency = -261.9172 cm^{-1}
SCF (DCE) Energy = -1305.84516906
SCF (BP86-D3) Energy = -1305.93413065
SCF (BS2) Energy = -1305.78953696

Rh -0.52023 -0.52909 -0.02610
C -1.99152 -2.24946 0.06967
C -0.68435 -2.71906 0.47900
C -0.26811 -1.94756 1.62704
C -1.38227 -1.04788 1.97876
C -2.43992 -1.25150 1.03931
C -1.06516 2.36001 -0.59962
C -1.91804 3.46632 -0.79359
C -1.38424 1.09470 -1.21498
C -3.10025 3.33983 -1.53644
H -1.66509 4.42158 -0.32023
C -2.59542 0.99536 -1.94340
C -3.45151 2.09542 -2.09486
H -3.75416 4.20754 -1.67282
H -2.84042 0.04939 -2.44255
H -4.37762 1.98993 -2.67031
C   0.87632 3.29571 0.97607
C   1.74495 2.51007 1.76270
H   0.82501 4.38276 0.91927
N   0.51598 1.10543 0.65913
N   1.50573 1.17478 1.56138
C   0.08582 2.35621 0.28025
C -0.01287 0.26442 -2.16581
C   0.88833 -0.76849 -1.61582
H   0.66866 -1.78445 -1.97638
C -3.78672 -0.58738 1.03189
H -4.58406 -1.31286 1.27831
H -3.83566 0.22834 1.76940

H -4.01938 -0.15309 0.04382
C -1.36555 -0.12730 3.16471
H -2.15867 0.63391 3.10379
H -1.51192 -0.69877 4.10037
H -0.39517 0.39301 3.23332
C -2.84524 -2.83858 -1.02058
H -3.45227 -3.68107 -0.63667
H -3.54479 -2.09239 -1.43056
H -2.23677 -3.22724 -1.85349
C   0.07923 -3.85265 -0.14557
H -0.18577 -4.80990 0.34114
H -0.14765 -3.95960 -1.21934
H   1.16705 -3.71514 -0.03948
C   0.99846 -2.08550 2.42282
H   1.73652 -2.71019 1.89582
H   1.45471 -1.09186 2.58236
H   0.80024 -2.54944 3.40768
H -0.61758 -0.08633 -3.00988
H   0.49362 1.21133 -2.39225
C   2.34443 -0.57029 -1.41908
C   3.18723 -1.70840 -1.48728
C   2.95908 0.69049 -1.22056
C   4.57811 -1.59990 -1.35882
H   2.73544 -2.69218 -1.66478
C   4.35043 0.79973 -1.10344
H   2.34394 1.59206 -1.14293
C   5.16910 -0.34063 -1.16720
H   5.20166 -2.49867 -1.41971
H   4.79925 1.78718 -0.95122
H   6.25593 -0.24787 -1.07060
C   2.82429 2.94334 2.71547
H   3.63819 3.48399 2.19937
H   2.43440 3.61601 3.50034
H   3.26304 2.05966 3.20511

E2$_{2,1}$
SCF Energy = -1305.87200160
Enthalpy 0K = -1305.369489
Energy 298K = -1305.338057
Free Energy 298K = -1305.429809
Lowest Frequency = 14.9009 cm^{-1}
SCF (DCE) Energy = -1305.88086086
SCF (BP86-D3) Energy = -1305.97448046
SCF (BS2) Energy = -1305.82465330

Rh 0.63787 -0.03779 0.12739
C   0.48333 -0.99938 2.11853
C   1.87376 -1.02507 1.72878
C   2.34102 0.35886 1.62332
C   1.25622 1.23800 1.93175
C   0.08555 0.39754 2.19460
C -2.93216 -0.31275 -0.43358
C -4.10390 -0.42116 0.35374
C -2.46939 -1.46365 -1.13170
C -4.79542 -1.63446 0.47172
H -4.46242 0.46997 0.88056
C -3.17381 -2.67480 -0.99921
C -4.32612 -2.77274 -0.20342
H -5.69820 -1.69048 1.08963
H -2.81953 -3.55369 -1.55239
H -4.85852 -3.72631 -0.12168
C -2.87465 2.25964 -0.72584
C -1.79927 3.16717 -0.83164
H -3.94156 2.46415 -0.81598
N -0.90711 1.18504 -0.57767
N -0.61467 2.50692 -0.72469
C -2.27288 0.99621 -0.56600
C -1.24267 -1.38673 -2.01780
C   0.05424 -1.56952 -1.23526
H -1.22885 -0.42291 -2.55462
H   0.11128 -2.56770 -0.77067
C -1.24678 0.91691 2.65096

H -1.21728 1.11856 3.73904
H -1.50539 1.85553 2.13661
H -2.05310 0.19245 2.46032
C   1.26530 2.73858 1.99326
H   0.94253 3.09662 2.98795
H   2.27262 3.14113 1.80174
H   0.58525 3.15394 1.22707
C -0.37495 -2.18068 2.47474
H -0.33282 -2.38326 3.56199
H -1.42782 -2.00861 2.20207
H -0.04107 -3.09354 1.95566
C   2.75717 -2.23795 1.64948
H   3.28541 -2.40053 2.60834
H   2.17664 -3.15022 1.43562
H   3.52037 -2.12984 0.86216
C   3.75180 0.74824 1.29081
H   4.08014 0.30253 0.33426
H   3.85781 1.84014 1.20289
H   4.44681 0.39804 2.07620
H -1.30147 -2.18655 -2.78481
C   1.34564 -1.08885 -1.73112
C   2.53938 -1.89765 -1.71564
C   1.48366 0.30514 -2.09486
C   3.77522 -1.35785 -2.02804
H   2.44128 -2.96347 -1.48153
C   2.77528 0.83553 -2.37725
H   0.60886 0.91152 -2.33880
C   3.90591 0.02931 -2.33510
H   4.66193 -2.00152 -2.04807
H   2.85595 1.88997 -2.66085
H   4.89063 0.44187 -2.57816
C -1.82060 4.65219 -1.06787
H -2.20642 4.90502 -2.07244
H -2.46066 5.17494 -0.33491
H -0.79940 5.05827 -0.98725

TS(E2-F2)$_{2,1}$
SCF Energy = -1305.77731405
Enthalpy 0K = -1305.278815
Energy 298K = -1305.246803
Free Energy 298K = -1305.341344
Lowest Frequency = -422.6920 cm^{-1}
SCF (DCE) Energy = -1305.78618182
SCF (BP86-D3) Energy = -1305.87189868
SCF (BS2) Energy = -1305.72943222

Rh 0.86718 0.23352 -0.10071
C   1.97139 2.08504 -0.73060
C   1.17804 2.35116 0.44265
C   1.78035 1.63080 1.59415
C   2.86097 0.86957 1.11382
C   2.91728 1.05240 -0.35133
C   0.56747 -1.99651 -0.06684
C   1.66751 -2.84270 0.30250
C   0.46465 -1.51177 -1.45189
C   2.65109 -3.17850 -0.61281
H   1.70541 -3.21975 1.33050
C   1.50745 -1.91241 -2.36031
C   2.57215 -2.70174 -1.95848
H   3.48279 -3.82431 -0.31230
H   1.42030 -1.60516 -3.41005
H   3.34289 -2.98858 -2.68172
C -1.18390 -2.25226 1.98150
C -2.02021 -1.18088 2.43163
H -1.03924 -3.22587 2.44870
N -1.04054 -0.45382 0.65940
N -1.92142 -0.09570 1.62288
C -0.57902 -1.74582 0.83205
C   0.08841 3.38052 0.57882
H -0.36678 3.61988 -0.39683
H -0.71291 3.02825 1.24869
H   0.48268 4.32764 0.99652

C	1.26281	1.73050	3.00140
H	1.42142	2.74301	3.41933
H	0.17874	1.52446	3.03922
H	1.76372	1.01101	3.66876
C	3.77636	-0.04107	1.87864
H	4.81561	0.33972	1.88146
H	3.45826	-0.14963	2.92802
H	3.80328	-1.05087	1.42791
C	3.99807	0.47569	-1.22128
H	4.97780	0.93754	-0.99218
H	4.09817	-0.61323	-1.07054
H	3.78931	0.64231	-2.28992
C	1.87915	2.77779	-2.06328
H	2.53400	3.67077	-2.10423
H	2.18182	2.11173	-2.88824
H	0.85221	3.12130	-2.27566
C	-0.89873	-1.12005	-2.00850
H	-1.59586	-1.97861	-1.97861
H	-0.79771	-0.84431	-3.07769
C	-1.46842	0.08727	-1.28098
H	-0.97118	1.02332	-1.55930
C	-2.92037	0.26074	-1.11491
C	-3.47117	1.56227	-1.18990
C	-3.80268	-0.83503	-0.94593
C	-4.85516	1.76417	-1.12856
H	-2.79651	2.41833	-1.30522
C	-5.18637	-0.63359	-0.89176
H	-3.39601	-1.84492	-0.83161
C	-5.71866	0.66466	-0.98580
H	-5.26222	2.77867	-1.19304
H	-5.85554	-1.49122	-0.76495
H	-6.80191	0.81859	-0.94071
C	-2.94424	-1.15641	3.61590
H	-3.73721	-1.92151	3.53020
H	-2.40383	-1.35392	4.55905
H	-3.42621	-0.16940	3.69098

F2$_{2,1}$

SCF Energy = -1305.84165764
Enthalpy 0K = -1305.339426
Energy 298K = -1305.307777
Free Energy 298K = -1305.402095
Lowest Frequency = 13.3876 cm^{-1}
SCF (DCE) Energy = -1305.84967636
SCF (BP86-D3) Energy = -1305.93694781
SCF (BS2) Energy = -1305.79436816

Rh	1.07852	-0.03678	0.14367
C	2.60506	-0.61584	1.69068
C	2.08869	-1.79225	1.03998
C	2.48448	-1.77006	-0.37925
C	3.17588	-0.54935	-0.61822
C	3.19282	0.20632	0.64129
C	-0.12709	1.33606	-1.03797
C	0.42733	2.09037	-2.13378
C	-0.02645	1.83625	0.35209
C	1.13796	3.24729	-1.88604
H	0.27485	1.73076	-3.15728
C	0.74886	3.04715	0.52304
C	1.32297	3.71485	-0.54079
H	1.56145	3.81793	-2.71925
H	0.83621	3.45835	1.53704
H	1.89792	4.63035	-0.36486
C	-1.15876	-0.86995	-2.15787
C	-1.62316	-2.00834	-1.45039
H	-1.02524	-0.76533	-3.23293
N	-1.12533	-0.55398	0.10055
N	-1.63747	-1.83973	-0.11112
C	-0.81562	0.06488	-1.16634
C	1.33679	-2.92679	1.67874
H	1.08791	-2.70488	2.72863
H	0.38933	-3.12085	1.14252

H	1.93295	-3.85842	1.66284
C	2.19867	-2.87307	-1.35857
H	2.86199	-3.74401	-1.19447
H	1.15843	-3.22803	-1.26411
H	2.34168	-2.53667	-2.39814
C	3.78681	-0.07261	-1.90555
H	4.89187	-0.12565	-1.87277
H	3.45002	-0.68050	-2.76085
H	3.51219	0.97655	-2.11322
C	3.92421	1.50441	0.83689
H	5.01900	1.34120	0.84266
H	3.69314	2.22095	0.03070
H	3.65090	1.98131	1.79137
C	2.60526	-0.31779	3.16532
H	3.53645	-0.66994	3.65140
H	2.52375	0.76443	3.36024
H	1.76167	-0.80894	3.67753
C	-1.16076	1.58939	1.35203
H	-1.91525	2.39624	1.27592
H	-0.74385	1.64914	2.37245
C	-1.86835	0.22142	1.15891
H	-1.76180	-0.40023	2.06260
C	-3.35292	0.32633	0.82061
C	-4.28847	-0.45955	1.51960
C	-3.81639	1.18793	-0.19556
C	-5.65792	-0.38233	1.22189
H	-3.93583	-1.14221	2.30083
C	-5.18352	1.26022	-0.50185
H	-3.10400	1.80778	-0.75111
C	-6.10914	0.47775	0.20852
H	-6.37191	-0.99740	1.78002
H	-5.52729	1.93303	-1.29482
H	-7.17694	0.53957	-0.02686
C	-2.08145	-3.31871	-2.02940
H	-2.83432	-3.15597	-2.81943
H	-1.24574	-3.87862	-2.48538
H	-2.52829	-3.94281	-1.24056

TS(E2-E2′)$_{2,1}$

SCF (BP86) Energy = -1305.85053993
Enthalpy 0K = -1305.348736
Energy 298K = -1305.317906
Free Energy 298K = -1305.408182
Lowest Frequency = -7.8716 cm^{-1}
SCF (DCE) Energy = -1305.85950228
SCF (BP86-D3) Energy = -1305.94403276
SCF (BS2) Energy = -1305.80527434

Rh	-0.12026	-0.75966	0.10947
C	-0.89531	-2.06800	1.64339
C	-1.99662	-1.77278	0.74011
C	-1.58482	-2.22766	-0.56173
C	-0.30689	-2.96516	-0.41675
C	0.09364	-2.89779	0.94149
C	2.35667	2.19210	0.08487
C	3.28797	2.94304	0.84477
C	1.19202	2.84332	-0.40231
C	3.08931	4.30307	1.10885
H	4.17420	2.43404	1.23792
C	1.00872	4.21327	-0.12949
C	1.94401	4.94746	0.61347
H	3.82458	4.85675	1.70249
H	0.11017	4.70846	-0.51651
H	1.77741	6.01235	0.80797
C	3.99535	0.33457	-0.50875
C	3.85542	-1.04599	-0.78329
H	4.90378	0.93508	-0.53945
N	1.83053	-0.29088	-0.36030
N	2.56175	-1.41203	-0.67971
C	2.69979	0.79885	-0.24975
C	0.12833	2.06826	-1.14124
C	-0.73423	1.26263	-0.11218

```
H   0.58890  1.40929 -1.89790
H  -0.45101  1.60929  0.90518
C   1.33802 -3.50349  1.52120
H   1.26813 -4.60722  1.53101
H   2.21125 -3.21275  0.91176
H   1.50832 -3.17127  2.55731
C   0.44439 -3.66029 -1.51028
H   0.53678 -4.73943 -1.28899
H  -0.05451 -3.55351 -2.48571
H   1.45951 -3.22450 -1.57178
C  -0.85943 -1.74446  3.10773
H  -1.24195 -2.59933  3.69856
H   0.16779 -1.53745  3.44828
H  -1.48307 -0.86796  3.34155
C  -3.34860 -1.25147  1.12468
H  -3.99620 -2.09358  1.43644
H  -3.29526 -0.54229  1.96464
H  -3.84338 -0.73111  0.29127
C  -2.39838 -2.16415 -1.81836
H  -3.06720 -1.28957 -1.81492
H  -1.75493 -2.10300 -2.71084
H  -3.02170 -3.07400 -1.91821
H  -0.52089  2.77930 -1.68461
C   4.90909 -2.04468 -1.16871
H   5.39085 -1.77790 -2.12665
H   5.71000 -2.10351 -0.41019
H   4.46279 -3.04590 -1.27878
C  -2.20353  1.50002 -0.28406
C  -2.99619  1.96030  0.79582
C  -2.84463  1.35877 -1.54175
C  -4.36151  2.24431  0.63859
C  -4.21000  1.63906 -1.70291
C  -4.98010  2.07950 -0.61163
H  -2.51932  2.11296  1.77209
H  -2.25341  1.03254 -2.40523
H  -4.94230  2.60693  1.49428
H  -4.67454  1.52214 -2.68875
H  -6.04407  2.30443 -0.73897
```

E2′$_{2,1}$

SCF (BP86) Energy = -1305.85055221
Enthalpy 0K = -1305.348584
Energy 298K = -1305.316904
Free Energy 298K = -1305.410932
Lowest Frequency = 7.2199 cm^{-1}
SCF (DCE) Energy = -1305.85950048
SCF (BP86-D3) Energy = -1305.94400892
SCF (BS2) Energy = -1305.80531112

```
Rh -0.11753 -0.77720  0.09658
C  -0.95515 -2.13752  1.55798
C  -2.01534 -1.81336  0.61700
C  -1.54397 -2.22276 -0.68012
C  -0.27105 -2.96218 -0.50189
C   0.06648 -2.94193  0.87531
C   2.33465  2.22462  0.08753
C   3.24514  3.01233  0.83566
C   1.17446  2.84863 -0.44512
C   3.03277  4.38028  1.04183
H   4.12641  2.52622  1.26690
C   0.97756  4.22693 -0.23153
C   1.89398  4.99689  0.49873
H   3.75241  4.96169  1.62806
H   0.08240  4.69937 -0.65317
H   1.71674  6.06735  0.64783
C   4.00780  0.36832 -0.39147
C   3.89505 -1.02131 -0.62831
H   4.90900  0.97999 -0.41275
N   1.84729 -0.28251 -0.29231
N   2.60362 -1.40152 -0.55628
C   2.69880  0.82292 -0.18729
C   0.12685  2.03359 -1.16379
```

```
C  -0.72014  1.24671 -0.10801
H   0.60209  1.36002 -1.89819
H  -0.39699  1.58349  0.90092
C   1.28316 -3.56721  1.49144
H   1.21901 -4.67060  1.45214
H   2.18369 -3.24721  0.93893
H   1.39914 -3.27748  2.54764
C   0.53146 -3.61914 -1.58268
H   0.59665 -4.70857 -1.40611
H   0.08941 -3.46123 -2.57841
H   1.55389 -3.19751 -1.56890
C  -0.98546 -1.86118  3.03208
H  -1.39292 -2.73461  3.57728
H   0.02514 -1.66552  3.42502
H  -1.62044 -0.99281  3.26563
C  -3.38533 -1.30913  0.95818
H  -4.04817 -2.16519  1.18951
H  -3.37555 -0.64670  1.83676
H  -3.83611 -0.74267  0.12967
C  -2.30064 -2.11642 -1.96920
H  -2.97808 -1.24846 -1.96100
H  -1.61813 -2.01446 -2.82832
H  -2.91043 -3.02609 -2.13354
H  -0.53356  2.71491 -1.73077
C   4.97407 -2.01685 -0.94576
H   5.74130 -2.05176 -0.15172
H   4.54260 -3.02482 -1.05355
H   5.49504 -1.76504 -1.88703
C  -2.18983  1.50527 -0.22587
C  -2.94499  1.92102  0.89846
C  -2.87270  1.42129 -1.46686
C  -4.31220  2.21960  0.79774
C  -4.24081  1.71564 -1.57121
C  -4.97212  2.11317 -0.43786
H  -2.43607  2.02762  1.86461
H  -2.31299  1.13069 -2.36345
H  -4.86249  2.54855  1.68657
H  -4.73759  1.64367 -2.54556
H  -6.03789  2.34995 -0.52073
```

TS(E2′-E$_{trans}$)

SCF (BP86) Energy = -1305.84820457
Enthalpy 0K = -1305.346484
Energy 298K = -1305.315556
Free Energy 298K = -1305.406732
Lowest Frequency = -61.0748 cm^{-1}
SCF (DCE) Energy = -1305.85762367
SCF (BP86-D3) Energy = -1305.94040928
SCF (BS2) Energy = -1305.80300525

```
Rh  0.22860 -0.81262  0.15656
C   1.76711 -1.94580 -0.85399
C   2.24337 -1.67533  0.47699
C   1.28903 -2.28758  1.39471
C   0.32753 -3.07972  0.64475
C   0.57351 -2.81814 -0.73829
C  -2.51297  2.02725  0.06077
C  -3.51608  2.91974 -0.40157
C  -1.41465  2.58672  0.77464
C  -3.47135  4.29248 -0.13799
H  -4.34193  2.51945 -0.99648
C  -1.39070  3.96816  1.04564
C  -2.40700  4.82624  0.60448
H  -4.26550  4.94381 -0.51827
H  -0.53890  4.37586  1.60270
H  -2.35677  5.89839  0.82104
C  -3.99766  0.01227 -0.39787
C  -3.77756 -1.37230 -0.53995
H  -4.95523  0.53065 -0.40591
N  -1.80608 -0.46644 -0.16462
N  -2.46287 -1.64595 -0.40250
C  -2.73215  0.58317 -0.17295
```

C -0.22657 1.74249 1.16993
C   0.53080 1.25302 -0.10005
H -0.57246 0.89082 1.80162
H -0.07062 1.48255 -0.99574
C -0.22056 -3.38567 -1.87345
H -0.09072 -4.48312 -1.91794
H -1.28965 -3.16100 -1.70647
H   0.08670 -2.96547 -2.84324
C -0.77305 -3.94037 1.19224
H -0.59813 -5.00612 0.95379
H -0.84901 -3.85354 2.28780
H -1.73571 -3.63057 0.74938
C   2.45877 -1.58409 -2.13317
H   3.21887 -2.34784 -2.38814
H   1.74826 -1.52784 -2.97315
H   2.96823 -0.61022 -2.05211
C   3.54958 -1.03871 0.84828
H   4.33861 -1.81170 0.92323
H   3.87018 -0.29483 0.10422
H   3.49649 -0.52463 1.82090
C   1.37047 -2.22042 2.89190
H   1.87328 -1.30044 3.22865
H   0.37032 -2.25709 3.35230
H   1.94921 -3.07973 3.28166
H   0.44603 2.32824 1.81971
C -4.77046 -2.47047 -0.79519
H -5.31154 -2.31814 -1.74631
H -4.25575 -3.44336 -0.84693
H -5.53120 -2.52712 0.00402
C   1.91267 1.77165 -0.31262
C   2.39384 1.94267 -1.63819
C   2.78801 2.14682 0.73791
C   3.67496 2.44708 -1.89983
C   4.06710 2.66309 0.47932
C   4.52385 2.81306 -0.83999
H   1.72939 1.68913 -2.47318
H   2.46539 2.03295 1.77874
H   4.00615 2.57278 -2.93684
H   4.71182 2.95067 1.31762
H   5.52085 3.21845 -1.04065

E_{trans}
SCF (BP86) Energy = -1305.86338493
Enthalpy 0K = -1305.361868
Energy 298K = -1305.330633
Free Energy 298K = -1305.422128
Lowest Frequency = 29.6591 cm^{-1}
SCF (DCE) Energy = -1305.87277369
SCF (BP86-D3) Energy = -1305.95614231
SCF (BS2) Energy = -1305.81802421

Rh 0.27159 -0.59839 0.38595
C   2.31493 -1.47779 0.09363
C   2.10502 -1.22440 1.49884
C   1.00862 -2.08599 1.96367
C   0.51104 -2.80943 0.84379
C   1.28506 -2.39903 -0.34342
C -2.81488 1.40870 0.19119
C -4.10933 1.98263 0.30447
C -1.74315 2.09674 0.83171
C -4.34038 3.17542 0.99475
H -4.95486 1.45857 -0.14941
C -1.99339 3.28537 1.54565
C -3.27589 3.84137 1.62367
H -5.35696 3.57883 1.05226
H -1.15201 3.78992 2.03630
H -3.44157 4.77379 2.17249
C -3.63553 -0.40785 -1.44288
C -3.06621 -1.60656 -1.89727
H -4.59947 0.01299 -1.72536
N -1.59646 -0.70765 -0.54430
N -1.83353 -1.77298 -1.34471

C -2.67480 0.16051 -0.57369
C -0.31966 1.59306 0.80055
C   0.32587 1.31805 -0.53106
H -0.42706 0.58140 1.48923
H -0.38002 1.30187 -1.37092
C   1.11686 -2.99726 -1.70758
H   1.40768 -4.06491 -1.70627
H   0.05529 -2.91719 -2.00569
H   1.73538 -2.47689 -2.45566
C -0.59056 -3.82523 0.81559
H -0.18124 -4.83561 0.62822
H -1.14558 -3.85431 1.76641
H -1.29418 -3.57883 -0.00025
C   3.43669 -0.94340 -0.74648
H   4.31866 -1.60699 -0.66858
H   3.15366 -0.88387 -1.80868
H   3.74298 0.06621 -0.43056
C   2.98744 -0.39258 2.38919
H   3.78563 -1.01281 2.83993
H   3.47422 0.41921 1.82644
H   2.41947 0.05956 3.21935
C   0.55042 -2.20853 3.39121
H   0.60316 -1.24405 3.92204
H -0.48808 -2.56918 3.45292
H   1.18761 -2.92289 3.94646
H   0.31991 2.19777 1.46287
C -3.62147 -2.63626 -2.84124
H -3.91934 -2.18846 -3.80593
H -2.86216 -3.40866 -3.04393
H -4.51460 -3.13854 -2.42692
C   1.62369 1.92317 -0.90349
C   1.97704 1.97361 -2.27681
C   2.54577 2.47135 0.02413
C   3.18790 2.53484 -2.70143
C   3.75635 3.03936 -0.40062
C   4.08819 3.07293 -1.76547
H   1.27576 1.56604 -3.01438
H   2.31184 2.46639 1.09431
H   3.42678 2.56106 -3.77024
H   4.44182 3.46544 0.34048
H   5.03158 3.51959 -2.09547

$TS(E\text{-}G)_{trans}$
SCF (BP86) Energy = -1305.86043189
Enthalpy 0K = -1305.361757
Energy 298K = -1305.330602
Free Energy 298K = -1305.422115
Lowest Frequency = -572.0674 cm^{-1}
Second Frequency = 31.1550 cm^{-1}
SCF (DCE) Energy = -1305.86950853
SCF (BP86-D3) Energy = -1305.95358935
SCF (BS2) Energy = -1305.81464555

C -4.20762 2.87524 1.82347
C -3.32166 2.30100 2.75113
C -2.08133 1.80424 2.33054
C -1.68401 1.86076 0.97033
C -2.58980 2.44673 0.05038
C -3.83145 2.94599 0.47140
C -0.34606 1.34578 0.60691
C   0.29007 1.59792 -0.68576
C   1.71165 2.07370 -0.77857
C   2.79822 1.42304 -0.12188
C   4.08536 2.00436 -0.25761
C   4.30130 3.16958 -0.99896
C   3.22786 3.79216 -1.65727
C   1.95007 3.23047 -1.54955
C   2.66389 0.19208 0.66496
C   3.59349 -0.35714 1.57852
C   3.01557 -1.55870 2.01803
N   1.80843 -1.74312 1.41333
N   1.59941 -0.68468 0.59987

Rh -0.20315 -0.55389 -0.43075
C -2.28355 -1.51207 -0.17341
C -2.05221 -1.16180 -1.56306
C -0.93085 -1.96080 -2.04754
C -0.42759 -2.72835 -0.94620
C -1.26326 -2.44071 0.22913
C -2.95676 -0.31451 -2.41639
C -0.44973 -2.02118 -3.47257
C   0.69768 -3.72079 -0.95616
C -1.10687 -3.11464 1.55998
C -3.43527 -1.03852 0.66255
H   4.93553 1.50548 0.21654
H   5.31248 3.58294 -1.07654
H   1.10243 3.70735 -2.05676
H   3.38342 4.69858 -2.25083
H   4.54026 0.07812 1.89597
C   3.53883 -2.57543 2.99388
H   0.59727 0.33784 -1.49977
H   0.33572 1.27585 1.46271
H -4.33170 -1.65319 0.45546
H -3.21537 -1.11711 1.73803
H -3.69294 0.01155 0.45128
H -1.78894 -2.68815 2.31234
H -1.32283 -4.19661 1.47972
H -0.06590 -2.98615 1.91049
H -3.75760 -0.92818 -2.87159
H -3.44237 0.47662 -1.82352
H -2.40555 0.16664 -3.24121
H -1.02129 -2.77821 -4.04219
H -0.57687 -1.05595 -3.98820
H   0.61533 -2.29459 -3.52894
H   1.30465 -3.63997 -1.87160
H   1.35176 -3.54718 -0.08382
H   0.30545 -4.75359 -0.89790
H -0.34810 2.09616 -1.42889
H -1.39291 1.36444 3.06150
H -2.31687 2.53115 -1.00680
H -3.59577 2.24555 3.81024
H -4.50545 3.40301 -0.26159
H -5.17493 3.26990 2.15043
H   2.79030 -3.36962 3.14575
H   4.47063 -3.05021 2.63651
H   3.76126 -2.12346 3.97696

G_{trans}

SCF Energy = -1305.86356201
Enthalpy 0K = -1305.363792
Energy 298K = -1305.331971
Free Energy 298K = -1305.425978
Lowest Frequency = 20.4482 cm^{-1}
SCF (DCE) Energy = -1305.87386930
SCF (BP86-D3) Energy = -1305.95877853
SCF (BS2) Energy = -1305.81639036

C   5.33952 0.74275 2.11158
C   5.28664 1.10374 0.75765
C   4.04655 1.23024 0.11163
C   2.83309 0.99616 0.79950
C   2.90507 0.63479 2.16666
C   4.14037 0.51240 2.81192
C   1.55371 1.18770 0.06878
C   0.30022 1.48830 0.65449
C -0.77736 2.29342 0.03173
C -2.12841 1.94698 0.34233
C -3.16879 2.76056 -0.15601
C -2.89179 3.87933 -0.95539
C -1.56409 4.19994 -1.28463
C -0.51515 3.40985 -0.78688
C -2.40578 0.73512 1.11575
C -3.51051 0.38307 1.91639
C -3.24630 -0.93228 2.35063
N -2.05975 -1.36936 1.84015
N -1.55778 -0.35524 1.10381
Rh 0.15044 -0.56961 -0.06159
C -0.28793 -0.73437 -2.40943
C   1.08787 -1.10233 -2.07743
C   1.07233 -2.25942 -1.20838
C -0.31256 -2.56235 -0.92315
C -1.14091 -1.62029 -1.69382
C   2.31220 -0.52756 -2.73264
C   2.27662 -3.05602 -0.78285
C -0.84744 -3.72526 -0.13529
C -2.64131 -1.65154 -1.70259
C -0.69098 0.35783 -3.36077
H -4.20605 2.49037 0.06878
H -3.71544 4.49634 -1.33021
H   0.52531 3.68472 -0.99447
H -1.34375 5.07169 -1.90958
H -4.35885 1.01706 2.17397
C -4.05463 -1.82417 3.25146
H   0.80583 -1.00879 1.27444
H   1.69128 1.50310 -0.97468
H -0.46524 0.08298 -4.40844
H -1.77008 0.56513 -3.29545
H -0.16769 1.30585 -3.14654
H -3.06995 -0.71228 -2.08375
H -3.00301 -2.47760 -2.34325
H -3.03133 -1.82059 -0.68571
H   2.56816 -1.11439 -3.63523
H   2.15159 0.51296 -3.05918
H   3.18674 -0.55036 -2.06291
H   2.54525 -3.80100 -1.55578
H   3.15269 -2.40862 -0.62089
H   2.08706 -3.59849 0.15591
H -0.03552 -4.26283 0.37918
H -1.55444 -3.36914 0.63469
H -1.36542 -4.44490 -0.79649
H   0.21819 1.40263 1.74638
H   4.01247 1.53469 -0.94102
H   1.98352 0.43032 2.72148
H   6.21046 1.29370 0.20076
H   4.17014 0.23028 3.86975
H   6.30369 0.64191 2.62037
H -3.56790 -2.80895 3.33431
H -4.14584 -1.40429 4.26960
H -5.08024 -1.97645 2.87012

$D1_{2,1}$

SCF Energy = -1305.86340867
Enthalpy 0K = -1305.362200
Energy 298K = -1305.330187
Free Energy 298K = -1305.423719
Lowest Frequency = 20.8536 cm^{-1}
SCF (BS2) Energy = -1305.81747704
SCF (BP86-D3) Energy = -1305.96491760
SCF (DCE) Energy = -1305.87180674

Rh -0.24637 -0.49882 -0.00200
C -0.13637 -2.57552 0.91052
C   1.15052 -1.92924 1.20061
C   0.90165 -0.77338 1.99529
C -0.54800 -0.69966 2.22210
C -1.16765 -1.85011 1.61321
C -2.58499 1.08694 -0.93362
C -3.84927 1.35749 -1.49999
C -2.06719 -0.24379 -0.94688
C -4.60139 0.32562 -2.08062
H -4.23867 2.38189 -1.48256
C -2.82809 -1.25973 -1.54714
C -4.09349 -0.98293 -2.10677
H -5.58096 0.54465 -2.51889
H -2.43808 -2.28446 -1.59359
H -4.67175 -1.79114 -2.56888
C -1.70956 3.43950 0.00201

C -0.48058 3.64921 0.66809
H -2.47960 4.17620 -0.22473
N -0.54207 1.54171 0.17203
N   0.22344 2.48034 0.76512
C -1.71557 2.06570 -0.30219
C   0.39789 -1.16046 -1.98016
C   1.03251 0.08066 -1.72604
H   0.52250 0.98672 -2.07485
H   0.97544 -2.08943 -1.89088
H -0.46908 -1.21323 -2.64333
C   2.49049 0.24437 -1.46940
C   2.97416 1.38439 -0.78220
C   3.42913 -0.68679 -1.97312
C   4.34991 1.56597 -0.58644
H   2.25101 2.09707 -0.36694
C   4.80651 -0.49838 -1.77972
H   3.07686 -1.54872 -2.55024
C   5.27279 0.62705 -1.08173
H   4.70605 2.44928 -0.04495
H   5.51654 -1.22611 -2.18776
H   6.34758 0.77785 -0.93445
C -2.60821 -2.25363 1.73838
H -2.72891 -2.98579 2.55882
H -3.25140 -1.38850 1.95993
H -2.98641 -2.71525 0.81300
C -0.30933 -3.90811 0.23508
H -0.18635 -4.73403 0.96131
H -1.31193 -4.00807 -0.21092
H   0.43388 -4.06310 -0.56352
C   2.49125 -2.47725 0.80806
H   2.80484 -3.26174 1.52261
H   2.47138 -2.93982 -0.19241
H   3.26631 -1.69712 0.79430
C   1.89850 0.19645 2.56069
H   2.90572 0.02172 2.15306
H   1.60325 1.23116 2.30728
H   1.95306 0.10516 3.66160
C -1.21685 0.33461 3.08002
H -1.09670 0.08059 4.15005
H -0.76784 1.32631 2.90955
H -2.29445 0.40526 2.86680
C   0.09378 4.92158 1.22722
H -0.58685 5.39391 1.95804
H   1.04818 4.70983 1.73531
H   0.28817 5.66805 0.43572

TS(D1-E1)$_{2,1}$
SCF Energy = -1305.83792665
Enthalpy 0K = -1305.337104
Energy 298K = -1305.305864
Free Energy 298K = -1305.397305
Lowest Frequency = -264.5389 cm^{-1}
SCF (DCE) Energy = -1305.84647208
SCF (BP86-D3) Energy = -1305.93713733
SCF (BS2) Energy = -1305.79216501

Rh -0.02200 -0.03850 0.26700
C   1.08020 -1.27641 1.83000
C   1.55880 0.08750 1.90830
C   0.42970 0.94250 2.18360
C -0.74560 0.07550 2.38210
C -0.33700 -1.27950 2.18710
C -2.47010 -0.80800 -1.25160
C -3.54410 -1.66990 -1.54900
C -1.15220 -1.35620 -1.04500
C -3.35080 -3.05810 -1.62170
H -4.54310 -1.24470 -1.69680
C -0.98850 -2.75940 -1.11470
C -2.07470 -3.60500 -1.39200
H -4.19610 -3.71450 -1.85320
H   0.01320 -3.18950 -0.99090
H -1.92010 -4.68810 -1.44820

C -3.52100 1.64800 -1.30680
C -2.91850 2.81280 -0.78440
H -4.49030 1.56010 -1.79680
N -1.49800 1.21080 -0.44640
N -1.68589 2.52730 -0.25710
C -2.57880 0.62700 -1.06790
C   0.23040 -0.48980 -1.99810
C   1.07430 0.59149 -1.46120
C -1.18290 -2.50830 2.35050
H -0.87650 -3.07300 3.25050
H -2.24790 -2.25280 2.45980
H -1.09280 -3.18230 1.48160
C -2.10520 0.58020 2.77270
H -2.89230 -0.15990 2.56110
H -2.13740 0.81200 3.85390
H -2.34880 1.50320 2.22120
C   1.93480 -2.50351 1.66680
H   2.31710 -2.85221 2.64570
H   1.36330 -3.33561 1.22460
H   2.80690 -2.30521 1.02350
C   2.99250 0.51789 1.82110
H   3.46350 0.44599 2.81980
H   3.57170 -0.11251 1.12870
H   3.08880 1.55919 1.47830
C   0.42971 2.43150 2.38390
H   1.39800 2.87229 2.09940
H -0.35319 2.90120 1.76040
H   0.23911 2.68690 3.44330
H   0.76920 -1.37850 -2.34500
H -0.49230 -0.12880 -2.73970
H   0.72000 1.60830 -1.67440
C   2.55040 0.48379 -1.44100
C   3.32240 1.67259 -1.43860
C   3.25030 -0.74941 -1.47310
C   4.72340 1.63419 -1.46710
H   2.80240 2.63779 -1.42570
C   4.65140 -0.78851 -1.50540
H   2.68990 -1.69080 -1.47610
C   5.39860 0.40279 -1.50130
H   5.29061 2.57139 -1.47020
H   5.16430 -1.75621 -1.53980
H   6.49240 0.37009 -1.53050
C -3.43039 4.22660 -0.77460
H -3.44279 4.66890 -1.78770
H -4.46059 4.28540 -0.38160
H -2.78239 4.85420 -0.14220

E1$_{2,1}$
SCF Energy = -1305.85389314
Enthalpy 0K = -1305.351724
Energy 298K = -1305.320076
Free Energy 298K = -1305.413445
Lowest Frequency = 16.4104 cm^{-1}
SCF (DCE) Energy = -1305.86354073
SCF (BP86-D3) Energy = -1305.95110789
SCF (BS2) Energy = -1305.80800876

Rh 0.42346 -0.70460 -0.03423
C -0.39912 -2.63352 0.73035
C   0.11726 -2.79192 -0.59273
C   1.55335 -2.44256 -0.54163
C   1.93283 -2.24147 0.85555
C   0.72783 -2.25693 1.60954
C   0.18794 2.92808 0.11928
C   0.08000 3.93951 1.10283
C -0.92487 2.65963 -0.72323
C -1.10114 4.67633 1.26017
H   0.94368 4.13676 1.74705
C -2.10185 3.41479 -0.55329
C -2.19993 4.41493 0.42557
H -1.16220 5.45329 2.02989
H -2.95415 3.21306 -1.21257

H -3.12720 4.98771 0.53343
C   2.75161 2.86204 -0.09645
C   3.65953 1.80866 -0.35314
H   2.96434 3.92548 0.00914
N   1.66544 0.89952 -0.28097
N   2.99701 0.63231 -0.45038
C   1.48565 2.25900 -0.07608
C -0.83948 1.59914 -1.79681
C -1.04237 0.13066 -1.32861
H -0.96095 -0.49270 -2.23962
H   0.14463 1.66003 -2.29537
C   0.58656 -1.99493 3.08041
H   0.46864 -2.94613 3.63355
H   1.46472 -1.47079 3.48679
H -0.30556 -1.38274 3.29217
C   3.32789 -1.97911 1.33582
H   3.35196 -1.76374 2.41569
H   3.97494 -2.85685 1.15286
H   3.74552 -1.11162 0.79241
C -1.78513 -2.95596 1.20770
H -1.83288 -3.99972 1.57383
H -2.09126 -2.29772 2.03574
H -2.53036 -2.83874 0.40626
C -0.61555 -3.31699 -1.79116
H -0.52272 -4.41798 -1.84760
H -1.68827 -3.07158 -1.74379
H -0.21015 -2.90260 -2.72860
C   2.52706 -2.45347 -1.67894
H   2.01805 -2.47287 -2.65491
H   3.15563 -1.54621 -1.61963
H   3.18504 -3.34144 -1.61554
H -1.60824 1.81784 -2.56592
C -2.38764 -0.12695 -0.69907
C -3.37202 -0.87757 -1.39021
C -2.73801 0.37644 0.58169
C -4.63513 -1.13065 -0.82940
H -3.14400 -1.24771 -2.39735
C -3.99904 0.12718 1.14247
H -2.00695 0.97210 1.13809
C -4.95464 -0.63120 0.44333
H -5.37366 -1.71107 -1.39391
H -4.23924 0.53219 2.13191
H -5.93910 -0.82305 0.88285
C   5.15020 1.86266 -0.53379
H   5.43580 2.51224 -1.38066
H   5.65647 2.26104 0.36394
H   5.54378 0.85271 -0.73132

TS(E1-F1)$_{2,1}$
SCF Energy = -1305.77528188
Enthalpy 0K = -1305.275451
Energy 298K = -1305.243880
Free Energy 298K = -1305.335994
Lowest Frequency = -395.4841 cm^{-1}
SCF (DCE) Energy = -1305.78220042
SCF (BP86-D3) Energy = -1305.87570946
SCF (BS2) Energy = -1305.72613989

Rh -0.40188 -0.29836 0.10942
C   0.09040 -2.27669 1.04437
C   0.55645 -1.19653 1.88042
C -0.62637 -0.52554 2.48035
C -1.78324 -1.11836 1.94928
C -1.34720 -2.12733 0.95902
C -1.74173 0.99265 -1.22059
C -3.16958 0.87761 -1.13700
C -0.98864 0.03244 -2.03539
C -3.84506 -0.14440 -1.78442
H -3.71381 1.62707 -0.55206
C -1.74605 -1.00381 -2.68310
C -3.12291 -1.10222 -2.55832
H -4.93583 -0.21155 -1.71419

H -1.20524 -1.70633 -3.32959
H -3.66319 -1.90050 -3.07787
C -1.18865 3.49016 -0.31343
C   0.00161 3.81293 0.41689
H -2.03734 4.14080 -0.52114
N   0.19207 1.73924 -0.13798
N   0.83525 2.74711 0.51537
C -1.01303 2.15429 -0.66496
C   1.97096 -0.96386 2.33227
H   2.69884 -1.34829 1.60108
H   2.17510 0.10997 2.47183
H   2.16315 -1.46931 3.29955
C -0.52763 0.60929 3.45946
H -0.05777 0.28328 4.40644
H   0.09060 1.42846 3.05063
H -1.51900 1.02443 3.70165
C -3.21887 -0.78149 2.22549
H -3.74373 -1.62344 2.71597
H -3.31140 0.09770 2.88330
H -3.76602 -0.56202 1.28937
C -2.29335 -3.05621 0.25232
H -2.75550 -3.76238 0.96891
H -3.10963 -2.50379 -0.24395
H -1.77839 -3.64922 -0.51984
C   0.92026 -3.38811 0.46708
H   1.08376 -4.19209 1.21174
H   0.42959 -3.84647 -0.40761
H   1.91210 -3.02943 0.14622
C   0.37471 0.39965 -2.61129
H   0.26015 1.16078 -3.41193
H   0.79449 -0.50004 -3.09442
C   1.35221 0.91109 -1.55341
H   1.62958 1.96126 -1.70809
C   2.54550 0.15197 -1.12577
C   3.54165 0.85050 -0.39053
C   2.82400 -1.18341 -1.51849
C   4.75582 0.23743 -0.05597
H   3.32722 1.87646 -0.07241
C   4.04997 -1.78133 -1.20537
H   2.07715 -1.75470 -2.07825
C   5.02302 -1.07926 -0.46644
H   5.50396 0.79627 0.51669
H   4.25186 -2.80405 -1.54247
H   5.97826 -1.55463 -0.22126
C   0.38264 5.12763 1.03552
H   0.39962 5.93987 0.28698
H -0.33056 5.42835 1.82417
H   1.38383 5.05081 1.48714

F1$_{2,1}$
SCF Energy = -1305.84036383
Enthalpy 0K = -1305.338299
Energy 298K = -1305.306675
Free Energy 298K = -1305.400125
Lowest Frequency = 18.2506 cm^{-1}
SCF (DCE) Energy = -1305.84856233
SCF (BP86-D3) Energy = -1305.93906570
SCF (BS2) Energy = -1305.79228817

Rh 0.79067 -0.12235 -0.14454
C   0.88214 -1.60619 -1.84160
C   0.56538 -0.28184 -2.33496
C   1.72060 0.60092 -2.12297
C   2.69652 -0.13975 -1.39688
C   2.16030 -1.49170 -1.16927
C   1.02671 0.74262 1.83557
C   2.32535 1.10796 2.34172
C   0.52194 -0.63672 1.98418
C   3.14970 0.14419 2.88736
H   2.64325 2.15461 2.28412
C   1.45302 -1.58725 2.55136
C   2.71963 -1.22348 2.96620

H   4.13623 0.42329 3.27235
H   1.10834 -2.62005 2.68956
H   3.39150 -1.97450 3.39524
C   0.08310 2.97457 0.70129
C -1.09682 3.07317 -0.07880
H   0.82312 3.74995 0.89065
N -1.05824 0.99534 0.60469
N -1.80732 1.92705 -0.12634
C   0.12388 1.63748 1.13846
C -0.68256 0.13567 -3.06235
H -1.42825 -0.67385 -3.08740
H -1.15638 1.00298 -2.56731
H -0.45432 0.42084 -4.10655
C   1.79148 2.03906 -2.55554
H   1.48156 2.16011 -3.60902
H   1.13358 2.68106 -1.93762
H   2.81639 2.43346 -2.46226
C   4.05045 0.32072 -0.93654
H   4.85898 -0.23115 -1.45176
H   4.19869 1.39487 -1.13218
H   4.17976 0.16100 0.14951
C   2.94166 -2.61400 -0.54626
H   3.72194 -2.98250 -1.23965
H   3.44205 -2.28754 0.38107
H   2.28994 -3.46518 -0.29228
C   0.11410 -2.87620 -2.08608
H   0.50141 -3.41606 -2.97275
H   0.17957 -3.56686 -1.22861
H -0.95384 -2.67672 -2.27204
C -0.96897 -0.89591 2.21057
H -1.19478 -0.91102 3.29627
H -1.21077 -1.90300 1.82999
C -1.89159 0.15581 1.54359
H -2.26631 0.86220 2.30885
C -3.09322 -0.44281 0.83044
C -4.39139 -0.00224 1.14384
C -2.93372 -1.46358 -0.12786
C -5.51237 -0.57048 0.51825
H -4.52307 0.79711 1.88211
C -4.05062 -2.02656 -0.76177
H -1.92240 -1.80108 -0.38072
C -5.34459 -1.58399 -0.43794
H -6.51683 -0.21870 0.77666
H -3.91343 -2.81795 -1.50702
H -6.21720 -2.02924 -0.92763
C -1.60388 4.27836 -0.82273
H -1.03970 4.44594 -1.75756
H -2.66400 4.13949 -1.08503
H -1.50352 5.19061 -0.21100

TS(E1-E_{cis})
SCF Energy = -1305.84384657
Enthalpy 0K = -1305.342657
Energy 298K = -1305.311447
Free Energy 298K = -1305.404020
Lowest Frequency = -51.3675 cm^{-1}
SCF (DCE) Energy = -1305.85451365
SCF (BP86-D3) Energy = -1305.93676901
SCF (BS2) Energy = -1305.79789335

Rh 1.05894 -0.13843 -0.35889
C   2.48450 -0.68405 1.10971
C   2.30869 -1.80742 0.18041
C   2.74442 -1.33494 -1.10321
C   3.38188 -0.00715 -0.91931
C   3.29081 0.35736 0.43439
C -2.46459 1.48679 -0.64735
C -3.72458 2.08024 -0.36558
C -2.44770 0.35781 -1.51403
C -4.91361 1.61441 -0.93202
H -3.76230 2.91854 0.33565
C -3.65655 -0.09516 -2.08133

C -4.88488 0.51713 -1.80661
H -5.86159 2.10342 -0.68356
H -3.62324 -0.96079 -2.75369
H -5.80566 0.13601 -2.26000
C -1.22300 3.43705 0.44637
C   0.07048 3.59558 0.97975
H -2.00732 4.19001 0.38428
N -0.02662 1.54543 0.19046
N   0.77541 2.45211 0.82650
C -1.27684 2.11670 -0.04031
C -1.19187 -0.40555 -1.85494
C -0.60103 -1.32058 -0.73838
H -0.17119 -2.20923 -1.23849
H -0.39988 0.31624 -2.19228
C   3.79963 1.59434 1.10346
H   4.46573 2.16944 0.44106
H   2.93358 2.23078 1.37042
H   4.36139 1.34352 2.02099
C   4.01652 0.77398 -2.03309
H   4.94134 0.27933 -2.38518
H   3.34145 0.85201 -2.90267
H   4.27322 1.79588 -1.71624
C   2.17720 -0.69092 2.57774
H   3.06334 -1.01422 3.15865
H   1.89845 0.31959 2.91632
H   1.34743 -1.37580 2.81125
C   1.84061 -3.18745 0.54292
H   2.66888 -3.76524 0.99431
H   1.00926 -3.16167 1.26540
H   1.49358 -3.74574 -0.34120
C   2.78027 -2.11843 -2.38615
H   2.01481 -2.91059 -2.40068
H   2.60983 -1.46855 -3.26055
H   3.76448 -2.60584 -2.52522
H -1.38157 -1.02809 -2.75113
C -1.48079 -1.82067 0.37263
C -1.87879 -3.18052 0.36449
C -1.91686 -1.02042 1.45626
C -2.68885 -3.71705 1.37713
H -1.55052 -3.82421 -0.46136
C -2.72771 -1.55393 2.46821
H -1.59138 0.02121 1.51134
C -3.12073 -2.90273 2.43539
H -2.98069 -4.77250 1.33816
H -3.04999 -0.91003 3.29408
H -3.75110 -3.31553 3.23024
C   0.68662 4.79747 1.63848
H   0.68233 5.67658 0.96950
H   0.14256 5.08583 2.55594
H   1.73155 4.58456 1.91567

E_{cis}
SCF (BP86) Energy = -1305.85203094
Enthalpy 0K = -1305.351706
Energy 298K = -1305.320175
Free Energy 298K = -1305.413139
Lowest Frequency = 23.4383 cm^{-1}
SCF (DCE) Energy = -1305.86243732
SCF (BP86-D3) Energy = -1305.94723058
SCF (BS2) Energy = -1305.80756394

C -3.39884 2.15428 2.77871
C -2.14281 2.60956 2.34620
C -1.18160 1.69935 1.88182
C -1.45334 0.31563 1.84235
C -2.72146 -0.12949 2.27997
C -3.68552 0.78064 2.74050
C -0.38877 -0.68246 1.47314
C -0.64173 -1.81024 0.50006
C -1.90573 -1.88641 -0.32041
C -2.31814 -0.85195 -1.21220
C -3.51934 -1.07104 -1.93909

C -4.28158 -2.23252 -1.79503
C -3.85865 -3.24827 -0.92168
C -2.66851 -3.06723 -0.20814
C -1.59639 0.40777 -1.43506
C -2.07541 1.54905 -2.12078
C -1.02111 2.47284 -2.09237
N   0.04674 1.93864 -1.43744
N -0.30138 0.69222 -1.04324
Rh 1.10327 -0.38182 0.04182
C   3.01010 0.36658 -0.98185
C   2.75605 1.02732 0.31362
C   2.84843 0.01872 1.35136
C   3.03756 -1.25764 0.70173
C   3.18005 -1.02382 -0.74246
C   2.61787 2.51104 0.48402
C   2.81482 0.26288 2.83469
C   3.24643 -2.58051 1.38850
C   3.49903 -2.08365 -1.76151
C   3.06460 1.09474 -2.29096
H -3.84616 -0.30758 -2.65004
H -5.20115 -2.35142 -2.37794
H -2.31815 -3.85395 0.47175
H -4.43943 -4.16868 -0.80518
H -3.06720 1.69586 -2.54572
C -0.94600 3.86159 -2.66245
H   0.13504 -1.03783 2.37774
H   0.19996 -1.80442 -0.41400
H   3.11360 0.40010 -3.14417
H   2.16072 1.72299 -2.39486
H   3.95259 1.75266 -2.33495
H   4.58745 -2.28045 -1.79486
H   3.00237 -3.03959 -1.52782
H   3.18338 -1.78069 -2.77184
H   3.56332 3.02616 0.22862
H   1.82207 2.87855 -0.18996
H   2.35751 2.77872 1.52051
H   3.83854 0.41414 3.22558
H   2.22431 1.15798 3.08419
H   2.37806 -0.58874 3.38185
H   2.73538 -2.61788 2.36429
H   2.87133 -3.41894 0.77848
H   4.32159 -2.76618 1.57314
H -0.39496 -2.79427 0.93368
H -0.21661 2.05507 1.50870
H -2.95455 -1.19944 2.25765
H -1.91254 3.68057 2.35928
H -4.66322 0.41289 3.07096
H -4.15094 2.86521 3.13735
H -0.00264 4.34292 -2.35767
H -0.98336 3.85879 -3.76723
H -1.78133 4.49270 -2.31098

TS(E-G)$_{cis}$
SCF (BP86) Energy = -1305.85048989
Enthalpy 0K = -1305.352349
Energy 298K = -1305.321125
Free Energy 298K = -1305.413243
Lowest Frequency = -535.4349 cm^{-1}
SCF (DCE) Energy = -1305.86068884
SCF (BP86-D3) Energy = -1305.94618823
SCF (BS2) Energy = -1305.80577997

Rh -1.08165 -0.38234 -0.04951
C -2.75305 1.11288 -0.13221
C -2.86614 0.25919 -1.28636
C -3.05384 -1.10304 -0.82083
C -3.14772 -1.07353 0.63671
C -2.94035 0.27612 1.06379
C   2.28493 -1.07164 1.04926
C   3.46671 -1.45579 1.73605
C   1.79716 -1.96102 0.04444
C   4.14136 -2.64633 1.45425

H   3.84508 -0.80250 2.52726
C   2.46951 -3.17662 -0.20417
C   3.64197 -3.52352 0.47682
H   5.04967 -2.89881 2.01167
H   2.06375 -3.85313 -0.96667
H   4.15123 -4.46671 0.25520
C   2.18572 1.24112 2.20236
C   1.16656 2.20090 2.29572
H   3.19444 1.30718 2.60768
N   0.35456 0.56324 1.09933
N   0.06058 1.77236 1.62389
C   1.64262 0.19251 1.42326
C   0.56662 -1.69238 -0.77718
C   0.37860 -0.47967 -1.59055
H -0.21382 -0.65892 -2.50223
H -0.33066 -1.74507 0.40085
C -2.95715 0.81233 2.46491
H -2.95587 0.00290 3.21167
H -2.06492 1.44359 2.62649
H -3.85822 1.43099 2.63500
C -3.47125 -2.25229 1.51509
H -4.56634 -2.38392 1.60279
H -3.05907 -3.19016 1.10932
H -3.06806 -2.12153 2.53120
C -2.59429 2.60458 -0.09643
H -3.51010 3.08932 0.29113
H -1.75024 2.86343 0.56989
H -2.39579 3.01686 -1.09880
C -2.85725 0.69820 -2.72490
H -3.88932 0.87255 -3.08280
H -2.29231 1.63334 -2.86104
H -2.40907 -0.06275 -3.38517
C -3.30955 -2.30532 -1.68983
H -2.79201 -2.22467 -2.65982
H -2.97143 -3.23566 -1.20462
H -4.38915 -2.42040 -1.90368
H   0.18539 -2.61094 -1.24823
C   1.43543 0.56534 -1.80644
C   1.12638 1.94096 -1.74923
C   2.73882 0.18630 -2.19878
C   2.08786 2.90853 -2.07366
H   0.13052 2.24254 -1.41035
C   3.70241 1.15455 -2.52163
H   2.99726 -0.87635 -2.25208
C   3.38033 2.51931 -2.46265
H   1.82951 3.97134 -2.01238
H   4.70785 0.83874 -2.82090
H   4.13252 3.27508 -2.71292
C   1.16005 3.52965 2.99837
H   1.26794 3.42016 4.09290
H   1.98484 4.17856 2.65392
H   0.21022 4.05305 2.80269

G$_{cis}$
SCF (BP86) Energy = -1305.86094636
Enthalpy 0K = -1305.361361
Energy 298K = -1305.329741
Free Energy 298K = -1305.422967
Lowest Frequency = 23.2776 cm^{-1}
SCF (DCE) Energy = -1305.87140130
SCF (BP86-D3) Energy = -1305.95795855
SCF (BS2) Energy = -1305.81512713

C -3.30295 -2.78400 -2.23211
C -1.98460 -3.13023 -1.89078
C -1.02980 -2.13032 -1.66287
C -1.37296 -0.76466 -1.76368
C -2.70184 -0.42883 -2.10609
C -3.65710 -1.43028 -2.33869
C -0.33611 0.30886 -1.65206
C -0.49037 1.57679 -1.00503
C -1.66718 2.00870 -0.19621

C -2.26031 1.24226 0.85674
C -3.41124 1.77240 1.49215
C -3.96527 3.00336 1.12758
C -3.36211 3.76792 0.11562
C -2.22115 3.26758 -0.52296
C -1.72850 -0.03503 1.34252
C -2.35314 -1.01041 2.15299
C -1.37077 -1.99027 2.37875
N -0.20969 -1.64000 1.75548
N -0.44421 -0.47175 1.12563
Rh 1.04681 0.42479 0.04577
C   2.89855 -0.11482 1.12640
C   2.75723 -1.17747 0.13174
C   2.81727 -0.58165 -1.16424
C   3.00298 0.86079 -0.99981
C   3.11947 1.13585 0.41218
C   2.57531 -2.62555 0.47430
C   2.80538 -1.29294 -2.48842
C   3.22941 1.83861 -2.12105
C   3.51355 2.45255 1.02784
C   2.97410 -0.35171 2.60927
H -3.85848 1.20534 2.31406
H -4.85413 3.37487 1.64852
H -1.74521 3.85417 -1.31853
H -3.77026 4.74302 -0.16927
H -3.38663 -1.01663 2.49720
C -1.45657 -3.27210 3.15897
H   0.37042 0.30839 -2.49554
H   0.65609 1.58789 1.00371
H   2.87836 0.58871 3.17427
H   2.15850 -1.02398 2.92357
H   3.93773 -0.81831 2.88882
H   4.61554 2.54358 1.06102
H   3.12447 3.30658 0.45158
H   3.13813 2.54527 2.05838
H   3.42329 -2.99340 1.08008
H   1.64534 -2.73891 1.06729
H   2.50569 -3.25462 -0.42728
H   3.83498 -1.39384 -2.88097
H   2.37729 -2.30366 -2.40811
H   2.22627 -0.74305 -3.24984
H   2.57359 1.63055 -2.98361
H   3.04023 2.87458 -1.79780
H   4.27226 1.79050 -2.48793
H   0.02984 2.42140 -1.47651
H -0.01076 -2.39995 -1.36824
H -2.98231 0.62518 -2.19583
H -1.70064 -4.18386 -1.79534
H -4.68185 -1.14884 -2.60436
H -4.04936 -3.56534 -2.41067
H -0.46442 -3.74918 3.20785
H -1.80503 -3.10098 4.19305
H -2.15585 -3.99120 2.69476

TS(E2-E1)$_{2,1}$
SCF (BP86) Energy = -1305.84094994
Enthalpy 0K = -1305.339576
Energy 298K = -1305.308031
Free Energy 298K = -1305.403205
Lowest Frequency = -21.0449 cm^{-1}
SCF (DCE) Energy = -1305.85195087
SCF (BP86-D3) Energy = -1305.93287894
SCF (BS2) Energy = -1305.79498347

Rh 0.98473 -0.06765 -0.25019
C   2.21925 -0.91000 -1.75479
C   2.66709 0.46840 -1.54807
C   3.10580 0.55898 -0.18611
C   3.10625 -0.80369 0.39982
C   2.60365 -1.70048 -0.56322
C -2.93441 -0.77628 -0.47153
C -4.22070 -1.38586 -0.41522
C -2.78093 0.34961 -1.33925
C -5.33168 -0.89745 -1.10389
H -4.33847 -2.29005 0.18662
C -3.93070 0.83690 -2.00638
C -5.19450 0.24924 -1.89776
H -6.29509 -1.41100 -1.01730
H -3.81120 1.71509 -2.65229
H -6.04911 0.67150 -2.43643
C -2.24250 -2.18522 1.55242
C -1.01171 -2.53561 2.14002
H -3.23256 -2.47489 1.89991
N -0.55250 -1.22795 0.43379
N   0.00176 -1.97002 1.44947
C -1.93011 -1.35807 0.45014
C -1.48424 1.02344 -1.77936
C -0.31172 1.43935 -0.87232
H -1.79796 1.94097 -2.31879
H   0.33066 2.05825 -1.52502
C   2.39633 -3.17384 -0.36838
H   3.34714 -3.67230 -0.10885
H   1.67658 -3.33710 0.45520
H   1.99965 -3.65348 -1.27650
C   3.52826 -1.15961 1.79533
H   4.56532 -1.54626 1.81172
H   3.49090 -0.28554 2.46474
H   2.86225 -1.93257 2.21034
C   1.72888 -1.48818 -3.05002
H   2.57739 -1.86813 -3.65218
H   1.03681 -2.32706 -2.87685
H   1.20126 -0.73292 -3.65386
C   2.69966 1.55046 -2.58901
H   3.63378 1.49561 -3.17864
H   1.85779 1.46322 -3.29573
H   2.65336 2.55479 -2.13753
C   3.66267 1.77188 0.50233
H   3.37601 2.70063 -0.01382
H   3.30500 1.84453 1.54235
H   4.76826 1.72748 0.53189
H -1.04216 0.37655 -2.56492
C -0.53546 2.21504 0.39242
C   0.26146 3.36917 0.63345
C -1.48656 1.86986 1.39009
C   0.12413 4.13409 1.79719
H   0.98861 3.67192 -0.13036
C -1.62714 2.63915 2.55359
H -2.11492 0.98763 1.25525
C -0.82426 3.77118 2.76965
H   0.74892 5.02265 1.94176
H -2.37316 2.34687 3.30067
H -0.93871 4.36679 3.68142
C -0.73902 -3.39335 3.34319
H -1.16802 -2.95621 4.26272
H -1.17048 -4.40417 3.23193
H   0.34768 -3.49744 3.49217

E1$_{2,1}$'
SCF (BP86) Energy = -1305.85389278
Enthalpy 0K = -1305.351731
Energy 298K = -1305.320078
Free Energy 298K = -1305.413472
Lowest Frequency = 16.3955 cm^{-1}
SCF (DCE) Energy = -1305.86353829
SCF (BP86-D3) Energy = -1305.95110767
SCF (BS2) Energy = -1305.80801189

Rh -0.42337 -0.70484 -0.03431
C   0.40059 -2.63295 0.73055
C -0.72622 -2.25647 1.60993
C -1.93160 -2.24220 0.85640
C -1.55254 -2.44371 -0.54075
C -0.11620 -2.79213 -0.59227
C -0.18954 2.92794 0.11918

C -0.08234 3.93944 1.10273
C 0.92363 2.65996 -0.72301
C 1.09842 4.67682 1.26037
H -0.94628 4.13631 1.74671
C 2.10021 3.41567 -0.55276
C 2.19755 4.41590 0.42609
H 1.15889 5.45383 2.03010
H 2.95278 3.21432 -1.21180
H 3.12453 4.98912 0.53418
C -2.75315 2.86064 -0.09695
C -3.66054 1.80682 -0.35375
H -2.96644 3.92396 0.00873
N -1.66599 0.89868 -0.28145
N -2.99741 0.63080 -0.45092
C -1.48689 2.25824 -0.07648
C 0.83903 1.59943 -1.79661
C 1.04239 0.13100 -1.32840
H 1.60789 1.81849 -2.56550
C 0.61643 -3.31701 -1.79090
H 0.52421 -4.41806 -1.84712
H 0.21039 -2.90299 -2.72823
H 1.68902 -3.07095 -1.74406
C -2.52667 -2.45552 -1.67768
H -2.01807 -2.47561 -2.65385
H -3.18458 -3.34346 -1.61336
H -3.15525 -1.54822 -1.61878
C 1.78692 -2.95432 1.20766
H 1.83459 -3.99682 1.57739
H 2.53138 -2.84019 0.40507
H 2.09426 -2.29328 2.03304
C -0.58454 -1.99386 3.08066
H -0.46710 -2.94492 3.63415
H 0.30798 -1.38212 3.29198
H -1.46232 -1.46904 3.48699
C -3.32655 -1.98003 1.33713
H -3.35012 -1.76357 2.41680
H -3.74488 -1.11326 0.79312
H -3.97333 -2.85824 1.15546
H -0.14497 1.65992 -2.29543
C -5.15117 1.86013 -0.53494
H -5.65752 2.26263 0.36088
H -5.43659 2.50587 -1.38482
H -5.54479 0.84934 -0.72805
C 2.38795 -0.12610 -0.69924
C 3.37246 -0.87626 -1.39068
C 2.73848 0.37747 0.58139
C 4.63587 -1.12873 -0.83028
H 3.14430 -1.24652 -2.39775
C 3.99983 0.12888 1.14175
H 2.00727 0.97272 1.13803
C 4.95556 -0.62907 0.44231
H 5.37449 -1.70883 -1.39501
H 4.24018 0.53406 2.13109
H 5.94026 -0.82043 0.88151
H 0.96103 -0.49234 -2.23945

TS(E1-E$_{cis}$)$_{2,1}$'
SCF (BP86) Energy = -1305.84384663
Enthalpy 0K = -1305.342657
Energy 298K = -1305.311447
Free Energy 298K = -1305.404020
Lowest Frequency = -51.3825 cm^{-1}
SCF (DCE) Energy = -1305.85451372
SCF (BP86-D3) Energy = -1305.93676837
SCF (BS2) Energy = -1305.79789373

Rh -1.05896 -0.13845 -0.35890
C -2.30881 -1.80740 0.18043
C -2.48459 -0.68401 1.10970
C -3.29077 0.35744 0.43430
C -3.38178 -0.00707 -0.91940
C -2.74447 -1.33494 -1.10322

C 2.46455 1.48671 -0.64745
C 3.72454 2.08020 -0.36576
C 2.44766 0.35769 -1.51408
C 4.91356 1.61439 -0.93222
H 3.76226 2.91852 0.33546
C 3.65650 -0.09527 -2.08141
C 4.88482 0.51706 -1.80676
H 5.86154 2.10343 -0.68382
H 3.62319 -0.96093 -2.75373
H 5.80560 0.13595 -2.26017
C 1.22299 3.43702 0.44621
C -0.07045 3.59556 0.97967
H 2.00729 4.18999 0.38398
N 0.02663 1.54537 0.19050
N -0.77538 2.45208 0.82652
C 1.27683 2.11663 -0.04036
C 1.19183 -0.40571 -1.85492
C 0.60100 -1.32065 -0.73828
H 1.38153 -1.02832 -2.75106
C -2.78031 -2.11847 -2.38613
H -3.76454 -2.60582 -2.52523
H -2.60979 -1.46863 -3.26056
H -2.01490 -2.91068 -2.40061
C -4.01631 0.77413 -2.03319
H -3.34166 0.85108 -2.90320
H -4.94181 0.28022 -2.38451
H -4.27182 1.79644 -1.71667
C -1.84076 -3.18744 0.54296
H -2.66906 -3.76519 0.99435
H -1.49376 -3.74576 -0.34116
H -1.00941 -3.16167 1.26543
C -2.17736 -0.69088 2.57774
H -3.06355 -1.01415 3.15860
H -1.34764 -1.37580 2.81130
H -1.89859 0.31961 2.91634
C -3.79954 1.59448 1.10331
H -4.36153 1.34370 2.02072
H -2.93345 2.23079 1.37047
H -4.46543 2.16969 0.44080
H 0.39985 0.31606 -2.19232
C -0.68639 4.79732 1.63882
H -0.14594 5.08168 2.55969
H -0.67644 5.67821 0.97228
H -1.73320 4.58655 1.91053
C 1.48080 -1.82064 0.37275
C 1.87896 -3.18044 0.36461
C 1.91674 -1.02036 1.45641
C 2.68906 -3.71688 1.37726
H 1.55078 -3.82416 -0.46125
C 2.72763 -1.55378 2.46836
H 1.59113 0.02124 1.51148
C 3.12082 -2.90252 2.43554
H 2.98103 -4.77230 1.33828
H 3.04981 -0.90985 3.29425
H 3.75122 -3.31526 3.23040
H 0.17117 -2.20934 -1.23831

E$_{cis}$'
SCF (BP86) Energy = -1305.85203095
Enthalpy 0K = -1305.351705
Energy 298K = -1305.320174
Free Energy 298K = -1305.413138
Lowest Frequency = 23.4230 cm^{-1}
SCF (DCE) Energy = -1305.86243739
SCF (BP86-D3) Energy = -1305.94722670
SCF (BS2) Energy = -1305.80756382

Rh -1.10322 -0.38186 0.04190
C -2.84837 0.01866 1.35139
C -2.75599 1.02726 0.31365
C -3.01007 0.36651 -0.98183
C -3.18002 -1.02388 -0.74243

C -3.03750 -1.25770 0.70176
C   2.31810 -0.85179 -1.21245
C   3.51931 -1.07078 -1.93938
C   1.90583 -1.88629 -0.32065
C   4.28165 -2.23220 -1.79538
H   3.84607 -0.30727 -2.65031
C   2.66872 -3.06704 -0.20842
C   3.85884 -3.24798 -0.92202
H   5.20120 -2.35100 -2.37832
H   2.31846 -3.85379 0.47148
H   4.43970 -4.16836 -0.80556
C   2.07508 1.54910 -2.12125
C   1.02079 2.47289 -2.09265
H   3.06674 1.69587 -2.54650
N   0.30132 0.69235 -1.04319
N -0.04690 1.93871 -1.43743
C   1.59625 0.40789 -1.43529
C   0.64187 -1.81027 0.49992
C   0.38891 -0.68258 1.47310
H   0.39524 -2.79436 0.93349
C -3.24650 -2.58054 1.38853
H -4.32168 -2.76608 1.57319
H -2.87151 -3.41902 0.77851
H -2.73545 -2.61797 2.36432
C -3.49904 -2.08375 -1.76143
H -3.00182 -3.03949 -1.52812
H -4.58739 -2.28104 -1.79421
H -3.18407 -1.78053 -2.77190
C -2.81484 0.26282 2.83473
H -3.83860 0.41386 3.22558
H -2.37790 -0.58870 3.38189
H -2.22453 1.15805 3.08423
C -2.61788 2.51099 0.48399
H -3.56321 3.02608 0.22806
H -2.35806 2.77877 1.52059
H -1.82178 2.87845 -0.18967
C -3.06462 1.09467 -2.29093
H -3.95274 1.75242 -2.33497
H -2.16084 1.72308 -2.39478
H -3.11344 0.40004 -3.14416
H -0.19987 -1.80444 -0.41404
C   0.94567 3.86182 -2.66227
H   1.77690 4.49511 -2.30493
H   0.99032 3.85982 -3.76675
H -0.00095 4.34034 -2.36326
C   1.45346 0.31547 1.84246
C   2.72151 -0.12965 2.28025
C   1.18170 1.69920 1.88196
C   3.68550 0.78048 2.74094
H   2.95462 -1.19960 2.25799
C   2.14284 2.60939 2.34651
H   0.21674 2.05491 1.50875
C   3.39882 2.15411 2.77916
H   4.66315 0.41271 3.07153
H   1.91255 3.68040 2.35962
H   4.15086 2.86504 3.13793
H -0.13488 -1.03806 2.37767

TS(E-G)$_{cis}$′
SCF (BP86) Energy = -1305.85048980
Enthalpy 0K = -1305.352348
Energy 298K = -1305.321125
Free Energy 298K = -1305.413239
Lowest Frequency = -535.4640 cm^{-1}
SCF (DCE) Energy = -1305.86068862
SCF (BP86-D3) Energy = -1305.94618767
SCF (BS2) Energy = -1305.80578024

Rh 1.08166 -0.38235 -0.04947
C   2.86611 0.25918 -1.28640
C   2.75311 1.11283 -0.13221
C   2.94042 0.27603 1.06375
C   3.14772 -1.07361 0.63661
C   3.05378 -1.10307 -0.82092
C -2.28495 -1.07141 1.04941
C -3.46677 -1.45541 1.73621
C -1.79719 -1.96095 0.04472
C -4.14146 -2.64596 1.45457
H -3.84513 -0.80200 2.52733
C -2.46959 -3.17656 -0.20373
C -3.64208 -3.52332 0.47728
H -5.04979 -2.89832 2.01201
H -2.06383 -3.85319 -0.96612
H -4.15136 -4.46652 0.25578
C -2.18558 1.24142 2.20237
C -1.16638 2.20115 2.29564
H -3.19428 1.30753 2.60775
N -0.35451 0.56341 1.09928
N -0.06045 1.77253 1.62379
C -1.64257 0.19276 1.42327
C -0.56665 -1.69247 -0.77694
C -0.37863 -0.47987 -1.59046
H -0.18544 -2.61111 -1.24784
C   3.30941 -2.30533 -1.68998
H   4.38901 -2.42051 -1.90379
H   2.97117 -3.23567 -1.20484
H   2.79193 -2.22458 -2.65999
C   3.47125 -2.25242 1.51495
H   3.05926 -3.19030 1.10902
H   4.56634 -2.38391 1.60282
H   3.06787 -2.12180 2.53100
C   2.85716 0.69827 -2.72492
H   3.88921 0.87275 -3.08282
H   2.40906 -0.06270 -3.38523
H   2.29212 1.63335 -2.86101
C   2.59444 2.60453 -0.09638
H   3.51023 3.08919 0.29133
H   2.39609 3.01689 -1.09874
H   1.75034 2.86341 0.56988
C   2.95728 0.81218 2.46489
H   3.85843 1.43071 2.63503
H   2.06513 1.44355 2.62650
H   2.95589 0.00271 3.21162
H   0.33064 -1.74500 0.40109
C -1.15985 3.53007 2.99799
H -1.98237 4.18039 2.65074
H -1.27147 3.42100 4.09217
H -0.20850 4.05176 2.80520
C -1.43549 0.56508 -1.80647
C -2.73883 0.18595 -2.19886
C -1.12650 1.94071 -1.74936
C -3.70245 1.15414 -2.52185
H -2.99724 -0.87672 -2.25204
C -2.08799 2.90822 -2.07395
H -0.13067 2.24234 -1.41044
C -3.38042 2.51891 -2.46297
H -4.70786 0.83826 -2.82115
H -1.82969 3.97105 -2.01274
H -4.13263 3.27463 -2.71337
H   0.21377 -0.65924 -2.50214

G$_{cis}$′
SCF (BP86) Energy = -1305.86094658
Enthalpy 0K = -1305.361360
Energy 298K = -1305.329741
Free Energy 298K = -1305.422962
Lowest Frequency = 23.2437 cm^{-1}
SCF (DCE) Energy = -1305.87140139
SCF (BP86-D3) Energy = -1305.95796345
SCF (BS2) Energy = -1305.81512665

Rh -1.04685 0.42479 0.04582
C -2.81720 -0.58137 -1.16447
C -2.75703 -1.17772 0.13125

C -2.89846 -0.11551 1.12635
C -3.11962 1.13541 0.41265
C -3.00316 0.86098 -0.99946
C   2.26017 1.24226 0.85685
C   3.41104 1.77237 1.49238
C   1.66703 2.00884 -0.19601
C   3.96502 3.00341 1.12803
H   3.85826 1.20521 2.31425
C   2.22095 3.26780 -0.52253
C   3.36185 3.76810 0.11618
H   4.85383 3.37489 1.64908
H   1.74501 3.85449 -1.31802
H   3.76996 4.74327 -0.16856
C   2.35332 -1.01080 2.15237
C   1.37100 -1.99074 2.37805
H   3.38690 -1.01712 2.49629
N   0.44412 -0.47180 1.12568
N   0.20977 -1.64030 1.75516
C   1.72847 -0.03516 1.34239
C   0.49026 1.57700 -1.00491
C   0.33604 0.30916 -1.65210
H -0.03002 2.42166 -1.47624
C -3.22994 1.83929 -2.12020
H -4.27287 1.79127 -2.48685
H -3.04073 2.87512 -1.79652
H -2.57430 1.63169 -2.98303
C -3.51383 2.45181 1.02886
H -3.12450 3.30614 0.45322
H -4.61582 2.54290 1.06171
H -3.13879 2.54394 2.05959
C -2.80520 -1.29219 -2.48891
H -3.83499 -1.39505 -2.88046
H -2.22800 -0.74080 -3.25067
H -2.37496 -2.30207 -2.40943
C -2.57492 -2.62595 0.47312
H -3.42285 -2.99421 1.07873
H -2.50525 -3.25456 -0.42878
H -1.64496 -2.73950 1.06609
C -2.97378 -0.35299 2.60914
H -3.93750 -0.81939 2.88870
H -2.15829 -1.02562 2.92296
H -2.87761 0.58717 3.17450
H -0.65648 1.58777 1.00409
C   1.45680 -3.27255 3.15832
H   2.16123 -3.98895 2.69780
H   1.79873 -3.10073 4.19449
H   0.46602 -3.75302 3.20138
C   1.37298 -0.76428 -1.76372
C   2.70177 -0.42831 -2.10635
C   1.03004 -2.12998 -1.66266
C   3.65714 -1.42965 -2.33894
H   2.98208 0.62572 -2.19627
C   1.98496 -3.12979 -1.89056
H   0.01110 -2.39974 -1.36781
C   3.30320 -2.78342 -2.23212
H   4.68182 -1.14811 -2.60478
H   1.70115 -4.18344 -1.79491
H   4.04972 -3.56467 -2.41067
H -0.37051 0.30870 -2.49555

TS(E1-E2'')$_{2,1}$
SCF (BP86) Energy = -1305.84993399
Enthalpy 0K = -1305.347756
Energy 298K = -1305.316892
Free Energy 298K = -1305.407726
Lowest Frequency = -11.2681 cm^{-1}
SCF (DCE) Energy = -1305.85982918
SCF (BP86-D3) Energy = -1305.94616770
SCF (BS2) Energy = -1305.80399110

Rh 0.07885 -0.53364 0.13568
C -1.33768 -2.14304 0.66786
C -0.01064 -2.63136 0.24768
C   0.96140 -2.30145 1.30932
C   0.29536 -1.46473 2.23315
C -1.12844 -1.34680 1.83371
C   1.73512 2.23078 -0.00199
C   2.37022 2.96755 1.02397
C   0.39926 2.56653 -0.38217
C   1.70336 4.00402 1.69026
H   3.39861 2.70545 1.29542
C -0.24973 3.61568 0.30178
C   0.38183 4.32426 1.33531
H   2.21345 4.56240 2.48268
H -1.25924 3.90411 -0.01460
H -0.14585 5.13868 1.84318
C   3.74854 1.00392 -1.18997
C   3.80884 -0.31863 -1.68828
H   4.51915 1.77367 -1.22739
N   1.80646 -0.06775 -0.87172
N   2.62910 -0.96483 -1.47125
C   2.44417 1.13230 -0.68090
C -0.24850 1.90550 -1.59478
C -0.92217 0.50265 -1.41975
H -0.69603 -0.09107 -2.32276
H   0.53828 1.79323 -2.36039
C -2.17997 -0.64933 2.64799
H -2.54020 -1.30607 3.46328
H -1.78377 0.26684 3.11606
H -3.04556 -0.36679 2.02955
C   0.88184 -0.78516 3.43589
H   0.63627 0.29066 3.44592
H   0.47732 -1.22183 4.36829
H   1.97782 -0.88006 3.46211
C -2.64980 -2.51916 0.05133
H -3.01049 -3.46862 0.49173
H -3.41880 -1.75016 0.21811
H -2.56354 -2.66797 -1.03612
C   0.30503 -3.50456 -0.92817
H   0.47188 -4.54975 -0.60315
H -0.51222 -3.50177 -1.66584
H   1.22589 -3.13982 -1.41885
C   2.40007 -2.72038 1.28964
H   2.48879 -3.81504 1.41732
H   2.85680 -2.44669 0.32031
H   2.97694 -2.23741 2.09369
H -1.00152 2.60438 -2.00529
C   4.93564 -1.01867 -2.39612
H   5.15094 -0.56399 -3.38033
H   5.87225 -0.98393 -1.81141
H   4.67249 -2.07543 -2.56371
C -2.41024 0.56050 -1.24402
C -3.26246 -0.10694 -2.15790
C -3.02978 1.31103 -0.21196
C -4.66014 -0.04214 -2.04440
H -2.81114 -0.67402 -2.98146
C -4.42612 1.38158 -0.09733
H -2.39970 1.84348 0.50841
C -5.25185 0.70152 -1.01071
H -5.28875 -0.56720 -2.77240
H -4.87432 1.97473 0.70811
H -6.34170 0.75907 -0.92167

E2''$_{2,1}$
SCF (BP86) Energy = -1305.85124796
Enthalpy 0K = -1305.349330
Energy 298K = -1305.317429
Free Energy 298K = -1305.411140
Lowest Frequency = 25.7895 cm^{-1}
SCF (DCE) Energy = -1305.85989881
SCF (BP86-D3) Energy = -1305.95053092
SCF (BS2) Energy = -1305.80424006

Rh 0.04906 -0.16908 0.31598

```
C  -1.40528 -0.84234  1.84236
C  -0.19092 -1.66163  1.79764
C   0.93084 -0.84144  2.30926
C   0.44342  0.47130  2.50814
C  -0.99928  0.49075  2.17679
C   2.01616  1.48077 -1.13672
C   2.88739  2.48387 -0.65390
C   0.62196  1.78742 -1.32597
C   2.40531  3.74898 -0.29797
H   3.94741  2.23881 -0.52923
C   0.16470  3.06987 -0.93326
C   1.03104  4.03744 -0.40910
H   3.09726  4.50953  0.07986
H  -0.88877  3.32450 -1.10098
H   0.64842  5.02431 -0.12851
C   3.63274 -0.54714 -1.79323
C   3.39132 -1.91595 -1.53244
H   4.50242 -0.09906 -2.27339
N   1.62986 -0.85836 -0.83308
N   2.17500 -2.08675 -0.92841
C   2.47124  0.10100 -1.33360
C  -0.29277  0.89883 -2.20513
C  -0.98935 -0.30469 -1.51763
H  -0.66055 -1.25322 -1.96882
H   0.33945  0.51270 -3.02381
C  -1.90086  1.67889  2.36472
H  -2.23307  1.76091  3.41770
H  -1.38332  2.61753  2.10780
H  -2.80015  1.60068  1.73351
C   1.21620  1.67548  2.96341
H   1.08806  2.52373  2.26828
H   0.87041  2.00967  3.95962
H   2.29358  1.46149  3.03348
C  -2.80863 -1.33743  1.66975
H  -3.20681 -1.67995  2.64403
H  -3.47726 -0.55248  1.28404
H  -2.86073 -2.18433  0.96876
C  -0.09520 -3.13049  1.50833
H   0.04785 -3.70703  2.44262
H  -1.00737 -3.50149  1.01562
H   0.76166 -3.32650  0.83893
C   2.32647 -1.36101  2.49396
H   2.37290 -2.06051  3.34933
H   2.65481 -1.90754  1.59227
H   3.04453 -0.54784  2.68313
H  -1.04829  1.56339 -2.66095
C   4.24703 -3.10947 -1.85479
H   4.25637 -3.33237 -2.93775
H   5.29575 -2.95547 -1.54556
H   3.85896 -3.99941 -1.33390
C  -2.47668 -0.26753 -1.44564
C  -3.22307 -1.44555 -1.70200
C  -3.21103  0.91011 -1.15115
C  -4.62505 -1.45144 -1.66200
H  -2.68162 -2.36612 -1.95201
C  -4.61302  0.90882 -1.11341
H  -2.66965  1.84037 -0.94618
C  -5.33111 -0.27345 -1.36650
H  -5.16945 -2.37851 -1.87399
H  -5.15002  1.83796 -0.89062
H  -6.42570 -0.27317 -1.34151
```

TS(E2″-E_{trans})

SCF (BP86) Energy = -1305.84710904
Enthalpy 0K = -1305.345060
Energy 298K = -1305.314086
Free Energy 298K = -1305.405636
Lowest Frequency = -14.5343 cm^{-1}
SCF (DCE) Energy = -1305.85720845
SCF (BP86-D3) Energy = -1305.93982691
SCF (BS2) Energy = -1305.80147454

```
Rh -0.11237 -0.40242  0.44549
C  -2.02791 -1.15931  1.26258
C  -1.20402 -2.19284  0.60911
C  -0.02212 -2.45058  1.45838
C  -0.02504 -1.47299  2.48011
C  -1.25395 -0.65229  2.34920
C   2.62712  1.40963 -0.42906
C   3.97953  1.66704 -0.08296
C   1.61704  2.28837  0.06470
C   4.34966  2.73280  0.74139
H   4.74108  0.97673 -0.46033
C   2.01613  3.33589  0.92910
C   3.35287  3.56819  1.27128
H   5.40431  2.89615  0.98723
H   1.24221  4.01217  1.31408
H   3.61318  4.39696  1.93813
C   3.13320 -0.38540 -2.26438
C   2.52082 -1.63630 -2.50909
H   3.99701  0.04941 -2.76708
N   1.40148 -0.70871 -0.88444
N   1.47001 -1.82038 -1.66641
C   2.37569  0.19393 -1.22904
C   0.14061  2.34120 -0.36129
C  -0.60481  1.11525 -0.93385
H  -0.12771  0.79632 -1.87274
H   0.07132  3.14306 -1.13122
C  -1.65688  0.42678  3.31426
H  -2.08697 -0.00968  4.23620
H  -0.79227  1.04065  3.61650
H  -2.41366  1.09582  2.87584
C   1.01905 -1.25431  3.53647
H   1.31269 -0.19221  3.59589
H   0.63697 -1.54431  4.53336
H   1.92758 -1.84220  3.33641
C  -3.44194 -0.81825  0.90686
H  -4.12993 -1.55812  1.35887
H  -3.72785  0.17972  1.27063
H  -3.60340 -0.82549 -0.18261
C  -1.57399 -3.01347 -0.58766
H  -1.92125 -4.01874 -0.27933
H  -2.37748 -2.53411 -1.16805
H  -0.68917 -3.12930 -1.23896
C   1.01318 -3.49091  1.15553
H   0.58761 -4.50647  1.25354
H   1.37364 -3.36577  0.11637
H   1.87683 -3.41724  1.83475
H  -0.41598  2.74033  0.50550
C   2.87907 -2.69257 -3.51694
H   2.85664 -2.29915 -4.54901
H   3.89290 -3.09852 -3.34686
H   2.16252 -3.52712 -3.45392
C  -2.05871  1.34479 -1.20606
C  -2.67120  0.64423 -2.27829
C  -2.87538  2.24316 -0.47567
C  -4.02493  0.81958 -2.59535
H  -2.05640 -0.04236 -2.87283
C  -4.23057  2.42587 -0.79524
H  -2.44722  2.82109  0.35046
C  -4.81707  1.71419 -1.85355
H  -4.46129  0.26593 -3.43436
H  -4.83005  3.13745 -0.21593
H  -5.87253  1.86173 -2.10465
```

E_{trans}′

SCF (BP86) Energy = -1305.86338499
Enthalpy 0K = -1305.361869
Energy 298K = -1305.330634
Free Energy 298K = -1305.422126
Lowest Frequency = 29.6598 cm^{-1}
SCF (DCE) Energy = -1305.87277309
SCF (BP86-D3) Energy = -1305.95614294
SCF (BS2) Energy = -1305.81802386

```
Rh -0.27162 -0.59833  0.38598
C  -2.31494 -1.47779  0.09370
C  -1.28506 -2.39902 -0.34333
C  -0.51101 -2.80935  0.84387
C  -1.00861 -2.08588  1.96374
C  -2.10507 -1.22437  1.49890
C   2.81491  1.40865  0.19113
C   4.10939  1.98251  0.30439
C   1.74321  2.09675  0.83163
C   4.34049  3.17531  0.99464
H   4.95488  1.45840 -0.14949
C   1.99350  3.28538  1.54555
C   3.27603  3.84132  1.62355
H   5.35708  3.57869  1.05214
H   1.15215  3.78998  2.03618
H   3.44175  4.77374  2.17235
C   3.63543 -0.40788 -1.44302
C   3.06609 -1.60661 -1.89734
H   4.59934  0.01299 -1.72560
N   1.59644 -0.70771 -0.54426
N   1.83346 -1.77305 -1.34468
C   2.67477  0.16046 -0.57375
C   0.31970  1.59312  0.80046
C  -0.32583  1.31808 -0.53112
H   0.38003  1.30183 -1.37101
H  -0.31984  2.19784  1.46279
C  -2.98750 -0.39247  2.38916
H  -3.78605 -1.01253  2.83949
H  -2.41965  0.05931  3.21959
H  -3.47384  0.41959  1.82641
C  -0.55026 -2.20830  3.39125
H  -0.60350 -1.24390  3.92215
H  -1.18699 -2.92307  3.94648
H   0.48846 -2.56836  3.45284
C  -3.43670 -0.94340 -0.74641
H  -4.31880 -1.60679 -0.66826
H  -3.74275  0.06635 -0.43068
H  -3.15378 -0.88418 -1.80865
C  -1.11691 -2.99733 -1.70746
H  -1.40779 -4.06496 -1.70609
H  -1.73540 -2.47697 -2.45557
H  -0.05534 -2.91735 -2.00559
C   0.59056 -3.82517  0.81574
H   0.18121 -4.83554  0.62836
H   1.29423 -3.57882 -0.00007
H   1.14552 -3.85427  1.76660
H   0.42716  0.58140  1.48922
C   3.62139 -2.63638 -2.84121
H   3.92086 -2.18835 -3.80529
H   4.51351 -3.13984 -2.42612
H   2.86152 -3.40789 -3.04519
C  -1.62367  1.92317 -0.90353
C  -1.97712  1.97343 -2.27682
C  -2.54569  2.47146  0.02410
C  -3.18802  2.53458 -2.70142
H  -1.27590  1.56577 -3.01439
C  -3.75630  3.03941 -0.40064
H  -2.31168  2.46663  1.09426
C  -4.08825  3.07280 -1.76547
H  -3.42697  2.56066 -3.77022
H  -4.44172  3.46559  0.34045
H  -5.03166  3.51941 -2.09545
```

TS(E-G)$_{trans}$'
SCF (BP86) Energy = -1305.86043198
Enthalpy 0K = -1305.361754
Energy 298K = -1305.330601
Free Energy 298K = -1305.422107
Lowest Frequency = -571.9853 cm^{-1}
SCF (DCE) Energy = -1305.86950863
SCF (BP86-D3) Energy = -1305.95358474
SCF (BS2) Energy = -1305.81464528

```
Rh  0.20322 -0.55384 -0.43070
C   2.28364 -1.51222 -0.17346
C   1.26330 -2.44082  0.22900
C   0.42757 -2.72828 -0.94634
C   0.93087 -1.96066 -2.04761
C   2.05229 -1.16179 -1.56307
C  -2.79829  1.42281 -0.12201
C  -4.08550  2.00393 -0.25791
C  -1.71176  2.07362 -0.77861
C  -4.30154  3.16909 -0.99930
H  -4.93565  1.50493  0.21617
C  -1.95026  3.23034 -1.54962
C  -3.22812  3.79183 -1.65749
H  -5.31277  3.58230 -1.07701
H  -1.10263  3.70734 -2.05676
H  -3.38374  4.69823 -2.25107
C  -3.59340 -0.35723  1.57865
C  -3.01532 -1.55864  2.01837
H  -4.54021  0.07799  1.89605
N  -1.59929 -0.68471  0.60004
N  -1.80816 -1.74302  1.41371
C  -2.66388  0.19194  0.66498
C  -0.29015  1.59794 -0.68573
C   0.34595  1.34584  0.60694
H  -0.33584  1.27589  1.46274
H   0.34800  2.09628 -1.42883
C   2.95707 -0.31468 -2.41633
H   3.75802 -0.92848 -2.87118
H   2.40611  0.16631 -3.24139
H   3.44256  0.47654 -1.82349
C   0.44972 -2.02084 -3.47263
H   0.57681 -1.05553 -3.98812
H   1.02128 -2.77777 -4.04238
H  -0.61534 -2.29427 -3.52901
C   3.43547 -1.03877  0.66242
H   4.33228 -1.65244  0.45404
H   3.69218  0.01176  0.45225
H   3.21629 -1.11887  1.73793
C   1.10688 -3.11486  1.55979
H   1.32253 -4.19688  1.47937
H   1.78913 -2.68864  2.31213
H   0.06597 -2.98614  1.91041
C  -0.69778 -3.72064 -0.95638
H  -0.30561 -4.75348 -0.89840
H  -1.35175 -3.54719 -0.08393
H  -1.30485 -3.63957 -1.87172
H  -0.59731  0.33795 -1.49958
C  -3.53855 -2.57531  2.99431
H  -3.76280 -2.12285  3.97674
H  -4.46930 -3.05153  2.63610
H  -2.78926 -3.36848  3.14775
C   1.68386  1.86093  0.97035
C   2.08112  1.80457  2.33059
C   2.58969  2.44681  0.05038
C   3.32141  2.30141  2.75120
H   1.39266  1.36482  3.06156
C   3.83130  2.94615  0.47142
H   2.31683  2.53107 -1.00683
C   4.20740  2.87557  1.82352
H   3.59547  2.24606  3.81032
H   4.50533  3.40310 -0.26159
H   5.17467  3.27030  2.15049
```

G$_{trans}$'
SCF (BP86) Energy = -1305.86848285
Enthalpy 0K = -1305.368200
Energy 298K = -1305.336750
Free Energy 298K = -1305.428791
Lowest Frequency = 29.9060 cm^{-1}
SCF (DCE) Energy = -1305.87753855

SCF (BP86-D3) Energy = -1305.96227291
SCF (BS2) Energy = -1305.82141625

Rh	0.09985	-0.51497	-0.51174
C	2.29336	-1.45581	-0.25767
C	1.31328	-2.44229	0.06429
C	0.44307	-2.61299	-1.09887
C	0.94581	-1.75165	-2.15865
C	2.04434	-0.98581	-1.61871
C	-2.80922	1.35441	-0.02726
C	-4.11962	1.86743	-0.18793
C	-1.74392	1.99925	-0.73087
C	-4.39261	2.96349	-1.01331
H	-4.94208	1.36442	0.33001
C	-2.04632	3.08279	-1.58537
C	-3.34991	3.57336	-1.72933
H	-5.42090	3.32678	-1.11304
H	-1.22404	3.56241	-2.13036
H	-3.54814	4.42115	-2.39312
C	-3.47121	-0.36967	1.80739
C	-2.84379	-1.55549	2.23033
H	-4.39875	0.06224	2.18172
N	-1.55690	-0.69736	0.69707
N	-1.68341	-1.74305	1.53936
C	-2.62159	0.16704	0.81380
C	-0.30546	1.63206	-0.59396
C	0.32784	1.36714	0.66150
H	-0.32901	1.21294	1.52501
H	0.31680	2.09212	-1.37335
C	2.94077	-0.06342	-2.39885
H	3.77573	-0.62524	-2.86005
H	2.39443	0.44035	-3.21267
H	3.38294	0.71124	-1.75281
C	0.49493	-1.75275	-3.59563
H	0.58653	-0.75544	-4.05363
H	1.11555	-2.45176	-4.18752
H	-0.55406	-2.07213	-3.69208
C	3.45702	-1.03828	0.59050
H	4.36723	-1.58549	0.27885
H	3.67062	0.03816	0.49536
H	3.28525	-1.25288	1.65598
C	1.17236	-3.21645	1.34067
H	1.28711	-4.30012	1.15460
H	1.92842	-2.91717	2.08330
H	0.16432	-3.03765	1.76323
C	-0.65166	-3.63882	-1.19269
H	-0.23042	-4.65708	-1.29300
H	-1.27338	-3.60917	-0.28236
H	-1.30483	-3.45661	-2.06051
H	-1.01346	-0.29663	-1.57038
C	-3.27811	-2.55244	3.26831
H	-3.39661	-2.08525	4.26241
H	-4.24601	-3.01986	3.01184
H	-2.52687	-3.35361	3.35636
C	1.68526	1.82002	1.03730
C	2.11715	1.62241	2.37299
C	2.55793	2.51379	0.16210
C	3.36391	2.08605	2.81311
H	1.45135	1.09970	3.06942
C	3.80781	2.97498	0.60136
H	2.24501	2.72054	-0.86630
C	4.22016	2.76313	1.92826
H	3.66651	1.92255	3.85301
H	4.45754	3.51771	-0.09400
H	5.19276	3.13112	2.27041

1,2-insertion

D1$_{1,2}$
SCF Energy = -1305.86228788
Enthalpy 0K = -1305.360983
Energy 298K = -1305.328943
Free Energy 298K = -1305.423040
Lowest Frequency = 20.2186 cm^{-1}
SCF (BS2) Energy = -1305.81662464
SCF (BP86-D3) Energy = -1305.96290951
SCF (DCE) Energy = -1305.87160037

Rh	0.21393	-0.45849	0.01295
C	-1.05132	-1.66770	1.51001
C	-0.49848	-2.59792	0.53535
C	0.93764	-2.54041	0.59821
C	1.28692	-1.57008	1.62879
C	0.06132	-1.07045	2.21487
C	1.30251	2.31120	0.23970
C	1.39585	3.66349	0.63348
C	0.14601	1.54792	0.58542
C	0.35325	4.26356	1.35574
H	2.28826	4.24173	0.36785
C	-0.89580	2.16898	1.28788
C	-0.79363	3.52172	1.68047
H	0.43336	5.31325	1.65827
H	-1.81041	1.61228	1.52327
H	-1.61581	3.98952	2.23415
C	3.57060	1.78018	-1.09252
C	3.90467	0.53107	-1.66652
H	4.16354	2.69431	-1.09316
N	1.94884	0.27911	-0.78126
N	2.90344	-0.37875	-1.46689
C	2.29945	1.57886	-0.52389
C	-1.06056	0.38685	-1.66978
C	-0.32090	-0.75387	-2.06809
H	0.60955	-0.63665	-2.63126
H	-0.59671	1.36183	-1.85651
H	-0.82337	-1.71934	-2.19612
C	-2.52820	0.44700	-1.47041
C	-3.12643	1.68232	-1.11977
C	-3.38467	-0.66047	-1.68787
C	-4.51454	1.79713	-0.96101
H	-2.48505	2.55652	-0.96971
C	-4.77327	-0.54519	-1.53168
H	-2.96229	-1.61330	-2.02244
C	-5.34645	0.68297	-1.15951
H	-4.94940	2.76530	-0.69017
H	-5.41293	-1.41471	-1.71894
H	-6.43138	0.77362	-1.04336
C	-0.02592	-0.19043	3.42748
H	-0.06188	-0.81582	4.33946
H	0.84498	0.47742	3.50761
H	-0.92598	0.44218	3.41534
C	2.68972	-1.25374	2.06288
H	2.74703	-0.27847	2.57015
H	3.06137	-2.02395	2.76466
H	3.36676	-1.22588	1.19413
C	-2.50805	-1.52091	1.85057
H	-2.86460	-2.37818	2.45299
H	-2.69019	-0.60741	2.43834
H	-3.13493	-1.46247	0.94486
C	-1.31070	-3.56309	-0.28084
H	-1.50768	-4.47855	0.30833
H	-2.28891	-3.14038	-0.55825
H	-0.78883	-3.87209	-1.20040
C	1.93049	-3.34085	-0.19753
H	1.42899	-3.97017	-0.94976
H	2.62604	-2.66386	-0.72790
H	2.51754	-4.00749	0.46055
C	5.13956	0.13429	-2.42744
H	5.09772	-0.93871	-2.67360
H	5.23843	0.69604	-3.37416
H	6.05929	0.31832	-1.84370

TS(D1-E1)$_{1,2}$

SCF Energy = -1305.82989340
Enthalpy 0K = -1305.329840
Energy 298K = -1305.298352
Free Energy 298K = -1305.391393
Lowest Frequency = -265.5985 cm^{-1}
SCF (DCE) Energy = -1305.83890421
SCF (BP86-D3) Energy = -1305.92724543
SCF (BS2) Energy = -1305.78430266

Rh	0.52061	-0.59182	-0.16024
C	0.01283	-2.51682	0.91963
C	0.67219	-2.81401	-0.33316
C	1.98297	-2.20133	-0.30379
C	2.15209	-1.58812	1.02877
C	0.95520	-1.79261	1.77506
C	0.12229	2.20639	0.82606
C	-0.14794	3.11168	1.87208
C	-0.72629	1.05167	0.63830
C	-1.19618	2.86971	2.77178
H	0.50243	3.98360	2.00383
C	-1.76708	0.83005	1.57260
C	-1.98908	1.71329	2.63923
H	-1.38832	3.57626	3.58636
H	-2.44328	-0.01934	1.42959
H	-2.80159	1.51788	3.34743
C	2.26250	3.21862	-0.41397
C	3.15565	2.49313	-1.22966
H	2.30942	4.27367	-0.14548
N	1.62729	1.07781	-0.62591
N	2.75556	1.18550	-1.34448
C	1.28477	2.27415	-0.03712
C	-1.26389	0.76284	-1.15459
C	-0.43869	-0.17807	-1.95007
H	0.25328	0.28083	-2.66710
H	-1.02874	1.80565	-1.40810
H	-0.94796	-1.06546	-2.34893
C	-2.75114	0.53251	-1.11597
C	-3.61860	1.64518	-1.14245
C	-3.31896	-0.75848	-1.08486
C	-5.01064	1.47449	-1.14925
H	-3.19210	2.65471	-1.15925
C	-4.71094	-0.93234	-1.08104
H	-2.66096	-1.63351	-1.06130
C	-5.56314	0.18389	-1.11542
H	-5.66488	2.35222	-1.18029
H	-5.13227	-1.94322	-1.05608
H	-6.64963	0.04809	-1.11797
C	0.67794	-1.36993	3.18975
H	0.62342	-2.24653	3.86206
H	1.46687	-0.70381	3.57187
H	-0.28002	-0.82672	3.26764
C	3.40930	-0.89547	1.46890
H	3.25226	-0.30485	2.38472
H	4.20792	-1.63336	1.67156
H	3.77049	-0.21484	0.67887
C	-1.31361	-3.05618	1.38035
H	-1.17727	-4.00589	1.93309
H	-1.82310	-2.35281	2.05832
H	-1.99091	-3.26375	0.53677
C	0.12009	-3.66604	-1.44271
H	0.37897	-4.72951	-1.28302
H	-0.97907	-3.60231	-1.50127
H	0.52634	-3.36819	-2.42230
C	3.05775	-2.26741	-1.35201
H	2.66249	-2.64940	-2.30676
H	3.47070	-1.25982	-1.53796
H	3.88190	-2.93529	-1.03649
C	4.38567	2.96727	-1.95256
H	4.13755	3.64355	-2.79119
H	5.06791	3.51826	-1.28150
H	4.93050	2.10419	-2.36708

E1$_{1,2}$
SCF Energy = -1305.85123542
Enthalpy 0K = -1305.349955
Energy 298K = -1305.317882
Free Energy 298K = -1305.413552
Lowest Frequency = 15.7827 cm^{-1}
SCF (DCE) Energy = -1305.86198632
SCF (BP86-D3) Energy = -1305.93842986
SCF (BS2) Energy = -1305.80715043

Rh	1.16217	-0.49755	0.13155
C	2.68069	-1.22369	1.48842
C	1.96922	-2.37134	0.93532
C	2.14844	-2.31298	-0.49177
C	3.08267	-1.19765	-0.79742
C	3.44394	-0.56667	0.42270
C	-1.25554	2.17283	0.72737
C	-1.45772	3.20518	1.67529
C	-2.14301	1.06145	0.71801
C	-2.50152	3.14866	2.60635
H	-0.76411	4.05277	1.67704
C	-3.18337	1.01845	1.66645
C	-3.36712	2.04406	2.60556
H	-2.63389	3.95965	3.33058
H	-3.87816	0.17244	1.64844
H	-4.18935	1.98254	3.32675
C	0.24197	3.52074	-0.87486
C	1.41877	3.18773	-1.59498
H	-0.27403	4.47979	-0.83929
N	0.78216	1.35963	-0.56807
N	1.74436	1.89390	-1.39095
C	-0.15030	2.33929	-0.23694
C	-1.93928	-0.06940	-0.29172
C	-0.85528	-1.04309	0.22179
H	-0.90233	-1.98252	-0.36445
H	-1.55621	0.39895	-1.21913
H	-1.04481	-1.30322	1.28319
C	-3.25157	-0.76124	-0.67952
C	-4.03887	-0.21961	-1.71835
C	-3.71709	-1.92741	-0.03665
C	-5.24761	-0.81796	-2.10282
H	-3.69250	0.68650	-2.22998
C	-4.92897	-2.52931	-0.41553
H	-3.12111	-2.37199	0.76768
C	-5.69917	-1.97782	-1.45089
H	-5.83711	-0.38015	-2.91598
H	-5.26991	-3.43429	0.09985
H	-6.64139	-2.44904	-1.75042
C	4.34905	0.62496	0.55452
H	5.40507	0.34333	0.38479
H	4.07127	1.39561	-0.18477
H	4.28353	1.07494	1.55759
C	3.54756	-0.77738	-2.15928
H	4.64983	-0.82211	-2.22564
H	3.13066	-1.41735	-2.95187
H	3.22615	0.26707	-2.33788
C	2.73961	-0.85484	2.94133
H	3.60961	-1.34042	3.42433
H	2.84691	0.23320	3.07511
H	1.83369	-1.17649	3.47794
C	1.20965	-3.41091	1.70967
H	1.85909	-4.26729	1.97022
H	0.80902	-2.99973	2.65033
H	0.35675	-3.80201	1.13143
C	1.62949	-3.29355	-1.50166
H	0.73481	-3.81910	-1.13355
H	1.36427	-2.79340	-2.44704
H	2.39846	-4.05624	-1.72976
C	2.24854	4.05417	-2.49836
H	1.67236	4.39429	-3.37778
H	2.60593	4.95991	-1.97663
H	3.12493	3.49333	-2.85997

TS(E1-F1)$_{1,2}$
SCF Energy = -1305.77186291
Enthalpy 0K = -1305.272502
Energy 298K = -1305.240812
Free Energy 298K = -1305.334231
Lowest Frequency = -519.7601 cm^{-1}
SCF (DCE) Energy = -1305.77955784
SCF (BP86-D3) Energy = -1305.86659413
SCF (BS2) Energy = -1305.72327032

Rh 0.64284 -0.34468 -0.15541
C 0.66772 -2.30421 -1.26591
C 1.64607 -1.39895 -1.81346
C 2.75217 -1.24568 -0.83086
C 2.39980 -1.95022 0.33102
C 1.04766 -2.51311 0.11728
C 0.22055 0.93711 1.65117
C 0.72032 0.52366 2.93149
C -1.05684 0.39465 1.16136
C 0.03889 -0.40953 3.69496
H 1.65464 0.96986 3.28932
C -1.73011 -0.54627 2.01227
C -1.19448 -0.95376 3.22468
H 0.43357 -0.71513 4.66966
H -2.71170 -0.91709 1.70020
H -1.74174 -1.67123 3.84538
C 1.64960 3.16292 1.03939
C 2.00060 3.52407 -0.29958
H 1.96313 3.64717 1.96338
N 0.75614 1.78208 -0.46760
N 1.44939 2.68198 -1.21062
C 0.83157 2.04465 0.88585
C 1.70966 -0.90120 -3.23258
H 0.71087 -0.87824 -3.69924
H 2.12598 0.11808 -3.28035
H 2.34983 -1.55493 -3.85666
C 3.99369 -0.44035 -1.09174
H 4.62428 -0.91099 -1.86992
H 3.74195 0.57501 -1.44485
H 4.60701 -0.33544 -0.18247
C 3.16541 -2.07900 1.61522
H 3.46890 -3.12669 1.80227
H 4.07936 -1.46355 1.60661
H 2.55314 -1.76046 2.47988
C 0.35994 -3.41553 1.10268
H 0.89666 -4.37910 1.19712
H 0.31698 -2.95731 2.10602
H -0.67526 -3.63541 0.79700
C -0.47423 -2.94885 -2.00231
H -0.14857 -3.86306 -2.53676
H -1.28291 -3.24771 -1.31440
H -0.91147 -2.27415 -2.75922
C -1.81650 1.21989 0.09150
H -1.89792 2.25753 0.46876
C -0.92506 1.20951 -1.15153
H -0.93335 2.11847 -1.76796
H -1.07092 0.32704 -1.79107
C -3.22634 0.75249 -0.24859
C -4.32650 1.59960 -0.00796
C -3.46821 -0.51271 -0.82978
C -5.63187 1.20181 -0.33919
H -4.15512 2.58407 0.44302
C -4.77163 -0.91445 -1.15794
H -2.62592 -1.18796 -1.01556
C -5.85847 -0.05737 -0.91565
H -6.47143 1.87831 -0.14692
H -4.93966 -1.89996 -1.60562
H -6.87524 -0.37034 -1.17511
C 2.83859 4.68303 -0.76030
H 2.40571 5.64962 -0.44565
H 3.86143 4.63474 -0.34571
H 2.90943 4.67804 -1.85895

F1$_{1,2}$
SCF Energy = -1305.84086640
Enthalpy 0K = -1305.338344
Energy 298K = -1305.306739
Free Energy 298K = -1305.400182
Lowest Frequency = 21.4122 cm^{-1}
SCF (DCE) Energy = -1305.84826885
SCF (BP86-D3) Energy = -1305.93902456
SCF (BS2) Energy = -1305.79291102

Rh 0.64817 -0.31555 -0.06795
C 0.53827 -1.71867 -1.83186
C 1.70893 -0.87537 -1.92443
C 2.62834 -1.20239 -0.82591
C 1.98053 -2.16302 0.00312
C 0.65856 -2.44250 -0.57984
C 0.26414 0.58559 1.86118
C 0.70538 -0.04597 3.07882
C -1.03716 0.23370 1.24774
C -0.03712 -1.07086 3.62965
H 1.63302 0.29638 3.55028
C -1.74817 -0.85338 1.88840
C -1.25954 -1.49770 3.00859
H 0.29874 -1.55480 4.55294
H -2.72502 -1.13534 1.48060
H -1.83147 -2.31708 3.45736
C 2.23372 2.28771 1.23003
C 2.35725 2.99116 0.00523
H 2.95465 2.23809 2.04371
N 0.44289 1.95561 -0.13590
N 1.29338 2.83179 -0.81229
C 1.00297 1.61098 1.14695
C 2.02559 0.10756 -3.01758
H 1.20481 0.17655 -3.74872
H 2.19004 1.12012 -2.60432
H 2.94031 -0.18953 -3.56367
C 3.98281 -0.57997 -0.63211
H 4.61295 -0.69322 -1.53331
H 3.90404 0.50211 -0.41462
H 4.51963 -1.04510 0.21035
C 2.50832 -2.81181 1.25099
H 2.64349 -3.90064 1.11092
H 3.48180 -2.38747 1.54488
H 1.81559 -2.66925 2.10065
C -0.28481 -3.49134 -0.06231
H 0.08941 -4.50676 -0.29530
H -0.40161 -3.41736 1.03204
H -1.28554 -3.39013 -0.51133
C -0.52207 -1.91047 -2.88097
H -0.23939 -2.70762 -3.59680
H -1.48731 -2.20130 -2.43483
H -0.68944 -0.99178 -3.46777
C -1.86676 1.40128 0.64661
H -2.15096 2.04115 1.50736
C -1.00042 2.26514 -0.30403
H -1.15128 3.34279 -0.11093
H -1.23531 2.07202 -1.36171
C -3.16651 0.96157 -0.01556
C -4.40799 1.27876 0.56964
C -3.16005 0.22114 -1.21692
C -5.61323 0.87310 -0.02688
H -4.42844 1.85332 1.50351
C -4.36148 -0.18739 -1.81477
H -2.19849 -0.04243 -1.67064
C -5.59368 0.13856 -1.22256
H -6.56750 1.13494 0.44269
H -4.33698 -0.75952 -2.74883
H -6.53163 -0.17592 -1.69225
C 3.50405 3.85430 -0.44460
H 4.37305 3.24767 -0.75612

H   3.19590 4.47352 -1.30108
H   3.84204 4.51656 0.37002

TS(E1-E_{gem})
SCF Energy = -1305.84316648
Enthalpy 0K = -1305.341776
Energy 298K = -1305.310729
Free Energy 298K = -1305.403118
Lowest Frequency = -34.4877 cm^{-1}
SCF (DCE) Energy = -1305.85380234
SCF (BP86-D3) Energy = -1305.93135784
SCF (BS2) Energy = -1305.79778541

Rh 0.97746 -0.44535 0.20496
C   1.67325 -2.18565 1.26607
C   1.37540 -2.58568 -0.07879
C   2.35870 -1.93949 -0.98271
C   3.22127 -1.12965 -0.21864
C   2.72037 -1.15855 1.17030
C -1.38260 2.63975 0.20772
C -1.93915 3.90132 0.54890
C -2.26805 1.51883 0.17837
C -3.30396 4.07870 0.79451
H -1.26742 4.76010 0.63365
C -3.64310 1.71732 0.40407
C -4.17055 2.98062 0.70595
H -3.68465 5.07164 1.05666
H -4.31412 0.85505 0.35155
H -5.24451 3.09693 0.88568
C   0.74279 3.76618 -0.63918
C   2.06786 3.34351 -0.86650
H   0.32187 4.75001 -0.84109
N   0.96953 1.58476 -0.14327
N   2.19254 2.03431 -0.55860
C   0.05312 2.63640 -0.15803
C -1.71044 0.10505 -0.01310
C -0.79809 -0.24145 1.20104
H -1.07808 -1.18988 1.68724
H -1.06510 0.16399 -0.93018
H -0.78015 0.56675 1.94863
C -2.74531 -0.96799 -0.33920
C -2.98838 -1.32451 -1.68242
C -3.49009 -1.62211 0.66629
C -3.93537 -2.30583 -2.01471
H -2.42579 -0.81983 -2.47743
C -4.44168 -2.60134 0.33868
H -3.32894 -1.35316 1.71591
C -4.66580 -2.95008 -1.00317
H -4.10233 -2.56733 -3.06531
H -5.01075 -3.09247 1.13566
H -5.40469 -3.71701 -1.25820
C   3.38324 -0.45245 2.31616
H   4.29688 -0.99565 2.62805
H   3.67852 0.56741 2.02304
H   2.71651 -0.38251 3.18943
C   4.36860 -0.28908 -0.68714
H   5.25439 -0.43588 -0.04404
H   4.65447 -0.53778 -1.72142
H   4.06647 0.77610 -0.65477
C   1.09230 -2.74183 2.53470
H   1.72912 -3.55712 2.92654
H   1.01634 -1.97073 3.31838
H   0.08481 -3.15773 2.37405
C   0.37735 -3.62347 -0.50912
H   0.86188 -4.61217 -0.62623
H -0.43610 -3.73254 0.22473
H -0.08338 -3.36421 -1.47623
C   2.39667 -2.15842 -2.46768
H   1.38962 -2.07523 -2.91025
H   3.04616 -1.42548 -2.96927
H   2.77179 -3.17166 -2.70592
C   3.24304 4.13062 -1.37334
H   4.12268 3.47483 -1.47399
H   3.03704 4.58186 -2.36038
H   3.50872 4.95644 -0.68882

E_{gem}
SCF Energy = -1305.85096405
Enthalpy 0K = -1305.350457
Energy 298K = -1305.319056
Free Energy 298K = -1305.411594
Lowest Frequency = 21.6854 cm^{-1}
SCF (DCE) Energy = -1305.86024824
SCF (BP86-D3) Energy = -1305.94525093
SCF (BS2) Energy = -1305.80550339

C   5.23784 -1.09563 0.13545
C   4.42994 -0.81312 1.25027
C   3.15977 -0.24610 1.07173
C   2.66900 0.04849 -0.21885
C   3.49396 -0.22715 -1.32763
C   4.76633 -0.79778 -1.15219
C   1.30296 0.70961 -0.38953
C   0.50384 0.27542 -1.59029
Rh -0.54905 -0.68285 -0.08651
N -1.66646 1.06316 -0.03048
N -3.01258 0.94226 -0.09614
C -3.53417 2.19832 -0.14136
C -2.50163 3.14568 -0.10533
C -1.30127 2.39682 -0.03546
C   0.06294 2.94355 0.06865
C   0.17344 4.33319 0.34939
C   1.40073 4.99200 0.43884
C   2.58992 4.26948 0.26541
C   2.51676 2.89290 0.02156
C   1.28425 2.21576 -0.08846
C -0.74960 -2.71575 -0.93871
C   0.09784 -2.80446 0.22494
C -0.70237 -2.44569 1.40432
C -2.00824 -2.09676 0.96622
C -2.04052 -2.21323 -0.50544
C -0.38651 -3.13180 -2.33719
C   1.49513 -3.35666 0.26335
C -0.21154 -2.50550 2.82496
C -3.18360 -1.67683 1.79631
C -3.26959 -2.01371 -1.34041
H -0.74364 4.90276 0.51859
H   1.42570 6.06568 0.65366
H   3.44302 2.32592 -0.10124
H   3.56484 4.76329 0.32936
H -2.60525 4.22838 -0.15404
C -5.02179 2.40209 -0.21221
H   0.99516 -0.43335 -2.26941
H   0.71248 0.32757 0.63588
H -0.05072 1.06479 -2.11159
H   2.53556 -0.01982 1.94479
H   3.14563 0.02345 -2.33473
H   4.79023 -1.03395 2.26072
H   5.39221 -1.00260 -2.02736
H   6.22933 -1.53984 0.27114
H -4.02248 -2.79509 -1.12309
H -3.70526 -1.02474 -1.10826
H -3.03904 -2.05317 -2.41678
H -3.97206 -2.45236 1.77606
H -2.90231 -1.50639 2.84750
H -3.60498 -0.74176 1.38322
H -0.62576 -4.19980 -2.49826
H -0.94136 -2.54791 -3.08823
H   0.68971 -3.00205 -2.53566
H   1.47990 -4.44124 0.48386
H   2.01356 -3.21813 -0.69837
H   2.10768 -2.86731 1.03710
H   0.84302 -2.19471 2.90389
H -0.80595 -1.85572 3.48590

H -0.27789 -3.53714 3.21977
H -5.52894 1.43181 -0.33756
H -5.41908 2.87600 0.70399
H -5.30568 3.04870 -1.06136

TS(E-G)$_{gem}$
SCF Energy = -1305.84949772
Enthalpy 0K = -1305.351397
Energy 298K = -1305.320307
Free Energy 298K = -1305.411748
Lowest Frequency = -546.3654 cm^{-1}
SCF (DCE) Energy = -1305.85851038
SCF (BP86-D3) Energy = -1305.94547184
SCF (BS2) Energy = -1305.80366025

Rh -0.43224 -0.68982 -0.06326
C -0.42467 -2.79076 -0.89872
C   0.49281 -2.72987 0.22103
C -0.28021 -2.42517 1.42197
C -1.64656 -2.24999 1.03697
C -1.74246 -2.46511 -0.41655
C -0.31681 2.91673 0.04849
C -0.35762 4.30168 0.36400
C   0.97252 2.32380 -0.13541
C   0.79173 5.08759 0.46237
H -1.33176 4.75981 0.55394
C   2.12489 3.13265 -0.02092
C   2.04986 4.50183 0.25976
H   0.70354 6.15173 0.70591
H   3.10652 2.67689 -0.17246
H   2.96628 5.09666 0.32825
C -2.89188 2.81612 -0.14739
C -3.79508 1.74375 -0.18237
H -3.12798 3.87735 -0.20964
N -1.80143 0.85902 -0.04749
N -3.11781 0.56344 -0.11950
C -1.60830 2.22359 -0.05984
C   1.16716 0.85422 -0.50954
C   0.42547 0.34807 -1.67387
H   0.93856 -0.35596 -2.33904
H   0.51198 0.38636 0.73022
H -0.26445 1.03424 -2.17663
C   2.57640 0.32444 -0.28753
C   3.09565 0.18841 1.01982
C   3.42211 0.02617 -1.37508
C   4.41047 -0.24701 1.23395
H   2.45496 0.43451 1.87488
C   4.73970 -0.41634 -1.16294
H   3.05516 0.16301 -2.39721
C   5.23854 -0.55689 0.14032
H   4.79094 -0.34412 2.25642
H   5.37927 -0.64009 -2.02345
H   6.26566 -0.89834 0.30509
C -3.02739 -2.45975 -1.18985
H -3.67393 -3.30301 -0.88162
H -3.56273 -1.51219 -0.99510
H -2.84932 -2.54811 -2.27336
C -2.82337 -1.95857 1.92055
H -3.47454 -2.84858 2.00824
H -2.50907 -1.66747 2.93524
H -3.41579 -1.13498 1.48325
C -0.06788 -3.19489 -2.30283
H -0.12629 -4.29368 -2.41701
H -0.75213 -2.74586 -3.03961
H   0.95927 -2.89391 -2.56618
C   1.94428 -3.11807 0.19141
H   2.06308 -4.19919 0.39680
H   2.40163 -2.91312 -0.78955
H   2.52942 -2.56757 0.94464
C   0.27211 -2.38663 2.82128
H   1.29763 -1.98401 2.84106
H -0.34887 -1.76418 3.48414

H   0.30931 -3.40422 3.25426
C -5.29583 1.75420 -0.26783
H -5.67363 0.72380 -0.36687
H -5.75877 2.19994 0.63144
H -5.65218 2.33400 -1.13777

G$_{gem}$
SCF Energy = -1305.86178927
Enthalpy 0K = -1305.361985
Energy 298K = -1305.330583
Free Energy 298K = -1305.422556
Lowest Frequency = 21.0994 cm^{-1}
SCF (DCE) Energy = -1305.87045320
SCF (BP86-D3) Energy = -1305.96027327
SCF (BS2) Energy = -1305.81451060

C   5.19346 -0.01519 0.09445
C   4.33520 0.28150 1.17015
C   2.98980 0.59376 0.93547
C   2.46250 0.61499 -0.37821
C   3.34011 0.33271 -1.44730
C   4.68987 0.01423 -1.21377
C   1.02682 1.00582 -0.63358
C   0.32224 0.38591 -1.71176
Rh -0.32722 -0.64623 0.06252
N -1.92283 0.63326 -0.05785
N -3.18105 0.15135 -0.12423
C -4.00891 1.22570 -0.25658
C -3.25825 2.41330 -0.25894
C -1.91040 2.00396 -0.12257
C -0.71144 2.84070 0.00943
C -0.92292 4.19746 0.36672
C   0.12406 5.10924 0.51758
C   1.44324 4.67635 0.32284
C   1.68120 3.33783 -0.00966
C   0.63811 2.39377 -0.17394
C -0.14351 -2.79142 -0.92618
C   0.89175 -2.58955 0.08474
C   0.23756 -2.38073 1.35269
C -1.19981 -2.38221 1.11827
C -1.41917 -2.69225 -0.29449
C   0.11772 -3.15114 -2.36223
C   2.36339 -2.80023 -0.12635
C   0.92358 -2.30987 2.69090
C -2.29676 -2.28261 2.14117
C -2.76757 -2.87242 -0.92146
H -1.94914 4.52689 0.55223
H -0.09063 6.14658 0.79551
H   2.71231 3.01296 -0.16912
H   2.28450 5.36871 0.43103
H -3.63027 3.43068 -0.37213
C -5.49480 1.02918 -0.37186
H   0.84093 -0.34621 -2.34010
H -0.06586 0.22378 1.33199
H -0.51490 0.90663 -2.18670
H   2.32390 0.82858 1.77263
H   2.96864 0.39083 -2.47597
H   4.71889 0.27237 2.19626
H   5.35034 -0.19601 -2.06207
H   6.24552 -0.25718 0.27740
H -3.31261 -3.70263 -0.43616
H -3.35333 -1.94159 -0.78566
H -2.69179 -3.09735 -1.99685
H -2.71839 -3.28047 2.36700
H -1.93123 -1.84928 3.08543
H -3.10738 -1.64050 1.75799
H   0.28223 -4.24024 -2.46657
H -0.72751 -2.87669 -3.01222
H   1.02140 -2.65284 -2.75101
H   2.61684 -3.87344 -0.02937
H   2.68532 -2.47280 -1.12726
H   2.96501 -2.24250 0.60748

```
H   1.89463 -1.79528  2.61939
H   0.30923 -1.77668  3.43268
H   1.11090 -3.32892  3.07863
H  -5.73746 -0.04404 -0.31336
H  -6.04234  1.54659  0.43629
H  -5.88797  1.41529 -1.32960
```

D2$_{1,2}$

SCF (BP86) Energy = -1305.85842185
Enthalpy 0K = -1305.357995
Energy 298K = -1305.325641
Free Energy 298K = -1305.421286
Lowest Frequency = 11.2808 cm^{-1}
SCF (DCE) Energy = -1305.86953197
SCF (BP86-D3) Energy = -1305.95907635
SCF (BS2) Energy = -1305.81321007

```
Rh  0.58883 -0.48313 -0.16676
C   1.01828 -2.59418  0.59989
C   1.87961 -2.36275 -0.56246
C   2.75463 -1.26960 -0.27128
C   2.43105 -0.80204  1.07829
C   1.41141 -1.66542  1.63054
C  -0.72692  1.83061  1.15788
C  -1.59682  2.53176  2.02014
C  -0.84450  0.41380  1.02570
C  -2.58024  1.84265  2.74495
H  -1.49754  3.61902  2.11824
C  -1.84433 -0.25589  1.74441
C  -2.70870  0.45132  2.60586
H  -3.25270  2.39313  3.41177
H  -1.98167 -1.33696  1.62256
H  -3.48730 -0.08905  3.15616
C   0.87293  3.68614  0.05928
C   1.91158  3.42028 -0.86411
H   0.56671  4.65257  0.45865
N   1.02772  1.50660 -0.38960
N   1.99289  2.08159 -1.13147
C   0.32258  2.42287  0.34530
C  -1.09344 -1.05574 -1.60438
C  -0.04215 -0.32658 -2.21645
H  -0.18061  0.72052 -2.49960
H   0.71892 -0.85471 -2.80112
C   0.91884 -1.65337  3.04819
H   1.50096 -2.37117  3.65640
H   1.02517 -0.65764  3.50439
H  -0.14346 -1.93365  3.11607
C   3.15922  0.30179  1.79039
H   2.56223  0.71204  2.61921
H   4.11324 -0.07031  2.20897
H   3.38713  1.12516  1.09483
C   0.06910 -3.74890  0.76507
H   0.59931 -4.64550  1.13946
H  -0.73129 -3.51464  1.48512
H  -0.40573 -4.02786 -0.18985
C   1.87646 -3.20702 -1.80647
H   2.30194 -4.20583 -1.59809
H   0.85660 -3.36816 -2.19782
H   2.47643 -2.75092 -2.60898
C   3.82762 -0.67711 -1.14025
H   3.87895 -1.17878 -2.11959
H   3.62303  0.39689 -1.31398
H   4.81872 -0.77528 -0.66090
H  -1.01743 -2.15211 -1.62550
C  -2.48071 -0.58096 -1.41576
C  -2.85317  0.78060 -1.51718
C  -3.49613 -1.53471 -1.17606
C  -4.19211  1.16504 -1.38796
H  -2.08491  1.54231 -1.68142
C  -4.83857 -1.14991 -1.04835
H  -3.22424 -2.59485 -1.10170
C  -5.19229  0.20381 -1.15425
H  -4.45867  2.22437 -1.46487
H  -5.60788 -1.90866 -0.86885
H  -6.23871  0.51057 -1.05477
C   2.85623  4.37897 -1.53506
H   2.31950  5.11522 -2.16047
H   3.45398  4.95097 -0.80247
H   3.55103  3.82400 -2.18542
```

TS(D2-E2)$_{1,2}$

SCF Energy = -1305.83009388
Enthalpy 0K = -1305.330108
Energy 298K = -1305.298618
Free Energy 298K = -1305.391419
Lowest Frequency = -268.4202 cm^{-1}
SCF (DCE) Energy = -1305.83958349
SCF (BP86-D3) Energy = -1305.92814087
SCF (BS2) Energy = -1305.78477380

```
Rh  1.02067 -0.11989 -0.34964
C   2.77007 -1.53784 -0.33870
C   2.99981 -0.49436 -1.31613
C   2.99948  0.77472 -0.62526
C   2.84663  0.49255  0.81551
C   2.72391 -0.91836  0.98745
C  -1.36372 -0.10520  1.44600
C  -2.07216 -0.53353  2.58721
C  -0.70669 -1.07773  0.60332
C  -2.10466 -1.88907  2.94188
H  -2.56720  0.21227  3.21896
C  -0.74117 -2.43768  1.00547
C  -1.41751 -2.84265  2.16424
H  -2.65613 -2.20485  3.83390
H  -0.25969 -3.19388  0.37248
H  -1.43107 -3.90030  2.44940
C  -1.68001  2.54498  1.42763
C  -0.90158  3.45934  0.68871
H  -2.51321  2.76272  2.09507
N  -0.12887  1.49275  0.19450
N   0.04541  2.80028 -0.05600
C  -1.15382  1.28097  1.08736
C  -1.07289 -1.02310 -1.28898
C  -0.16128 -0.16552 -2.06334
H  -0.52060  0.82702 -2.35880
H   0.39798 -0.67550 -2.85988
C   2.58655 -1.66809  2.28221
H   3.51118 -2.22874  2.51463
H   2.38901 -0.98491  3.12266
H   1.75460 -2.39238  2.24322
C   2.85114  1.55662  1.87520
H   2.47071  1.17825  2.83665
H   3.87623  1.93787  2.04083
H   2.21981  2.40800  1.56879
C   2.80372 -3.01935 -0.59969
H   3.83746 -3.41019 -0.53107
H   2.19700 -3.57143  0.13595
H   2.42708 -3.26708 -1.60558
C   3.25071 -0.70523 -2.78371
H   4.32792 -0.86845 -2.97442
H   2.71330 -1.58794 -3.16831
H   2.93559  0.16658 -3.37866
C   3.23188  2.14233 -1.20297
H   3.20216  2.12231 -2.30401
H   2.44733  2.84000 -0.85850
H   4.21865  2.54031 -0.89891
H  -0.89563 -2.09136 -1.46896
C  -2.55006 -0.72239 -1.26597
C  -3.06641  0.57874 -1.44721
C  -3.45594 -1.79370 -1.10439
C  -4.45276  0.79501 -1.46811
H  -2.38649  1.42677 -1.56634
C  -4.83978 -1.57794 -1.13682
H  -3.06469 -2.80586 -0.94874
```

C -5.34413 -0.27882 -1.31694
H -4.83644 1.81134 -1.60692
H -5.52525 -2.42435 -1.02125
H -6.42526 -0.10579 -1.34075
C -0.99990 4.95807 0.61832
H -1.94258 5.29010 0.14613
H -0.95986 5.41908 1.62124
H -0.16457 5.35777 0.02165

E2$_{1,2}$
SCF Energy = -1305.84431795
Enthalpy 0K = -1305.342406
Enthalpy 298K = -1305.310506
Free Energy 298K = -1305.406138
Lowest Frequency = 12.5970 cm^{-1}
SCF (DCE) Energy = -1305.85626894
SCF (BP86-D3) Energy = -1305.93178241
SCF (BS2) Energy = -1305.79922884

Rh 1.19087 -0.38325 0.02545
C   2.44569 -2.04741 0.80178
C   2.32471 -2.12705 -0.62056
C   2.85412 -0.85840 -1.15586
C   3.48158 -0.11082 -0.04276
C   3.18958 -0.81118 1.14723
C -1.41748 1.54097 1.18432
C -1.74405 2.45018 2.22573
C -1.85802 0.18929 1.29949
C -2.46225 2.06182 3.35957
H -1.38886 3.48142 2.12976
C -2.55817 -0.18341 2.47164
C -2.86157 0.72225 3.49325
H -2.69082 2.79390 4.14145
H -2.90273 -1.22127 2.55789
H -3.41034 0.38737 4.37984
C -0.68112 3.35165 -0.54343
C   0.46849 3.43035 -1.36742
H -1.46866 4.09453 -0.41678
N   0.57364 1.51052 -0.33850
N   1.21927 2.30847 -1.23601
C -0.59676 2.09589 0.08247
C -1.76299 -0.91729 0.22981
C -0.63481 -0.85861 -0.83118
H -0.86416 -0.10178 -1.59863
H -0.58191 -1.84340 -1.32965
C   3.53547 -0.40879 2.55205
H   4.32160 -1.06623 2.96881
H   3.90105 0.62823 2.59738
H   2.66020 -0.48537 3.21980
C   4.17154 1.20876 -0.21374
H   4.48300 1.63382 0.75321
H   5.07211 1.10347 -0.84597
H   3.48241 1.92318 -0.70584
C   2.03210 -3.09000 1.80199
H   2.89723 -3.71044 2.10544
H   1.62239 -2.63035 2.71672
H   1.26544 -3.76492 1.39026
C   1.81875 -3.28372 -1.43273
H   2.65592 -3.93371 -1.74920
H   1.11210 -3.90502 -0.85944
H   1.30034 -2.94300 -2.34369
C   2.95887 -0.45541 -2.59539
H   2.28182 -1.04549 -3.23215
H   2.69503 0.61147 -2.69882
H   3.99240 -0.59925 -2.96584
H -1.61928 -1.85615 0.79851
C -3.10771 -1.06429 -0.50163
C -3.74703 -2.31745 -0.57715
C -3.70660 0.03785 -1.14837
C -4.95613 -2.47050 -1.27566
H -3.29230 -3.18270 -0.07863
C -4.91345 -0.11217 -1.84580
H -3.22151 1.01928 -1.09759
C -5.54400 -1.36698 -1.91258
H -5.43956 -3.45283 -1.31837
H -5.36627 0.75535 -2.33834
H -6.48765 -1.48187 -2.45639
C   0.90281 4.53342 -2.29107
H   0.13659 4.75227 -3.05627
H   1.09474 5.47556 -1.74601
H   1.83095 4.24366 -2.80909

TS(E2-F2)$_{1,2}$
SCF Energy = -1305.77184663
Enthalpy 0K = -1305.272700
Energy 298K = -1305.240969
Free Energy 298K = -1305.334874
Lowest Frequency = -523.3440 cm^{-1}
SCF (DCE) Energy = -1305.78037585
SCF (BP86-D3) Energy = -1305.86548126
SCF (BS2) Energy = -1305.72422846

Rh -0.99820 -0.04754 0.25013
C -2.68578 -0.68658 1.58950
C -2.71602 0.74027 1.38261
C -3.12817 1.00717 -0.02137
C -3.23737 -0.22666 -0.68178
C -2.84867 -1.28296 0.27975
C   0.59391 -0.43351 -1.31863
C   0.22727 -1.02184 -2.57565
C   0.82273 -1.30454 -0.15693
C   0.05965 -2.39099 -2.70480
H   0.08552 -0.35559 -3.43361
C   0.65519 -2.71884 -0.36052
C   0.27295 -3.24856 -1.58212
H -0.22072 -2.82012 -3.67241
H   0.87502 -3.38762 0.48085
H   0.16249 -4.33192 -1.69750
C   1.34411 2.10020 -1.93882
C   1.13592 3.21219 -1.06406
H   1.73059 2.11794 -2.95696
N   0.48753 1.47902 0.02430
N   0.61712 2.82609 0.13005
C   0.93365 0.99921 -1.18950
C -2.62478 1.80194 2.44564
H -2.05970 1.44893 3.32439
H -2.12353 2.70664 2.06509
H -3.63093 2.09899 2.80033
C -3.34862 2.38535 -0.57802
H -4.23502 2.86739 -0.12341
H -2.48175 3.03896 -0.37703
H -3.50332 2.36140 -1.66861
C -3.59353 -0.49170 -2.11540
H -4.54160 -1.05633 -2.19937
H -3.71138 0.44466 -2.68409
H -2.81303 -1.09424 -2.61714
C -2.93521 -2.75262 -0.02233
H -3.99018 -3.07219 -0.12492
H -2.41789 -3.00203 -0.96456
H -2.47676 -3.35778 0.77583
C -2.55570 -1.40461 2.90506
H -3.54035 -1.53911 3.39461
H -2.11385 -2.40674 2.77796
H -1.91782 -0.84892 3.61430
C   1.64068 -0.81636 1.04371
H   1.48532 -1.57521 1.83901
C   0.96847 0.46092 1.56600
H   1.64307 1.29168 1.81204
H   0.25691 0.30284 2.38913
C   3.15439 -0.70045 0.82668
C   3.96135 -0.20377 1.87305
C   3.78029 -1.10325 -0.36846
C   5.35185 -0.09940 1.72573
H   3.49530 0.09880 2.81892

C   5.17338 -1.00264 -0.51751
H   3.17473 -1.49756 -1.19035
C   5.96420 -0.49851 0.52608
H   5.95843 0.29046 2.55034
H   5.64036 -1.31808 -1.45685
H   7.04994 -0.41860 0.40790
C   1.44105 4.66167 -1.31510
H   2.51792 4.82378 -1.50148
H   0.89671 5.04496 -2.19662
H   1.14880 5.26107 -0.43907

F2$_{1,2}$
SCF (BP86) Energy = -1305.84180527
Enthalpy 0K = -1305.339379
Energy 298K = -1305.307701
Free Energy 298K = -1305.402431
Lowest Frequency = 10.5332 cm^{-1}
SCF (DCE) Energy = -1305.84981183
SCF (BP86-D3) Energy = -1305.93594099
SCF (BS2) Energy = -1305.79468230

Rh 0.96152 -0.24924 -0.11188
C   2.04923 -1.42028 -1.69301
C   2.72067 -0.16684 -1.44457
C   3.25521 -0.16914 -0.07337
C   2.83639 -1.37789 0.55073
C   2.03441 -2.13075 -0.42524
C -0.55332 0.17020 1.39787
C -0.48343 -0.38536 2.72615
C -1.16639 -0.59730 0.28950
C -0.89869 -1.68109 2.95638
H -0.09572 0.23616 3.54066
C -1.56270 -1.95056 0.61625
C -1.41532 -2.48124 1.88237
H -0.84239 -2.10223 3.96570
H -2.04133 -2.54601 -0.17102
H -1.73500 -3.50926 2.08354
C   0.63523 2.56841 1.59066
C   0.95134 3.41785 0.50025
H   0.90540 2.70178 2.63642
N -0.10986 1.73272 -0.39654
N   0.49517 2.96223 -0.68742
C -0.03523 1.47113 1.01977
C   2.94345 0.95021 -2.42636
H   2.44027 0.75053 -3.38553
H   2.54718 1.90528 -2.03363
H   4.02129 1.08810 -2.63222
C   4.08953 0.93300 0.51562
H   5.10562 0.95213 0.07720
H   3.63228 1.92075 0.33244
H   4.19955 0.81664 1.60583
C   3.13458 -1.84761 1.94659
H   3.84335 -2.69752 1.94370
H   3.58008 -1.04476 2.55588
H   2.21622 -2.18408 2.45953
C   1.50516 -3.51759 -0.19103
H   2.32624 -4.25953 -0.21522
H   1.00635 -3.59758 0.78943
H   0.77138 -3.80465 -0.96088
C   1.52831 -1.92639 -3.01044
H   2.29082 -2.52391 -3.54796
H   0.64388 -2.57092 -2.87501
H   1.23291 -1.09755 -3.67455
C -1.94224 0.09892 -0.85355
H -1.73564 -0.48999 -1.76605
C -1.41538 1.53955 -1.07939
H -2.12099 2.29114 -0.68081
H -1.25077 1.76089 -2.14462
C -3.45969 0.09834 -0.64958
C -4.31219 -0.41607 -1.64573
C -4.03888 0.60894 0.53231
C -5.70643 -0.41862 -1.47413

H -3.87626 -0.81846 -2.56854
C -5.42983 0.60699 0.70768
H -3.39161 1.00257 1.32437
C -6.26992 0.09408 -0.29595
H -6.35112 -0.82256 -2.26228
H -5.86078 1.00484 1.63285
H -7.35621 0.09227 -0.15760
C   1.70826 4.71712 0.54389
H   1.32729 5.36771 1.34925
H   2.78402 4.55618 0.73592
H   1.60556 5.24522 -0.41648

TS(E2-E_{gem})
SCF Energy = -1305.84054772
Enthalpy 0K = -1305.338937
Energy 298K = -1305.308139
Free Energy 298K = -1305.399028
Lowest Frequency = -27.8982 cm^{-1}
SCF (DCE) Energy = -1305.85151074
SCF (BP86-D3) Energy = -1305.92899208
SCF (BS2) Energy = -1305.79485407

Rh 0.97415 -0.53153 0.10534
C   1.28544 -2.61809 0.81417
C   1.54453 -2.50954 -0.58877
C   2.63085 -1.52535 -0.74089
C   3.14730 -1.19419 0.60503
C   2.29657 -1.81571 1.54354
C -1.04676 2.52728 0.41521
C -1.42208 3.79224 0.95273
C -1.98261 1.45368 0.54187
C -2.64654 4.02123 1.58041
H -0.69470 4.60645 0.89265
C -3.20434 1.70485 1.21120
C -3.55198 2.95947 1.71983
H -2.88044 5.01560 1.97550
H -3.91374 0.87816 1.31689
H -4.51507 3.10096 2.22139
C   0.96567 3.61748 -0.76075
C   2.26977 3.16860 -1.05701
H   0.55000 4.60797 -0.94147
N   1.21198 1.46615 -0.17554
N   2.40701 1.86933 -0.70198
C   0.29864 2.50683 -0.20705
C -1.75921 -0.00054 0.08812
C -0.71255 -0.25153 -1.03286
H -0.64124 0.59149 -1.73782
H -0.95824 -1.17173 -1.58697
C   2.36691 -1.71699 3.04086
H   2.74336 -2.66002 3.48036
H   3.03515 -0.90354 3.36173
H   1.37318 -1.52816 3.48143
C   4.29974 -0.26896 0.85129
H   4.45570 -0.08784 1.92634
H   5.23566 -0.68703 0.43716
H   4.09882 0.69892 0.35219
C   0.27639 -3.51521 1.47538
H   0.73772 -4.47792 1.76871
H -0.13148 -3.05844 2.39248
H -0.56768 -3.74077 0.80467
C   0.89538 -3.28560 -1.69851
H   1.47884 -4.19615 -1.93089
H -0.12544 -3.60261 -1.43107
H   0.82779 -2.68799 -2.62193
C   3.28452 -1.08809 -2.01570
H   2.66889 -1.33518 -2.89445
H   3.43671 0.00548 -1.98597
H   4.26954 -1.57913 -2.13541
H -1.41427 -0.54490 1.00563
C -3.05805 -0.70742 -0.32260
C -3.49898 -1.86785 0.34204
C -3.80500 -0.23827 -1.42602

```
C  -4.65233 -2.55057 -0.08319
H  -2.93608 -2.23560  1.20872
C  -4.95606 -0.91544 -1.85011
H  -3.48016  0.67054 -1.94419
C  -5.38388 -2.07723 -1.18218
H  -4.98031 -3.44925  0.45074
H  -5.52470 -0.53466 -2.70553
H  -6.28378 -2.60485 -1.51519
C   3.41777  3.91788 -1.67226
H   3.14085  4.36065 -2.64556
H   3.76199  4.74574 -1.02592
H   4.26852  3.23677 -1.83437
```

E_{gem}′

SCF Energy = -1305.85096406
Enthalpy 0K = -1305.350457
Energy 298K = -1305.319056
Free Energy 298K = -1305.411594
Lowest Frequency = 21.6855 cm^{-1}
SCF (DCE) Energy = -1305.86024828
SCF (BP86-D3) Energy = -1305.94525098
SCF (BS2) Energy = -1305.80550338

```
C  -5.23784 -1.09562  0.13545
C  -4.42994 -0.81311  1.25027
C  -3.15977 -0.24610  1.07173
C  -2.66900  0.04849 -0.21885
C  -3.49396 -0.22714 -1.32763
C  -4.76633 -0.79778 -1.15219
C  -1.30296  0.70961 -0.38953
C  -0.50385  0.27542 -1.59029
Rh  0.54905 -0.68285 -0.08651
N   1.66646  1.06315 -0.03048
N   3.01258  0.94226 -0.09615
C   3.53418  2.19831 -0.14136
C   2.50164  3.14568 -0.10534
C   1.30127  2.39682 -0.03547
C  -0.06293  2.94355  0.06865
C  -0.17343  4.33319  0.34939
C  -1.40072  4.99200  0.43884
C  -2.58991  4.26948  0.26541
C  -2.51675  2.89291  0.02156
C  -1.28424  2.21577 -0.08846
C  -0.09784 -2.80446  0.22494
C   0.74959 -2.71576 -0.93871
C   2.04051 -2.21323 -0.50544
C   2.00824 -2.09676  0.96622
C   0.70236 -2.44569  1.40432
C  -1.49513 -3.35665  0.26336
C   0.38650 -3.13181 -2.33719
C   3.26959 -2.01372 -1.34041
C   3.18360 -1.67683  1.79631
C   0.21154 -2.50549  2.82497
H   0.74365  4.90276  0.51859
H  -1.42569  6.06568  0.65366
H  -3.44302  2.32592 -0.10124
H  -3.56483  4.76330  0.32937
H   2.60526  4.22838 -0.15405
C   5.02180  2.40209 -0.21220
H   0.05072  1.06479 -2.11160
H  -0.99516 -0.43335 -2.26941
H   0.27789 -3.53714  3.21977
H   0.80595 -1.85571  3.48590
H  -0.84302 -2.19470  2.90389
H   2.90231 -1.50639  2.84750
H   3.97205 -2.45236  1.77606
H   3.60498 -0.74177  1.38322
H  -1.47991 -4.44124  0.48386
H  -2.10768 -2.86731  1.03711
H  -2.01357 -3.21812 -0.69836
H   0.62575 -4.19980 -2.49826
H  -0.68972 -3.00205 -2.53566
```

```
H   0.94135 -2.54791 -3.08823
H   3.03903 -2.05318 -2.41678
H   3.70526 -1.02475 -1.10826
H   4.02248 -2.79510 -1.12309
H  -0.71248  0.32757  0.63588
H  -2.53556 -0.01981  1.94479
H  -3.14563  0.02346 -2.33473
H  -4.79023 -1.03394  2.26073
H  -5.39221 -1.00259 -2.02736
H  -6.22933 -1.53983  0.27114
H   5.52894  1.43180 -0.33761
H   5.30569  3.04875 -1.06131
H   5.41909  2.87593  0.70403
```

TS(E-G)$_{gem}$′

SCF Energy = -1305.84949774
Enthalpy 0K = -1305.351398
Energy 298K = -1305.320307
Free Energy 298K = -1305.411751
Lowest Frequency = -546.2835 cm^{-1}
SCF (DCE) Energy = -1305.85851033
SCF (BP86-D3) Energy = -1305.94547037
SCF (BS2) Energy = -1305.80366040

```
C  -1.16718  0.85420 -0.50953
C  -0.42545  0.34807 -1.67386
Rh  0.43225 -0.68982 -0.06325
N   1.80140  0.85907 -0.04750
N   3.11778  0.56355 -0.11959
C   3.79500  1.74388 -0.18248
C   2.89177  2.81621 -0.14744
C   1.60822  2.22364 -0.05983
C   0.31671  2.91674  0.04855
C   0.35748  4.30167  0.36412
C  -0.79189  5.08754  0.46252
C  -2.05001  4.50175  0.25990
C  -2.12500  3.13258 -0.02083
C  -0.97261  2.32378 -0.13536
C  -0.49270 -2.72988  0.22119
C   0.42467 -2.79075 -0.89865
C   1.74250 -2.46505 -0.41661
C   1.64673 -2.24993  1.03692
C   0.28043 -2.42514  1.42205
C  -1.94413 -3.11823  0.19174
C   0.06776 -3.19493 -2.30271
C   3.02736 -2.45966 -1.19003
C   2.82361 -1.95848  1.92040
C  -0.27176 -2.38659  2.82142
H   1.33161  4.75982  0.55407
H  -0.70373  6.15168  0.70611
H  -3.10661  2.67680 -0.17238
H  -2.96645  5.09656  0.32842
H   3.12783  3.87745 -0.20969
C   5.29575  1.75438 -0.26806
H   0.26447  1.03428 -2.17657
H  -0.93851 -0.35593 -2.33907
H  -0.30891 -3.40417  3.25441
H   0.34928 -1.76412  3.48421
H  -1.29728 -1.98396  2.84128
H   2.50939 -1.66733  2.93510
H   3.47477 -2.84850  2.00808
H   3.41601 -1.13491  1.48301
H  -2.06278 -4.19937  0.39713
H  -2.52924 -2.56783  0.94507
H  -2.40163 -2.91332 -0.78915
H   0.12619 -4.29371 -2.41688
H  -0.95943 -2.89398 -2.56597
H   0.75192 -2.74588 -3.03957
H   2.84919 -2.54799 -2.27352
H   3.56271 -1.51211 -0.99531
H   3.67393 -3.30292 -0.88188
H  -0.51204  0.38634  0.73020
```

H 5.67359 0.72394 -0.36661
H 5.65201 2.33375 -1.13831
H 5.75873 2.20060 0.63096
C -2.57642 0.32438 -0.28760
C -3.42210 0.02612 -1.37518
C -3.09572 0.18830 1.01974
C -4.73969 -0.41642 -1.16309
H -3.05512 0.16300 -2.39729
C -4.41052 -0.24716 1.23382
H -2.45505 0.43437 1.87482
C -5.23856 -0.55701 0.14015
H -5.37923 -0.64016 -2.02362
H -4.79102 -0.34432 2.25627
H -6.26569 -0.89849 0.30489
H -0.28214 -4.24023 -2.46660
H -1.02144 -2.65288 -2.75100
H 0.72750 -2.87659 -3.01224
H 2.69179 -3.09734 -1.99687
H 3.35334 -1.94158 -0.78567
H 3.31262 -3.70262 -0.43618
H 0.06585 0.22379 1.33199
H -2.32390 0.82858 1.77263
H -2.96864 0.39083 -2.47596
H -4.71889 0.27235 2.19626
H -5.35034 -0.19602 -2.06206
H -6.24552 -0.25719 0.27741
H 5.73746 -0.04403 -0.31337
H 5.88797 1.41532 -1.32958
H 6.04233 1.54658 0.43632

G_{gem}'
SCF Energy = -1305.86178925
Enthalpy 0K = -1305.361985
Energy 298K = -1305.330583
Free Energy 298K = -1305.422556
Lowest Frequency = 21.0995 cm^{-1}
SCF (DCE) Energy = -1305.87045315
SCF (BP86-D3) Energy = -1305.96027327
SCF (BS2) Energy = -1305.81451074

C -5.19346 -0.01520 0.09446
C -4.33520 0.28149 1.17015
C -2.98980 0.59375 0.93548
C -2.46250 0.61498 -0.37820
C -3.34011 0.33270 -1.44729
C -4.68987 0.01422 -1.21376
C -1.02682 1.00582 -0.63358
C -0.32224 0.38591 -1.71176
Rh 0.32722 -0.64623 0.06252
N 1.92282 0.63327 -0.05785
N 3.18105 0.15136 -0.12422
C 4.00891 1.22571 -0.25657
C 3.25824 2.41330 -0.25894
C 1.91040 2.00397 -0.12257
C 0.71144 2.84070 0.00943
C 0.92291 4.19746 0.36671
C -0.12407 5.10924 0.51758
C -1.44325 4.67635 0.32284
C -1.68121 3.33782 -0.00966
C -0.63812 2.39377 -0.17394
C -0.89174 -2.58955 0.08473
C 0.14352 -2.79141 -0.92619
C 1.41918 -2.69225 -0.29450
C 1.19982 -2.38221 1.11827
C -0.23755 -2.38074 1.35268
C -2.36338 -2.80024 -0.12635
C -0.11771 -3.15112 -2.36224
C 2.76757 -2.87240 -0.92147
C 2.29676 -2.28262 2.14117
C -0.92357 -2.30989 2.69090
H 1.94913 4.52690 0.55222
H 0.09062 6.14658 0.79550
H -2.71232 3.01295 -0.16912
H -2.28451 5.36871 0.43103
H 3.63026 3.43069 -0.37213
C 5.49480 1.02919 -0.37185
H 0.51489 0.90663 -2.18670
H -0.84093 -0.34621 -2.34009
H -1.11088 -3.32894 3.07862
H -0.30922 -1.77669 3.43268
H -1.89462 -1.79530 2.61939
H 1.93124 -1.84929 3.08542
H 2.71840 -3.28047 2.36699
H 3.10738 -1.64050 1.75799
H -2.61683 -3.87345 -0.02935
H -2.96500 -2.24250 0.60747
H -2.68531 -2.47282 -1.12726

Organic Products

3aa$_{trans}$
SCF (BP86) Energy = -801.863440378
Enthalpy 0K = -801.614985
Energy 298K = -801.597633
Free Energy 298K = -801.662380
Lowest Frequency = 30.5220 cm^{-1}
SCF (DCE) Energy = -801.875377412
SCF (BP86-D3) Energy = -801.893122588
SCF (BS2) Energy = -802.076100779

```
C  -1.89225 -0.22796 -0.11356
C  -3.16420 -0.67487  0.36226
C  -3.22868 -2.03109  0.05849
N  -2.03842 -2.31155 -0.56648
N  -1.20969 -1.24607 -0.68994
C  -4.28516 -3.06646  0.30200
C  -1.34491  1.14052 -0.04278
C  -2.25616  2.21993 -0.10185
C  -1.82543  3.54974 -0.05348
C  -0.45262  3.83205  0.05631
C   0.46395  2.77976  0.12097
C   0.05454  1.42338  0.07189
C   1.04927  0.35298  0.20111
C   2.37081  0.45296 -0.09600
C   3.27267 -0.69364  0.13474
O   4.55265 -0.37761 -0.26070
O   2.96979 -1.78702  0.60759
H  -3.32245  1.99941 -0.21647
H  -2.55661  4.36316 -0.10482
H   1.52832  2.99956  0.25140
H  -0.10136  4.86793  0.10357
H  -3.91926 -0.09752  0.89265
H  -3.90454 -3.91017  0.90501
H  -5.12940 -2.62005  0.84824
H  -4.68051 -3.48295 -0.64204
H   0.69805 -0.62197  0.55546
H   2.81647  1.35250 -0.53267
H  -1.73057 -3.20751 -0.93660
C   5.50547 -1.44287 -0.07052
H   6.46778 -1.04637 -0.42365
H   5.56983 -1.72702  0.99295
H   5.21760 -2.33515 -0.65082
```

3aa$_{cis}$
SCF (BP86) Energy = -801.853150919
Enthalpy 0K = -801.604642
Energy 298K = -801.587372
Free Energy 298K = -801.652154
Lowest Frequency = 24.1879 cm^{-1}
SCF (DCE) Energy = -801.865395294
SCF (BP86-D3) Energy = -801.884935439
SCF (BS2) Energy = -802.065968895

```
C   0.77480  1.03792 -0.10483
C   0.97489  2.39165  0.25123
C  -0.51331  0.63138 -0.57655
C  -0.04620  3.33962  0.13159
H   1.94932  2.69324  0.64875
C  -1.52843  1.60990 -0.69506
C  -1.30631  2.94623 -0.34988
H   0.14002  4.38129  0.41325
H  -2.49889  1.30444 -1.09320
H  -2.11080  3.68030 -0.46278
C   3.30203  0.42495  0.01271
C   3.97304 -0.77893  0.19757
H   3.76282  1.39640 -0.15601
N   1.72433 -1.22243  0.23520
N   2.98220 -1.72234  0.32114
C   1.90786  0.10563  0.03930
C  -0.74655 -0.73796 -1.06860
C  -1.83862 -1.54598 -0.98313
C   5.43222 -1.11628  0.26430
H   5.72077 -1.85609 -0.50371
H   6.03473 -0.21082  0.09852
H   5.71547 -1.53030  1.24886
H   0.10002 -1.16126 -1.62390
C  -3.09433 -1.43100 -0.20690
H  -1.81281 -2.49094 -1.53559
O  -3.10258 -0.41993  0.71679
O  -4.03690 -2.20965 -0.34766
C  -4.30960 -0.35300  1.50517
H  -4.45373 -1.28304  2.07924
H  -4.16918  0.50129  2.18184
H  -5.19276 -0.20103  0.86309
H   3.09622 -2.71824  0.49279
```

4aa
SCF (BP86) Energy = -801.880630535
Enthalpy 0K = -801.630568
Energy 298K = -801.614246
Free Energy 298K = -801.675875
Lowest Frequency = 24.6483 cm^{-1}
SCF (DCE) Energy = -801.887964518
SCF (BP86-D3) Energy = -801.913289535
SCF (BS2) Energy = -802.089626261

```
C   0.19120 -0.32413  0.56885
C   0.07060  1.17535  0.28590
C  -1.26226  1.50041 -0.10353
C  -2.04062  0.26651 -0.07888
N  -1.19436 -0.73892  0.32157
C   1.04801  2.16765  0.37849
C   0.69516  3.49520  0.06109
C  -0.61565  3.81629 -0.33321
C  -1.60795  2.82398 -0.41621
C  -3.27639 -0.34735 -0.29751
C  -3.04841 -1.72579 -0.00633
N  -1.76326 -1.96115  0.37018
C  -4.02661 -2.86449 -0.06760
C   1.15455 -1.11422 -0.33886
C   2.60735 -0.97021  0.08120
O   3.01017 -0.38805  1.08213
O   3.42412 -1.60010 -0.80611
H  -2.62952  3.07878 -0.71515
H  -0.87013  4.85380 -0.57393
H   2.06254  1.91417  0.69977
H   1.45069  4.28468  0.12782
H  -4.20689  0.11622 -0.61928
H   0.87887 -2.18426 -0.29163
H   1.04176 -0.81070 -1.39388
H  -3.51966 -3.80482  0.19780
H  -4.86771 -2.71626  0.63260
H  -4.45798 -2.97572 -1.07801
H   0.46637 -0.50651  1.62416
C   4.83352 -1.54042 -0.47495
H   5.34525 -2.08446 -1.27998
H   5.17721 -0.49470 -0.43123
H   5.02334 -2.01729  0.49974
```

3ab$_{trans}$
SCF Energy = -805.033670789
Enthalpy 0K = -804.748130
Energy 298K = -804.730499
Free Energy 298K = -804.796501
Lowest Frequency = 18.1336 cm^{-1}
SCF (DCE) Energy = -805.043664977
SCF (BP86-D3) Energy = -805.066627239
SCF (BS2) Energy = -805.229567791

```
C   5.42276 -1.39377  0.04766
C   5.44769 -0.09408  0.57798
C   4.28055  0.68095  0.59279
```

```
C   3.05604  0.18149  0.08273
C   3.05274 -1.13131 -0.45577
C   4.21795 -1.90505 -0.46935
C   1.86560  1.03700  0.13160
C   0.59476  0.67292 -0.19625
C  -0.58286  1.55125 -0.15675
C  -1.91651  1.02259 -0.11815
C  -3.00489  1.92206 -0.04009
C  -2.82187  3.30863 -0.00533
C  -1.51927  3.83169 -0.06257
C  -0.42952  2.96135 -0.14311
C  -2.24800 -0.42209 -0.11343
C  -3.35021 -1.03204  0.56301
C  -3.28473 -2.38838  0.26013
N  -1.54040 -1.34863 -0.80210
H  -4.02015  1.51202 -0.03018
H  -3.68911  3.97513  0.04408
H   0.57945  3.37859 -0.22102
H  -1.35496  4.91442 -0.06228
H  -4.07770 -0.55259  1.21528
C  -4.14147 -3.55085  0.66316
H   2.05423  2.05624  0.49678
H   0.39344 -0.35833 -0.50021
H   4.30544  1.69506  1.00913
H   2.12835 -1.54173 -0.87479
H   6.37922  0.31809  0.98070
H   4.19079 -2.91532 -0.89232
H   6.33274 -2.00258  0.03187
H  -4.63665 -4.02072 -0.20588
H  -3.55837 -4.33501  1.17859
H  -4.92966 -3.21429  1.35304
N  -2.18758 -2.51647 -0.55355
H  -1.83378 -3.36754 -0.98274
```

3ab$_{cis}$

SCF Energy = -805.025866444
Enthalpy 0K = -804.740187
Energy 298K = -804.722731
Free Energy 298K = -804.788441
Lowest Frequency = 17.5158 cm^{-1}
SCF (DCE) Energy = -805.035206737
SCF (BP86-D3) Energy = -805.061654754
SCF (BS2) Energy = -805.222470479

```
C  -4.96518 -0.65292  1.34596
C  -5.07701 -1.32877  0.12027
C  -3.95127 -1.48638 -0.69956
C  -2.69016 -0.95730 -0.32915
C  -2.59234 -0.29406  0.91961
C  -3.71552 -0.14424  1.74235
C  -1.53925 -1.18681 -1.22308
C  -0.37319 -0.50066 -1.37412
C   0.00876  0.80923 -0.79825
C   1.30957  1.06790 -0.25998
C   1.59933  2.36858  0.21594
C   0.65744  3.40210  0.16884
C  -0.61445  3.15386 -0.37190
C  -0.92137  1.87529 -0.84971
C   2.37085  0.04411 -0.13381
C   3.43946  0.03497  0.81748
C   4.19178 -1.10114  0.53900
N   3.56068 -1.69091 -0.52752
N   2.45718 -1.02712 -0.95611
H   2.60044  2.57222  0.60967
H   0.92161  4.39908  0.53676
H  -1.90488  1.67828 -1.28704
H  -1.35901  3.95439 -0.43410
H   3.62353  0.74277  1.62346
C   5.42023 -1.67498  1.17885
H  -1.64664 -2.08082 -1.85394
H   0.38229 -0.95591 -2.02202
H  -4.04184 -2.02274 -1.65163
H  -1.62266  0.09046  1.24663
H  -6.04181 -1.74049 -0.19531
H  -3.61355  0.36629  2.70624
H  -5.84048 -0.53350  1.99312
H   6.26717 -1.73122  0.47130
H   5.73370 -1.04291  2.02308
H   5.24538 -2.69323  1.57053
H   3.82561 -2.54917 -1.00386
```

4ab

SCF (BP86) Energy = -805.049542579
Enthalpy 0K = -804.762081
Energy 298K = -804.745690
Free Energy 298K = -804.807495
Lowest Frequency = 26.6490 cm^{-1}
SCF (DCE) Energy = -805.055886626
SCF (BP86-D3) Energy = -805.086856679
SCF (BS2) Energy = -805.242809310

```
C   1.60630  1.51728 -0.05228
C   1.91829  2.87769  0.09147
C   0.26656  1.10545 -0.31936
C   0.88588  3.82299 -0.03771
H   2.94529  3.19632  0.29556
C  -0.75072  2.05220 -0.45738
C  -0.43220  3.41802 -0.31066
H   1.11382  4.88850  0.07111
H  -1.77800  1.74269 -0.67123
H  -1.22132  4.16976 -0.41394
C   3.70376 -0.22634  0.20196
C   3.51722 -1.63477  0.06609
H   4.63180  0.30150  0.41284
N   1.60502 -0.75530 -0.23467
N   2.22243 -1.95404 -0.19887
C   2.43157  0.31594  0.00478
C   0.18953 -0.41871 -0.42574
H  -0.38945 -2.20083  0.61538
C  -0.69431 -1.13863  0.63306
H  -0.43524 -0.74168  1.62955
C   4.54515 -2.72499  0.17619
H   4.06403 -3.70489  0.03483
H   5.33743 -2.61589 -0.58547
H   5.03848 -2.72010  1.16415
H  -0.13463 -0.73289 -1.43652
C  -2.18265 -1.01162  0.37479
C  -2.99172 -0.17253  1.16774
C  -2.79018 -1.73238 -0.67588
C  -4.36875 -0.05369  0.91824
H  -2.53515  0.39007  1.98999
C  -4.16451 -1.61343 -0.93043
H  -2.17941 -2.40217 -1.29337
C  -4.95895 -0.77218 -0.13343
H  -4.98095  0.59952  1.54921
H  -4.61781 -2.18423 -1.74789
```
